# Supplementary material for: 1,2-Amino oxygenation of alkenes with hydrogen evolution reaction
Source: Nat Commun. 2022 Jul 30;13:4430. doi: 10.1038/s41467-022-32084-8 (PMC9338937; doi:10.1038/s41467-022-32084-8)
Supplement: Supplementary file 1 — Supplementary Information [file 41467_2022_32084_MOESM1_ESM.pdf]

Supplementary Information

1,2-Amino Oxygenation of Alkenes with Hydrogen Evolution Reaction

Shengzhang Liu,[1,2,*] Shengchun Wang,[3,*] Pengjie Wang,[3] Zhiliang Huang,[3,✉] Tao Wang[1,✉] and Aiwen Lei[1,3,✉]

[1] National Research Center for Carbohydrate Synthesis and Jiangxi Province Key Laboratory of Chemical Biology, Jiangxi Normal University, Nanchang 330022, Jiangxi (P. R. China)

[2] College of Traditional Chinese Medicine, Jiangxi University of Chinese Medicine Nanchang 330022, Jiangxi (P. R. China)

[3] The Institute for Advanced Studies (IAS) and College of Chemistry and Molecular Sciences, Wuhan University, Wuhan, 430072, Hubei (P. R. China)

*These authors contributed equally to this work.

✉Corresponding author. Email: zlhhx@live.cn; wangtao@jxnu.edu.cn; aiwenlei@whu.edu.cn

Contents

Supplementary Methods 3
General information 3
Optimization of conditions 4
Experimental procedure A for 1,2-aminoxygenation with 2,2,2-trifluoroethanol 6
Experimental procedure B for 1,2-aminoxygenation with HOAc 6
Experimental procedure C for 1,2-aminoxygenation with acids 7
Experimental procedure D for 1,2-aminoxygenation with alcohols 7
Experimental procedure E for 1,2-aminoxygenation with pyrazoles 8
Experimental procedures for scale-up reaction in undivided cell 8
Experimental procedure scale-up reaction in continuous-flow electro-reactor 9
Experimental procedure for applications in pharmaceuticals 10
Mechanistic studies 12
Procedure for NMR experiments 12
Procedures for competitive experiment 13
Procedures for the radical clock experiments 13
Experimental procedure for the detection and quantification of hydrogen 14
Experimental procedure for cyclic voltammetry studies 15
Scope of molecules that divided from natural products or drugs 18
Characterization of products 19
Copies of product NMR spectra 54
Supplementary References 154

Supplementary Methods

General information

All glasswares were oven dried at 110 °C for hours and cooled down under vacuum. **5a–5l,**[1] **5m,**[2] **5n–5q,**[3] **5r,**[4] **5s,**[5] **5t,**[6] **10,**[1] and **11**[7] were prepared according to reported procedures. Unless otherwise noted, materials were obtained from commercial suppliers and used without further purification. The instrument for electrolysis is dual display potentiostat (DJS-292B). The anodic electrode was graphite rod (ϕ 6 mm, hard) and cathodic electrode was platinum sheet (1.5 cm × 1.5 cm × 0.3 mm). In continuous flow gram-scale experiment, the anodic electrode was carbon paper (9.3 cm × 9.3 cm × 0.2 mm) and cathodic electrode was platinum plate (9.3 cm × 9.3 cm × 0.3 mm). The pump used in the experiment is a KCS PRO model peristaltic pump manufactured by Kamoer. Thin layer chromatography (TLC) employed glass 0.25 mm silica gel plates. Flash chromatography columns were packed with 200-300 mesh silica gel in petroleum (bp. 60-90 °C). ^{1}H, ^{13}C and ^{19}F NMR data were recorded with ADVANCE III 400 MHz with tetramethylsilane as an internal standard. All chemical shifts (δ) are reported in ppm and coupling constants (*J*) in Hz. All chemical shifts were reported relative to tetramethylsilane (0 ppm for ^{1}H), $CDCl_3$ (77.0 ppm for ^{13}C) respectively. High resolution mass spectra (HRMS) were measured with a Waters Micromass GCT instrument and accurate masses were reported for the molecular hydrogen ion $(M+H)^+$ or $(M+Na)^+$.

Optimization of conditions

Supplementary Table 1. Optimization of the electro-oxidative 1,2-amino oxygenation with TFE.

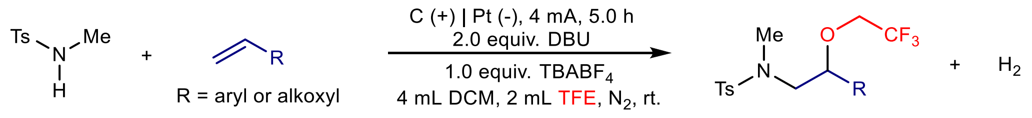

Entry	Variation from the standard conditions	Yield of **4**
1	none	76%
2	NO DBU	n.d.
3	K_3PO_4 instead of DBU	55%
4	Cs_2CO_3 instead of DBU	38%
5	DCM (6 mL) used as solvent	trace
6	TFE (6 mL) used as solvent	trace
7	HFIP (6 mL) used as solvent	n.d.
8	EtOH (6 mL) used as solvent	trace[b]
9	HOAc (6 mL) used as solvent	n.d.
10	*n*-Bu_4NPF_6 instead of *n*-Bu_4NBF_4	74%
11	$LiClO_4$ instead of *n*-Bu_4NBF_4	63%
12	2 mA, 10 h	74%
13	10 mA, 2.5 h	70%
14	C cloth instead of Carbon rod	73%
15	C felt instead of Carbon rod	8%
16	Carbon rod as cathode	66%
17	Ni plate as cathode	67%
18	reaction in the air	60%
19	without current	n.d.

[a] Reaction conditions: graphite rod anode (Φ 6 mm), Pt plate cathode (1.5 cm x 1.5 cm x 0.3 mm), constant current =4 mA, 1a (0.30 mmol), 2a (0.90 mmol), DBU (0.60 mmol), *n*-Bu_4NBF_4 (0.30 mmol), DCM/TFE (4/2 mL), N_2, rt, 5 h, undivided cell. Isolated yields. n.d. = no 1,2-amino oxygenation product was detected. [b]trace 1,2-amino oxygenation product was detected.

Supplementary Table 2. Optimization of the electro-oxidative 1,2-amino oxygenation with MeOH.

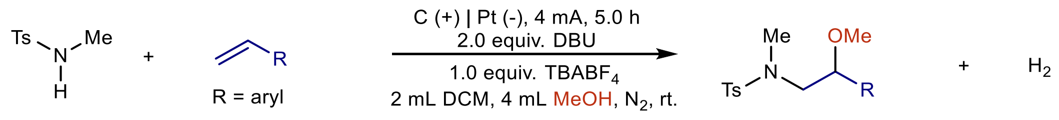

Entry	Variation from the standard conditions	Yields[b] of 6
1	none	60%
2	DCM/MeOH = 5 mL/0.5 mL	21%
3	DCM/MeOH = 5 mL/1 mL	35%
4	DCM/MeOH = 4.5 mL/1.5 mL	39%
5	DCM/MeOH = 4 mL/2 mL	40%
6	DCM/MeOH = 3 mL/3 mL	48%
7	DCM/MeOH = 1 mL/5 mL	56%
8	MeCN/MeOH = 2 mL/4 mL	16%
9	THF/MeOH = 2 mL/4 mL	trace
10	DMSO/MeOH = 2 mL/4 mL	7%
11	DMF/MeOH = 2 mL/4 mL	4%
12	MeOH only	52%
13	*n*-BuOK instead of DBU	5%
14	Cs_2CO_3 instead of DBU	9%
15	*n*-Bu_4NI instead of *n*-Bu_4NBF_4	n.d.[c]
16	Ni plate as cathode	33%
17	10 mA, 2.5 h	50%
18	without current	n.d.

[a] Reaction conditions: graphite rod anode (Φ 6 mm), Pt plate cathode (1.5 cm x 1.5 cm x 0.3 mm), constant current =4 mA, 1a (0.30 mmol), 2a (0.90 mmol), DBU (0.60 mmol), *n*-Bu_4NBF_4 (0.30 mmol), DCM/TFE (2/4 mL), N_2, rt, 5 h, undivided cell. [b] Isolated yields. [c] N.D. = not detected.

Supplementary Table 3. Optimization of the equivalents of DBU.

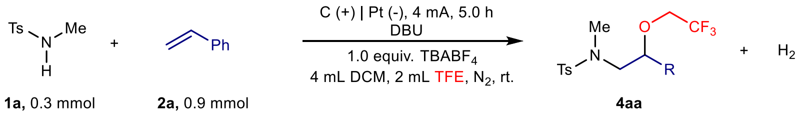

Entry	Equivalent of DBU	Isolated yield of **4aa**
1	0	N.d.
2	0.5	12%
3	1.0	41%
4	1.5	64%
5	2.0	76%

[a] Reaction conditions: graphite rod anode (Φ 6 mm), Pt plate cathode (1.5 cm x 1.5 cm x 0.3 mm), constant current =4 mA, 1a (0.30 mmol), 2a (0.90 mmol), DBU, n-Bu_4NBF_4 (0.30 mmol), DCM/TFE (2/4 mL), N_2, rt, 5 h, undivided cell. [b] Isolated yields. [c] N.D. = not detected.

Experimental procedure A for 1,2-aminoxygenation with 2,2,2-trifluoroethanol

In an oven-dried undivided three-necked bottle (10 mL) equipped with a stir bar, Sulfonamide substrate **1a** (0.3 mmol) and $^nBu_4NBF_4$ (0.3 mmol) were combined and added. The undivided cell was equipped with graphite rod anode (ф 6 mm), platinum plate cathode (1.5 cm × 1.5 cm × 0.3 mm) and was then charged with nitrogen. Under the atmosphere of nitrogen, 1,8-Diazabicyclo[5.4.0]undec-7-ene (DBU) (0.6 mmol), alkenes **2** (R=aryl, 0.9 mmol; R=alkoxyl, 1.8 mmol) were added, then Dichloromethane (DCM) (4.0 mL) and 2,2,2-Trifluoroethanol (TFE) (2.0 mL) were injected respectively into the tubes via syringes. The mixture was electrolyzed using constant current conditions (4.0 mA) for 5 h at room temperature under magnetic stirring. When TLC analysis indicated that the electrolysis was complete (witnessed by the disappearance of the **1a**), the solvent was removed under reduced pressure. The residue was purified by column chromatography on silica gel using a mixture of PE/EA (v: v = 25:1) as eluent to afford the desired pure product.

Experimental procedure B for 1,2-aminoxygenation with HOAc

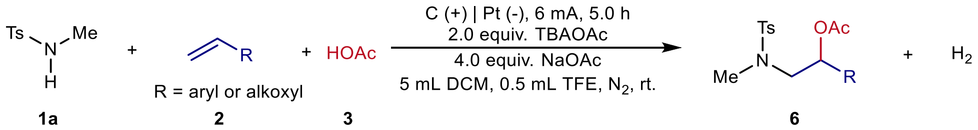

In an oven-dried undivided three-necked bottle (10 mL) equipped with a stir bar, sulfonamide substrate **1a** (0.3 mmol), TBAOAc (0.6 mmol) and NaOAc (1.2 mmol) were combined and added. The undivided cell was equipped with graphite rod anode (ф 6 mm), platinum plate cathode (1.5 cm × 1.5 cm × 0.3 mm) and was then charged with nitrogen. Under the atmosphere of nitrogen, alkenes **2** (1.8 mmol) and **Acetic acid 36% aqueous solution (AR, HOAc)** (1.0 mL) were added, then DCM (5.0 mL) and

TFE (0.5 mL) were injected respectively into the tubes via syringes. The mixture was electrolyzed using constant current conditions (6.0 mA) for 5 h at room temperature under magnetic stirring. When TLC analysis indicated that the electrolysis was completed (witnessed by the disappearance of the **1a**). The residue was then extracted with DCM (3×5 mL), dried over Na_2SO_4, and concentrated in vacuo. The residue was purified by column chromatography on silica gel using a mixture of PE/EA (v: v = 12:1) as eluent to afford the desired pure product.

Experimental procedure C for 1,2-aminoxygenation with acids

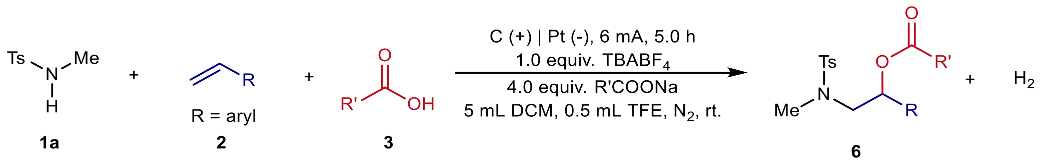

In an oven-dried undivided three-necked bottle (10 mL) equipped with a stir bar, sulfonamide substrate **1a** (0.3 mmol), $^{n}Bu_4NBF_4$ (0.3 mmol) and R′COONa (1.2 mmol) were combined and added. The undivided cell was equipped with graphite rod anode (ф 6 mm), platinum plate cathode (1.5 cm × 1.5 cm × 0.3 mm) and was then charged with nitrogen. Under the atmosphere of nitrogen, alkenes **2** (1.8 mmol) and R′ COOH (1.8 mmol) were added, then DCM (5.0 mL) and TFE (0.5 mL) were injected respectively into the tubes via syringes. The mixture was electrolyzed using constant current conditions (6.0 mA) for 5 h at room temperature under magnetic stirring. When the reaction was finished, the solvent was removed under reduced pressure. The residue was purified by column chromatography on silica gel using a mixture of PE/EA (v: v = 12:1) as eluent to afford the desired pure product.

Experimental procedure D for 1,2-aminoxygenation with alcohols

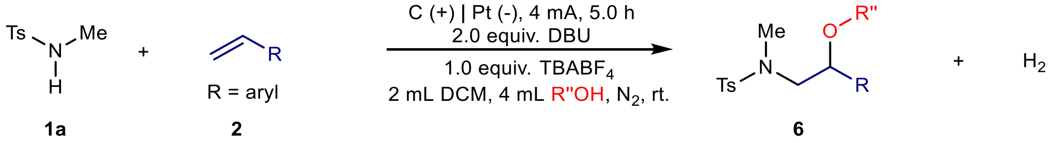

In an oven-dried undivided three-necked bottle (10 mL) equipped with a stir bar, sulfonamide substrate **1a** (0.3 mmol) and $^{n}Bu_4NBF_4$ (0.3 mmol) were combined and added. The undivided cell was equipped with graphite rod anode (ф 6 mm), platinum plate cathode (1.5 cm × 1.5 cm × 0.3 mm) and was then charged with nitrogen. Under the atmosphere of nitrogen, DBU (0.6 mmol), alkenes **2** (0.9 mmol) were added, then DCM (2.0 mL) and R”OH (4.0 mL) were injected respectively into the tubes via syringes. The mixture was electrolyzed using constant current conditions (4.0 mA) for 5 h at room temperature under magnetic stirring. When the reaction was finished, the solvent was removed under reduced pressure. The residue was purified by column

chromatography on silica gel using a mixture of PE/EA (v: v = 20:1) as eluent to afford the desired pure product.

Experimental procedure E for 1,2-aminoxygenation with pyrazoles

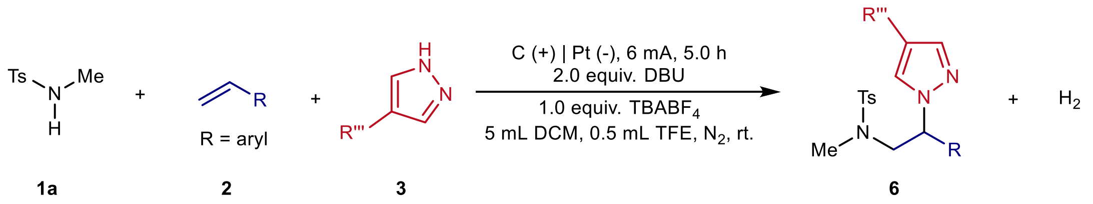

In an oven-dried undivided three-necked bottle (10 mL) equipped with a stir bar, sulfonamide substrate **1a** (0.3 mmol), $^{n}Bu_4NBF_4$ (0.3 mmol) and pyrazole derivatives **3** (1.8 mmol) were combined and added. The undivided cell was equipped with graphite rod anode (ϕ 6 mm), platinum plate cathode (1.5 cm × 1.5 cm × 0.3 mm) and was then charged with nitrogen. Under the atmosphere of nitrogen, DBU (0.6 mmol), alkenes **2** (1.8 mmol) were added, then DCM (5.0 mL) and TFE (0.5 mL) were injected respectively into the tubes via syringes. The mixture was electrolyzed using constant current conditions (6.0 mA) for 5.0 h at room temperature under magnetic stirring. When the reaction was finished, the solvent was removed under reduced pressure. The residue was purified by column chromatography on silica gel using a mixture of PE/EA (v: v = 15:1) as eluent to afford the desired pure product.

Experimental procedures for scale-up reaction in undivided cell

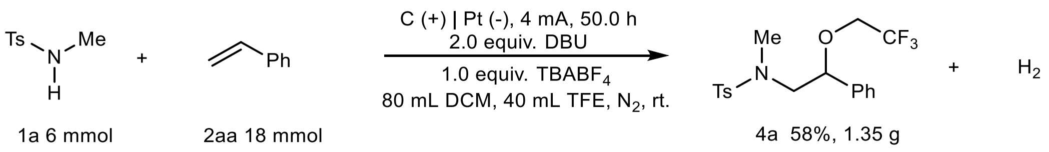

In an oven-dried undivided three-necked bottle (250 mL) equipped with a stir bar, *N*-methyl-*p*-toluenesulfonamide (6.0 mmol) and $^{n}Bu_4NBF_4$ (6.0 mmol) were combined and added. The undivided cell was equipped with graphite rod anode (ϕ 6 mm), platinum plate cathode (1.5 cm × 1.5 cm × 0.3 mm) and was then charged with nitrogen. Under the atmosphere of nitrogen, DBU (12.0 mmol), alkenes **2** (18 mmol) were added, then DCM (80.0 mL) and TFE (40.0 mL) were injected respectively into the tubes via syringes. The mixture was electrolyzed using constant current conditions (4.0 mA) for 50.0 h at room temperature under magnetic stirring. When the reaction was finished, the solvent was removed under reduced pressure. The residue was purified by column chromatography on silica gel using a mixture of PE/EA (v: v = 35:1) as eluent to afford the desired pure product. Isolated yield: 58 %, 1.35 g.

Experimental procedure scale-up reaction in continuous-flow electro-reactor

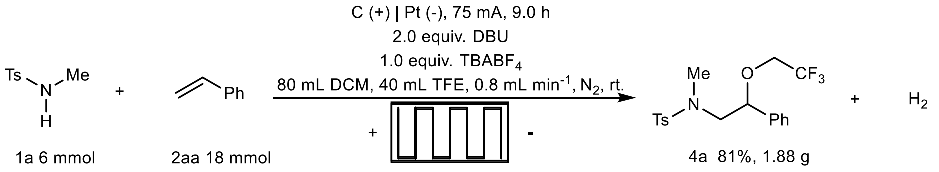

In an oven-dried three-necked bottle (250 mL) equipped with a stir bar, *N*-Methyl-*p*-toluenesulfonamide **1a** (6.0 mmol) and $^{n}Bu_4NBF_4$ (6.0 mmol) were combined and added. The flow cell was equipped with carbon paper (9.3 cm × 9.3 cm × 0.2 mm) as the anode (contact area 1.6 cm^2) and platinum plate (9.3 cm × 9.3 cm × 0.3 mm) as the cathode (contact area 1.6 cm^2). In order to preclude the possibility that air was involved, we flushed the whole system with nitrogen before the direct electrolysis. DBU (12.0 mmol), alkenes **2aa** (18 mmol) were added, then DCM (80.0 mL) and TFE (40.0 mL) were injected respectively into the tubes via syringes. The reaction mixture was pumped into the electrochemical reactor in a flow rate of 0.8 mL min^{-1} (24 r/min). A constant current of 75 mA was employed during the electrolysis under room temperature for 9 h. When the reaction was finished, the solvent was removed under reduced pressure. The residue was purified by column chromatography on silica gel using a mixture of PE/EA (v: v = 35:1) as eluent to afford the desired pure product. Isolated yield: 81 %, 1.88 g.

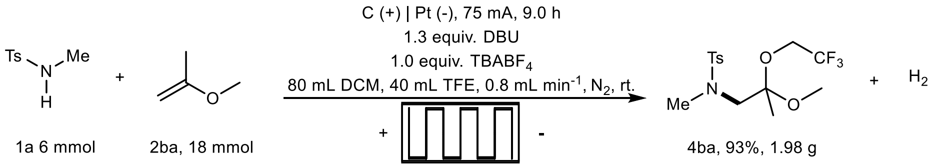

In an oven-dried three-necked bottle (250 mL) equipped with a stir bar, *N*-Methyl-*p*-toluenesulfonamide **1a** (6.0 mmol) and $^{n}Bu_4NBF_4$ (6.0 mmol) were combined and added. The flow cell was equipped with carbon paper (9.3 cm × 9.3 cm × 0.2 mm) as the anode (contact area 1.6 cm^2) and platinum plate (9.3 cm × 9.3 cm × 0.3 mm) as the cathode (contact area 1.6 cm^2). In order to preclude the possibility that air was involved, we flushed the whole system with nitrogen before the direct electrolysis. DBU (7.8 mmol), alkenes **2 ba** (18 mmol) were added, then DCM (80.0 mL) and TFE (40.0 mL) were injected respectively into the tubes via syringes. The reaction mixture was pumped into the electrochemical reactor in a flow rate of 0.8 mL min^{-1} (24 r/min). A constant current of 75 mA was employed during the electrolysis under room temperature for 9 h. When the reaction was finished, the solvent was removed under reduced pressure. The residue was purified by column chromatography on silica gel using a mixture of PE/EA (v: v = 18:1) as eluent to afford the desired pure product. Isolated yield: 93 %, 1.98 g.

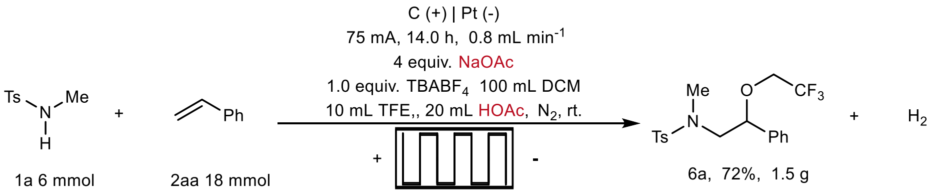

In an oven-dried three-necked bottle (250 mL) equipped with a stir bar, *N*-Methyl-*p*-toluenesulfonamide **1a** (6.0 mmol) and ^{n}Bu4NBF4 (6.0 mmol), NaOAc (24.0 mmol) were combined and added. The flow cell was equipped with carbon paper (9.3 cm × 9.3 cm × 0.2 mm) as the anode (contact area 1.6 cm^2) and platinum plate (9.3 cm × 9.3 cm × 0.3 mm) as the cathode (contact area 1.6 cm^2). In order to preclude the possibility that air was involved, we flushed the whole system with nitrogen before the direct electrolysis. alkenes **2 aa** (18 mmol) were added, then DCM (100.0 mL), TFE (10.0 mL) and HOAc (20.0 mL) were injected respectively into the tubes via syringes. The reaction mixture was pumped into the electrochemical reactor in a flow rate of 0.8 mL min^{-1} (24 r/min). A constant current of 75 mA was employed during the electrolysis under room temperature for 14 h. When the reaction was finished, the solvent was removed under reduced pressure. The residue was purified by column chromatography on silica gel using a mixture of PE/EA (v: v = 15:1) as eluent to afford the desired pure product. Isolated yield: 72 %, 1.5 g.

Experimental procedure for applications in pharmaceuticals

The synthesis of halostachine

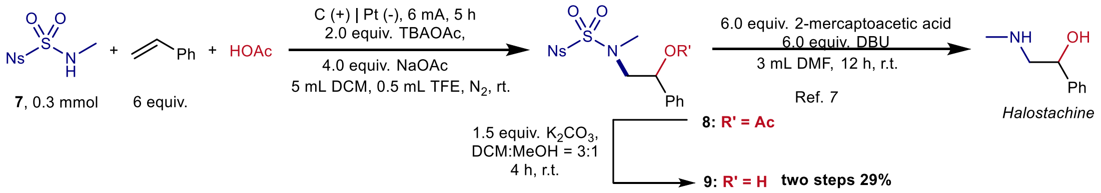

Supplementary Figure 1. The synthesis of halostachine.

Step 1| Procedure for 2-((N-methyl-4-nitrophenyl)sulfonamido)-1-phenylethyl acetate **8**:

In an oven-dried undivided three-necked bottle (10 mL) equipped with a stir bar, sulfonamide substrate **1a** (0.2 mmol), TBAOAc (0.4 mmol) and NaOAc (0.8 mmol) were combined and added. The undivided cell was equipped with graphite rod anode (ф 6 mm), platinum plate cathode (1.5 cm × 1.5 cm × 0.3 mm) and was then charged with nitrogen. Under the atmosphere of nitrogen, alkenes **2** (0.6 mmol) and **Acetic acid 36% aqueous solution (AR, HOAc)** (1.0 mL) were added, t then DCM (5.0 mL) and TFE (0.5 mL) were injected respectively into the tubes via syringes. The mixture was electrolyzed using constant current conditions (6.0 mA) for 5 h at room temperature under magnetic stirring. The residue was then extracted with DCM (3×5 mL), dried

over Na_2SO_4, and concentrated in vacuo and used in the next step without further purification.

Step 2| Procedure for *N*-(2-hydroxy-2-phenylethyl)-*N*-methyl-4-nitrobenzenesulfonamide **9**:

Potassium carbonate (42 mg, 0.30 mmol) was added to a solution of crude product **8** in CH_2Cl_2 (1.5 mL) and MeOH (0.5 mL) and stirred at room temperature for 4 h. Concentrated under reduced pressure to give the crude product. Purification by silica gel flash column chromatography PE/EA (v: v = 2:1) gave the desired product **9** (two step, 14 mg, 29%), as a colorless oil. Spectral data are in accordance with those reported in literature [7].

Step 3| Procedure for halostachine:

Based on a known literature procedure [7], a round-bottom flask equipped with a magnetic stir bar was charged with DBU (1.2 mmol, 6.0 equiv.), 2-mercaptoacetic acid (1.2 mmol, 6.0 equiv.) and DMF (2.0 mL). A solution of *N*-(2-hydroxy-2-phenylethyl)-N-methyl-4-nitrobenzenesulfonamide (67.3 mg, 0.2 mmol, 1.0 equiv) in DMF (1.0 mL) was added dropwise and the resulting reaction mixture was stirred for 12 h at room temperature.

Mechanistic studies

Procedure for NMR experiments

The *N*-Methyl-*p*-toluenesulfonamide (37.05 mg, 0.20 mmol) was dissolved in 2 mL $CDCl_3$, which called Solution A. 1,8-Diazabicyclo [5.4.0] undec-7-ene (DBU) of different equivalents with 0.5mL of A solution were mixed into an NMR tube, and made 1 H NMR test. 1) 500 μL of A. 2) 500 μL of A, 0.5 equiv. DBU. 3) 500 μL of A, 1.0 equiv. DBU. 4) 500 μL of A, 2.0 equiv. DBU.

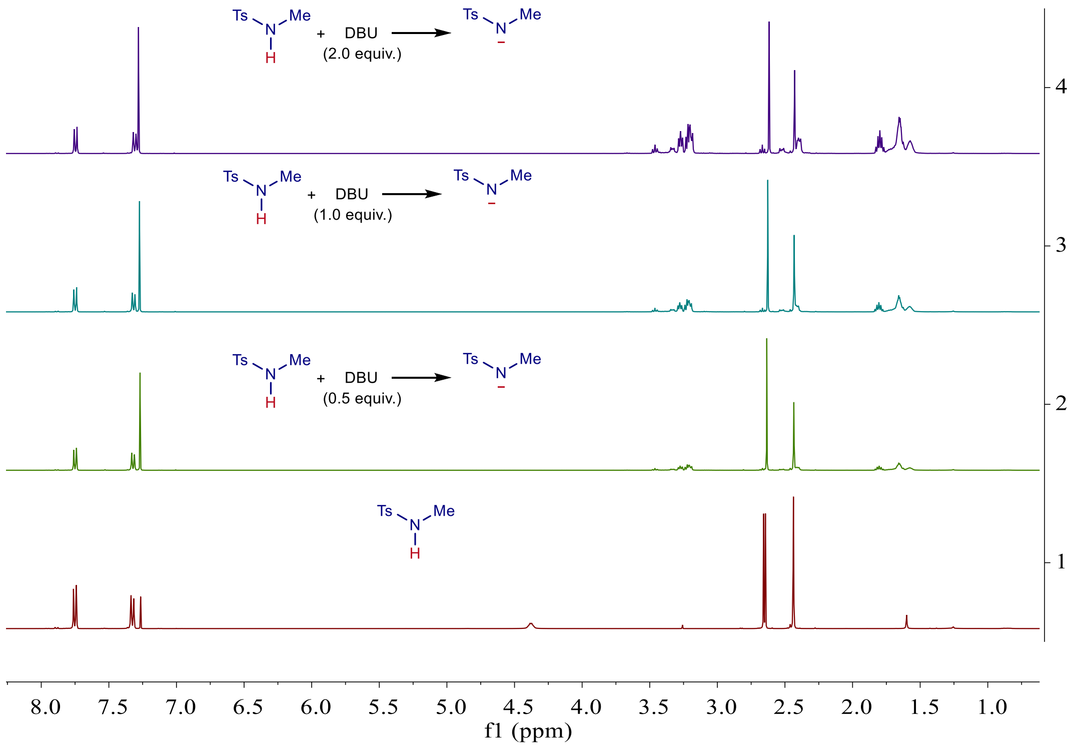

Supplementary Figure 2. NMR experiments of sulfonamide with the addition of DBU.

Procedures for competitive experiment

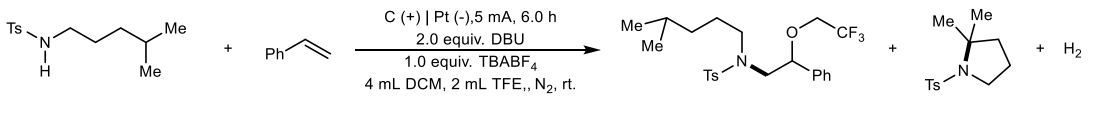

10 0.3 mmol **2aa** 0.9 mmol **10a** 15% **10b** 41%

Supplementary Figure 3. Competitive experiment.

The sulfonamide substrate **10** (0.3 mmol) and $^{n}Bu_4NBF_4$ (0.3 mmol) were combined and added in an oven-dried undivided three-necked bottle (10 mL) equipped with a stir bar, Then the undivided cell was equipped with graphite rod anode (ф 6 mm), platinum plate cathode (1.5 cm × 1.5 cm × 0.3 mm). Under a nitrogen atmosphere, DBU (0.6 mmol), Styrene (0.9 mmol) were added, then DCM (4.0 mL) and TFE (2.0 mL) were injected respectively into the tubes via syringes. The mixture was electrolyzed using constant current conditions (5.0 mA) for 6 h at room temperature under magnetic stirring. When the reaction was finished, the solvent was removed under reduced pressure. The residue was purified by column chromatography on silica gel using a mixture of PE/EA (v: v = 25:1) gave the desired product **10a** (21 mg, 15%) and **10b** (31 mg, 41%), both as a colorless oil.

Procedures for the radical clock experiments

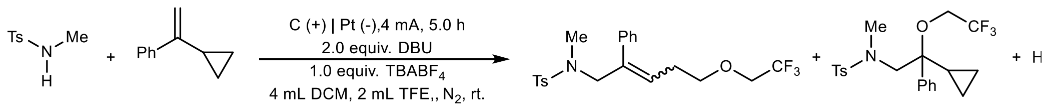

1a 0.3 mmol **11** 0.9 mmol **11a** 14% **11b** 6%

Supplementary Figure 4. Radical clock experiments.

The compound **1a** (0.3 mmol) and $^{n}Bu_4NBF_4$ (0.3 mmol) were combined and added in an oven-dried undivided three-necked bottle (10 mL) equipped with a stir bar, Then the undivided cell was equipped with graphite rod anode (ф 6 mm), platinum plate cathode (1.5 cm × 1.5 cm × 0.3 mm). Under a nitrogen atmosphere, DBU (0.6 mmol), alkenes **11** (0.9 mmol) were added, then DCM (4.0 mL) and TFE (2.0 mL) were injected respectively into the tubes via syringes. The mixture was electrolyzed using constant current conditions (4.0 mA) for 5 h at room temperature under magnetic stirring. When the reaction was finished, the solvent was removed under reduced pressure. The residue was purified by column chromatography on silica gel using a mixture of PE/EA (v: v = 20:1) gave the desired product **11a** (18 mg, 14%) and **11b** (7.7 mg, 6%), both as a colorless oil.

Experimental procedure for the detection and quantification of hydrogen

After the operation of the standard condition, 4 mL methane was injected into reaction tube. Then, the tube shook to mix up the gas for GC detection.

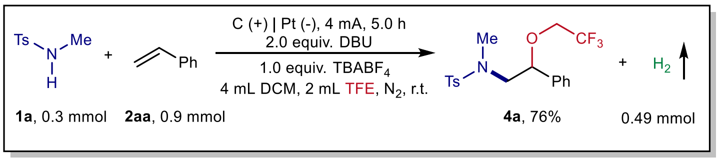

Standard curve between methane and hydrogen as follow:

Volume of hydrogen	Peak area	Volume of methane	Peak area
0	0	4	382437
2	570479	4	352352
4	1101022	4	320472
6	1900810	4	345025
8	2364625	4	329597
10	2670934	4	313532
12	3414752	4	329899
14	3677295	4	335308
Reaction	2693827	4	294283

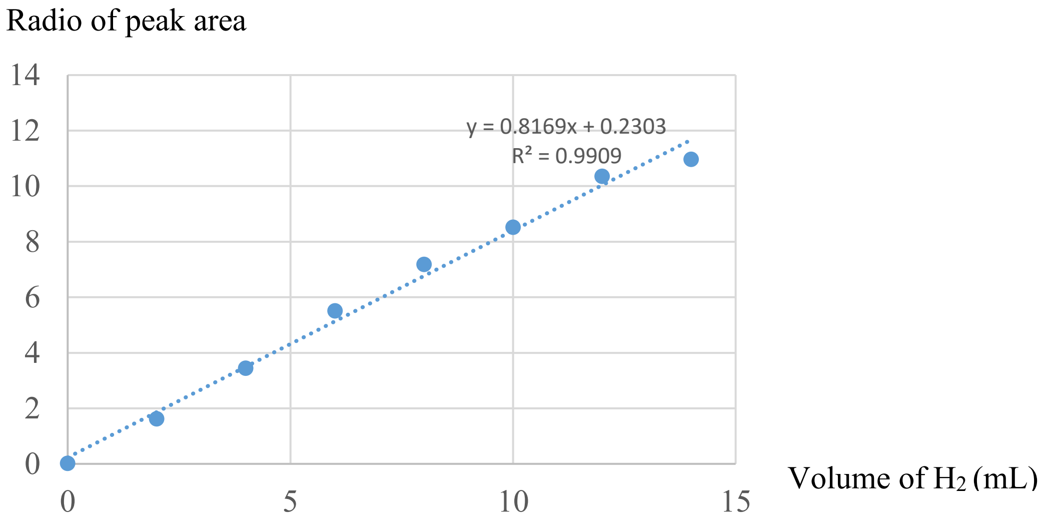

Supplementary Table 4. Quantitative analysis of hydrogen evolution monitored by GC.

Experimental procedure for cyclic voltammetry studies

Cyclic voltammetry experiment was performed in a three-electrode cell connected to an undivided three-necked bottle with stir bar under nitrogen at room temperature. The working electrode was a glass carbon electrode, the counter electrode a platinum wire. The reference was an Ag/AgCl electrode submerged in saturated aqueous KCl solution. After adding 0.60 mmol $TBABF_4$, 0.06 mmol substrates, exchange the air into N_2, then liquid substrates were added, finally anhydrous degassed 4 mL DCM and 2 mL TFE were injected into the electrochemical cell in all experiments. The scan rate 0.1 V/s, voltage range was 0 V ~ 2.5 V. Data was analyzed using Origin by subtracting a background current prior to identifying the maximum current (Cp) and determining the potential (Ep) at this value (Cp). The oxidation peak of DCM : TFE (4 mL : 2 mL), $TBABF_4$ (0.1 M) were not observed.

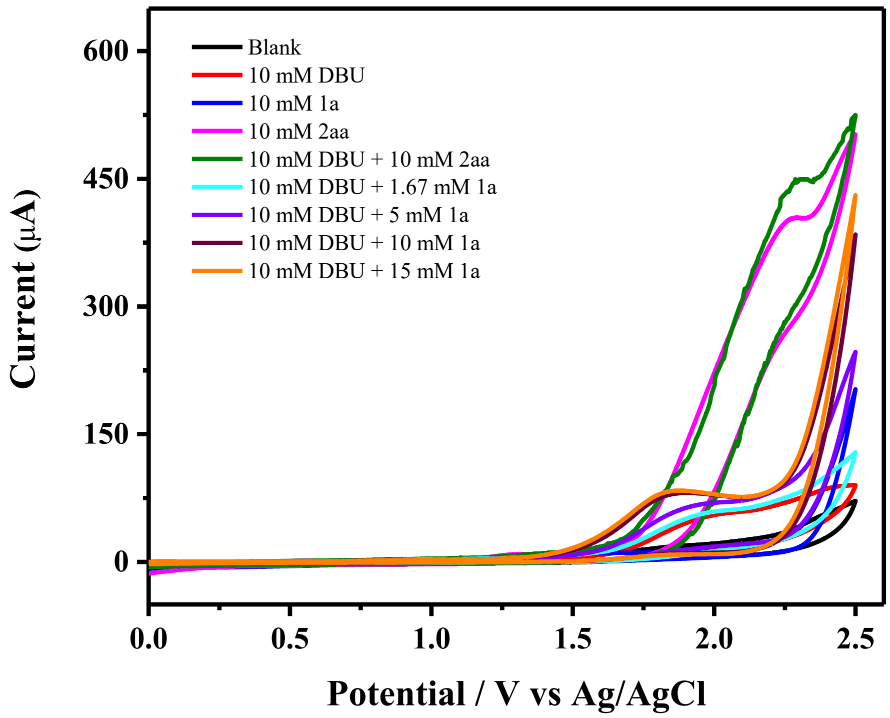

Supplementary Figure 5. Cyclic voltammetry studies for exploring the reaction in the anode. Test conditions: A cyclic voltammograms in solvent (6 mL) by using glassy carbon as the working electrode, Pt wire as the counter electrode and Ag/AgCl as the reference electrode under N_2 at room temperature. The scan rate is 0.1 V/s. Black line: 4 mL DCM and 2 mL TFE, $TBABF_4$ (0.1 M). Red line: 10 mM DBU. The oxidation peaks of red line were observed at 2.056 V (vs Ag/AgCl). Blue line: 10 mM *N*-methyl-*p*-toluenesulfonamide (**1a**). The oxidation peaks of blue line were not observed. Violet line: 10 mM styrene (**2aa**). The oxidation peaks of violet line were observed at 2.268

V (vs Ag/AgCl). Green line: 10 mM DBU, 10 mM **2aa**. The oxidation peaks of green line were observed at 2.296 V (vs Ag/AgCl). Turquoise line: 10 mM DBU, 1.67 mM **1a**. The oxidation peaks of turquoise line were observed at 2.000 V (vs Ag/AgCl). Lavender line: 10 mM DBU, 5 mM **1a**. The oxidation peaks of lavender line were observed at 1.987V (vs Ag/AgCl). Dark purple line: 10 mM DBU, 10 mM **1a**. The oxidation peaks of dark purple line were observed at 1.884 V (vs Ag/AgCl). Orange line: 10 mM DBU, 15 mM **1a**. The oxidation peaks of orange line were observed at 1.876 V (vs Ag/AgCl).

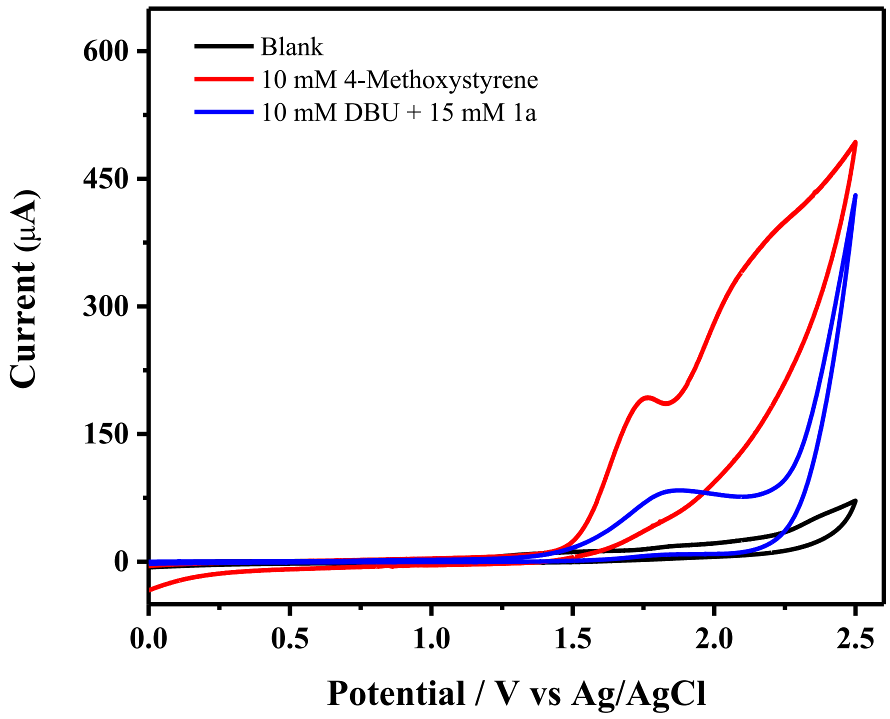

Supplementary Figure 6. Comparison of 4-methoxystyrene and the mixture of DBU and 1a. The cyclic voltammograms in solvent (6 mL) by using glassy carbon as the working electrode, Pt wire as the counter electrode and Ag/AgCl as the reference electrode under N_2 at room temperature. The scan rate is 0.1 V/s. Black line: 4 mL DCM and 2 mL TFE, $TBABF_4$ (0.1 M). Red line: 10 mM 4-Methoxystyrene. The oxidation peaks of red line were observed at 1.75 V (vs Ag/AgCl). Blue line: 10 mM DBU, 15 mM 1a (*N*-Methyl-*p*-toluenesulfonamide). The oxidation peaks of blue line line were observed at 1.85 V (vs Ag/AgCl).

Alkenes with electron-donating groups showed lower oxidation potentials than the mixture of DBU and amide **1a**, proving a prioritized oxidation of alkenes in the anode. Therefore, with these alkenes, the formation of NCRs might be suppressed, causing the low yields in this electro-chemical oxygen amination.

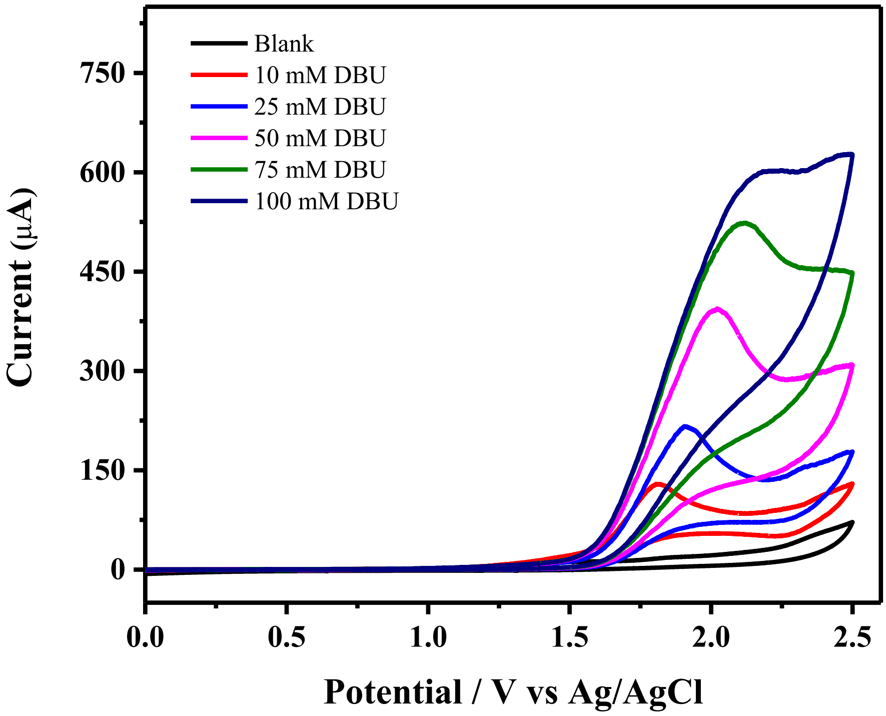

Supplementary Figure 7. Cyclic voltammetry studies of DBU at different concentrations. Test conditions: A cyclic voltammograms in solvent (6 mL) by using glassy carbon as the working electrode, Pt wire as the counter electrode and Ag/AgCl as the reference electrode under N_2 at room temperature. The scan rate is 0.1 V/s. Black line: 4 mL DCM and 2 mL TFE, $TBABF_4$ (0.1 M). Red line: 10 mM DBU. Blue line: 25 mM DBU. Violet line: 50 mM DBU. Green line: 75 mM DBU. Dark blue: 100 mM DBU. The oxidation peak of DBU were observed at 1.818 V, 1.912V, 2.03, 2.11, 2.20 V (vs Ag/AgCl).

Scope of molecules that divided from natural products or drugs

To examine whether this synthetic strategy is suitable for the late-stage functionalization of complex molecules, several molecules that divided from natural products or drugs have been tested in standard conditions. However, only menthol and indanol derivatives were tolerated in standard conditions, forming desired products in low yields. In addition, molecules from diacetone-D-glucose or cholesterol were failed to afford corresponding products. Combined our previous work, these results may cause by the feature of NCRs that have a potential to react with C(sp^3)-H bond via a HAT progress.

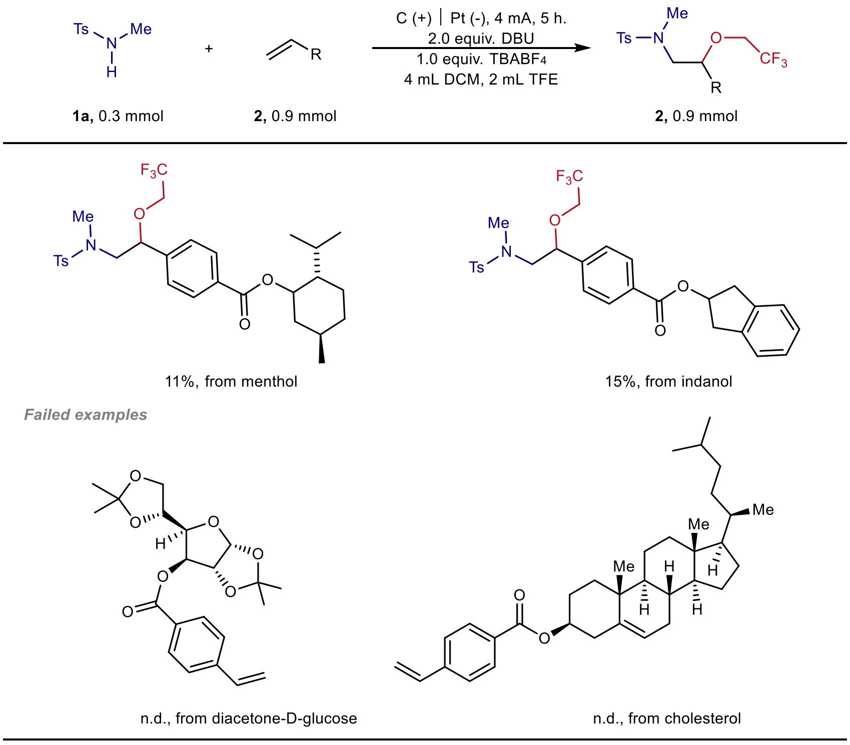

Supplementary Figure 8. Scope of molecules that divided from natural products or drugs

Characterization of products

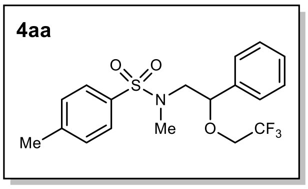

N,4-Dimethyl-N-(2-phenyl-2-(2,2,2-trifluoroethoxy)ethyl)benzenesulfonamide 4aa

The reaction was carried out following the procedure A using *N*-methyl-*p*-toluenesul - fonamide, styrene and 2,2,2-trifluoroethanol. Then 88.2 mg colorless oily liquid was obtained in 76% isolated yield following purification procedure A.

^{1}H NMR (400 MHz, $CDCl_3$) δ 7.65 (d, J = 8.0 Hz, 2H), 7.42–7.31 (m, 5H), 7.29 (d, J = 8.0 Hz, 2H), 4.72 (dd, J = 8.0, 4.0 Hz, 1H), 3.77–3.60 (m, 2H), 3.40 (dd, J = 12.0, 4.0 Hz, 1H), 3.11 (dd, J = 12.0, 4.0 Hz, 1H), 2.85 (s, 3H), 2.41 (s, 3H).

^{13}C NMR (101 MHz, $CDCl_3$) δ 143.44, 137.49, 135.11, 129.73, 128.97, 128.87, 127.25, 126.72, 126.51 (q, $J_{C\text{-}F}$ = 278.76 Hz), 84.06, 66.22 (q, J = 34.0 Hz), 56.44, 37.32, 21.50.

^{19}F NMR (377 MHz, $CDCl_3$) δ -74.19.

HRMS (ESI) calculated for $C_{19}H_{22}F_3NO_3S^+$, $[M+H]^+$: 388.11888; found: 388.11876.

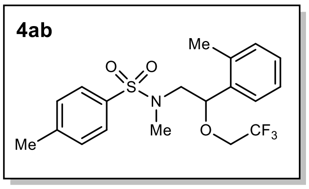

N,4-Dimethyl-N-(2-(o-tolyl)-2-(2,2,2-trifluoroethoxy)ethyl)benzenesulfonamide 4ab

The reaction was carried out following the procedure A using *N*-methyl-*p*-toluenesul - fonamide, 2-methylstyrene and 2,2,2-trifluoroethanol. Then 57.7 mg colorless oily liquid was obtained in 48% isolated yield following purification procedure A.

^{1}H NMR (400 MHz, $CDCl_3$) δ 7.65 (d, J = 8.0 Hz, 2H), 7.35–7.32 (m, 1H), 7.29 (d, J = 8.0 Hz, 2H), 7.25–7.20 (m, 3H), 5.01 (dd, J = 8.0, 4.0 Hz, 1H), 3.77–3.58 (m, 2H), 3.45 (dd, J = 16.0, 4.0 Hz, 1H), 2.96–2.90 (m, 4H), 2.42 (s, 3H), 2.41 (s, 3H).

^{13}C NMR (101 MHz, $CDCl_3$) δ143.52, 136.10, 135.56, 135.18, 130.93, 129.74, 128.41, 127.20, 126.59, 125.57, 126.55 (q, $J_{C\text{-}F}$ = 278.76 Hz), 81.24, 66.26 (q, $J_{C\text{-}F}$ = 34.4 Hz), 55.65, 37.51, 21.49, 19.10.

^{19}F NMR (377 MHz, $CDCl_3$) δ -74.16.

HRMS (ESI) calculated for $C_{19}H_{23}F_3NO_3S^+$, $[M+H]^+$: 402.13453; found: 402.13411.

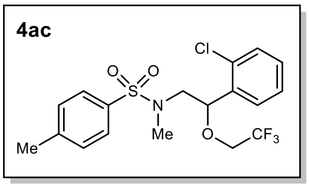

N-(2-(2-Chlorophenyl)-2-(2,2,2-trifluoroethoxy)ethyl)-N,4-dimethylbenzene-sulfonamide 4ac

The reaction was carried out following the procedure A using *N*-methyl-*p*-toluenesul - fonamide, 2-chlorostyrene and 2,2,2-trifluoroethanol. Then 55.6 mg colorless oily liquid was obtained in 44% isolated yield following purification procedure A.

^{1}H NMR (400 MHz, $CDCl_3$) δ 7.67 (d, J = 8.0 Hz, 2H), 7.45 (d, J = 4.0 Hz, 1H), 7.38 (d, J = 8.0 Hz, 1H), 7.34–7.28 (m, 4H), 5.16 (dd, J = 8.0, 4.0 Hz, 1H), 3.79–3.62 (m, 2H), 3.47 (dd, J = 16.0, 4.0 Hz, 1H), 3.07 (dd, J = 16.0, 8.0 Hz, 1H), 2.94 (s, 3H), 2.41 (s, 3H).

^{13}C NMR (101 MHz, $CDCl_3$) δ143.38, 135.20, 135.08, 133.05, 129.87, 129.83, 129.79, 129.68, 127.50, 127.36, 126.3 (q, $J_{C\text{-}F}$ = 279.77 Hz), 79.87, 66.86 (q, $J_{C\text{-}F}$ = 34.6 Hz), 54.76, 37.16, 21.49.

^{19}F NMR (377 MHz, $CDCl_3$) δ -74.24.

HRMS (ESI) calculated for $C_{18}H_{20}ClF_3NO_3S^+$, $[M+H]^+$: 422.07990; found: 422.07971.

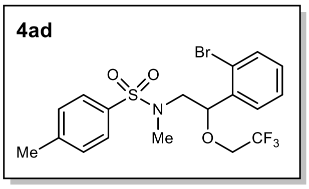

N-(2-(2-Bromophenyl)-2-(2,2,2-trifluoroethoxy)ethyl)-N,4-dimethylbenzene-sulfonamide 4ad

The reaction was carried out following the procedure A using *N*-methyl-*p*-toluenesul - fonamide, 2-bromostyrene and 2,2,2-trifluoroethanol. Then 83.7 mg colorless oily liquid was obtained in 60% isolated yield following purification procedure A.

^{1}H NMR (400 MHz, $CDCl_3$) δ 7.68 (d, J = 8.0 Hz, 2H), 7.56 (dd, J = 8.0, 4.0 Hz, 1H), 7.44 (dd, J = 8.0, 4.0 Hz, 1H), 7.37 (t, J = 7.6 Hz, 1H), 7.29 (d, J = 8.0 Hz, 2H), 7.23–7.18(m, 1H), 5.12 (dd, J = 12.0, 4.0 Hz, 1H), 3.79–3.61 (m, 2H), 3.47 (dd, J = 16.0, 4.0 Hz, 1H), 3.06 (dd, J = 16.0, 8.0 Hz, 1H), 2.95 (s, 3H), 2.41 (s, 3H).

^{13}C NMR (101 MHz, $CDCl_3$) δ143.37, 136.69, 135.20, 133.16, 130.14, 129.68, 128.12, 127.79, 127.38, 126.3 (q, $J_{C\text{-}F}$ = 279.8 Hz), 122.96, 81.91, 66.80 (q, $J_{C\text{-}F}$ = 34.6 Hz), 54.77, 37.14, 21.50.

^{19}F NMR (377 MHz, $CDCl_3$) δ -74.21.

HRMS (ESI) calculated for $C_{18}H_{20}BrF_3NO_3S^+$, $[M+H]^+$: 466.02939; found: 466.02932.

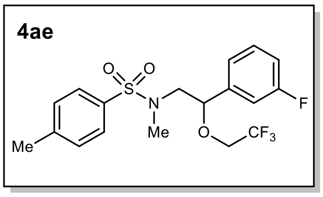

N-(2-(3-Fluorophenyl)-2-(2,2,2-trifluoroethoxy)ethyl)-N,4-dimethylbenzene-sulfonamide 4ae

The reaction was carried out following the procedure A using *N*-methyl-*p*-toluenesul - fonamide, 3-fluorostyrene and 2,2,2-trifluoroethanol. Then 80.2 mg colorless oily liquid was obtained in 66% isolated yield following purification procedure A.

^{1}H NMR (400 MHz, $CDCl_3$) δ 7.64 (d, J = 8.0 Hz, 2H), 7.40–7.33 (m, 1H), 7.30 (d, J = 8.0 Hz, 2H), 7.11 (d, J = 8.0 Hz, 1H), 7.07–7.01 (m, 2H), 4.72 (dd, J = 8.0, 4.0 Hz, 1H), 3.78–3.65 (m, 2H), 3.37 (dd, J = 16.0, 4.0 Hz, 1H), 3.07 (dd, J = 16.0, 8.0 Hz, 1H), 2.84 (s, 3H), 2.41 (s, 3H).

^{13}C NMR (101 MHz, $CDCl_3$) δ163.13 (d, $J_{C\text{-}F}$ = 247.6 Hz), 143.58, 140.23 (d, $J_{C\text{-}F}$ = 6.7 Hz), 134.94, 130.65 (d, $J_{C\text{-}F}$ = 8.1 Hz), 129.78, 127.23, 126.38 (q, $J_{C\text{-}F}$ = 278.76 Hz)，122.47 (d, $J_{C\text{-}F}$ = 2.9 Hz), 115.83 (d, $J_{C\text{-}F}$ = 21.2 Hz), 113.46 (d, $J_{C\text{-}F}$ = 22.0 Hz), 83.52, 66.48 (q, $J_{C\text{-}F}$ = 34.5 Hz), 56.36, 37.39, 21.50.

^{19}F NMR (377 MHz, $CDCl_3$) δ -74.21, -111.67.

HRMS (ESI) calculated for $C_{18}H_{20}F_4NO_3S^+$, $[M+H]^+$: 406.10945; found: 406.10923.

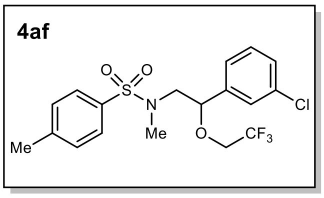

N-(2-(3-Chlorophenyl)-2-(2,2,2-trifluoroethoxy)ethyl)-N,4-dimethylbenzene-sulfonamide 4af

The reaction was carried out following the procedure A using *N*-methyl-*p*-toluenesul - fonamide, 3-chlorostyrene and 2,2,2-trifluoroethanol. Then 99.8 mg colorless oily liquid was obtained in 79% isolated yield following purification procedure A.

^{1}H NMR (400 MHz, $CDCl_3$) δ 7.64 (d, J = 8.0 Hz, 2H), 7.34–7.28 (m, 5H), 7.24–7.20 (m, 1H), 4.69 (dd, J = 8.0, 4.0 Hz, 1H), 3.79–3.62 (m, 2H), 3.37 (dd, J = 16.0, 4.0 Hz, 1H), 3.07 (dd, J = 12.0, 8.0 Hz, 1H), 2.85 (s, 3H), 2.41 (s, 3H).

^{13}C NMR (101 MHz, $CDCl_3$) δ 143.58, 139.71, 134.98, 134.94, 130.33, 129.79, 129.06, 127.23, 126.70, 124.93, 125.99 (q, $J_{C\text{-}F}$ = 279.77 Hz), 83.55, 66.51 (q, $J_{C\text{-}F}$ = 34.5 Hz), 56.36, 37.40, 21.51.

^{19}F NMR (377 MHz, $CDCl_3$) δ -74.18.

HRMS (ESI) calculated for $C_{18}H_{20}ClF_3NO_3S^+$, $[M+H]^+$: 422.07990; found: 422.08014.

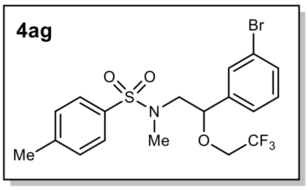

N-(2-(3-Bromophenyl)-2-(2,2,2-trifluoroethoxy)ethyl)-N,4-dimethylbenzene-sulfonamide 4ag

The reaction was carried out following the procedure A using *N*-methyl-*p*-toluenesul - fonamide, 3-bromostyrene and 2,2,2-trifluoroethanol. Then 75.3 mg colorless oily liquid was obtained in 54% isolated yield following purification procedure A.

^{1}H NMR (400 MHz, $CDCl_3$) δ 7.64 (d, J = 8.0 Hz, 2H), 7.50–7.46 (m, 1H), 7.46 (s, 1H), 7.32–7.27 (m, 4H), 4.68 (dd, J = 8.0, 4.0 Hz, 1H), 3.80–3.62 (m, 2H), 3.38 (dd, J = 16.0, 4.0 Hz, 1H), 3.07 (dd, J = 16.0, 8.0 Hz, 1H), 2.85 (s, 3H), 2.41 (s, 3H).

^{13}C NMR (101 MHz, $CDCl_3$) δ 143.56, 139.98, 135.02, 132.00, 130.59, 129.83, 129.62, 127.24, 126.4 (q, J_{C-F} = 279.77 Hz), 125.38, 123.12, 83.52, 66.54 (q, J_{C-F} = 34.6 Hz), 56.39, 37.39, 21.50.

^{19}F NMR (377 MHz, $CDCl_3$) δ -74.17.

HRMS (ESI) calculated for $C_{18}H_{20}BrF_3NO_3S^+$, $[M+H]^+$: 466.02939; found: 466.02997.

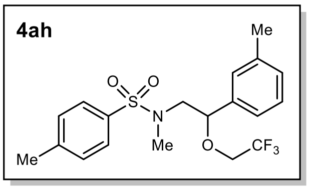

N,4-Dimethyl-N-(2-(m-tolyl)-2-(2,2,2-trifluoroethoxy)ethyl)benzenesulfonamide 4ah

The reaction was carried out following the procedure A using *N*-methyl-*p*-toluenesul - fonamide, 3-methylstyrene and 2,2,2-trifluoroethanol. Then 56.5 mg colorless oily liquid was obtained in 47% isolated yield following purification procedure A.

^{1}H NMR (400 MHz, $CDCl_3$) δ 7.65 (d, J = 8.0 Hz, 2H), 7.31–7.26 (m, 3H), 7.16 (d, J = 8.0 Hz, 1H), 7.12 (s, 2H), 4.67 (dd, J = 8.0, 4.0 Hz, 1H), 3.78–3.58 (m, 2H), 3.41 (dd, J = 16.0, 4.0 Hz, 1H), 3.08 (dd, J = 16.0, 8.0 Hz, 1H), 2.86 (s, 3H), 2.41 (s, 3H), 2.36 (s, 3H).

^{13}C NMR (101 MHz, $CDCl_3$) δ 143.37, 138.73, 137.43, 135.23, 129.70, 129.60, 128.85, 127.40, 127.25, 126.6 (q, J_{C-F} = 279.77 Hz), 123.71, 84.14, 66.17 (q, J_{C-F} = 34.4 Hz), 56.45, 37.28, 21.49, 21.42.

^{19}F NMR (377 MHz, $CDCl_3$) δ -74.17.

HRMS (ESI) calculated for $C_{19}H_{23}F_3NO_3S^+$, $[M+H]^+$: 402.13453; found: 402.13434.

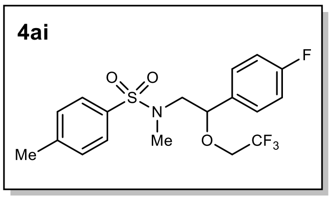

N-(2-(4-Fluorophenyl)-2-(2,2,2-trifluoroethoxy)ethyl)-N,4-dimethylbenzene-sulfonamide 4ai

The reaction was carried out following the procedure A using *N*-methyl-*p*-toluenesul - fonamide, 4-fluorostyrene and 2,2,2-trifluoroethanol. Then 92.3 mg colorless oily liquid was obtained in 76% isolated yield following purification procedure A.

^{1}H NMR (400 MHz, $CDCl_3$) δ 7.64 (d, J = 8.0 Hz, 2H), 7.33–7.28 (m, 4H), 7.10–7.04 (m, 2H), 4.70 (dd, J = 8.0, 4.0 Hz, 1H), 3.73–3.62 (m, 2H), 3.34 (dd, J = 16.0, 4.0 Hz, 1H), 3.09 (dd, J = 16.0, 8.0 Hz, 1H), 2.82 (s, 3H), 2.41 (s, 3H).

^{13}C NMR (101 MHz, $CDCl_3$) δ 162.92 (d, $J_{C\text{-}F}$ = 247.6 Hz), 143.54, 134.97, 133.31 (d, $J_{C\text{-}F}$ = 3.2 Hz), 129.76, 128.49 (d, $J_{C\text{-}F}$ = 8.3 Hz), 127.22, 126.30 (q, $J_{C\text{-}F}$ = 279.77 Hz), 115.95 (d, $J_{C\text{-}F}$ = 21.6 Hz), 83.33, 66.23 (q, $J_{C\text{-}F}$ = 34.4 Hz), 56.40, 37.32, 21.48.

^{19}F NMR (377 MHz, $CDCl_3$) δ -74.20, -112.76.

HRMS (ESI) calculated for $C_{18}H_{20}F_4NO_3S^+$, $[M+H]^+$: 406.10945; found: 406.10967.

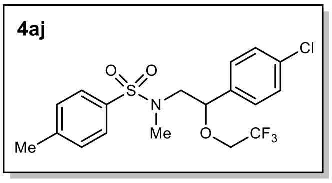

N-(2-(4-Chlorophenyl)-2-(2,2,2-trifluoroethoxy)ethyl)-N,4-dimethylbenzene-sulfonamide 4aj

The reaction was carried out following the procedure A using *N*-methyl-*p*-toluenesul - fonamide, 4-chlorostyrene and 2,2,2-trifluoroethanol. Then 94.7 mg colorless oily liquid was obtained in 75% isolated yield following purification procedure A.

^{1}H NMR (400 MHz, $CDCl_3$) δ 7.63 (d, J = 8.0 Hz, 2H), 7.36 (d, J = 8.0 Hz, 2H), 7.31–7.26 (m, 4H), 4.70 (dd, J = 8.0, 4.0 Hz, 1H), 3.74–3.63 (m, 2H), 3.34 (dd, J = 16.0, 4.0 Hz, 1H), 3.08 (dd, J = 16.0, 8.0 Hz, 1H), 2.82 (s, 3H), 2.41 (s, 3H).

^{13}C NMR (101 MHz, $CDCl_3$) δ 143.57, 136.05, 134.91, 134.71, 129.77, 129.19, 128.09, 127.22, 126.19 (q, $J_{C\text{-}F}$ = 279.77 Hz), 83.39, 66.37 (q, $J_{C\text{-}F}$ = 34.5 Hz), 56.33, 37.38, 21.50.

^{19}F NMR (377 MHz, $CDCl_3$) δ -74.21.

HRMS (ESI) calculated for $C_{18}H_{20}ClF_3NO_3S^+$, $[M+H]^+$: 422.07990; found: 422.07957.

4ak

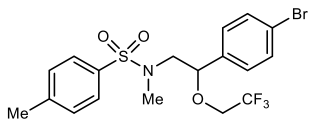

N-(2-(4-Bromophenyl)-2-(2,2,2-trifluoroethoxy)ethyl)-N,4 dimethylbenzene-sulfonamide 4ak

The reaction was carried out following the procedure A using *N*-methyl-*p*-toluenesul - fonamide, 4-bromostyrene and 2,2,2-trifluoroethanol. Then 113.0 mg colorless oily liquid was obtained in 81% isolated yield following purification procedure A.

^{1}H NMR (400 MHz, $CDCl_3$) δ 7.63 (d, J = 8.0 Hz, 2H), 7.51 (d, J = 8.0 Hz, 2H), 7.29 (d, J = 8.0 Hz, 2H), 7.21 (d, J = 8.0 Hz, 2H), 4.69 (dd, J = 8.0, 4.0 Hz, 1H), 3.73–3.64 (m, 2H), 3.34 (dd, J = 16.0, 4.0 Hz, 1H), 3.08 (dd, J = 16.0, 8.0 Hz, 1H), 2.82 (s, 3H), 2.41 (s, 3H).

^{13}C NMR (101 MHz, CDCl3) δ 143.58, 136.58, 134.90, 132.14, 129.78, 128.40, 127.23, 126.51 (q, $J_{C\text{-}F}$ = 278.77 Hz), 122.86, 83.45, 66.40 (q, $J_{C\text{-}F}$ = 34.6 Hz), 56.27, 37.38, 21.51.

^{19}F NMR (377 MHz, $CDCl_3$) δ -74.19.

HRMS (ESI) calculated for $C_{18}H_{20}BrF_3NO_3S^+$, $[M+H]^+$: 466.02939; found: 466.02917.

4al

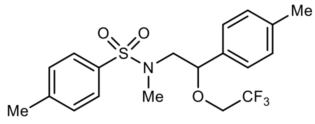

N,4-Dimethyl-N-(2-(p-tolyl)-2-(2,2,2-trifluoroethoxy)ethyl)benzenesulfonamide 4al

The reaction was carried out following the procedure A using *N*-methyl-*p*-toluenesul - fonamide, 4-methylstyrene and 2,2,2-trifluoroethanol. Then 92.6 mg colorless oily liquid was obtained in 77% isolated yield following purification procedure A.

^{1}H NMR (400 MHz, $CDCl_3$) δ 7.65 (d, J = 8.0 Hz, 2H), 7.29 (d, J = 8.0 Hz, 2H), 7.21 (s, 4H), 4.68 (dd, J = 8.0, 4.0 Hz, 1H), 3.76–3.57 (m, 2H), 3.39 (dd, J = 16.0, 4.0 Hz, 1H), 3.10 (dd, J = 16.0, 8.0 Hz, 1H), 2.85 (s, 3H), 2.41 (s, 3H), 2.36 (s, 3H).

^{13}C NMR (101 MHz, $CDCl_3$) δ 143.40, 138.76, 135.14, 134.43, 129.71, 129.63, 127.26, 126.72, 125.92 (q, $J_{C\text{-}F}$ = 279.77 Hz)， 83.84, 66.05 (q, $J_{C\text{-}F}$ = 34.4 Hz), 56.41, 37.28, 21.50, 21.21.

^{19}F NMR (377 MHz, $CDCl_3$) δ -74.17.

HRMS (ESI) calculated for $C_{19}H_{23}F_3NO_3S^+$, $[M+H]^+$: 402.13453; found: 402.13421.

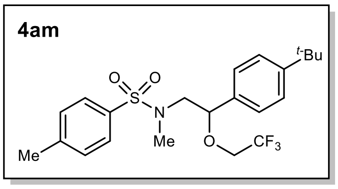

N-(2-(4-(*tert*-Butyl)phenyl)-2-(2,2,2-trifluoroethoxy)ethyl)-N,4-dimethylbenzene-sulfonamide 4am

The reaction was carried out following the procedure A using *N*-methyl-*p*-toluenesul - fonamide, 4-tert-butylstyrene and 2,2,2-trifluoroethanol. Then 93.0 mg white solid was obtained in 70% isolated yield following purification procedure A.

^{1}H NMR (400 MHz, $CDCl_3$) δ 7.66 (d, J = 8.0 Hz, 2H), 7.40 (d, J = 8.0 Hz, 2H), 7.29 (d, J = 8.0 Hz, 2H), 7.24 (d, J = 8.0 Hz, 2H), 4.69 (dd, J = 8.0, 4.0 Hz, 1H), 3.80–3.56 (m, 2H), 3.42 (dd, J = 16.0, 4.0 Hz, 1H), 3.07 (dd, J = 16.0, 8.0 Hz, 1H), 2.87 (s, 3H), 2.41 (s, 3H), 1.32 (s, 9H).

^{13}C NMR (101 MHz, $CDCl_3$) δ 151.91, 143.38, 135.19, 134.37, 129.71, 127.26, 126.42, 125.84, 123.84 (q, $J_{C\text{-}F}$ = 278.76 Hz), 83.90, 66.12 (q, $J_{C\text{-}F}$ = 34.3 Hz), 56.42, 37.27, 34.66, 31.32, 21.49.

^{19}F NMR (377 MHz, $CDCl_3$) δ -74.13.

HRMS (ESI) calculated for $C_{22}H_{29}F_3NO_3S^+$, $[M+H]^+$: 444.18148; found: 444.18119.

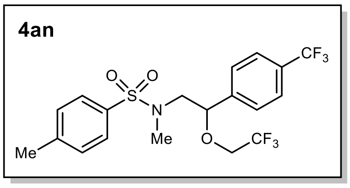

N,4-Dimethyl-N-(2-(2,2,2-trifluoroethoxy)-2-(4-(trifluoromethyl)phenyl)ethyl)-benzenesulfonamide 4an

The reaction was carried out following the procedure A using *N*-methyl-*p*-toluenesul - fonamide, 4-(trifluoromethyl)styrene and 2,2,2-trifluoroethanol. Then 61.4 mg colorless oily liquid was obtained in 45% isolated yield following purification procedure A.

^{1}H NMR (400 MHz, $CDCl_3$) δ 7.67 (s, 1H), 7.65 (s, 2H), 7.62 (s, 1H), 7.47 (d, J = 8.0 Hz, 2H), 7.30 (d, J = 8.0 Hz, 2H), 4.81 (dd, J = 8.0, 4.0 Hz, 1H), 3.78–3.68 (m, 2H), 3.37 (dd, J = 16.0, 4.0 Hz, 1H), 3.09 (dd, J = 16.0, 8.0 Hz, 1H), 2.84 (s, 3H), 2.41 (s, 3H).

^{13}C NMR (101 MHz, $CDCl_3$) δ 143.66, 141.66, 134.80, 129.88, 129.80, 127.23, 127.03, 126.75 (q, $J_{C\text{-}F}$ = 237.71 Hz), 126.32 (q, $J_{C\text{-}F}$ = 279.77 Hz), 125.95 (q, J = 3.7 Hz), 83.66, 66.70 (q, J = 34.6 Hz), 56.39, 37.47, 21.50.

^{19}F NMR (377 MHz, $CDCl_3$) δ -62.69, -74.25.

HRMS (ESI) calculated for $C_{19}H_{20}F_6NO_3S^+$, $[M+H]^+$: 456.10626; found: 456.10687.

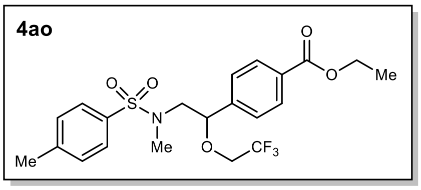

Ethyl 4-(2-((*N*,4-Dimethylphenyl)sulfonamido)-1-(2,2,2-trifluoroethoxy)ethyl)-benzoate 4ao

The reaction was carried out following the procedure A using *N*-methyl-*p*-toluenesul - fonamide, ethyl-4-vinylbenzoate and 2,2,2-trifluoroethanol. Then 50.9 mg light yellow oily liquid was obtained in 37% isolated yield following purification procedure A.

^{1}H NMR (400 MHz, $CDCl_3$) δ 8.06 (d, J = 8.0 Hz, 2H), 7.63 (d, J = 8.0 Hz, 2H), 7.41 (d, J = 8.0 Hz, 2H), 7.29 (d, J = 8.0 Hz, 2H), 4.78 (dd, J = 8.0, 4.0 Hz, 1H), 4.38 (q, J = 8.0 Hz, 2H), 3.75–3.66 (m, 2H), 3.36 (dd, J = 16.0, 4.0 Hz, 1H), 3.10 (dd, J = 16.0, 8.0 Hz, 1H), 2.82 (s, 3H), 2.41 (s, 3H), 1.39 (t, J = 7.1 Hz, 3H).

^{13}C NMR (101 MHz, $CDCl_3$) δ 166.09, 143.58, 142.45, 134.90, 131.04, 130.18, 129.78, 127.23, 126.64, 126.25 (q, $J_{C\text{-}F}$ = 278.76 Hz), 83.75, 66.58 (q, $J_{C\text{-}F}$ = 34.5 Hz), 61.18, 56.32, 37.43, 21.50, 14.33.

^{19}F NMR (377 MHz, $CDCl_3$) δ -74.24.

HRMS (ESI) calculated for $C_{21}H_{25}F_3NO_5S^+$, $[M+H]^+$: 460.14000; found: 460.13967.

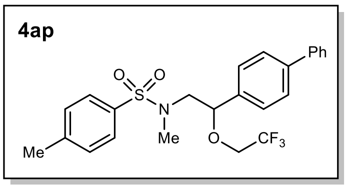

***N*-(2-([1,1'-Biphenyl]-4-yl)-2-(2,2,2-trifluoroethoxy)ethyl)-*N*,4-dimethylbenzene-sulfonamide 4ap**

The reaction was carried out following the procedure A using *N*-methyl-*p*-toluenesul - fonamide, 4-vinylbiphenyl and 2,2,2-trifluoroethanol. Then 41.7 mg light yellow oily liquid was obtained in 30% isolated yield following purification procedure A.

^{1}H NMR (400 MHz, $CDCl_3$) δ 7.67 (d, J = 8.0 Hz, 2H), 7.64–7.57 (m, 4H), 7.48–7.43 (m, 2H), 7.42–7.35 (m, 3H), 7.30 (d, J = 8.0 Hz, 2H), 4.77 (dd, J = 8.0, 4.0 Hz, 1H), 3.87–3.59 (m, 2H), 3.45 (dd, J = 16.0, 4.0 Hz, 1H), 3.13 (dd, J = 16.0, 8.0 Hz, 1H), 2.88 (s, 3H), 2.41 (s, 3H).

^{13}C NMR (101 MHz, $CDCl_3$) δ 143.45, 141.82, 140.38, 136.42, 135.11, 129.74, 128.89, 127.67, 127.63, 127.27, 127.18, 127.11, 126.52 (q, $J_{C\text{-}F}$ = 279.77 Hz), 83.89, 66.29 (q, $J_{C\text{-}F}$ = 34.4 Hz), 56.45, 37.38, 21.52.

^{19}F NMR (377 MHz, $CDCl_3$) δ -74.14.

HRMS (ESI) calculated for $C_{24}H_{25}F_3NO_3S^+$, $[M+H]^+$: 464.15018; found: 464.14963.

4aq

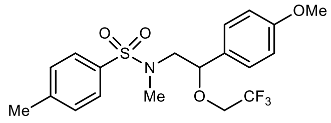

N-(2-(4-Methoxyphenyl)-2-(2,2,2-trifluoroethoxy)ethyl)-N,4-dimethylbenzene-sulfonamide 4aq

The reaction was carried out following the procedure A using *N*-methyl-*p*-toluenesul - fonamide, 4-methoxystyrene and 2,2,2-trifluoroethanol. Then 13.8 mg light yellow oily liquid was obtained in 11% isolated yield following purification procedure A.

^{1}H NMR (400 MHz, $CDCl_3$) δ 7.62 (d, J = 8.1 Hz, 2H), 7.27 (d, J = 8.0 Hz, 2H), 7.21 (d, J = 8.0 Hz, 2H), 6.89 (d, J = 8.0 Hz, 2H), 4.63 (dd, J = 8.0, 4.0 Hz, 1H), 3.79 (s, 3H), 3.69–3.57 (m, 2H), 3.33 (dd, J = 16.0, 4.0 Hz, 1H), 3.08 (dd, J = 16.0, 8.0 Hz, 1H), 2.80 (s, 3H), 2.39 (s, 3H).

^{13}C NMR (101 MHz, $CDCl_3$) δ 160.00, 143.38, 135.14, 129.70, 129.37, 128.08, 127.25, 126.57 (q, $J_{C\text{-}F}$ = 279.77 Hz), 114.31, 83.49, 65.91 (q, $J_{C\text{-}F}$ = 34.1 Hz), 56.35, 55.33, 37.26, 21.50.

^{19}F NMR (377 MHz, $CDCl_3$) δ -74.15.

HRMS (ESI) calculated for $C_{19}H_{23}F_3NO_4S^+$, $[M+H]^+$: 418.12944; found: 418.12913.

4ar

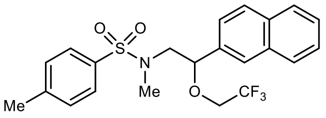

N,4-Dimethyl-N-(2-(naphthalen-2-yl)-2-(2,2,2-trifluoroethoxy)ethyl)benzene-sulfonamide 4ar

The reaction was carried out following the procedure A using *N*-methyl-*p*-toluenesul - fonamide, 2-vinylnaphthalene and 2,2,2-trifluoroethanol. Then 24.9 mg light yellow oily liquid was obtained in 19% isolated yield following purification procedure A.

^{1}H NMR (400 MHz, $CDCl_3$) δ 7.91–7.83 (m, 3H), 7.79 (s, 1H), 7.65 (d, J = 8.0 Hz, 2H), 7.55–7.51 (m, 2H), 7.44 (dd, J = 8.0, 4.0 Hz, 1H), 7.38–7.31 (m, 1H), 7.28 (s, 1H), 4.89 (dd, J = 8.0, 4.0 Hz, 1H), 3.80–3.67 (m, 2H), 3.49 (dd, J = 16.0, 4.0 Hz, 1H), 3.21 (dd, J = 16.0, 8.0 Hz, 1H), 2.87 (s, 3H), 2.40 (s, 3H).

^{13}C NMR (101 MHz, $CDCl_3$) δ 143.44, 135.08, 134.84, 133.52, 133.16, 129.72, 129.04, 128.03, 127.81, 127.25, 126.65, 126.60, 126.46, 123.77, 126.51 (q, $J_{C\text{-}F}$ = 277.75 Hz), 84.21, 66.26 (q, $J_{C\text{-}F}$ = 34.34 Hz), 56.34, 37.37, 21.50.

^{19}F NMR (377 MHz, $CDCl_3$) δ -74.12.

HRMS (ESI) calculated for $C_{22}H_{23}F_3NO_3S^+$, $[M+H]^+$: 438.13453; found: 438.13405.

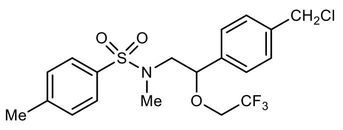

N-(2-(4-(Chloromethyl)phenyl)-2-(2,2,2-trifluoroethoxy)ethyl)-N,4-dimethyl-benzenesulfonamide 4as

The reaction was carried out following the procedure A using *N*-methyl-*p*-toluenesul - fonamide, 4-(chloromethyl)styrene and 2,2,2-trifluoroethanol. Then 101.8 mg colorless oily liquid was obtained in 78% isolated yield following purification procedure A.

^{1}H NMR (400 MHz, $CDCl_3$) δ 7.64 (d, *J* = 8.0 Hz, 2H), 7.42 (d, *J* = 8.0 Hz, 2H), 7.33 (d, *J* = 8.0 Hz, 2H), 7.29 (d, *J* = 8.0 Hz, 2H), 4.73 (dd, *J* = 8.0, 4.0 Hz, 1H), 4.59 (s, 2H), 3.76–3.62 (m, 2H), 3.38 (dd, *J* = 16.0, 4.0 Hz, 1H), 3.07 (dd, *J* = 16.0, 8.0 Hz, 1H), 2.84 (s, 3H), 2.41 (s, 3H).

^{13}C NMR (101 MHz, $CDCl_3$) δ 143.52, 138.17, 137.81, 134.98, 129.76, 129.20, 127.24, 127.11, 126.29 (q, $J_{C\text{-}F}$ = 279.77 Hz), 83.77, 66.36 (q, $J_{C\text{-}F}$ = 34.4 Hz), 56.40, 45.71, 37.36, 21.50.

^{19}F NMR (377 MHz, $CDCl_3$) δ -74.19.

HRMS (ESI) calculated for $C_{19}H_{22}ClF_3NO_3S^+$, $[M+H]^+$: 436.09555; found: 436.09500.

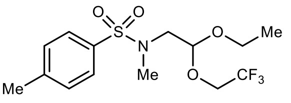

N-(2-Ethoxy-2-(2,2,2-trifluoroethoxy)ethyl)-N,4-dimethylbenzenesulfonamide 4at

The reaction was carried out following the procedure A using *N*-methyl-*p*-toluenesul - fonamide, ethyl vinyl ether and 2,2,2-trifluoroethanol. Then 70.3 mg colorless oily liquid was obtained in 66% isolated yield following purification procedure A.

^{1}H NMR (400 MHz, $CDCl_3$) δ 7.66 (d, *J* = 8.0 Hz, 2H), 7.32 (d, *J* = 8.0 Hz, 2H), 4.78 (t, *J* = 8.0 Hz, 1H), 4.05–3.87 (m, 2H), 3.76 (dq, *J* = 9.4, 7.1 Hz, 1H), 3.61–3.54 (m, 1H), 3.16 (dd, *J* = 16.0, 4.0 Hz, 1H), 3.08 (dd, *J* = 16.0, 4.0 Hz, 1H), 2.83 (s, 3H), 2.42 (s, 3H), 1.21 (t, *J* = 8.0 Hz, 3H).

^{13}C NMR (101 MHz, $CDCl_3$) δ 143.64, 134.55, 129.79, 127.30, 126.53 (q, $J_{C\text{-}F}$ = 278.76 Hz), 102.53, 63.77, 63.44 (q, $J_{C\text{-}F}$ = 34.4 Hz), 52.20, 36.82, 21.49, 15.07.

^{19}F NMR (377 MHz, $CDCl_3$) δ -74.28.

HRMS (ESI) calculated for $C_{14}H_{20}F_3NNaO_4S^+$, $[M+Na]^+$: 378.09573; found: 378.09537.

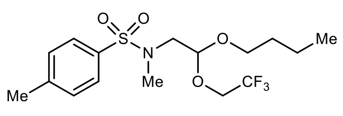

N-(2-Butoxy-2-(2,2,2-trifluoroethoxy)ethyl)-N,4-dimethylbenzenesulfonamide 4au

The reaction was carried out following the procedure A using *N*-methyl-*p*-toluenesul - fonamide, butyl vinyl ether and 2,2,2-trifluoroethanol. Then 59.7 mg light yellow oily liquid was obtained in 52% isolated yield following purification procedure A.

^{1}H NMR (400 MHz, $CDCl_3$) δ 7.66 (d, J = 8.0 Hz, 2H), 7.32 (d, J = 8.0 Hz, 2H), 4.77 (t, J = 4.0 Hz, 1H), 4.06–3.84 (m, 2H), 3.69 (dd, J = 16.0, 4.0 Hz, 1H), 3.50 (dd, J = 16.0, 8.0 Hz, 1H), 3.16 (dd, J = 12.0, 4.0 Hz, 1H), 3.07 (dd, J = 16.0, 4.0 Hz, 1H), 2.83 (s, 3H), 2.42 (s, 3H), 1.59–1.52 (m, 2H), 1.42–1.30 (m, 2H), 0.92 (t, J = 8.0 Hz, 3H).

^{13}C NMR (101 MHz, $CDCl_3$) δ 143.62, 134.59, 129.79, 127.30, 126.53 (q, $J_{C\text{-}F}$ = 278.76 Hz), 102.69, 68.03, 63.41 (q, $J_{C\text{-}F}$ = 34.8 Hz), 52.14, 36.82, 31.66, 21.50, 19.21, 13.80.

^{19}F NMR (377 MHz, $CDCl_3$) δ -74.26.

HRMS (ESI) calculated for $C_{16}H_{24}F_3NNaO_4S^+$, $[M+Na]^+$: 406.12703; found: 406.12645.

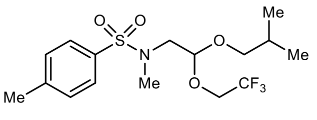

N-(2-Isobutoxy-2-(2,2,2-trifluoroethoxy)ethyl)-N,4-dimethylbenzenesulfonamide 4av

The reaction was carried out following the procedure A using N-methyl-*p*-toluenesul - fonamide, isobutyl vinyl ether and 2,2,2-trifluoroethanol. Then 57.5 mg light yellow oily liquid was obtained in 50% isolated yield following purification procedure A.

^{1}H NMR (400 MHz, $CDCl_3$) δ 7.66 (d, J = 8.0 Hz, 2H), 7.32 (d, J = 8.0 Hz, 2H), 4.77 (t, J = 4.0 Hz, 1H), 4.05–3.84 (m, 2H), 3.46 (dd, J = 8.0, 4.0 Hz, 1H), 3.27 (dd, J = 8.0, 4.0 Hz, 1H), 3.17 (dd, J = 16.0, 4.0 Hz, 1H), 3.07 (dd, J = 16.0, 4.0 Hz, 1H), 2.83 (s, 3H), 2.42 (s, 3H), 1.89–1.79 (m, 1H), 0.92 (d, J = 4.0 Hz, 3H), 0.90 (d, J = 4.0 Hz, 3H).

^{13}C NMR (101 MHz, $CDCl_3$) δ 143.64, 134.54, 129.81, 127.29, 126.53 (q, $J_{C\text{-}F}$ = 278.76 Hz), 102.74, 74.78, 63.34 (q, $J_{C\text{-}F}$ = 34.9 Hz), 52.07, 36.83, 28.54, 21.50, 19.23.

^{19}F NMR (377 MHz, $CDCl_3$) δ -74.25.

HRMS (ESI) calculated for $C_{16}H_{24}F_3NNaO_4S^+$, $[M+Na]^+$: 406.12703; found: 406.12634.

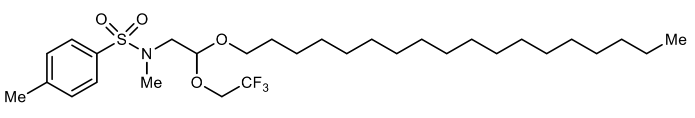

N,4-Dimethyl-N-(2-(octadecyloxy)-2-(2,2,2-trifluoroethoxy)ethyl)benzenesulfonamide 4aw

The reaction was carried out following the procedure A using *N*-methyl-*p*-toluenesul - fonamide, 1-(vinyloxy)octadecane and 2,2,2-trifluoroethanol. Then 43.4 mg white solid was obtained in 25% isolated yield following purification procedure A.

^{1}H NMR (400 MHz, $CDCl_3$) δ 7.67 (d, J = 8.0 Hz, 2H), 7.32 (d, J = 8.0 Hz, 2H), 4.77 (t, J = 4.0 Hz, 1H), 4.03–3.85 (m, 2H), 3.71–3.66 (m, 1H), 3.52–3.46 (m, 1H), 3.16 (dd, J = 16.0, 4.0 Hz, 1H), 3.08 (dd, J = 16.0, 4.0 Hz, 1H), 2.84 (s, 3H), 2.43 (s, 3H), 1.25 (s, 32H), 0.87 (t, J = 6.8 Hz, 3H).

^{13}C NMR (101 MHz, $CDCl_3$) δ 143.60, 134.62, 129.79, 127.32, 126.52 (q, $J_{C\text{-}F}$ = 278.76 Hz), 102.75, 68.38, 63.38 (q, $J_{C\text{-}F}$ = 35.35 Hz), 52.16, 36.84, 32.83, 31.94, 29.71, 29.67, 29.63, 29.62, 29.58, 29.45, 29.39, 29.38, 26.03, 25.75, 22.71, 21.52, 14.13.

^{19}F NMR (377 MHz, $CDCl_3$) δ -74.25.

HRMS (ESI) calculated for $C_{30}H_{52}F_3N_aO_4S^+$, $[M+Na]^+$: 602.34614; found: 602.34593.

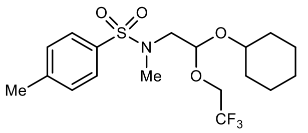

N-(2-(Cyclohexyloxy)-2-(2,2,2-trifluoroethoxy)ethyl)-N,4-dimethylbenzenesulfonamide 4ax

The reaction was carried out following the procedure A using *N*-methyl-*p*-toluenesul - fonamide, 1-(vinyloxy)octadecane and 2,2,2-trifluoroethanol. Then 69.9 mg colorless oily liquid was obtained in 57% isolated yield following purification procedure A.

^{1}H NMR (400 MHz, $CDCl_3$) δ 7.66 (d, J = 8.0 Hz, 2H), 7.32 (d, J = 8.0 Hz, 2H), 4.90 (t, J = 4.0 Hz, 1H), 4.03–3.85 (m, 2H), 3.60–3.51 (m, 1H), 3.17 (dd, J = 16.0, 4.0 Hz, 1H), 3.01 (dd, J = 16.0, 4.0 Hz, 1H), 2.84 (s, 3H), 2.42 (s, 3H), 1.92–1.85 (m, 2H), 1.76–1.69 (m, 2H), 1.55–1.51 (m, 1H), 1.41–1.16 (m, 5H).

^{13}C NMR (101 MHz, $CDCl_3$) δ 143.58, 134.74, 129.79, 127.27, 126.60 (q, $J_{C\text{-}F}$ = 278.76 Hz), 100.91, 62.81 (q, $J_{C\text{-}F}$ = 34.8 Hz), 52.82, 36.95, 32.99, 32.13, 25.45, 24.08, 23.87, 21.50.

^{19}F NMR (377 MHz, $CDCl_3$) δ -74.16.

HRMS (ESI) calculated for $C_{18}H_{26}F_3NN_aO_4S^+$, $[M+Na]^+$: 432.14268; found: 432.14230.

4ay

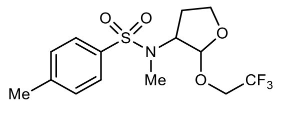

N,4-Dimethyl-N-(2-(2,2,2-trifluoroethoxy)tetrahydrofuran-3-yl)benzenesulfon-amide 4ay

The reaction was carried out following the procedure A using *N*-methyl-*p*-toluenesul - fonamide, 2,3-dihydrofuran and 2,2,2-trifluoroethanol. Then 63.5 mg light yellow oily liquid was obtained in 60% isolated yield following purification procedure A.

^{1}H NMR (400 MHz, $CDCl_3$) δ 7.69 (d, J = 8.0 Hz, 2H), 7.31 (d, J = 8.0 Hz, 2H), 4.69–4.63 (m, 2H), 4.03–3.98 (m, 1H), 3.86–3.74 (m, 2H), 3.67–3.57 (m, 1H), 2.68 (s, 3H), 2.42 (s, 3H), 2.29–2.21 (m, 1H), 1.73–1.63 (m, 1H).

^{13}C NMR (101 MHz, $CDCl_3$) δ 143.71, 135.24, 129.82, 127.33, 126.06 (q, $J_{C\text{-}F}$ = 278.76 Hz), 104.76, 66.84, 66.35 (q, $J_{C\text{-}F}$ = 35.35 Hz), 62.82, 29.83, 27.19, 21.51.

^{19}F NMR (377 MHz, $CDCl_3$) δ -74.06.

HRMS (ESI) calculated for $C_{14}H_{19}F_3NO_4S^+$, $[M+H]^+$: 354.09814; found: 354.09771.

4az

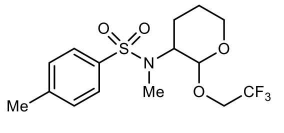

N,4-Dimethyl-N-(2-(2,2,2-trifluoroethoxy)tetrahydro-2H-pyran-3-yl)benzene-sulfonamide 4az

The reaction was carried out following the procedure A using *N*-methyl-*p*-toluenesul - fonamide, dihydropyran and 2,2,2-trifluoroethanol. Then 56.2 mg light yellow oily liquid was obtained in 51% isolated yield following purification procedure A.

^{1}H NMR (400 MHz, $CDCl_3$) δ 7.69 (d, J = 8.0 Hz, 2H), 7.25 (d, J = 8.0 Hz, 2H), 4.38 (d, J = 8.0 Hz, 1H), 3.96–3.72 (m, 4H), 3.44–3.36 (m, 1H), 2.80 (s, 3H), 2.39 (s, 3H), 2.00–1.93 (m, 1H), 1.78–1.67 (m, 3H).

^{13}C NMR (101 MHz, $CDCl_3$) δ 143.07, 136.61, 129.35, 127.36, 123.57 (q, $J_{C\text{-}F}$ = 279.8 Hz), 101.00, 65.36, 64.14 (q, $J_{C\text{-}F}$ = 34.8 Hz), 56.77, 29.79, 27.05, 24.77, 21.46.

^{19}F NMR (377 MHz, $CDCl_3$) δ -74.00.

HRMS (ESI) calculated for $C_{15}H_{21}F_3NO_4S^+$, $[M+H]^+$: 368.11379; found: 368.11367.

4ba

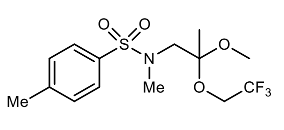

N-(2-Methoxy-2-(2,2,2-trifluoroethoxy)propyl)-N,4-dimethylbenzenesulfonamide 4ba

The reaction was carried out following the procedure: graphite rod anode (Φ 6 mm), Pt plate cathode (15 mm x 15 mm x 0.3 mm), constant current = 5 mA, N-methyl-*p*-toluenesul -fonamide (0.3 mmol, 1 equiv.), 2-methoxypropene (1.8 mmol, 6 equiv.), DBU (0.39 mmol, 1.3 equiv.), TBABF4 (0.3 mmol), DCM/TFE (4/2 mL), r.t., 6 h, undivided cell under N2. Then 86.3 mg colorless oily liquid was obtained in 83% isolated yield following purification procedure A.

^{1}H NMR (400 MHz, $CDCl_3$) δ 7.65 (d, J = 8.0 Hz, 2H), 7.32 (d, J = 8.0 Hz, 2H), 3.88–3.74 (m, 2H), 3.24 (s, 3H), 3.14 (s, 2H), 2.80 (s, 3H), 2.43 (s, 3H), 1.50 (s, 3H).

^{13}C NMR (101 MHz, $CDCl_3$) δ 143.60, 134.39, 129.81, 127.38, 126.83 (q, $J_{C\text{-}F}$ = 278.76 Hz), 102.79, 59.21 (q, $J_{C\text{-}F}$ = 34.3 Hz), 54.30, 48.99, 36.46, 21.53, 20.44.

^{19}F NMR (377 MHz, $CDCl_3$) δ -73.93.

HRMS (ESI) calculated for $C_{14}H_{20}F_3NNaO_4S^+$, $[M+Na]^+$: 378.09573; found: 378.09484.

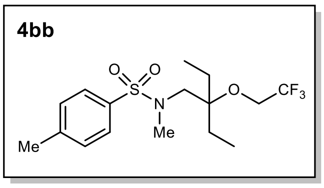

N-(2-ethyl-2-(2,2,2-trifluoroethoxy)butyl)-N,4-dimethylbenzenesulfonamide 4bb

The reaction was carried out following the procedure: graphite rod anode (Φ 6 mm), Pt plate cathode (15 mm x 15 mm x 0.3 mm), constant current = 4 mA, N-methyl-*p*-toluenesul -fonamide (0.3 mmol, 1 equiv.), 2-methoxypropene (3.0 mmol, 10 equiv.), DBU (0.6 mmol, 2.0 equiv.), TBABF4 (0.3 mmol), DCM/TFE (4/2 mL), r.t., 5 h, undivided cell under N2. Then 17.6 mg light yellow oily liquid was obtained in 16% isolated yield following purification procedure A.

^{1}H NMR (400 MHz, $CDCl_3$) δ 7.65 (d, J = 8.0 Hz, 2H), 7.32 (d, J = 8.0 Hz, 2H), 3.68 (q, J = 8.0 Hz, 2H), 3.05 (s, 2H), 2.80 (s, 3H), 2.43 (s, 3H), 1.77 – 1.57 (m, 4H), 0.93 (t, J = 7.5 Hz, 6H).

^{13}C NMR (101 MHz, $CDCl_3$) δ 143.46, 133.98, 129.73, 127.53, 126.90 (q, $J_{C\text{-}F}$ = 287.76 Hz), 81.99, 59.80 (q, JC-F = 34.3 Hz), 53.36, 37.04, 25.87, 21.53, 7.48.

^{19}F NMR (377 MHz, $CDCl_3$) δ -74.13.

HRMS (ESI) calculated for $C_{16}H_{25}F_3NO_3S^+$, $[M+H]^+$: 368.15018; found: 368.14984.

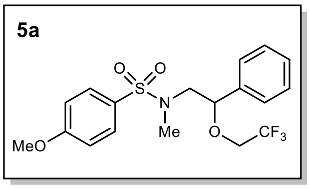

4-Methoxy-N-methyl-N-(2-phenyl-2-(2,2,2-trifluoroethoxy)ethyl)benzenesulfon-amide 5a

The reaction was carried out following the procedure A using 4-methoxy-*N*-methylbenzenesulfonamide, styrene and 2,2,2-trifluoroethanol. Then 70.1 mg colorless oily liquid was obtained in 58% isolated yield following purification procedure A.
^{1}H NMR (400 MHz, $CDCl_3$) δ 7.70 (d, *J* = 8.0 Hz, 2H), 7.41–7.35 (m, 3H), 7.33–7.31 (m, 2H), 6.96 (d, *J* = 8.0 Hz, 2H), 4.71 (dd, *J* = 8.0, 3.9 Hz, 1H), 3.85 (s, 3H), 3.76–3.63 (m, 2H), 3.38 (dd, *J* = 16.0, 4.0 Hz, 1H), 3.10 (dd, *J* = 16.0, 8.0 Hz, 1H), 2.83 (s, 3H).
^{13}C NMR (101 MHz, $CDCl_3$) δ 162.87, 137.52, 129.74, 129.33, 128.96, 128.86, 126.72, 126.39 (q, $J_{C\text{-}F}$ = 279.77 Hz), 114.26, 84.02, 66.23 (q, $J_{C\text{-}F}$ = 34.4 Hz), 56.42, 55.61, 37.27.
^{19}F NMR (377 MHz, $CDCl_3$) δ -74.17.
HRMS (ESI) calculated for $C_{18}H_{21}F_3NO_4S^+$, $[M+H]^+$: 404.11379; found: 404.11395.

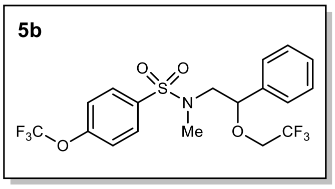

_N_-Methyl-_N_-(2-phenyl-2-(2,2,2-trifluoroethoxy)ethyl)-4-(trifluoromethoxy)-benzenesulfonamide 5b

The reaction was carried out following the procedure A using *N*-methyl-4-(trifluoromethoxy)benzenesulfonamide, styrene and 2,2,2-trifluoroethanol. Then 91.9 mg colorless oily liquid was obtained in 67% isolated yield following purification procedure A.
^{1}H NMR (400 MHz, $CDCl_3$) δ 7.82 (d, *J* = 8.0 Hz, 2H), 7.43–7.40 (m, 1H), 7.39–7.36 (m, 2H), 7.35–7.30 (m, 4H), 4.71 (dd, *J* = 8.6, 3.8 Hz, 1H), 3.77–3.57 (m, 2H), 3.42 (dd, *J* = 16.0, 4.0 Hz, 1H), 3.19 (dd, *J* = 16.0, 8.0 Hz, 1H), 2.90 (s, 3H).
^{13}C NMR (101 MHz, $CDCl_3$) δ 152.10 (s), 137.16, 136.74, 129.29, 129.05, 129.03, 126.72, 126.49 (q, $J_{C\text{-}F}$ = 278.76 Hz), 122.80 (q, $J_{C\text{-}F}$ = 260.58 Hz), 121.05, 83.78, 66.10 (q, $J_{C\text{-}F}$ = 34.4 Hz), 56.31, 37.08.
^{19}F NMR (377 MHz, $CDCl_3$) δ -57.75, -74.17.
HRMS (ESI) calculated for $C_{18}H_{18}F_6NO_4S^+$, $[M+H]^+$: 458.08552; found: 458.08532.

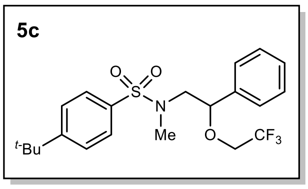

4-(_tert_-Butyl)-N-methyl-N-(2-phenyl-2-(2,2,2-trifluoroethoxy)ethyl)benzene-sulfonamide 5c

The reaction was carried out following the procedure A using 4-(tert-butyl)-*N*-methylbenzenesulfonamide, styrene and 2,2,2-trifluoroethanol. Then 88.8 mg colorless oily liquid was obtained in 69% isolated yield following purification procedure A.

^{1}H NMR (400 MHz, $CDCl_3$) δ 7.70 (d, J = 8.0 Hz, 2H), 7.50 (d, J = 8.0 Hz, 2H), 7.42–7.34 (m, 3H), 7.32 (d, J = 8.0 Hz, 2H), 4.71 (dd, J = 8.0, 4.0 Hz, 1H), 3.76–3.56 (m, 2H), 3.42 (dd, J = 16.0, 4.0 Hz, 1H), 3.13 (dd, J = 16.0, 8.0 Hz, 1H), 2.88 (s, 3H), 1.33 (s, 9H).
^{13}C NMR (101 MHz, $CDCl_3$) δ 156.36, 137.51, 135.15, 128.96, 128.86, 127.08, 126.71, 126.51 (q, $J_{C\text{-}F}$ = 279.77 Hz), 126.10, 83.96, 66.20 (q, $J_{C\text{-}F}$ = 34.5 Hz), 56.50, 37.38, 35.13, 31.07.
^{19}F NMR (377 MHz, $CDCl_3$) δ -74.17.
HRMS (ESI) calculated for $C_{21}H_{27}F_3NO_3S^+$, $[M+H]^+$: 430.16583; found: 43016602.

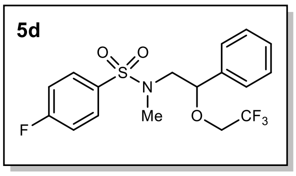

4-Fluoro-*N*-methyl-*N*-(2-phenyl-2-(2,2,2-trifluoroethoxy)ethyl)benzenesulfon-amide 5d

The reaction was carried out following the procedure A using 4-fluoro-*N*-methylbenzenesulfonamide, styrene and 2,2,2-trifluoroethanol. Then 99.7 mg colorless oily liquid was obtained in 85% isolated yield following purification procedure A.
^{1}H NMR (400 MHz, $CDCl_3$) δ 7.80–7.75 (m, 2H), 7.43–7.35 (m, 3H), 7.34–7.30 (m, 21H), 7.17 (t, J = 8.0 Hz, 2H), 4.71 (dd, J = 8.0, 4.0 Hz, 1H), 3.77–3.60 (m, 2H), 3.40 (dd, J = 16.0, 4.0 Hz, 1H), 3.16 (dd, J = 16.0, 8.0 Hz, 1H), 2.86 (s, 3H).
^{13}C NMR (101 MHz, $CDCl_3$) δ 165.08 (d, $J_{C\text{-}F}$ = 254.6 Hz), 137.28, 134.32 (d, $J_{C\text{-}F}$ = 3.2 Hz), 129.87 (d, $J_{C\text{-}F}$ = 9.3 Hz), 129.03, 128.98, 126.72, 126.52 (q, $J_{C\text{-}F}$ = 279.77 Hz), 116.35 (d, $J_{C\text{-}F}$ = 22.5 Hz), 83.90, 66.16 (q, $J_{C\text{-}F}$ = 34.4 Hz), 56.32, 37.11.
^{19}F NMR (377 MHz, $CDCl_3$) δ -74.16, -105.42.
HRMS (ESI) calculated for $C_{17}H_{18}F_4NO_3S^+$, $[M+H]^+$: 392.09380; found: 392.09364.

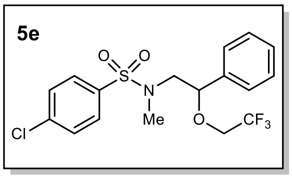

4-Chloro-*N*-methyl-*N*-(2-phenyl-2-(2,2,2-trifluoroethoxy)ethyl)benzenesulfon-amide 5e

The reaction was carried out following the procedure A using 4-chloro-*N*-methylbenzenesulfonamide, styrene and 2,2,2-trifluoroethanol. Then 89.1 mg colorless oily liquid was obtained in 73% isolated yield following purification procedure A.
^{1}H NMR (400 MHz, $CDCl_3$) δ 7.70 (d, J = 8.0 Hz, 2H), 7.47 (d, J = 8.0 Hz, 2H), 7.42–7.35 (m, 3H), 7.32 (dd, J = 8.0, 4.0 Hz, 2H), 4.71 (dd, J = 8.0, 4.0 Hz, 1H),

3.79–3.59 (m, 2H), 3.40 (dd, J = 16.0, 4.0 Hz, 1H), 3.15 (dd, J = 16.0, 8.0 Hz, 1H), 2.87 (s, 3H).
^{13}C NMR (101 MHz, $CDCl_3$) δ 139.15, 137.22, 136.71, 129.43, 129.04, 129.01, 128.63, 126.72, 126.51 (q, $J_{C\text{-}F}$ = 278.76 Hz), 83.89, 66.16 (q, $J_{C\text{-}F}$ = 34.4 Hz), 56.32, 37.12.
^{19}F NMR (377 MHz, $CDCl_3$) δ -74.14.
HRMS (ESI) calculated for $C_{17}H_{18}ClF_3NO_3S^+$, $[M+H]^+$: 408.06425; found: 408.06398.

5f

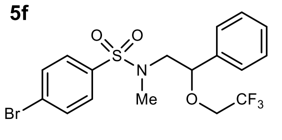

4-Bromo-*N*-methyl-*N*-(2-phenyl-2-(2,2,2-trifluoroethoxy)ethyl)benzenesulfonamide 5f

The reaction was carried out following the procedure A using 4-bromo-*N*-methylbenzenesulfonamide, styrene and 2,2,2-trifluoroethanol. Then 97.4 mg colorless oily liquid was obtained in 72% isolated yield following purification procedure A.
^{1}H NMR (400 MHz, $CDCl_3$) δ 7.63 (s, 4H), 7.42–7.36 (m, 3H), 7.34–7.30 (m, 2H), 4.71 (dd, J = 8.0, 4.0 Hz, 1H), 3.78–3.61 (m, 2H), 3.39 (dd, J = 16.0, 4.0 Hz, 1H), 3.15 (dd, J = 16.0, 8.0 Hz, 1H), 2.87 (s, 3H).
^{13}C NMR (101 MHz, $CDCl_3$) δ 137.23, 137.21, 132.41, 129.04, 129.01, 128.72, 127.62, 126.72, 126.51 (q, $J_{C\text{-}F}$ = 279.77 Hz), 83.90, 66.16 (q, $J_{C\text{-}F}$ = 34.5 Hz), 56.32, 37.13.
^{19}F NMR (377 MHz, $CDCl_3$) δ -74.13.
HRMS (ESI) calculated for $C_{17}H_{18}BrF_3NO_3S^+$, $[M+H]^+$: 452.01374; found: 452.01362.

5g

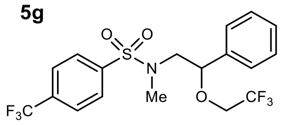

***N*-Methyl-*N*-(2-phenyl-2-(2,2,2-trifluoroethoxy)ethyl)-4-(trifluoromethyl)-benzenesulfonamide 5g**

The reaction was carried out following the procedure A using *N*-methyl-4-(trifluoromethyl)benzenesulfonamide, styrene and 2,2,2-trifluoroethanol. Then 79.4 mg colorless oily liquid was obtained in 60% isolated yield following purification procedure A.
^{1}H NMR (400 MHz, $CDCl_3$) δ 7.90 (d, J = 8.2 Hz, 2H), 7.77 (d, J = 8.0 Hz, 2H), 7.43–7.36 (m, 3H), 7.33–7.31 (m, 2H), 4.72 (dd, J = 8.0, 4.0 Hz, 1H), 3.77–3.58 (m, 2H), 3.43 (dd, J = 16.0, 4.0 Hz, 1H), 3.20 (dd, J = 16.0, 8.0 Hz, 1H), 2.91 (s, 3H).
^{13}C NMR (101 MHz, $CDCl_3$) δ 141.89, 141.88, 137.06, 134.31 (q, J = 33.1 Hz), 129.08, 127.66, 126.72, 126.48 (q, $J_{C\text{-}F}$ = 278.76 Hz), 125.94 (q, $J_{C\text{-}F}$ = 273.71 Hz), 126.28 (q, J = 3.7 Hz), 83.79, 66.10 (q, $J_{C\text{-}F}$ = 34.4 Hz), 56.30, 37.05.
^{19}F NMR (377 MHz, $CDCl_3$) δ -63.16, -74.16.

HRMS (ESI) calculated for $C_{18}H_{18}F_6NO_3S^+$, $[M+H]^+$: 422.09061; found: 422.09133.

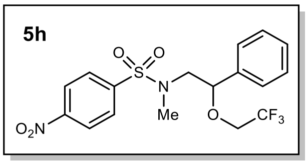

N-Methyl-4-nitro-N-(2-phenyl-2-(2,2,2-trifluoroethoxy)ethyl)benzenesulfonamide 5h

The reaction was carried out following the procedure A using *N*-methyl-4-nitrobenzenesulfonamide, styrene and 2,2,2-trifluoroethanol. Then 61.4 mg light yellow oily liquid was obtained in 49% isolated yield following purification procedure A.

^{1}H NMR (400 MHz, $CDCl_3$) δ 8.34 (d, J = 8.0 Hz, 2H), 7.95 (d, J = 8.0 Hz, 2H), 7.45–7.36 (m, 3H), 7.34–7.29 (m, 2H), 4.72 (dd, J = 8.0, 4.0 Hz, 1H), 3.78–3.57 (m, 2H), 3.44 (dd, J = 16.0, 4.0 Hz, 1H), 3.24 (dd, J = 16.0, 8.0 Hz, 1H), 2.93 (s, 3H).

^{13}C NMR (101 MHz, $CDCl_3$) δ 150.02, 144.21, 136.85, 129.19, 129.14, 128.37, 126.71, 126.47 (q, $J_{C\text{-}F}$ = 279.77 Hz), 124.40, 83.70, 66.07 (q, $J_{C\text{-}F}$ = 34.3 Hz), 56.26, 36.95.

^{19}F NMR (377 MHz, $CDCl_3$) δ -74.10.

HRMS (ESI) calculated for $C_{17}H_{18}F_3N_2O_5S^+$, $[M+H]^+$: 419.08830; found: 419.08762.

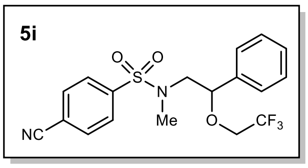

4-Cyano-N-methyl-N-(2-phenyl-2-(2,2,2-trifluoroethoxy)ethyl)benzenesulfon-amide 5i

The reaction was carried out following the procedure A using 4-cyano-*N*-methylbenzenesulfonamide, styrene and 2,2,2-trifluoroethanol. Then 66.9 mg colorless oily liquid was obtained in 56% isolated yield following purification procedure A.

^{1}H NMR (400 MHz, $CDCl_3$) δ 7.88 (d, J = 8.0 Hz, 2H), 7.79 (d, J = 8.0 Hz, 2H), 7.43–7.34 (m, 3H), 7.34–7.29 (m, 2H), 4.71 (dd, J = 8.0, 4.0 Hz, 1H), 3.76–3.57 (m, 2H), 3.41 (dd, J = 16.0, 4.0 Hz, 1H), 3.21 (dd, J = 16.0, 8.0 Hz, 1H), 2.91 (s, 3H).

^{13}C NMR (101 MHz, $CDCl_3$) δ 142.65, 136.90, 132.97, 129.16, 129.12, 127.77, 126.71, 126.48 (q, $J_{C\text{-}F}$ = 279.77 Hz), 117.32, 116.33, 83.70, 66.06 (q, $J_{C\text{-}F}$ = 34.4 Hz), 56.25, 36.97.

^{19}F NMR (377 MHz, $CDCl_3$) δ -74.10.

HRMS (ESI) calculated for $C_{18}H_{18}F_3N_2O_3S^+$, $[M+H]^+$: 399.09847; found: 399.09805.

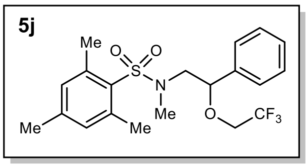

N,2,4,6-tetramethyl-N-(2-phenyl-2-(2,2,2 trifluoroethoxy)ethyl)benzenesulfon-amide 5j

The reaction was carried out following the procedure A using N,2,4,6-tetramethylbenzenesulfonamide, styrene and 2,2,2-trifluoroethanol. Then 44.8 mg colorless oily liquid was obtained in 36% isolated yield following purification procedure A.

^{1}H NMR (400 MHz, $CDCl_3$) δ 7.38–7.30 (m, 3H), 7.23 (dd, J = 8.0, 4.0 Hz, 2H), 6.94 (s, 2H), 4.62 (dd, J = 8.0, 4.0 Hz, 1H), 3.74–3.58 (m, 2H), 3.50 (dd, J = 16.0, 4.0 Hz, 1H), 3.24 (dd, J = 16.0, 8.0 Hz, 1H), 2.87 (s, 3H), 2.57 (s, 6H), 2.30 (s, 3H).

^{13}C NMR (101 MHz, $CDCl_3$) δ 142.56, 140.37, 137.58, 131.94, 128.95, 128.78, 126.55, 126.52 (q, J_{C-F} = 278.76 Hz), 83.55, 66.12 (q, J_{C-F} = 34.4 Hz), 55.40, 35.61, 22.75, 20.95.

^{19}F NMR (377 MHz, $CDCl_3$) δ -74.12.

HRMS (ESI) calculated for $C_{20}H_{25}F_3NO_3S^+$, $[M+H]^+$: 416.15018; found: 416.15013.

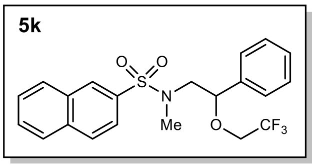

N-Methyl-N-(2-phenyl-2-(2,2,2-trifluoroethoxy)ethyl)naphthalene-2-sulfonamide 5k

The reaction was carried out following the procedure A using N-methylnaphthalene-2-sulfonamide, styrene and 2,2,2-trifluoroethanol. Then 69.8 mg colorless oily liquid was obtained in 55% isolated yield following purification procedure A.

^{1}H NMR (400 MHz, $CDCl_3$) δ 8.35 (d, J = 4.0 Hz, 1H), 7.98–7.93 (m, 2H), 7.90 (d, J = 8.0 Hz, 1H), 7.74 (dd, J = 8.0, 4.0 Hz, 1H), 7.67–7.58 (m, 2H), 7.42–7.36 (m, 3H), 7.36–7.32 (m, 2H), 4.75 (dd, J = 8.0, 4.0 Hz, 1H), 3.76–3.62 (m, 2H), 3.50 (dd, J = 16.0, 4.0 Hz, 1H), 3.19 (dd, J = 16.0, 8.0 Hz, 1H), 2.92 (s, 3H).

^{13}C NMR (101 MHz, $CDCl_3$) δ 137.41, 135.16, 134.79, 132.21, 129.44, 129.21, 129.00, 128.93, 128.79, 128.51, 127.91, 127.59, 126.76, 126.53 (q, J_{C-F} = 279.77 Hz), 122.51, 84.12, 66.20 (q, J_{C-F} = 34.4 Hz), 56.45, 37.36.

^{19}F NMR (377 MHz, $CDCl_3$) δ -74.14.

HRMS (ESI) calculated for $C_{21}H_{21}F_3NO_3S^+$, $[M+H]^+$: 424.11888; found: 424.11866.

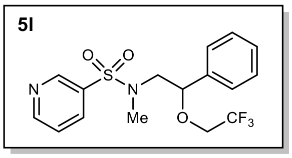

N-Methyl-N-(2-phenyl-2-(2,2,2-trifluoroethoxy)ethyl)pyridine-3-sulfonamide 5l

The reaction was carried out following the procedure A using *N*-methylpyridine-3-sulfonamide, styrene and 2,2,2-trifluoroethanol. Then 55.0 mg light yellow oily liquid was obtained in 49% isolated yield following purification procedure A.

^{1}H NMR (400 MHz, $CDCl_3$) δ 8.99 (d, J = 4.0 Hz, 1H), 8.79 (d, J = 4.0, 1H), 8.07–8.02 (m, 1H), 7.45 (dd, J = 8.0, 4.0 Hz, 1H), 7.42–7.35 (m, 3H), 7.33–7.30 (m, 2H), 4.71 (dd, J = 8.0, 4.0 Hz, 1H), 3.78–3.56 (m, 2H), 3.43 (dd, J = 16.0, 4.0 Hz, 1H), 3.21 (dd, J = 16.0, 8.0 Hz, 1H), 2.92 (s, 3H).

^{13}C NMR (101 MHz, $CDCl_3$) δ 153.21, 147.94, 136.97, 135.06, 134.83, 129.11, 129.09, 126.70, 126.48 (q, $J_{C\text{-}F}$ = 279.77 Hz), 123.80, 83.69, 66.06 (q, $J_{C\text{-}F}$ = 34.4 Hz), 56.26, 36.96.

^{19}F NMR (377 MHz, $CDCl_3$) δ -74.12.

HRMS (ESI) calculated for $C_{16}H_{18}F_3N_2O_3S^+$, $[M+H]^+$: 375.09847; found: 375.09787.

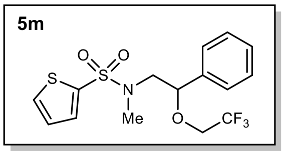

N-Methyl-N-(2-phenyl-2-(2,2,2-trifluoroethoxy)ethyl)thiophene-2-sulfonamide 5m

The reaction was carried out following the procedure A using *N*-methylthiophene-2-sulfonamide, styrene and 2,2,2-trifluoroethanol. Then 60.3 mg light yellow oily liquid was obtained in 53% isolated yield following purification procedure A.

^{1}H NMR (400 MHz, $CDCl_3$) δ 7.57 (d, J = 4.0 Hz, 1H), 7.54 (d, J = 4.0 Hz, 1H), 7.43–7.40 (m, 1H), 7.39–7.37 (m, 2H), 7.35–7.32 (m, 2H), 7.10 (dd, J = 4.0, 4.0 Hz, 1H), 4.73 (dd, J = 8.0, 4.0 Hz, 1H), 3.80–3.60 (m, 2H), 3.42 (dd, J = 16.0, 4.0 Hz, 1H), 3.12 (dd, J = 16.0, 8.0 Hz, 1H), 2.90 (s, 3H).

^{13}C NMR (101 MHz, $CDCl_3$) δ 138.17, 137.32, 131.92, 131.68, 129.01, 128.95, 127.51, 126.72, 126.51 (q, $J_{C\text{-}F}$ = 279.77 Hz), 83.95, 66.25 (q, $J_{C\text{-}F}$ = 34.5 Hz), 56.67, 37.48.

^{19}F NMR (377 MHz, $CDCl_3$) δ -74.18.

HRMS (ESI) calculated for $C_{15}H_{17}F_3NO_3S_2^+$, $[M+H]^+$: 380.05965; found: 380.05947.

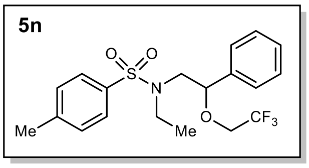

N-Ethyl-4-methyl-N-(2-phenyl-2-(2,2,2-trifluoroethoxy)ethyl)benzenesulfon-amide 5n

The reaction was carried out following the procedure A using *N*-ethyl-4-methylbenzenesulfonamide, styrene and 2,2,2-trifluoroethanol. Then 72.2 mg light yellow oily liquid was obtained in 60% isolated yield following purification procedure A.

^{1}H NMR (400 MHz, $CDCl_3$) δ 7.69 (d, *J* = 8.3 Hz, 2H), 7.42–7.36 (m, 2H), 7.36–7.31 (m, 3H), 7.28 (d, *J* = 8.0 Hz, 2H), 4.75 (dd, *J* = 8.0, 4.0 Hz, 1H), 3.76–3.57 (m, 2H), 3.45–3.35 (m, 2H), 3.28–3.14 (m, 2H), 2.41 (s, 3H), 1.04 (t, *J* = 8.0 Hz, 3H).

^{13}C NMR (101 MHz, $CDCl_3$) δ 143.29, 137.74, 137.12, 129.68, 128.93, 128.77, 127.14, 126.69, 126.51 (q, $J_{C\text{-}F}$ = 279.77 Hz), 83.67, 66.31 (q, $J_{C\text{-}F}$ = 34.4 Hz), 53.70, 44.78, 21.46, 13.41.

^{19}F NMR (377 MHz, $CDCl_3$) δ -74.11.

HRMS (ESI) calculated for $C_{19}H_{23}F_3NO_3S_2^+$, $[M+H]^+$: 402.13453; found: 402.13328.

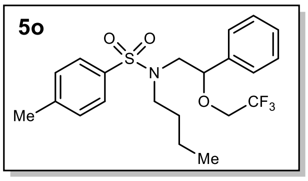

N-Butyl-4-methyl-N-(2-phenyl-2-(2,2,2-trifluoroethoxy)ethyl)benzenesulfon-amide 5o

The reaction was carried out following the procedure A using *N*-butyl-4-methylbenzenesulfonamide, styrene and 2,2,2-trifluoroethanol. Then 65.6 mg light yellow oily liquid was obtained in 51% isolated yield following purification procedure A.

^{1}H NMR (400 MHz, $CDCl_3$) δ 7.69 (d, *J* = 8.3 Hz, 2H), 7.41–7.36 (m, 2H), 7.36–7.31 (m, 3H), 7.28 (d, *J* = 8.0 Hz, 2H), 4.74 (dd, *J* = 8.0, 4.0 Hz, 1H), 3.74–3.53 (m, 2H), 3.40 (dd, *J* = 16.0, 4.0 Hz, 1H), 3.34–3.21 (m, 2H), 3.13–3.02 (m, 1H), 2.41 (s, 3H), 1.54–1.36 (m, 2H), 1.27–1.14 (m, 2H), 0.85 (t, *J* = 8.0 Hz, 3H).

^{13}C NMR (101 MHz, $CDCl_3$) δ 143.27, 137.79, 136.97, 129.65, 128.93, 128.76, 127.19, 126.69, 126.26 (q, $J_{C\text{-}F}$ = 278.76 Hz), 83.52, 66.24 (q, $J_{C\text{-}F}$ = 34.4 Hz), 54.32, 49.90, 30.12, 21.46, 19.82, 13.62.

^{19}F NMR (377 MHz, $CDCl_3$) δ -74.10.

HRMS (ESI) calculated for $C_{21}H_{27}F_3NO_3S^+$, $[M+H]^+$: 430.16583; found: 430.16571.

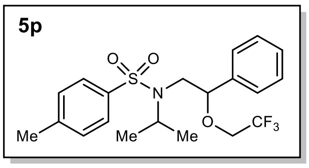

N-Isopropyl-4-methyl-N-(2-phenyl-2-(2,2,2-trifluoroethoxy)ethyl)benzenesulfonamide 5p

The reaction was carried out following the procedure A using *N*-isopropyl-4-methylbenzenesulfonamide, styrene and 2,2,2-trifluoroethanol. Then 53.5 mg light yellow oily liquid was obtained in 43% isolated yield following purification procedure A.

^{1}H NMR (400 MHz, $CDCl_3$) δ 7.72 (d, J = 8.0 Hz, 2H), 7.41 (s, 2H), 7.40 (s, 2H), 7.38–7.32 (m, 1H), 7.28 (d, J = 8.0 Hz, 2H), 4.95 (dd, J = 8.0, 4.0 Hz, 1H), 4.04 – 3.92 (m, 1H), 3.79 – 3.62 (m, 2H), 3.21 (dd, J = 16.0, 4.0 Hz, 2H), 2.41 (s, 3H), 1.12 (d, J = 4.0 Hz, 3H), 0.73 (d, J = 4.0 Hz, 3H).

^{13}C NMR (101 MHz, $CDCl_3$) δ 143.32, 138.29, 137.31, 129.67, 128.87, 128.61, 127.24, 126.76, 126.55 (q, $J_{C\text{-}F}$ = 278.76 Hz), 83.06, 66.53 (q, $J_{C\text{-}F}$ = 34.4 Hz), 50.20, 49.57, 21.50, 21.43, 19.75.

^{19}F NMR (377 MHz, $CDCl_3$) δ -73.97.

HRMS (ESI) calculated for $C_{20}H_{25}F_3NO_3S^+$, $[M+H]^+$: 416.15018; found: 416.14993.

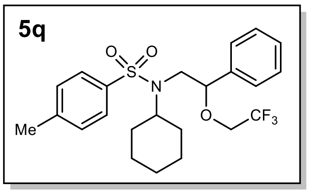

N-Cyclohexyl-4-methyl-N-(2-phenyl-2-(2,2,2-trifluoroethoxy)ethyl)benzenesulfonamide 5q

The reaction was carried out following the procedure A using *N*-cyclohexyl-4-methylbenzenesulfonamide, styrene and 2,2,2-trifluoroethanol. Then 54.6 mg light yellow oily liquid was obtained in 40% isolated yield following purification procedure A.

^{1}H NMR (400 MHz, $CDCl_3$) δ 7.72 (d, J = 8.0 Hz, 2H), 7.41–7.37 (m, 4H), 7.36–7.31 (m, 1H), 7.27 (d, J = 8.0 Hz, 2H), 4.91 (dd, J = 8.0, 4.0 Hz, 1H), 3.76–3.61 (m, 2H), 3.57–3.49 (m, 1H), 3.32–3.20 (m, 2H), 2.41 (s, 3H), 1.79–1.69 (m, 2H), 1.61–1.42 (m, 3H), 1.24–0.96 (m, 5H).

^{13}C NMR (101 MHz, $CDCl_3$) δ 143.21, 138.28, 137.83, 129.65, 128.84, 128.57, 127.08, 126.75, 126.55 (q, $J_{C\text{-}F}$ = 279.77 Hz), 83.31, 66.53 (q, $J_{C\text{-}F}$ = 34.3 Hz), 58.83, 50.56, 32.02, 30.44, 26.18, 26.04, 25.29, 21.50.

^{19}F NMR (377 MHz, $CDCl_3$) δ -73.96.

HRMS (ESI) calculated for $C_{23}H_{29}F_3NO_3S^+$, $[M+H]^+$: 456.18148; found: 456.18153.

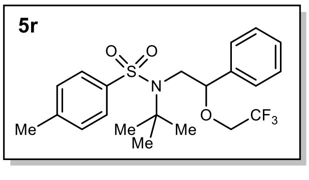

N-(*tert*-Butyl)-4-methyl-N-(2-phenyl-2-(2,2,2-trifluoroethoxy)ethyl)benzene-sulfonamide 5r

The reaction was carried out following the procedure A using *N*-(tert-butyl)-4-methylbenzenesulfonamide, styrene and 2,2,2-trifluoroethanol. Then 56.6 mg light yellow oily liquid was obtained in 44% isolated yield following purification procedure A.

^{1}H NMR (400 MHz, $CDCl_3$) δ 7.77 (d, J = 8.0 Hz, 2H), 7.41–7.39 (m, 4H), 7.37–7.33 (m, 1H), 7.27 (d, J = 8.0 Hz, 2H), 4.98 (dd, J = 8.0, 4.0 Hz, 1H), 3.83–3.65 (m, 2H), 3.62 (dd, J = 16.0, 4.0 Hz, 1H), 3.48 (dd, J = 16.0, 8.0 Hz, 1H), 2.41 (s, 3H), 1.33 (s, 9H).

^{13}C NMR (101 MHz, $CDCl_3$) δ 142.82, 141.33, 138.17, 129.49, 128.97, 128.64, 126.80, 126.65, 126.65 (q, $J_{C\text{-}F}$ = 278.76 Hz), 83.42, 66.34 (q, $J_{C\text{-}F}$ = 34.3 Hz), 59.55, 52.12, 29.67, 21.45.

^{19}F NMR (377 MHz, $CDCl_3$) δ -73.83.

HRMS (ESI) calculated for $C_{21}H_{26}F_3NNaO_3S^+$, $[M+Na]^+$: 452.14777; found: 452.14632.

5s

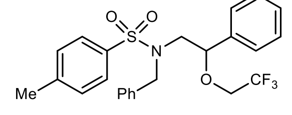

N-Benzyl-4-methyl-N-(2-phenyl-2-(2,2,2-trifluoroethoxy)ethyl)benzenesulfon-amide 5s

The reaction was carried out following the procedure A using *N*-benzyl-4-methylbenzenesulfonamide, styrene and 2,2,2-trifluoroethanol. Then 20.8 mg colorless oily liquid was obtained in 15% isolated yield following purification procedure A.

^{1}H NMR (400 MHz, $CDCl_3$) δ 7.79 (d, J = 8.0 Hz, 2H), 7.38–7.32 (m, 5H), 7.33–7.28 (m, 3H), 7.22–7.17 (m, 4H), 4.62 (dd, J = 8.0, 4.0 Hz, 1H), 4.56 (d, J = 4.0 Hz, 2H), 3.60 (dq, J = 12.0, 8.0 Hz, 1H), 3.45–3.29 (m, 3H), 2.47 (s, 3H).

^{13}C NMR (101 MHz, $CDCl_3$) δ 143.42, 137.73, 137.40, 136.01, 129.71, 128.88, 128.78, 128.61, 128.54, 127.72, 127.22, 126.63, 126.05 (q, $J_{C\text{-}F}$ = 278.76 Hz), 82.95, 65.92 (q, $J_{C\text{-}F}$ = 34.4 Hz), 52.56, 52.43, 21.52.

^{19}F NMR (377 MHz, $CDCl_3$) δ -74.00.

HRMS (ESI) calculated for $C_{24}H_{25}F_3NO_3S^+$, $[M+H]^+$: 464.15018; found: 464.14986.

5t

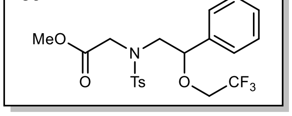

Methyl N-(2-phenyl-2-(2,2,2-trifluoroethoxy)ethyl)-N-tosylglycinate 5t

The reaction was carried out following the procedure A using methyl tosylglycinate, styrene and 2,2,2-trifluoroethanol. Then 20.7 mg white solid was obtained in 17% isolated yield following purification procedure A.

^{1}H NMR (400 MHz, CDCl$_3$) δ 7.63 (d, *J* = 8.0 Hz, 2H), 7.38–7.29 (m, 7H), 5.06 (dd, *J* = 8.0, 4.0 Hz, 1H), 4.04–3.91 (m, 2H), 3.90–3.77 (m, 2H), 3.42 (s, 3H), 2.47 (d, *J* = 12.0 Hz, 1H), 2.43 (s, 3H), 2.35 (d, *J* = 12.0 Hz, 1H).

^{13}C NMR (101 MHz, CDCl$_3$) δ 144.25, 136.97, 132.58, 129.94, 128.65, 128.63, 127.66, 126.50 (q, $J_{C\text{-}F}$ = 277.75 Hz), 126.13, 110.02, 74.03, 59.88 (q, $J_{C\text{-}F}$ = 35.8 Hz), 50.58, 49.90, 47.78, 21.56.

^{19}F NMR (377 MHz, CDCl$_3$) δ -73.44.

HRMS (ESI) calculated for $C_{20}H_{22}F_3NNaO_5S^+$, $[M+Na]^+$: 468.10630; found: 468.10548.

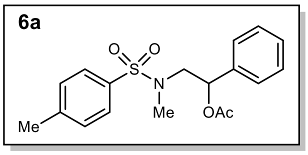

2-((*N*,4-Dimethylphenyl)sulfonamido)-1-phenylethyl acetate 6a

The reaction was carried out following the procedure B using *N*-methyl-*p*-toluenesulfonamide, styrene and acetic acid (36%). Then 70.8 mg colorless oily liquid was obtained in 68% isolated yield following purification procedure B.

^{1}H NMR (400 MHz, CDCl$_3$) δ 7.63 (d, *J* = 8.0 Hz, 2H), 7.39–7.31 (m, 5H), 7.28 (d, *J* = 8.0 Hz, 2H), 5.96 (dd, *J* = 8.0, 4.0 Hz, 1H), 3.53 (dd, *J* = 16.0, 8.0 Hz, 1H), 3.16 (dd, *J* = 16.0, 4.0 Hz, 1H), 2.75 (s, 3H), 2.40 (s, 3H), 2.10 (s, 3H).

^{13}C NMR (101 MHz, CDCl$_3$) δ 170.07, 143.50, 137.57, 134.82, 129.75, 128.71, 128.56, 127.35, 126.45, 73.25, 55.04, 36.15, 21.52, 21.17.

HRMS (ESI) calculated for $C_{18}H_{22}NO_4S^+$, $[M+H]^+$: 348.12641; found: 348.12612.

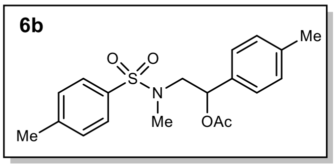

2-((*N*,4-Dimethylphenyl)sulfonamido)-1-(*p*-tolyl)ethyl acetate 6b

The reaction was carried out following the procedure B using *N*-methyl-*p*-toluenesulfonamide, 4-methylstyrene and acetic acid (36%). Then 49.8 mg colorless oily liquid was obtained in 46% isolated yield following purification procedure B.

^{1}H NMR (400 MHz, CDCl$_3$) δ 7.63 (d, *J* = 8.0 Hz, 2H), 7.28 (d, *J* = 8.0 Hz, 2H), 7.24 (d, *J* = 8.0 Hz, 2H), 7.16 (d, *J* = 8.0 Hz, 2H), 5.92 (dd, *J* = 8.0, 4.0 Hz, 1H), 3.53 (dd, *J* = 16.0, 8.0 Hz, 1H), 3.14 (dd, *J* = 16.0, 4.0 Hz, 1H), 2.75 (s, 3H), 2.41 (s, 3H), 2.34 (s, 3H), 2.09 (s, 3H).

^{13}C NMR (101 MHz, $CDCl_3$) δ 170.09, 143.42, 138.41, 134.89, 134.57, 129.70, 129.36, 127.36, 126.63, 73.06, 54.96, 36.05, 21.50, 21.20, 21.18.
HRMS (ESI) calculated for $C_{19}H_{24}NO_4S^+$, $[M+H]^+$: 362.14206; found: 362.14094.

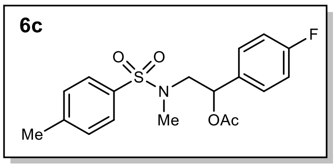

2-((*N*,4-Dimethylphenyl)sulfonamido)-1-(4-fluorophenyl)ethyl acetate 6c

The reaction was carried out following the procedure B using *N*-methyl-*p*-toluenesulfonamide, 4-fluorostyrene and acetic acid (36%). Then 63.5 mg colorless oily liquid was obtained in 58% isolated yield following purification procedure B.
^1H NMR (400 MHz, $CDCl_3$) δ 7.62 (d, J = 8.0 Hz, 2H), 7.36–7.31 (m, 2H), 7.29 (d, J = 8.0 Hz, 2H), 7.04 (t, J = 8.0 Hz, 2H), 5.93 (dd, J = 8.0, 4.0 Hz, 1H), 3.48 (dd, J = 16.0, 8.0 Hz, 1H), 3.17 (dd, J = 16.0, 4.0 Hz, 1H), 2.74 (s, 3H), 2.41 (s, 3H), 2.09 (s, 3H).
^{13}C NMR (101 MHz, $CDCl_3$) δ 169.98, 162.70 (d, $J_{C\text{-}F}$ = 247.3 Hz), 143.56, 134.77, 133.39 (d, $J_{C\text{-}F}$ = 3.2 Hz), 129.76, 128.53 (d, $J_{C\text{-}F}$ = 8.3 Hz), 127.33, 115.66 (d, $J_{C\text{-}F}$ = 21.6 Hz), 72.70, 54.86, 36.19, 21.50, 21.12.
^{19}F NMR (377 MHz, $CDCl_3$) δ -113.10.
HRMS (ESI) calculated for $C_{18}H_{21}FNO_4S^+$, $[M+H]^+$: 366.11698; found: 366.11678.

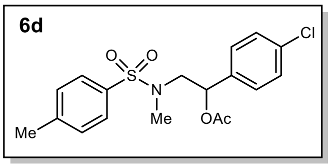

1-(4-Chlorophenyl)-2-((*N*,4-dimethylphenyl)sulfonamido)ethyl acetate 6d

The reaction was carried out following the procedure B using *N*-methyl-*p*-toluenesulfonamide, 4-chlorostyrene and acetic acid (36%). Then 48.0 mg colorless oily liquid was obtained in 42% isolated yield following purification procedure B.
^1H NMR (400 MHz, $CDCl_3$) δ 7.61 (d, J = 8.0 Hz, 2H), 7.32 (d, J = 8.0 Hz, 2H), 7.28 (d, J = 8.0 Hz, 4H), 5.91 (dd, J = 8.0, 4.0 Hz, 1H), 3.47 (dd, J = 16.0, 8.0 Hz, 1H), 3.17 (dd, J = 16.0, 4.0 Hz, 1H), 2.74 (s, 3H), 2.41 (s, 3H), 2.10 (s, 3H).
^{13}C NMR (101 MHz, $CDCl_3$) δ 169.94, 143.59, 136.07, 134.73, 134.42, 129.76, 128.90, 128.08, 127.33, 72.74, 54.79, 36.24, 21.51, 21.10.
HRMS (ESI) calculated for $C_{18}H_{21}ClNO_4S^+$, $[M+H]^+$: 382.08743; found: 382.08705.

6e

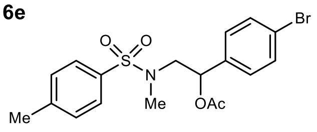

1-(4-Bromophenyl)-2-((*N*,4-dimethylphenyl)sulfonamido)ethyl acetate 6e

The reaction was carried out following the procedure B using N-methyl-*p*-toluenesulfonamide, 4-bromostyrene and acetic acid (36%). Then 63.8 mg colorless oily liquid was obtained in 50% isolated yield following purification procedure B.

^{1}H NMR (400 MHz, $CDCl_3$) δ 7.61 (d, *J* = 8.0 Hz, 2H), 7.47 (d, *J* = 8.0 Hz, 2H), 7.28 (d, *J* = 8.0 Hz, 2H), 7.22 (d, *J* = 8.0 Hz, 2H), 5.89 (dd, *J* = 8.0, 4.0 Hz, 1H), 3.46 (dd, *J* = 16.0, 8.0 Hz, 1H), 3.17 (dd, *J* = 16.0, 4.0 Hz, 1H), 2.74 (s, 3H), 2.41 (s, 3H), 2.10 (s, 3H).

^{13}C NMR (101 MHz, $CDCl_3$) δ 169.93, 143.60, 136.59, 134.72, 131.86, 129.77, 128.38, 127.32, 122.58, 72.80, 54.73, 36.25, 21.52, 21.09.

HRMS (ESI) calculated for $C_{18}H_{21}BrNO_4S^+$, $[M+H]^+$: 426.03692; found: 426.03638.

6f

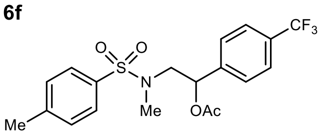

2-((*N*,4-Dimethylphenyl)sulfonamido)-1-(4-(trifluoromethyl)phenyl)ethyl acetate 6f

The reaction was carried out following the procedure B using *N*-methyl-*p*-toluenesulfonamide, 4-(trifluoromethyl)styrene and acetic acid (36%). Then 42.3 mg colorless oily liquid was obtained in 34% isolated yield following purification procedure B.

^{1}H NMR (400 MHz, $CDCl_3$) δ 7.62 (s, 2H), 7.60 (s, 2H), 7.47 (d, *J* = 8.0 Hz, 2H), 7.28 (d, *J* = 8.0 Hz, 2H), 5.99 (dd, *J* = 8.0, 4.0 Hz, 1H), 3.48 (dd, *J* = 16.0, 8.0 Hz, 1H), 3.22 (dd, *J* = 16.0, 4.0 Hz, 1H), 2.75 (s, 3H), 2.41 (s, 3H), 2.12 (s, 3H).

^{13}C NMR (101 MHz, $CDCl_3$) δ 169.89, 143.68, 141.55, 134.64, 130.54, 129.79, 127.32, 126.99, 126.66 (q, J_{C-F} = 272.70 Hz), 125.69 (q, J_{C-F} = 3.8 Hz), 72.94, 54.81, 36.33, 21.49, 21.03.

^{19}F NMR (377 MHz, $CDCl_3$) δ -62.66.

HRMS (ESI) calculated for $C_{19}H_{21}F_3NO_4S^+$, $[M+H]^+$: 416.11379; found: 416.11394.

6g

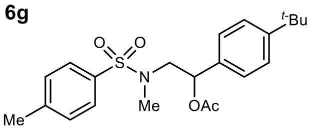

1-(4-(*tert*-Butyl)phenyl)-2-((*N*,4-dimethylphenyl)sulfonamido)ethyl acetate 6g

The reaction was carried out following the procedure B using *N*-methyl-*p*-toluenesulfonamide, 4-*tert*-butylstyrene and acetic acid (36%). Then 38.7 mg light yellow oily liquid was obtained in 32% isolated yield following purification procedure B.

NMR (400 MHz, $CDCl_3$) δ 7.63 (d, *J* = 8.0 Hz, 2H), 7.37 (d, *J* = 8.0 Hz, 2H), 7.29 (d, *J* = 4.0 Hz, 2H), 7.27 (d, *J* = 4.0 Hz, 2H), 5.95 (dd, *J* = 8.0, 4.0 Hz, 1H), 3.56 (dd, *J* = 16.0, 8.0 Hz, 1H), 3.13 (dd, *J* = 16.0, 4.0 Hz, 1H), 2.77 (s, 3H), 2.40 (s, 3H), 2.09 (s, 3H), 1.30 (s, 9H).

^{13}C NMR (101 MHz, $CDCl_3$) δ 170.16, 151.59, 143.43, 134.89, 134.46, 129.71, 127.37, 126.42, 125.61, 72.93, 54.97, 36.01, 34.62, 31.30, 21.50, 21.19.

HRMS (ESI) calculated for $C_{22}H_{30}NO_4S^+$, $[M+H]^+$: 404.18901; found: 404.18820.

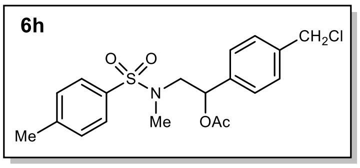

1-(4-(Chloromethyl)phenyl)-2-((*N*,4-dimethylphenyl)sulfonamido)ethyl acetate 6h

The reaction was carried out following the procedure B using *N*-methyl-*p*-toluenesulfonamide, 4-(chloromethyl)styrene and acetic acid (36%). Then 49.8 mg colorless oily liquid was obtained in 42% isolated yield following purification procedure B.

^{1}H NMR (400 MHz, $CDCl_3$) δ 7.62 (d, *J* = 8.0 Hz, 2H), 7.38 (d, *J* = 8.0 Hz, 2H), 7.34 (d, *J* = 8.0 Hz, 2H), 7.29 (d, *J* = 8.0 Hz, 2H), 5.95 (dd, *J* = 8.0, 4.2 Hz, 1H), 4.57 (s, 2H), 3.50 (dd, *J* = 16.0, 8.0 Hz, 1H), 3.17 (dd, *J* = 16.0, 4.0 Hz, 1H), 2.75 (s, 3H), 2.40 (s, 3H), 2.10 (s, 3H).

^{13}C NMR (101 MHz, $CDCl_3$) δ 170.01, 143.56, 137.82, 134.73, 129.77, 128.93, 127.35, 127.27, 127.05, 73.01, 54.91, 45.74, 36.19, 21.51, 21.12.

HRMS (ESI) calculated for $C_{19}H_{23}ClNO_4S^+$, $[M+H]^+$: 396.10308; found: 396.10263.

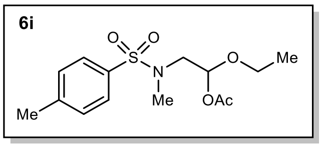

2-((*N*,4-Dimethylphenyl)sulfonamido)-1-ethoxyethyl acetate 6i

The reaction was carried out following the procedure B using *N*-methyl-*p*-toluenesulfonamide, ethyl vinyl ether and acetic acid (36%). Then 31.2 mg colorless oily liquid was obtained in 33% isolated yield following purification procedure B.

^{1}H NMR (400 MHz, $CDCl_3$) δ 7.66 (d, *J* = 8.0 Hz, 2H), 7.30 (d, *J* = 8.0 Hz, 2H), 5.91 (t, *J* = 4.0 Hz, 1H), 3.78–3.70 (m, 1H), 3.58–3.52 (m, 1H), 3.29 (dd, *J* = 16.0, 4.0 Hz,

1H), 3.11 (dd, J = 16.0, 4.0 Hz, 1H), 2.84 (s, 3H), 2.41 (s, 3H), 2.08 (s, 3H), 1.16 (t, J = 8.0 Hz, 3H).
^{13}C NMR (101 MHz, $CDCl_3$) δ 170.53, 143.48, 135.00, 129.72, 127.34, 95.76, 65.74, 52.58, 36.90, 21.50, 21.12, 15.02.
HRMS (ESI) calculated for $C_{14}H_{21}NNaO_5S^+$, $[M+Na]^+$: 338.10326; found: 338.10248.

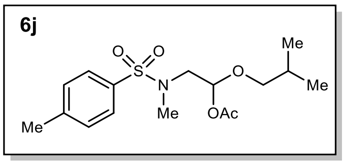

2-((*N*,4-Dimethylphenyl)sulfonamido)-1-isobutoxyethyl acetate 6j
The reaction was carried out following the procedure B using *N*-methyl-*p*-toluenesulfonamide, isobutyl vinyl ether and acetic acid (36%). Then 36.0 mg colorless oily liquid was obtained in 35% isolated yield following purification procedure B.
^{1}H NMR (400 MHz, $CDCl_3$) δ 7.66 (d, J = 8.0 Hz, 2H), 7.30 (d, J = 8.0 Hz, 2H), 5.90 (t, J = 4.0 Hz, 1H), 3.45 (dd, J = 8.0, 4.0 Hz, 1H), 3.34–3.23 (m, 2H), 3.12 (dd, J = 16.0, 4.0 Hz, 1H), 2.84 (s, 3H), 2.41 (s, 3H), 2.08 (s, 3H), 1.85−1.74 (m, 1H), 0.87 (s, 3H), 0.86 (s, 3H).
^{13}C NMR (101 MHz, $CDCl_3$) δ 170.54, 143.46, 135.04, 129.73, 127.33, 96.12, 52.49, 36.94, 28.42, 21.50, 21.13, 19.15.
HRMS (ESI) calculated for $C_{16}H_{25}NNaO_5S^+$, $[M+Na]^+$: 366.13456; found: 366.13416.

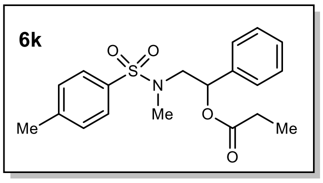

2-((*N*,4-Dimethylphenyl)sulfonamido)-1-phenylethyl propionate 6k
The reaction was carried out following the procedure C using *N*-methyl-*p*-toluenesulfonamide, styrene and propionic acid. Then 69.3 mg colorless oily liquid was obtained in 64% isolated yield following purification procedure C.
^{1}H NMR (400 MHz, $CDCl_3$) δ 7.62 (d, J = 8.0 Hz, 2H), 7.36–7.31 (m, 5H), 7.28 (d, J = 8.0 Hz, 2H), 5.97 (dd, J = 8.0, 4.0 Hz, 1H), 3.54 (dd, J = 16.0, 8.0 Hz, 1H), 3.16 (dd, J = 16.0, 4.0 Hz, 1H), 2.75 (s, 3H), 2.44–2.35 (m, 5H), 1.15 (t, J = 8.0 Hz, 3H).
^{13}C NMR (101 MHz, $CDCl_3$) δ 173.48, 143.44, 137.72, 134.89, 129.72, 128.69, 128.50, 127.34, 126.59, 73.02, 55.11, 36.11, 27.72, 21.50, 9.02.
HRMS (ESI) calculated for $C_{19}H_{24}NO_4S^+$, $[M+H]^+$: 362.14206; found: 362.14164.

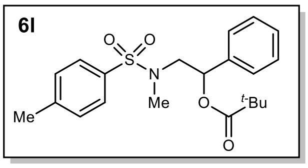

2-((*N*,4-Dimethylphenyl)sulfonamido)-1-phenylethyl pivalate 6l

The reaction was carried out following the procedure C using *N*-methyl-*p*-toluenesulfonamide, styrene and pivalic acid. Then 58.4 mg white solid was obtained in 50% isolated yield following purification procedure C.

^{1}H NMR (400 MHz, $CDCl_3$) δ 7.61 (d, *J* = 8.0 Hz, 2H), 7.40–7.35 (m, 1H), 7.34–7.30 (m, 4H), 7.28 (d, *J* = 8.0 Hz, 2H), 5.94 (dd, *J* = 8.0, 4.0 Hz, 1H), 3.56 (dd, *J* = 16.0, 8.0 Hz, 1H), 3.11 (dd, *J* = 16.0, 4.0 Hz, 1H), 2.77 (s, 3H), 2.40 (s, 3H), 1.25 (s, 9H).

^{13}C NMR (101 MHz, $CDCl_3$) δ 177.47, 143.43, 137.97, 134.75, 129.74, 128.69, 128.40, 127.33, 126.35, 72.92, 55.36, 38.84, 36.10, 27.18, 21.49.

HRMS (ESI) calculated for $C_{21}H_{28}NO_4S^+$, $[M+H]^+$: 390.17336; found: 390.17320.

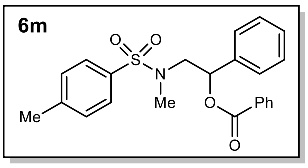

2-((*N*,4-Dimethylphenyl)sulfonamido)-1-phenylethyl benzoate 6m

The reaction was carried out following the procedure C using *N*-methyl-*p*-toluenesulfonamide, styrene and benzoic acid. Then 73.6 mg colorless oily liquid was obtained in 60% isolated yield following purification procedure C.

^{1}H NMR (400 MHz, $CDCl_3$) δ 8.13–8.09 (m, 2H), 7.64 (d, *J* = 8.0 Hz, 2H), 7.60–7.55 (m, 1H), 7.46 (dd, *J* = 8.0, 8.0 Hz, 4H), 7.41–7.31 (m, 3H), 7.24 (d, *J* = 8.0 Hz, 2H), 6.19 (dd, *J* = 8.0, 4.0 Hz, 1H), 3.75 (dd, *J* = 16.0, 8.0 Hz, 1H), 3.31 (dd, *J* = 16.0, 4.0 Hz, 1H), 2.83 (s, 3H), 2.38 (s, 3H).

^{13}C NMR (101 MHz, $CDCl_3$) δ 165.60, 143.46, 137.69, 134.94, 133.22, 129.93, 129.83, 129.74, 128.78, 128.62, 128.47, 127.34, 126.57, 74.02, 55.12, 36.20, 21.50.

HRMS (ESI) calculated for $C_{23}H_{24}NO_4S^+$, $[M+H]^+$: 410.14206; found: 410.14189.

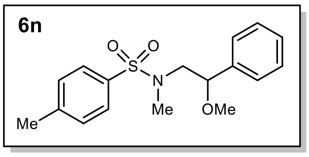

***N*-(2-Methoxy-2-phenylethyl)-*N*,4-dimethylbenzenesulfonamide 6n**

The reaction was carried out following the procedure D using *N*-methyl-*p*-toluenesulfonamide, styrene and methanol. Then 57.4 mg white solid was obtained in 60% isolated yield following purification procedure D.

^{1}H NMR (400 MHz, $CDCl_3$) δ 7.64 (d, J = 8.0 Hz, 2H), 7.39–7.34 (m, 2H), 7.33–7.30 (m, 3H), 7.28 (d, J = 8.0 Hz, 2H), 4.43 (dd, J = 8.0, 4.0 Hz, 1H), 3.24 (dd, J = 16.0, 4.0 Hz, 1H), 3.21 (s, 3H), 3.12 (dd, J = 16.0, 8.0 Hz, 1H), 2.79 (s, 3H), 2.40 (s, 3H).
^{13}C NMR (101 MHz, $CDCl_3$) δ 143.22, 139.31, 135.18, 129.62, 128.62, 128.17, 127.31, 126.79, 83.85, 56.86, 56.64, 37.16, 21.49.
HRMS (ESI) calculated for $C_{17}H_{22}NO_3S^+$, $[M+H]^+$: 320.13149; found: 320.13106.

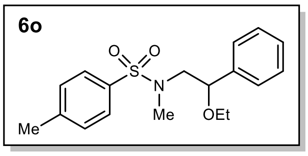

***N*-(2-Ethoxy-2-phenylethyl)-*N*,4-dimethylbenzenesulfonamide 6o**
The reaction was carried out following the procedure D using *N*-methyl-*p*-toluenesulfonamide, styrene and ethanol. Then 43.9 mg light yellow oily liquid was obtained in 44% isolated yield following purification procedure D.
^{1}H NMR (400 MHz, $CDCl_3$) δ 7.64 (d, J = 8.0 Hz, 2H), 7.38–7.34 (m, 1H), 7.34–7.30 (m, 4H), 7.28 (d, J = 8.0 Hz, 2H), 4.54 (dd, J = 8.0, 4.0 Hz, 1H), 3.43–3.32 (m, 2H), 3.29 (dd, J = 16.0, 4.0 Hz, 1H), 3.07 (dd, J = 16.0, 8.0 Hz, 1H), 2.81 (s, 3H), 2.40 (s, 3H), 1.15 (t, J = 8.0 Hz, 3H).
^{13}C NMR (101 MHz, $CDCl_3$) δ 143.18, 140.01, 135.31, 129.62, 128.55, 128.00, 127.29, 126.69, 82.21, 64.56, 56.73, 37.24, 21.49, 15.34.
HRMS (ESI) calculated for $C_{18}H_{24}NO_3S^+$, $[M+H]^+$: 334.14714; found: 334.14678.

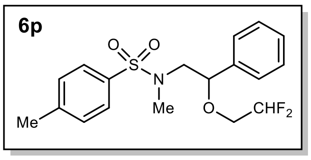

***N*-(2-(2,2-Difluoroethoxy)-2-phenylethyl)-*N*,4-dimethylbenzenesulfonamide 6p**
The reaction was carried out following the procedure D using *N*-methyl-*p*-toluenesulfonamide, styrene and 2,2-difluoroethanol. Then 67.5 mg light yellow oily liquid was obtained in 61% isolated yield following purification procedure D.
^{1}H NMR (400 MHz, $CDCl_3$) δ 7.65 (d, J = 8.3 Hz, 2H), 7.41 – 7.27 (m, 7H), 5.80 (tt, J = 55.4, 4.0 Hz, 1H), 4.64 (dd, J = 8.0, 4.0 Hz, 1H), 3.63 – 3.43 (m, 2H), 3.33 (dd, J = 16.0, 4.0 Hz, 2H), 3.12 (dd, J = 16.0, 8.0 Hz, 2H), 2.82 (s, 3H), 2.41 (s, 3H).
^{13}C NMR (101 MHz, $CDCl_3$) δ 143.40, 138.14, 135.14, 129.71, 128.87, 128.67, 127.27, 126.73, 114.17 (t, $J_{C\text{-}F}$ = 241.0 Hz), 83.57, 68.19 (t, $J_{C\text{-}F}$ = 27.7 Hz), 56.52, 37.30, 21.51.
^{19}F NMR (377 MHz, $CDCl_3$) δ -125.27.
HRMS (ESI) calculated for $C_{18}H_{22}F_2NO_3S^+$, $[M+H]^+$: 370.12830; found:.370.12863.

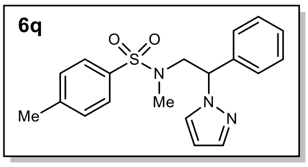

N,4-Dimethyl-N-(2-phenyl-2-(1H-pyrazol-1-yl)ethyl)benzenesulfonamide 6q

The reaction was carried out following the procedure E using *N*-methyl-*p*-toluenesulfonamide, styrene and pyrazole. Then 24.5 mg colorless oily liquid was obtained in 23% isolated yield following purification procedure E.

^{1}H NMR (400 MHz, $CDCl_3$) δ 7.65 (d, J = 8.0 Hz, 2H), 7.56 (d, J = 16.0 Hz, 2H), 7.37–7.31 (m, 6H), 7.29 (s, 1H), 6.29 (s, 1H), 5.64 (dd, J = 8.0, 4.0 Hz, 1H), 3.84 (dd, J = 16.0, 8.0 Hz, 1H), 3.75 (dd, J = 16.0, 4.0 Hz, 1H), 2.48 (s, 3H), 2.42 (s, 3H).

^{13}C NMR (101 MHz, $CDCl_3$) δ 143.62, 139.80, 138.22, 134.50, 130.62, 129.81, 128.79, 128.32, 127.33, 127.06, 105.72, 66.30, 54.80, 36.64, 21.53.

HRMS (ESI) calculated for $C_{19}H_{22}N_3O_2S^+$, $[M+H]^+$: 356.14272; found: 356.14230.

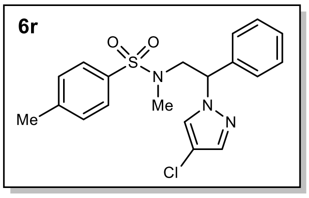

N-(2-(4-Chloro-1H-pyrazol-1-yl)-2-phenylethyl)-N,4-dimethylbenzenesulfon-amide 6r

The reaction was carried out following the procedure E using *N*-methyl-*p*-toluenesulfonamide, styrene and 4-chloropyrazole. Then 30.3 mg colorless oily liquid was obtained in 26% isolated yield following purification procedure E.

^{1}H NMR (400 MHz, $CDCl_3$) δ 7.65 (d, J = 8.0 Hz, 2H), 7.50 (d, J = 4.0 Hz, 2H), 7.35–7.31 (m, 6H), 7.30 (s, 1H), 5.57 (dd, J = 8.0, 4.0 Hz, 1H), 3.84 (dd, J = 16.0, 8.0 Hz, 1H), 3.68 (dd, J = 16.0, 4.0 Hz, 1H), 2.55 (s, 3H), 2.42 (s, 3H).

^{13}C NMR (101 MHz, $CDCl_3$) δ 143.77, 138.17, 137.51, 134.28, 129.86, 128.91, 128.64, 128.51, 127.35, 127.00, 110.26, 66.78, 54.43, 36.90, 21.54.

HRMS (ESI) calculated for $C_{19}H_{21}ClN_3O_2S^+$, $[M+H]^+$: 390.10375; found: 390.10357.

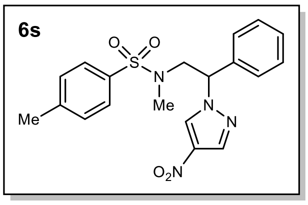

N,4-Dimethyl-N-(2-(4-nitro-1H-pyrazol-1-yl)-2-phenylethyl)benzenesulfonamide 6s

The reaction was carried out following the procedure E using *N*-methyl-*p*-toluenesulfonamide, styrene and 4-nitropyrazole. Then 14.4 mg colorless oily liquid was obtained in 12% isolated yield following purification procedure E.
^{1}H NMR (400 MHz, $CDCl_3$) δ 8.24 (s, 1H), 8.13 (s, 1H), 7.65 (d, *J* = 8.0 Hz, 2H), 7.38 (s, 5H), 7.32 (d, *J* = 8.0 Hz, 2H), 5.68 (dd, *J* = 8.0, 4.0 Hz, 1H), 3.89 (dd, *J* = 16.0, 8.0 Hz, 1H), 3.65 (dd, *J* = 16.0, 4.0 Hz, 1H), 2.60 (s, 3H), 2.43 (s, 3H).
^{13}C NMR (101 MHz, $CDCl_3$) δ 144.08, 136.01, 135.91, 133.85, 129.94, 129.68, 129.55, 129.29, 129.20, 127.40, 127.15, 67.58, 54.41, 37.27, 21.56.
HRMS (ESI) calculated for $C_{19}H_{21}N_4O_4S^+$, $[M+H]^+$: 401.12780; found: 401.12745.

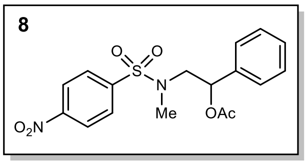

2-((*N*-methyl-4-nitrophenyl)sulfonamido)-1-phenylethyl acetate 8
The reaction was carried out following the procedure B using *N*-methyl-4-nitrobenzenesulfonamide, styrene and acetic acid (36%). Then 33.2 mg colorless oily liquid was obtained in 44% isolated yield following purification procedure B.
^{1}H NMR (400 MHz, $CDCl_3$) δ 8.33 (d, J = 8.0 Hz, 2H), 7.91 (d, J = 8.0 Hz, 2H), 7.44–7.28 (m, 5H), 5.97 (dd, J = 8.0, 4.0 Hz, 1H), 3.60 (dd, J = 16.0, 8.0 Hz, 1H), 3.27 (dd, J = 16.0, 4.0 Hz, 1H), 2.82 (s, 3H), 2.12 (s, 3H).
^{13}C NMR (101 MHz, $CDCl_3$) δ 170.01, 150.06, 143.96, 137.07, 128.86, 128.83, 128.46, 126.62, 124.43, 72.93, 54.95, 36.06, 21.14.
HRMS (ESI) calculated for $C_{17}H_{18}N_2NaO_6S^+$, $[M+Na]^+$: 401.07778; found: 401.07758.

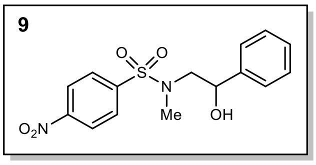

***N*-(2-hydroxy-2-phenylethyl)-*N*-methyl-4-nitrobenzenesulfonamide 9**
The reaction was carried out following the procedure for synthesis of halostachine using 2-((*N*-methyl-4-nitrophenyl)sulfonamido)-1-phenylethyl acetate, Potassium carbonate was added to in CH_2Cl_2 and MeOH and stirred at room temperature for 4 h. Concentrated under reduced pressure to give the crude product. Purification by silica gel flash column chromatography PE/EA (v: v = 2:1) gave the desired product **9**. Then 19.5 mg colorless oily liquid was obtained in 66% isolated yield.
^{1}H NMR (400 MHz, $CDCl_3$) δ 8.34 (d, J = 8.0 Hz, 2H), 7.96 (d, J = 8.0 Hz, 2H), 7.40–7.29 (m, 5H), 4.95 (dd, J = 8.0, 4.0 Hz, 1H), 3.34 (dd, J = 16.0, 8.0 Hz, 1H), 3.18 (dd, J = 16.0, 4.0 Hz, 1H), 2.89 (s, 3H).

^{13}C NMR (101 MHz, $CDCl_3$) δ 150.11, 143.65, 140.82, 128.78, 128.54, 128.38, 125.96, 124.44, 72.72, 57.91, 36.68.

10a

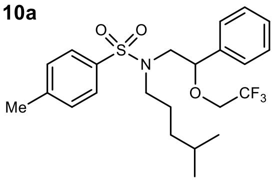

4-Methyl-*N*-(4-methylpentyl)-*N*-(2-phenyl-2-(2,2,2-trifluoroethoxy)ethyl)benzeneesulfonamide 10a

The reaction was carried out following the procedure A using 4-methyl-*N*-(4-methylpentyl)benzenesulfonamide, styrene and 2,2,2-trifluoroethanol. Then 20.6 mg colorless oily liquid was obtained in 15% isolated yield following purification procedure A.

^{1}H NMR (400 MHz, $CDCl_3$) δ 7.69 (d, J = 8.0 Hz, 2H), 7.43–7.28 (m, 5H), 7.28 (d, J = 8.0 Hz, 2H), 4.74 (dd, J = 8.0, 4.0 Hz, 1H), 3.76–3.52 (m, 2H), 3.39 (dd, J = 16.0, 4.0 Hz, 1H), 3.31–3.17 (m, 2H), 3.12–2.96 (m, 1H), 2.41 (s, 3H), 1.55–1.33 (m, 3H), 1.07–0.91 (m, 2H), 0.82 (s, 3H), 0.80 (s, 3H).

^{13}C NMR (101 MHz, $CDCl_3$) δ 143.28, 137.80, 136.98, 129.66, 128.94, 128.78, 127.21, 126.72, 126.50 (q, $J_{C\text{-}F}$ = 281.79 Hz), 83.54, 66.25 (q, $J_{C\text{-}F}$ = 34.3 Hz), 54.31, 50.42, 35.71, 27.70, 25.91, 22.49, 22.46, 21.50.

^{19}F NMR (377 MHz, CDCl3) δ -74.07.

HRMS (ESI) calculated for $C_{23}H_{31}F_3NO_3S^+$, $[M+H]^+$: 458.19713; found: 458.19703.

10b

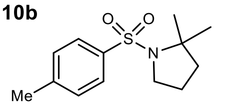

2,2-Dimethyl-1-tosylpyrrolidine 10b

The reaction was carried out following the procedure A using 4-methyl-*N*-(4-methylpentyl)benzenesulfonamide, styrene and 2,2,2-trifluoroethanol. Then 31.1 mg colorless oily liquid was obtained in 41% isolated yield following purification procedure A.

^{1}H NMR (400 MHz, $CDCl_3$) δ 7.72 (d, J = 8.0 Hz, 2H), 7.26 (d, J = 8.0 Hz, 2H), 3.38 (t, J = 6.4 Hz, 2H), 2.40 (s, 3H), 1.83 – 1.72 (m, 4H), 1.43 (s, 6H).

^{13}C NMR (101 MHz, $CDCl_3$) δ 142.55, 138.67, 129.36, 127.13, 65.10, 49.35, 42.91, 28.26, 22.49, 21.49.

HRMS (ESI) calculated for $C_{13}H_{20}NO_2S^+$, $[M+H]^+$: 254.12093; found: 254.12031.

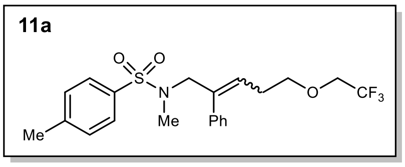

(*E/Z*)-*N*,4-Dimethyl-*N*-(2-phenyl-5-(2,2,2-trifluoroethoxy)pent-2-en-1-yl)benzene-sulfonamide 11a (Mixing Ratio = 3:2)

The reaction was carried out following the procedure A using *N*-methyl-*p*-toluenesulfonamide, (1-cyclopropylvinyl)benzene and 2,2,2-trifluoroethanol. Then 17.9 mg colorless oily liquid was obtained in 14% isolated yield following purification procedure A. The ratio of different products is determined by ^{1}**H NMR** spectrum and the result of (3 : 2) can be concluded by the integral of peak (5.66–5.96)

^{1}H NMR (400 MHz, $CDCl_3$) δ 7.63 (d, *J* = 8.0 Hz, 1.22H), 7.55 (d, *J* = 8.0 Hz, 0.78H), 7.41–7.38 (m, 1.23H), 7.36–7.25 (m, 4.23H), 7.25 (s, 0.57H), 7.18–7.14 (m, 0.77H), 5.94 (t, *J* = 8.0 Hz, 0.6H), 5.68 (t, *J* = 8.0 Hz, 0.4H), 4.08 (s, 1.21H), 3.88 (s, 0.78H), 3.81 (q, *J* = 8.0 Hz, 1.21H), 3.74 (q, *J* = 8.0 Hz, 0.79H), 3.68 (t, *J* = 8.0 Hz, 1.21H), 3.58 (t, *J* = 8.0 Hz, 0.78H), 2.62 (s, 1.16H), 2.51 (dd, *J* = 16.0, 4.0 Hz, 1.23H), 2.47 (s, 1.82H), 2.44 (s, 1.82H), 2.41 (s, 1.17H), 2.34 (dd, *J* = 16.0, 4.0 Hz, 0.80H).

^{13}C NMR (101 MHz, $CDCl_3$) δ 143.47, 143.23, 140.30, 137.67, 137.54, 136.45, 134.48, 133.59, 129.83, 129.69, 129.58, 128.52, 128.36, 128.35, 127.66, 127.49, 127.44, 127.02, 126.71 (q, $J_{C\text{-}F}$ = 278.76 Hz), 126.65, 71.96, 71.84, 68.41 (q, *J* = 34.0 Hz), 68.22 (q, *J* = 34.34 Hz), 56.93, 47.76, 33.96, 33.61, 29.72, 29.21, 28.87, 21.55.

^{19}F NMR (377 MHz, $CDCl_3$) δ *Z/E* (-74.16, -74.17.)

HRMS (ESI) calculated for $C_{21}H_{25}F_3NO_3S^+$, $[M+H]^+$: 428.15018; found: 428.15109.

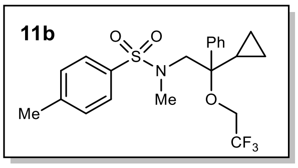

***N*-(2-Cyclopropyl-2-phenyl-2-(2,2,2-trifluoroethoxy)ethyl)-*N*,4-dimethylbenzene-sulfonamide 11b**

The reaction was carried out following the procedure A using *N*-methyl-*p*-toluenesulfonamide, (1-cyclopropylvinyl)benzene and 2,2,2-trifluoroethanol. Then 7.7 mg colorless oily liquid was obtained in 6% isolated yield following purification procedure A.

^{1}H NMR (400 MHz, $CDCl_3$) δ 7.55–7.50 (m, 4H), 7.41 –7.37 (m, 2H), 7.35–7.30 (m, 1H), 7.27 (s, 1H), 7.25 (s, 1H), 4.0–3.91 (m, 1H), 3.85–3.76 (m, 1H), 3.34 (d, *J* = 16.0 Hz, 1H), 3.21 (d, *J* = 16.0 Hz, 1H), 2.73 (s, 3H), 2.40 (s, 3H), 1.40–1.34 (m, 1H), 1.01–0.94 (m, 1H), 0.90–0.82 (m, 1H), 0.73–0.65 (m, 2H).

^{13}C NMR (101 MHz, $CDCl_3$) δ 143.35, 140.36, 134.24, 129.64, 128.35, 128.04, 127.49, 127.07, 126.97 (q, J_{C-F} = 278.76 Hz), 82.85, 66.22 (q, J_{C-F} = 34.34 Hz), 56.94, 37.11, 21.50, 17.26, 3.42, 2.31.
^{19}F NMR (377 MHz, $CDCl_3$) δ -73.83.
HRMS (ESI) calculated for $C_{21}H_{24}F_3NNaO_3S^+$, $[M+Na]^+$: 450.13212; found: 450.13204.

Copies of product NMR spectra

^{1}H NMR spectrum of 4aa (400 MHz, $CDCl_3$):

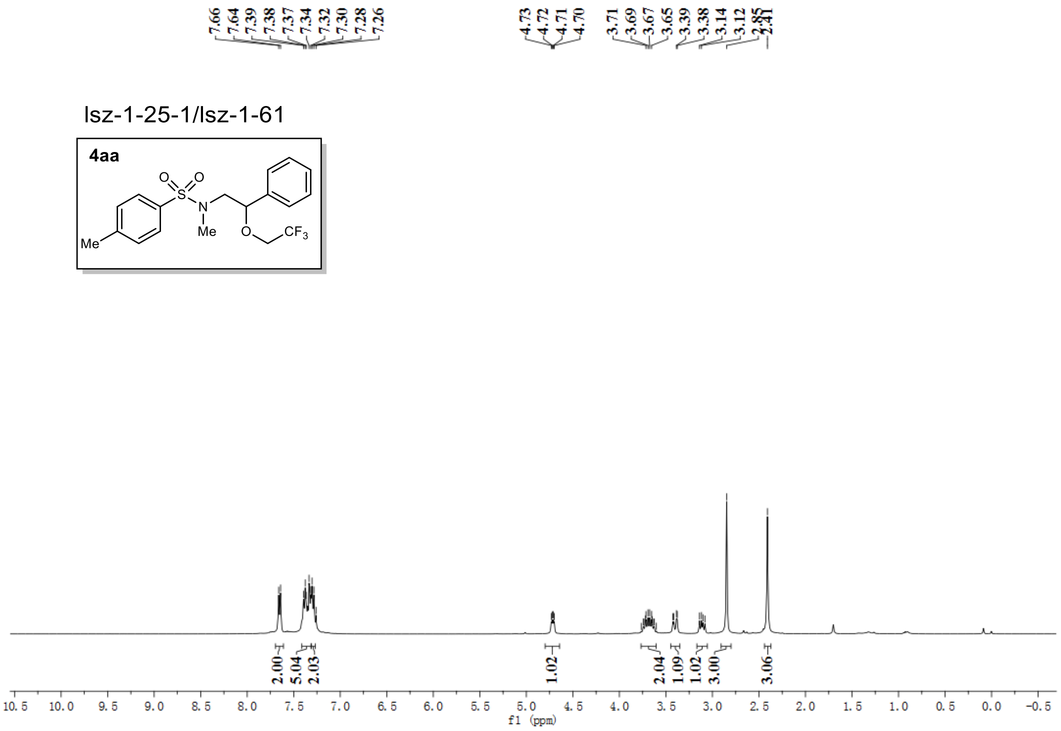

^{13}C NMR spectrum of 4aa (101 MHz, $CDCl_3$):

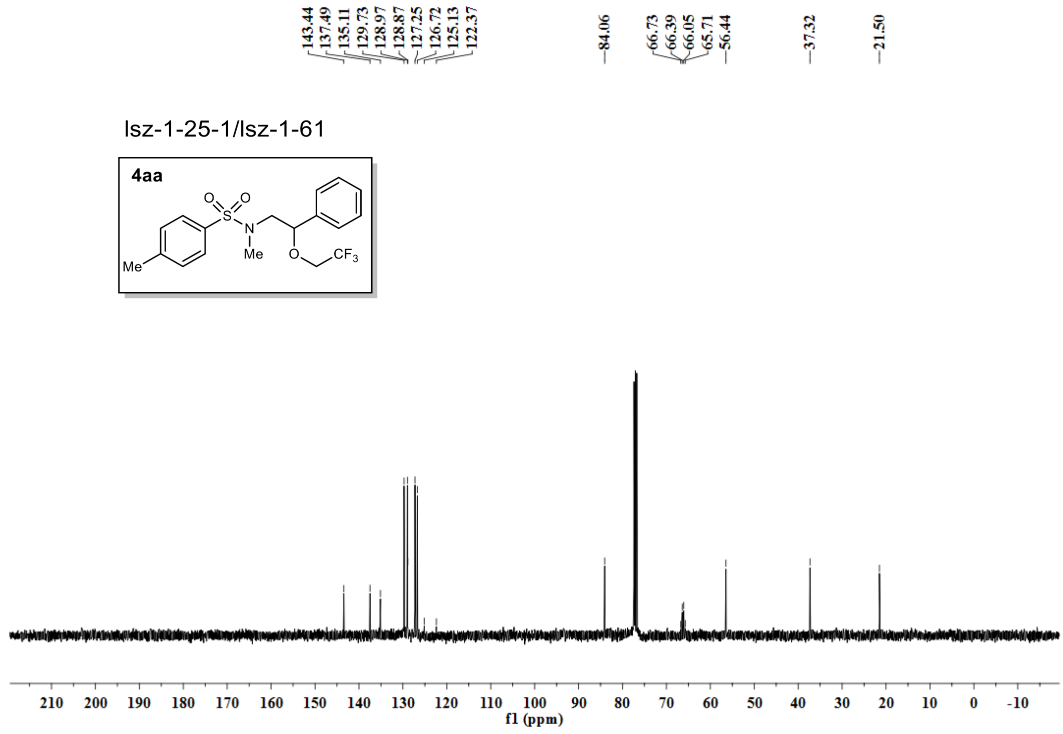

^{19}F NMR spectrum of 4aa (377 MHz, $CDCl_3$):

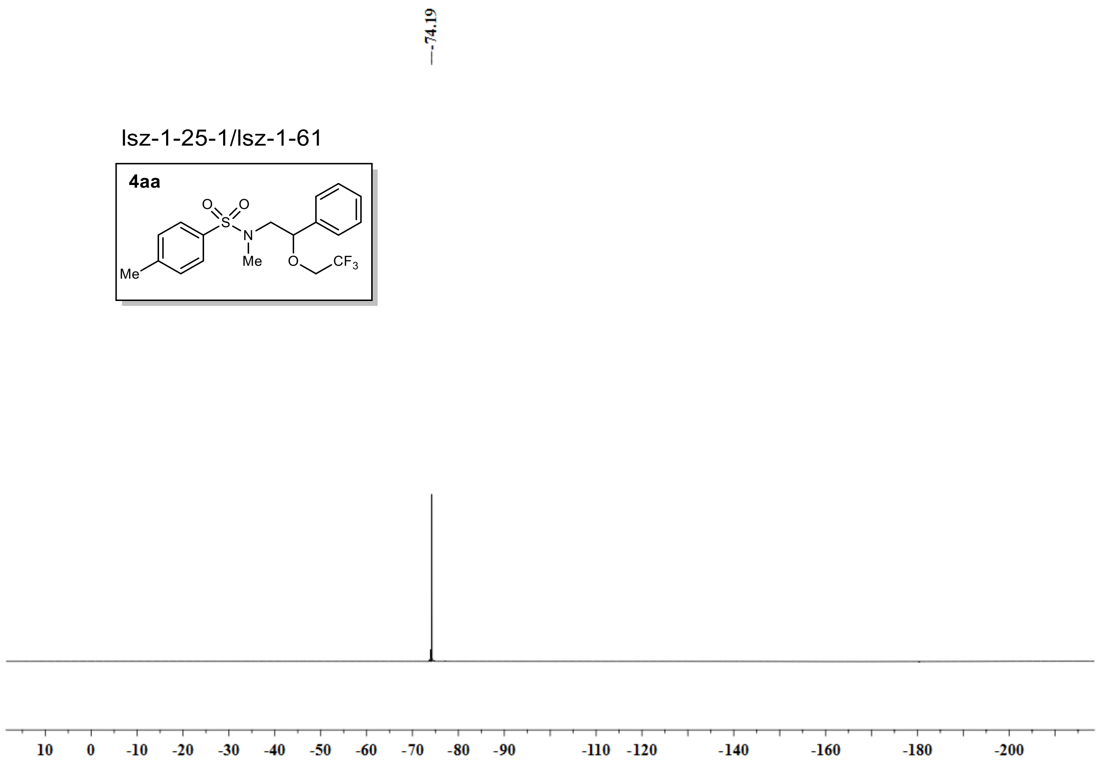

^{1}H NMR spectrum of 4ab (400 MHz, $CDCl_3$):

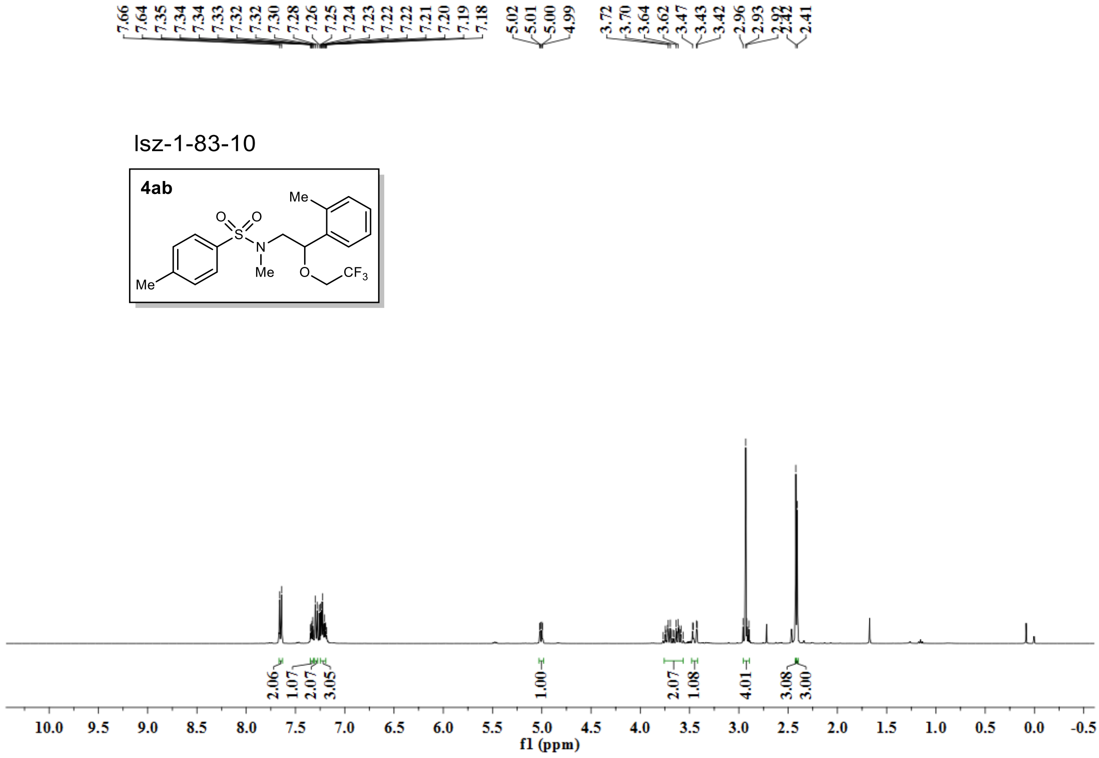

^{13}C NMR spectrum of 4ab (101 MHz, $CDCl_3$):

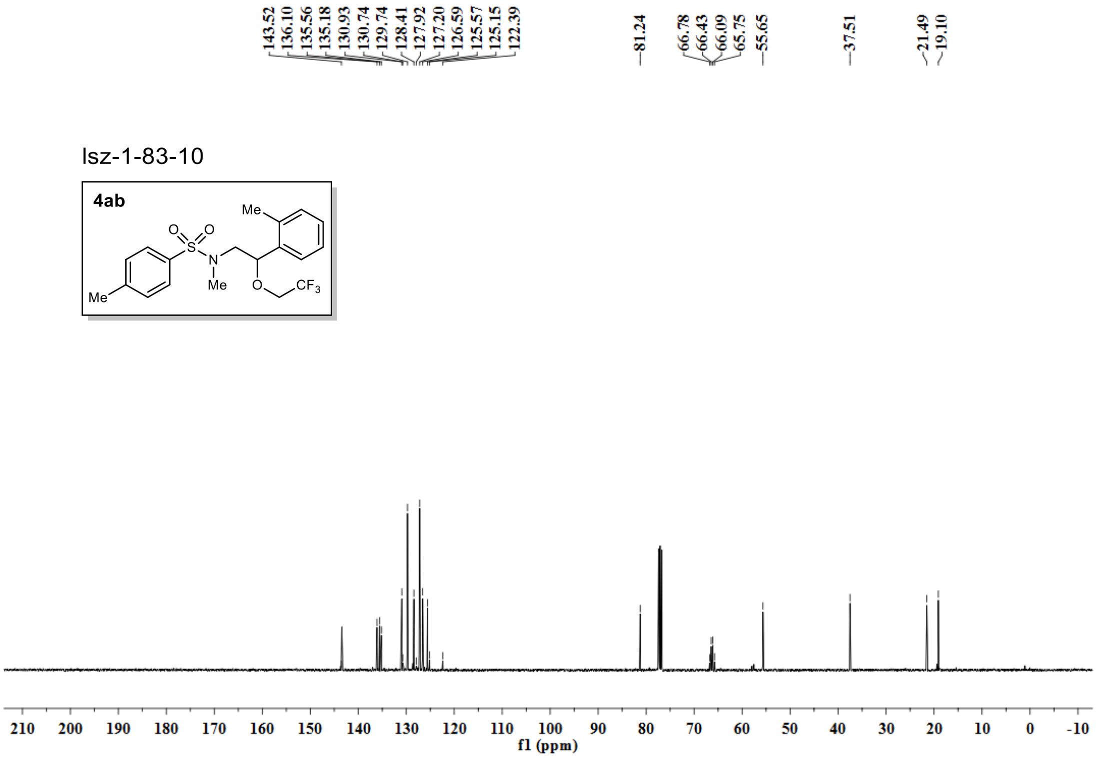

^{19}F NMR spectrum of 4ab (377 MHz, $CDCl_3$):

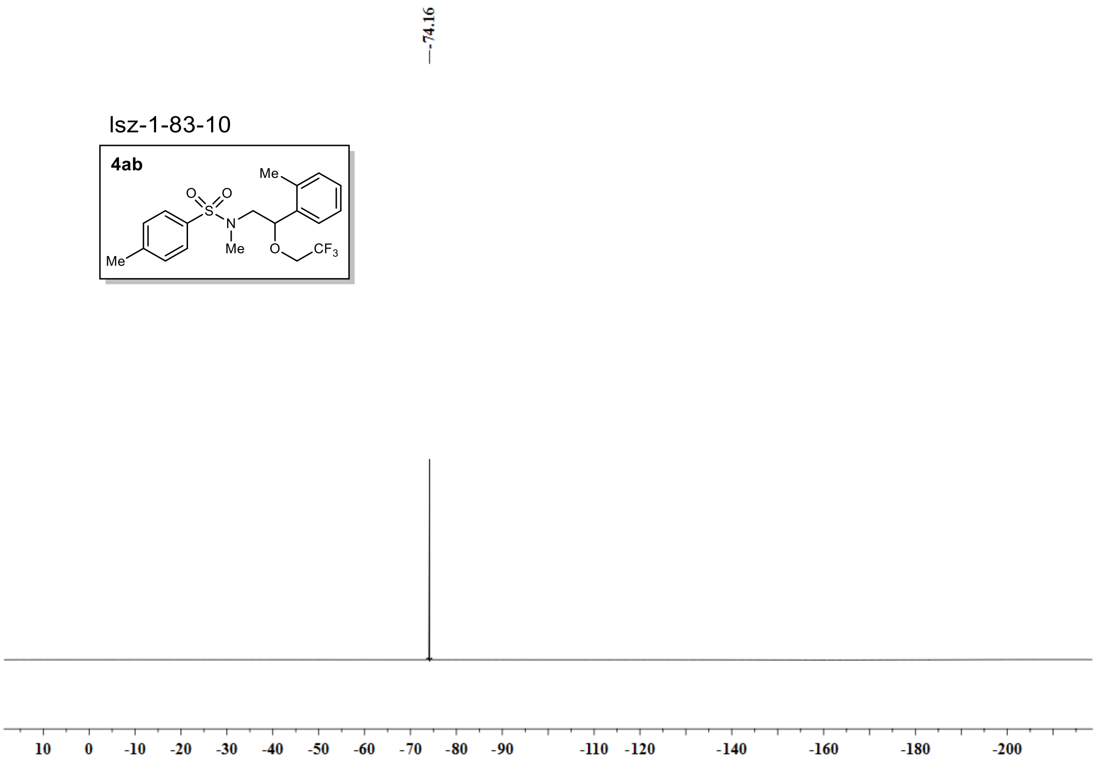

^{1}H NMR spectrum of 4ac (400 MHz, $CDCl_3$):

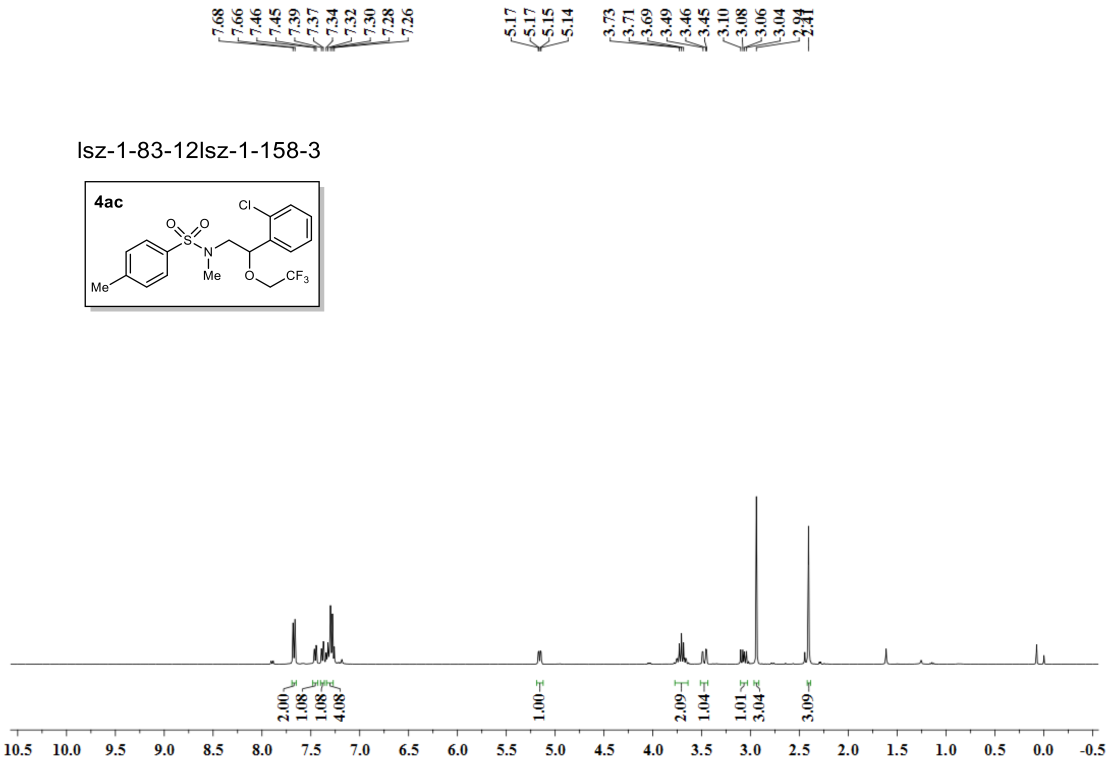

^{13}C NMR spectrum of 4ac (101 MHz, $CDCl_3$):

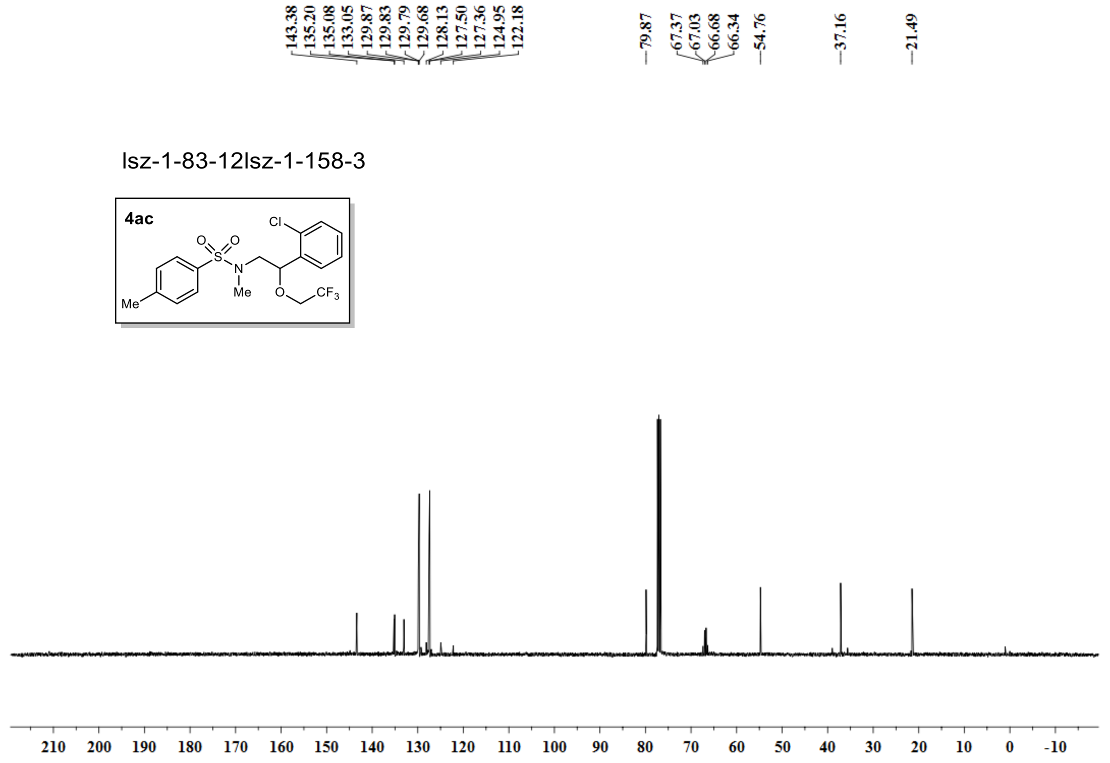

^{19}F NMR spectrum of 4ac (377 MHz, $CDCl_3$):

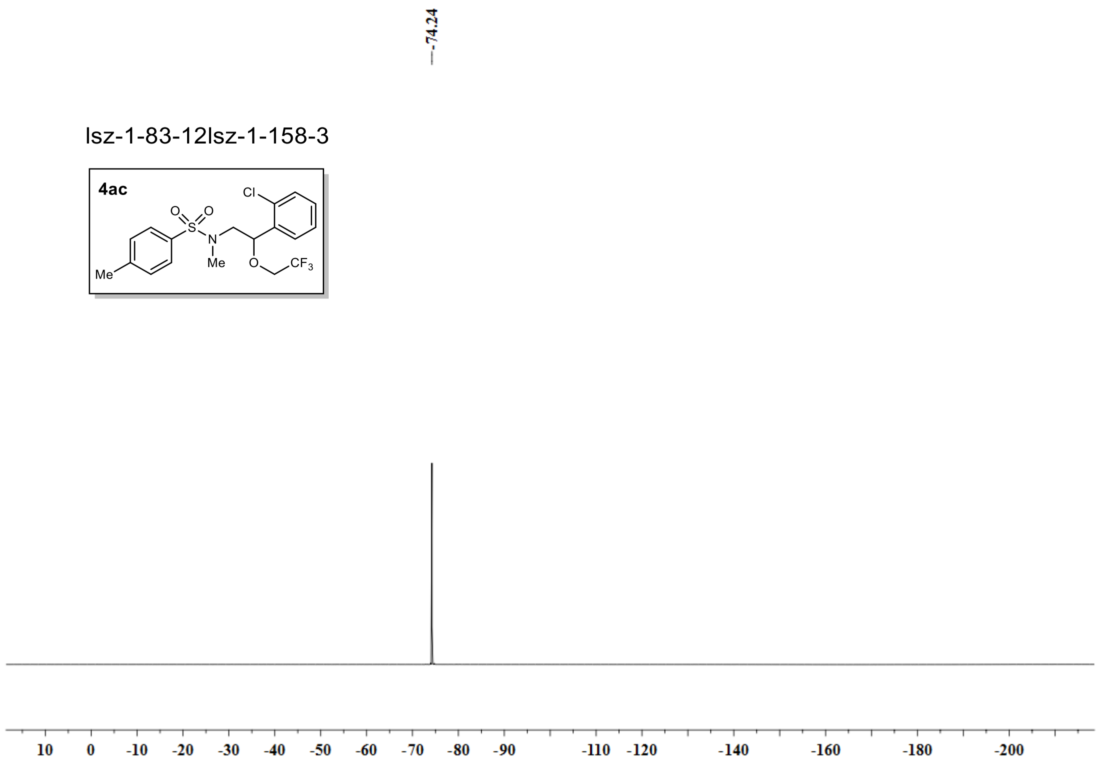

^{1}H NMR spectrum of 4ad (400 MHz, $CDCl_3$):

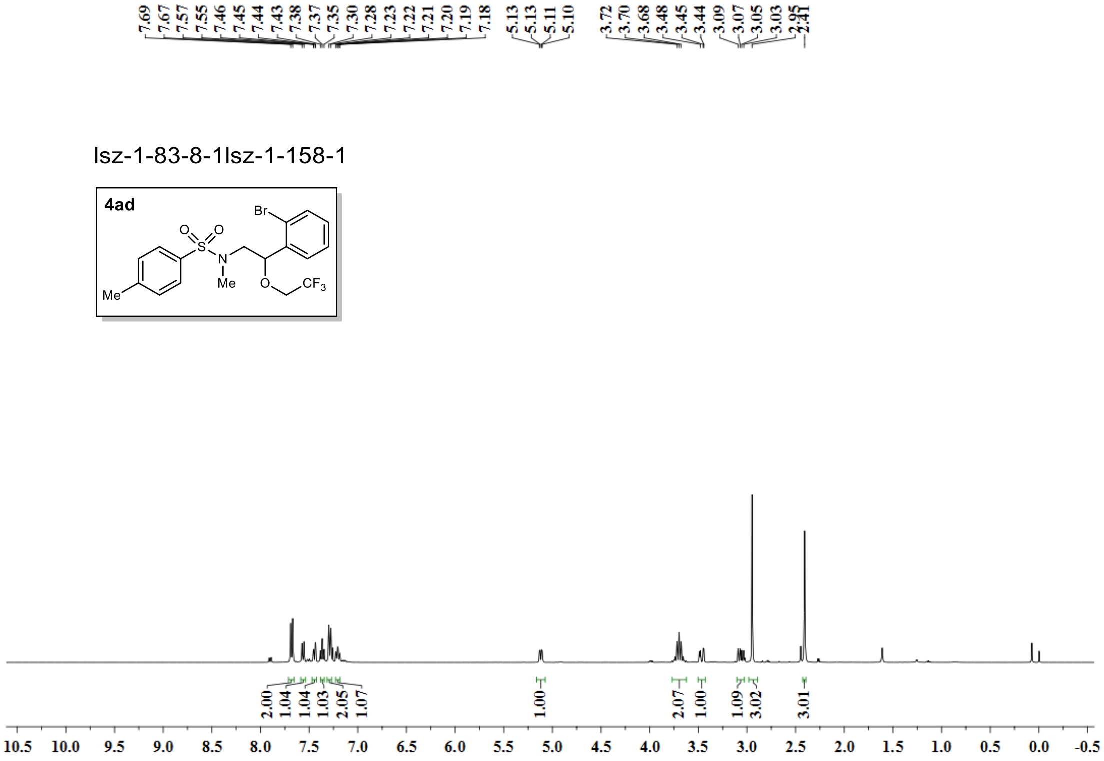

^{13}C NMR spectrum of 4ad (101 MHz, $CDCl_3$):

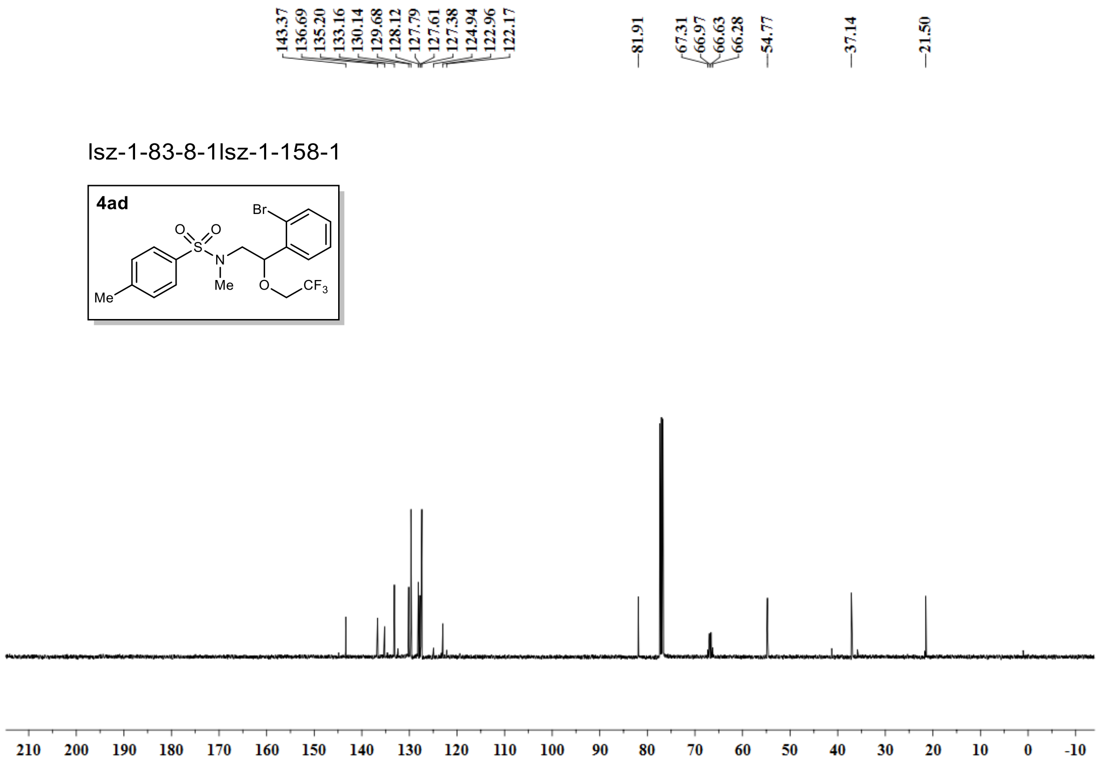

^{19}F NMR spectrum of 4ad (377 MHz, $CDCl_3$):

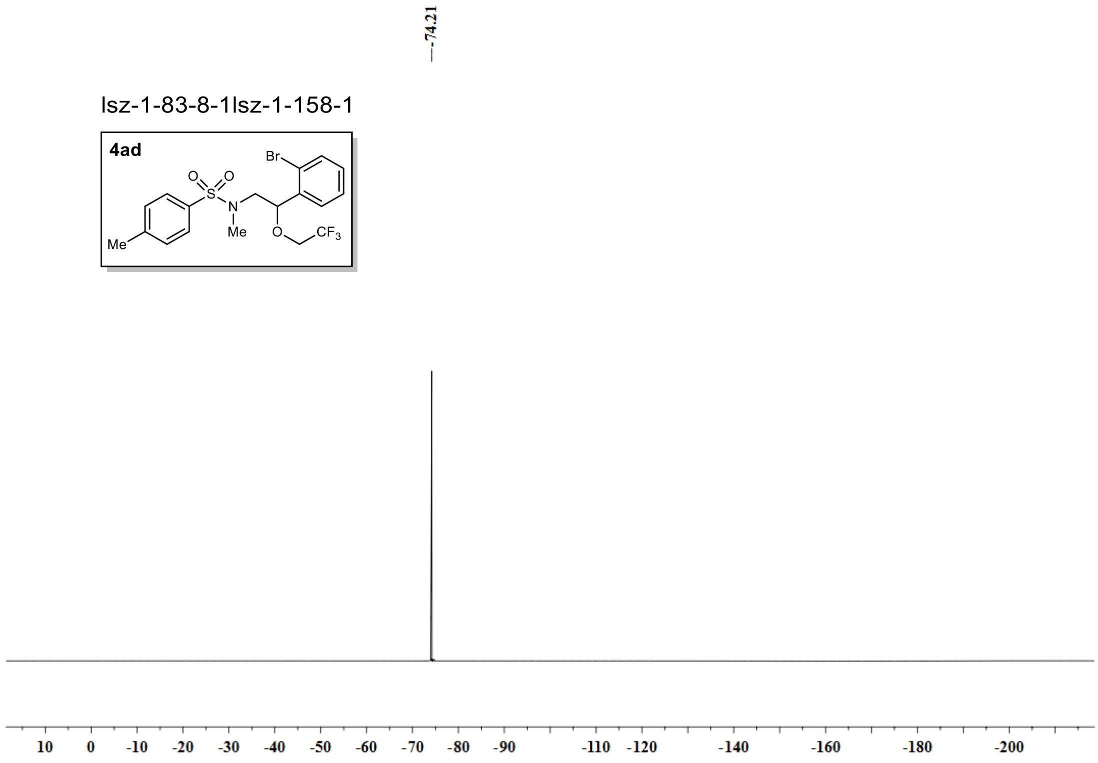

¹H NMR spectrum of 4ae (400 MHz, $CDCl_3$):

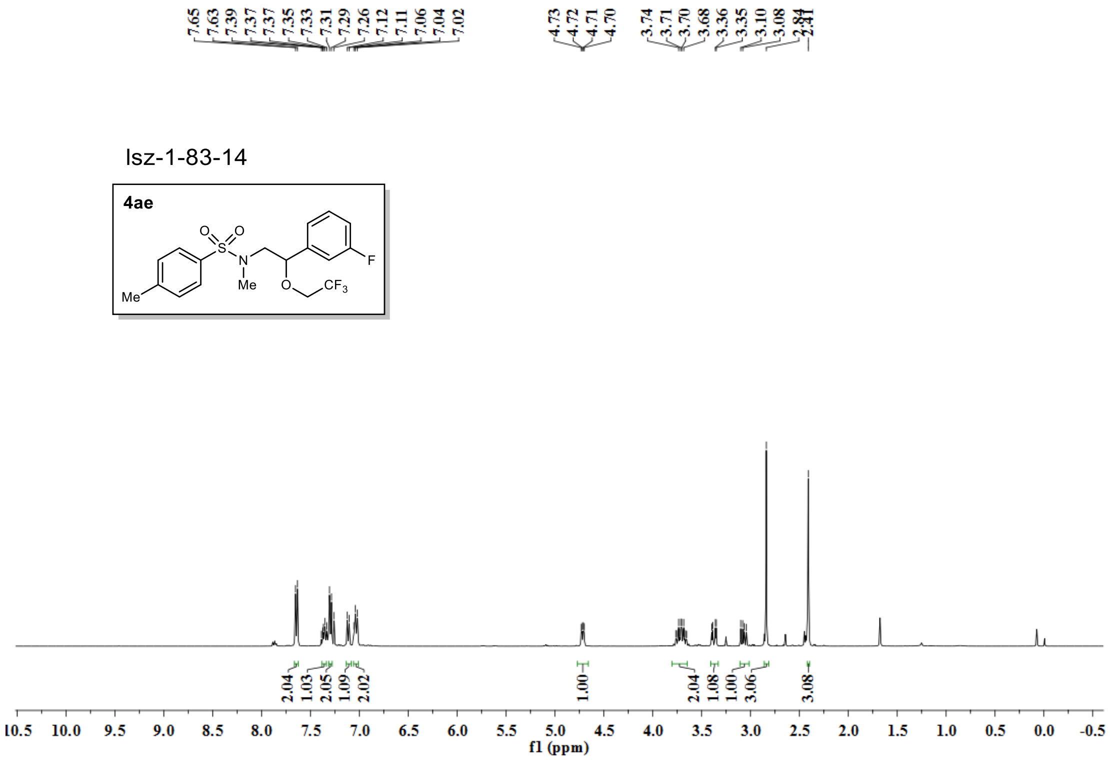

¹³C NMR spectrum of 4ae (101 MHz, $CDCl_3$):

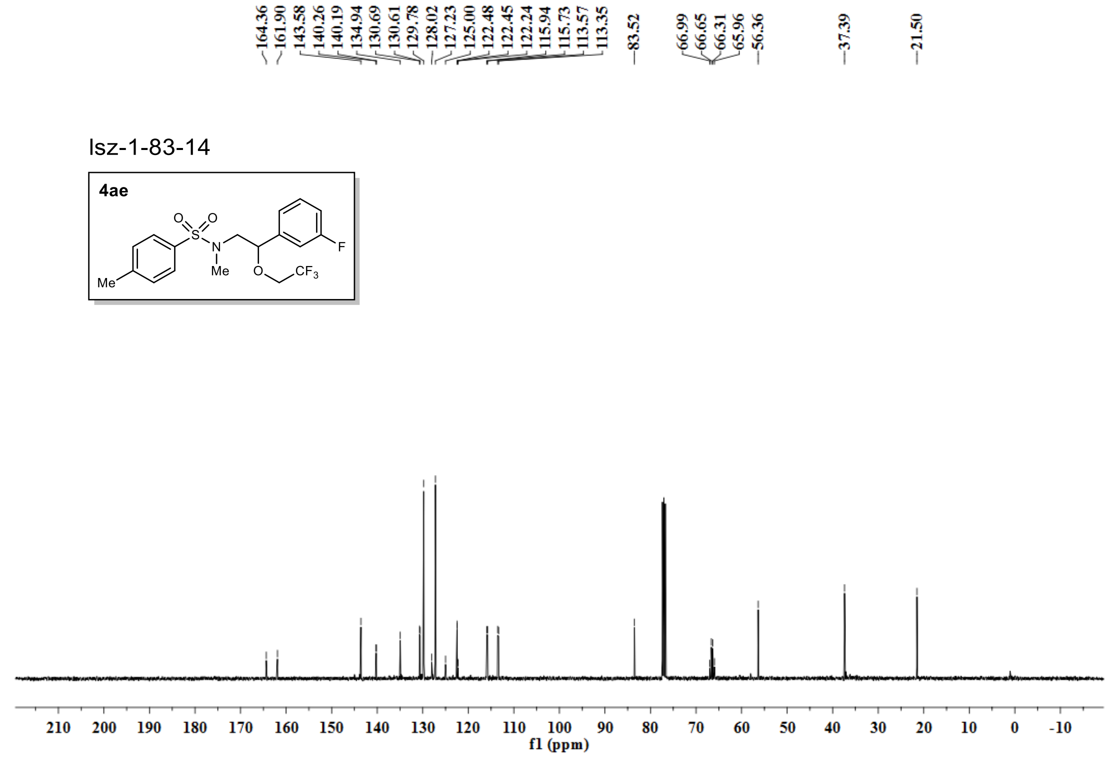

^{19}F NMR spectrum of 4ae (377 MHz, $CDCl_3$):

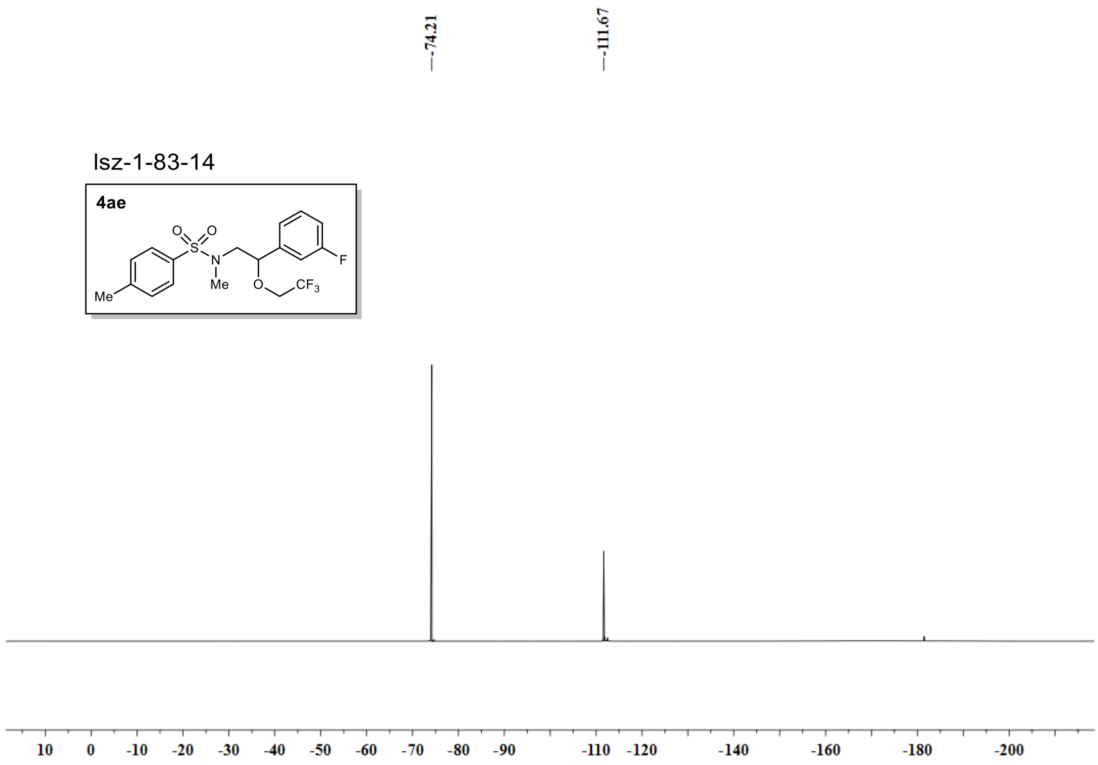

^{1}H NMR spectrum of 4af (400 MHz, $CDCl_3$):

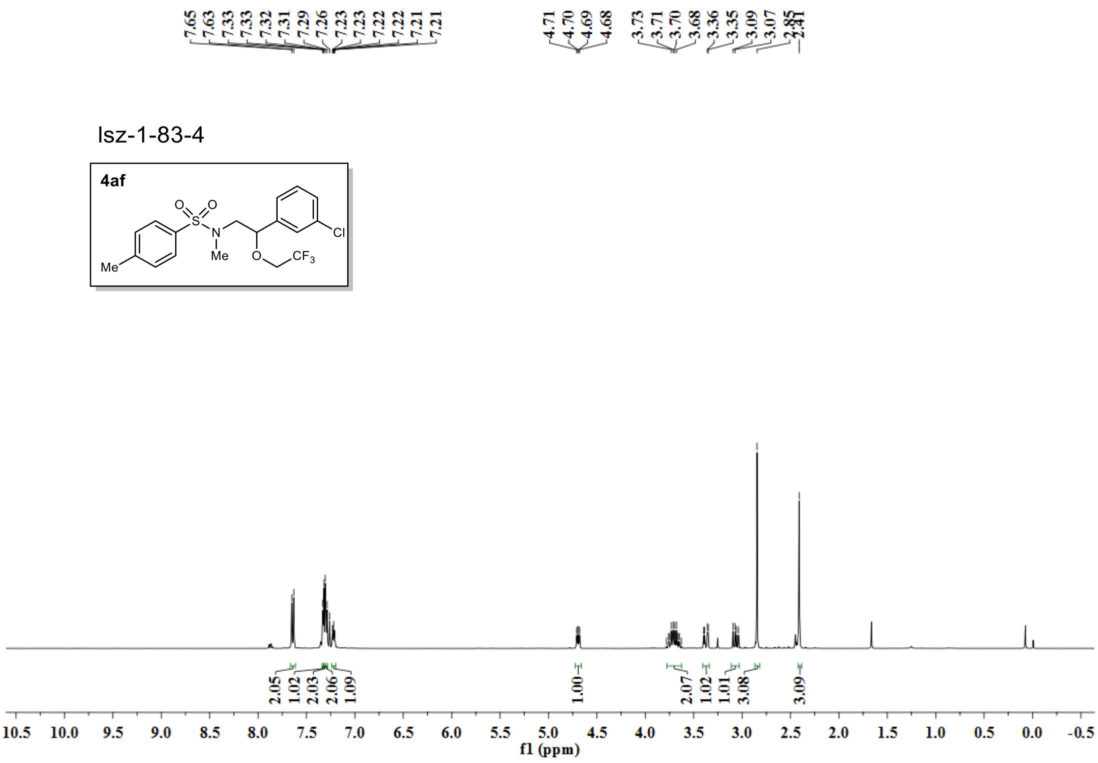

^{13}C NMR spectrum of 4af (101 MHz, $CDCl_3$):

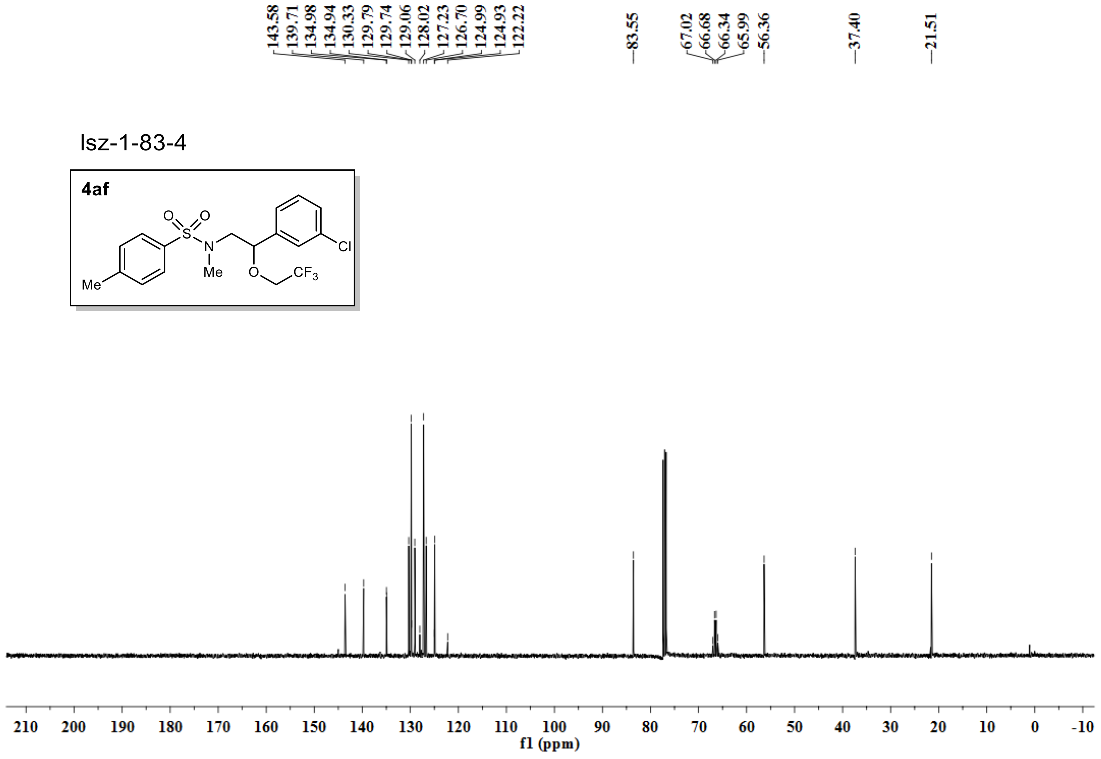

^{19}F NMR spectrum of 4af (377 MHz, $CDCl_3$):

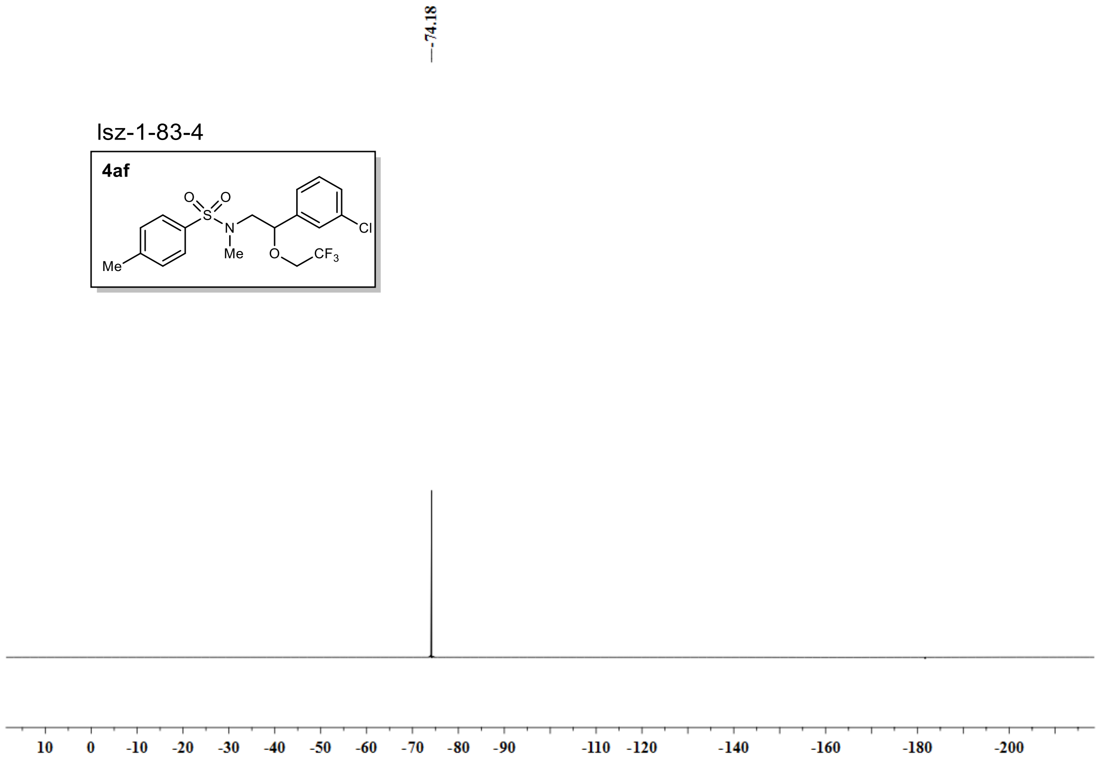

^{1}H NMR spectrum of 4ag (400 MHz, $CDCl_3$):

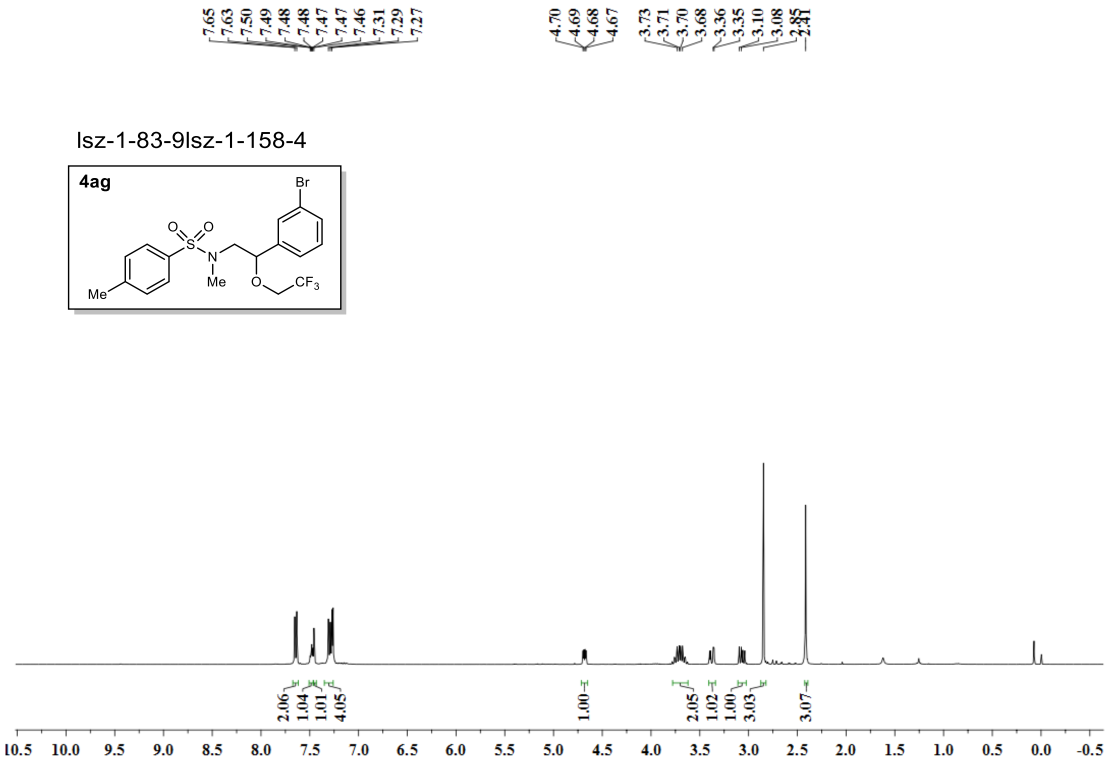

^{13}C NMR spectrum of 4ag (101 MHz, $CDCl_3$):

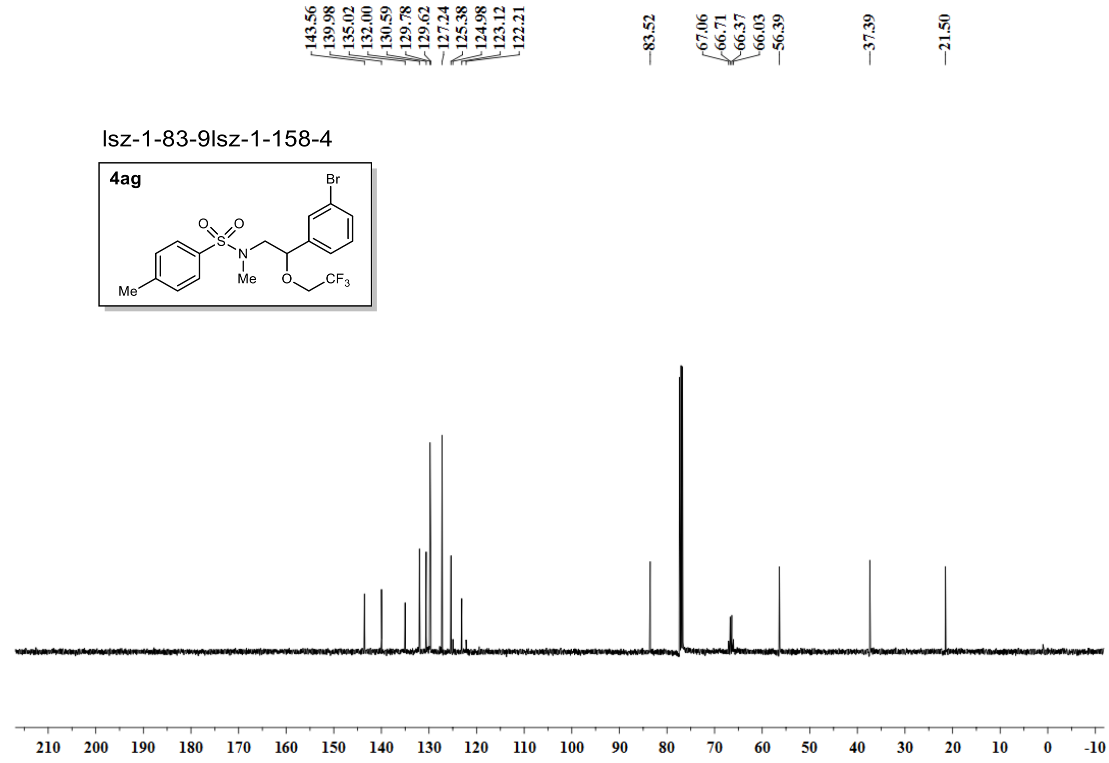

^{19}F NMR spectrum of 4ag (377 MHz, $CDCl_3$):

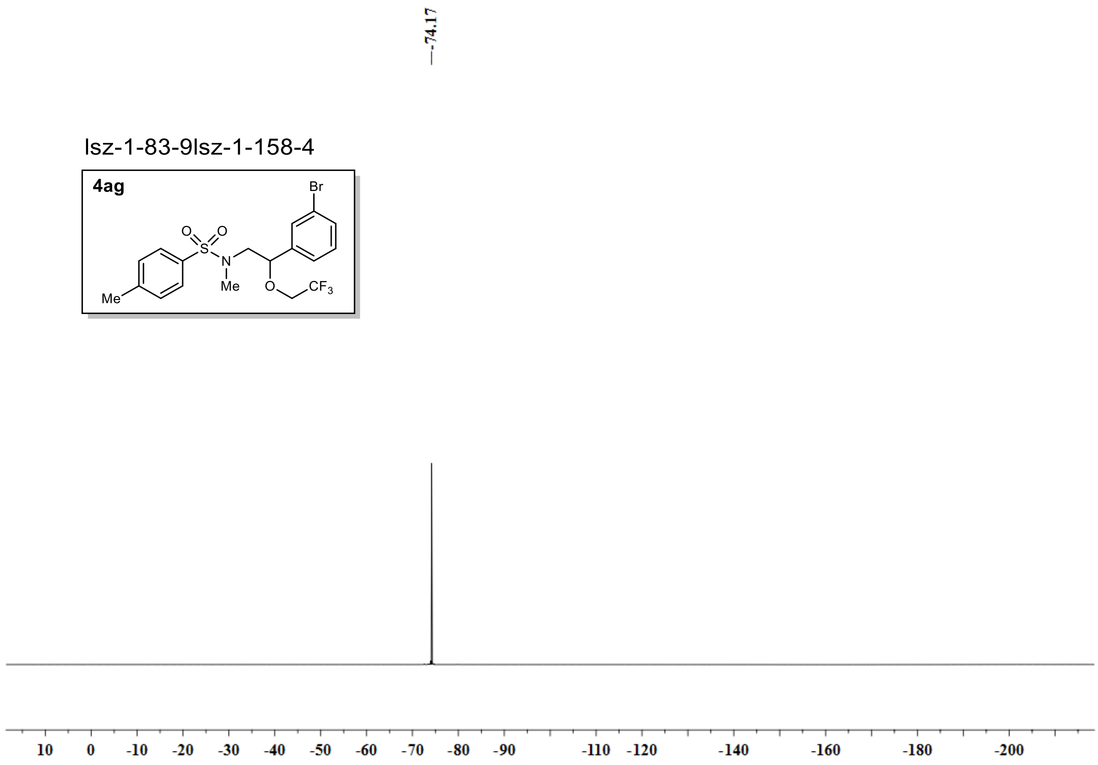

^{1}H NMR spectrum of 4ah (400 MHz, $CDCl_3$):

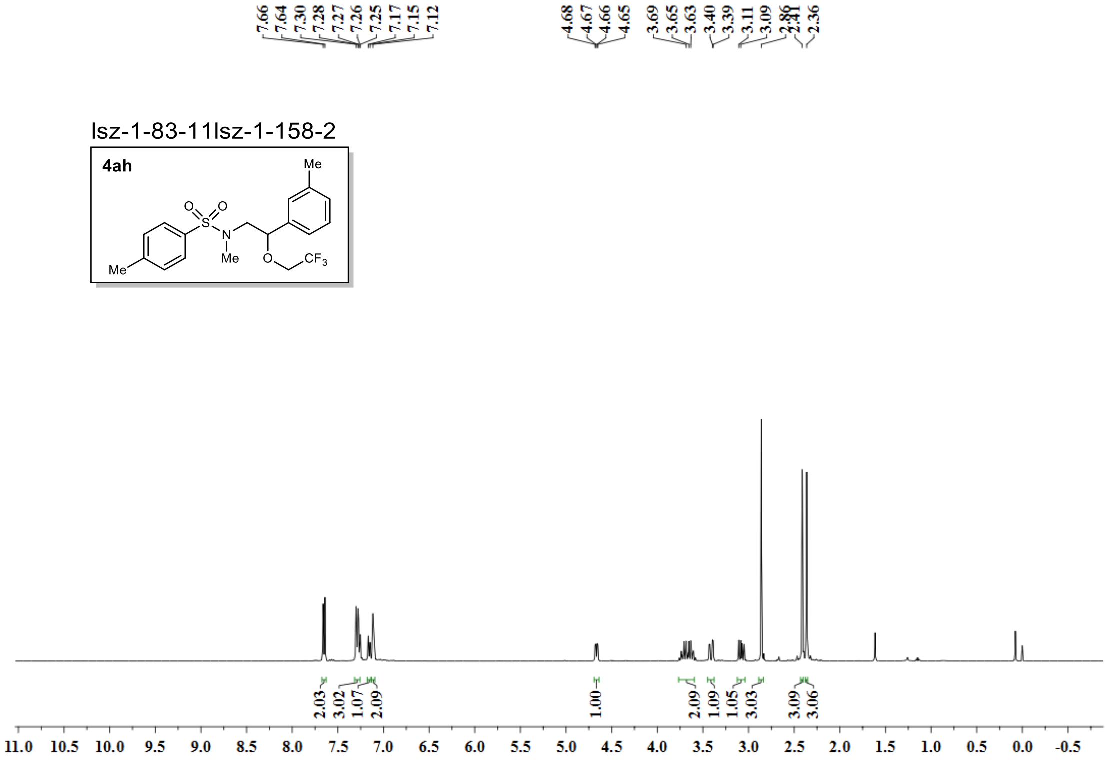

^{13}C NMR spectrum of 4ah (101 MHz, $CDCl_3$):

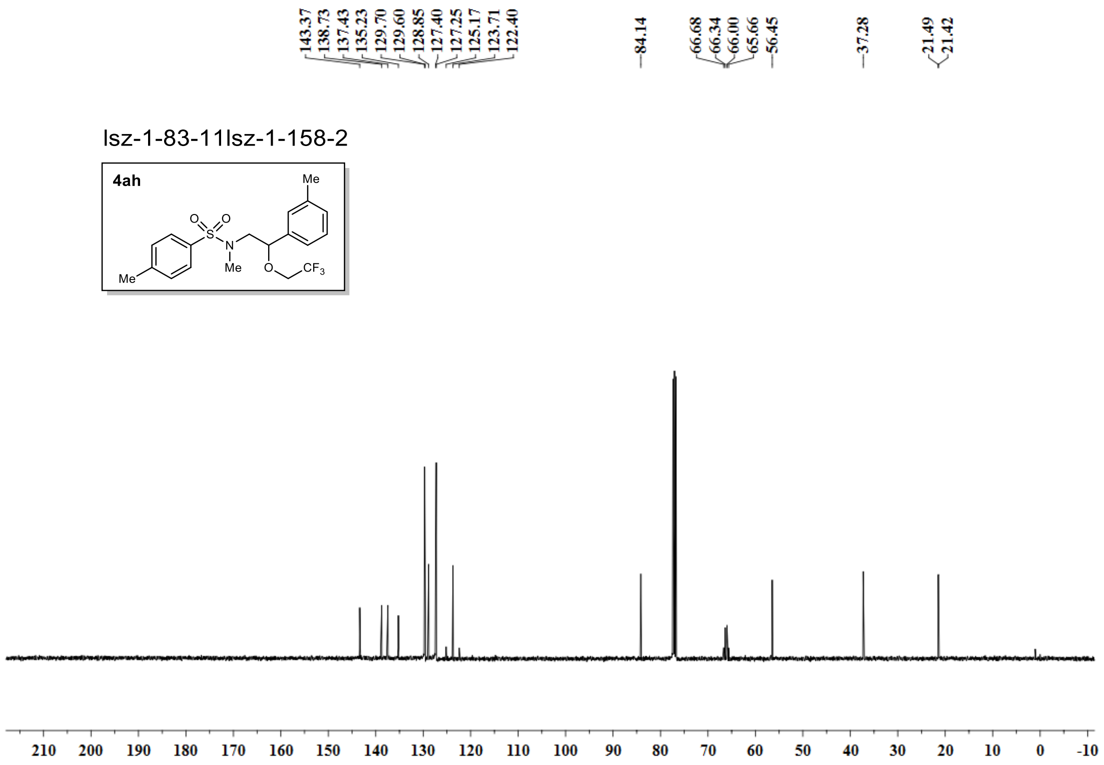

^{19}F NMR spectrum of 4ah (377 MHz, $CDCl_3$):

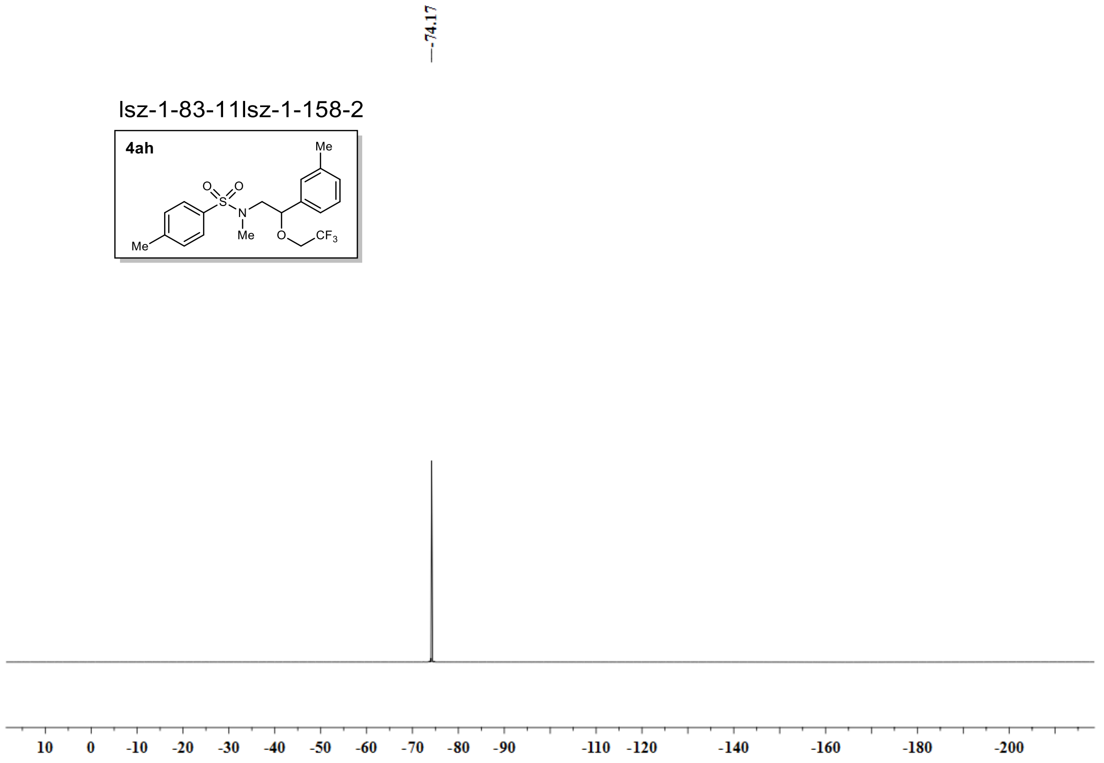

^{1}H NMR spectrum of 4ai (400 MHz, $CDCl_3$):

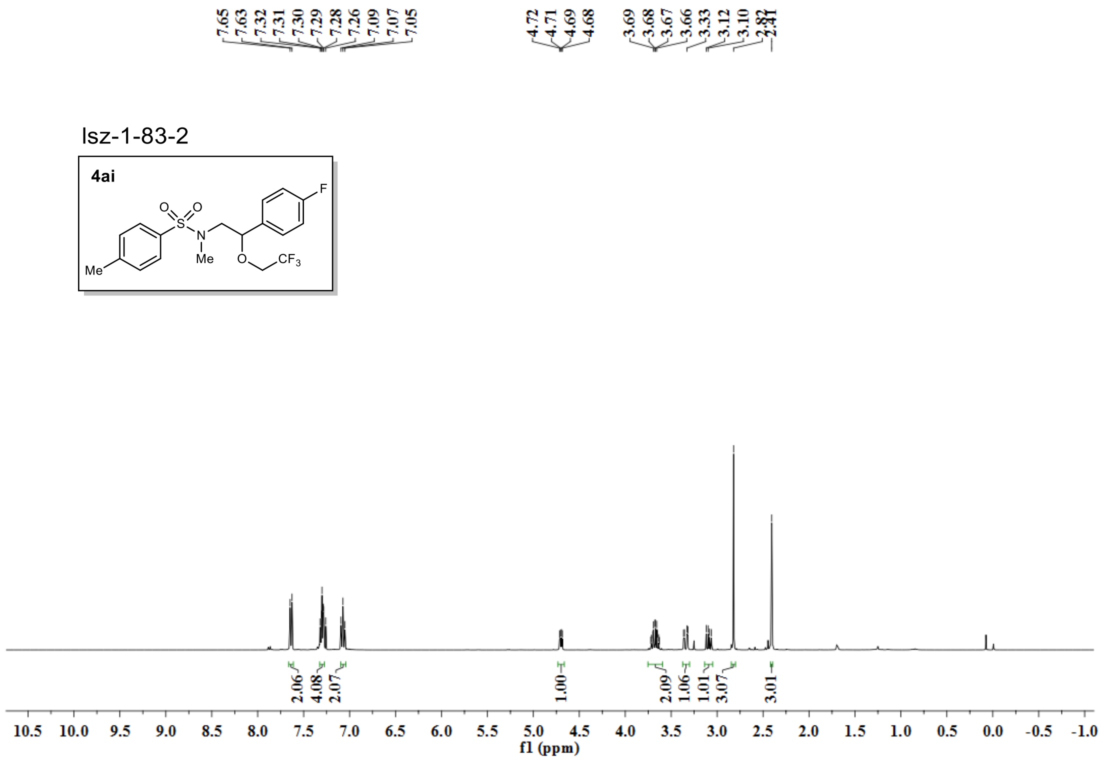

^{13}C NMR spectrum of 4ai (101 MHz, $CDCl_3$):

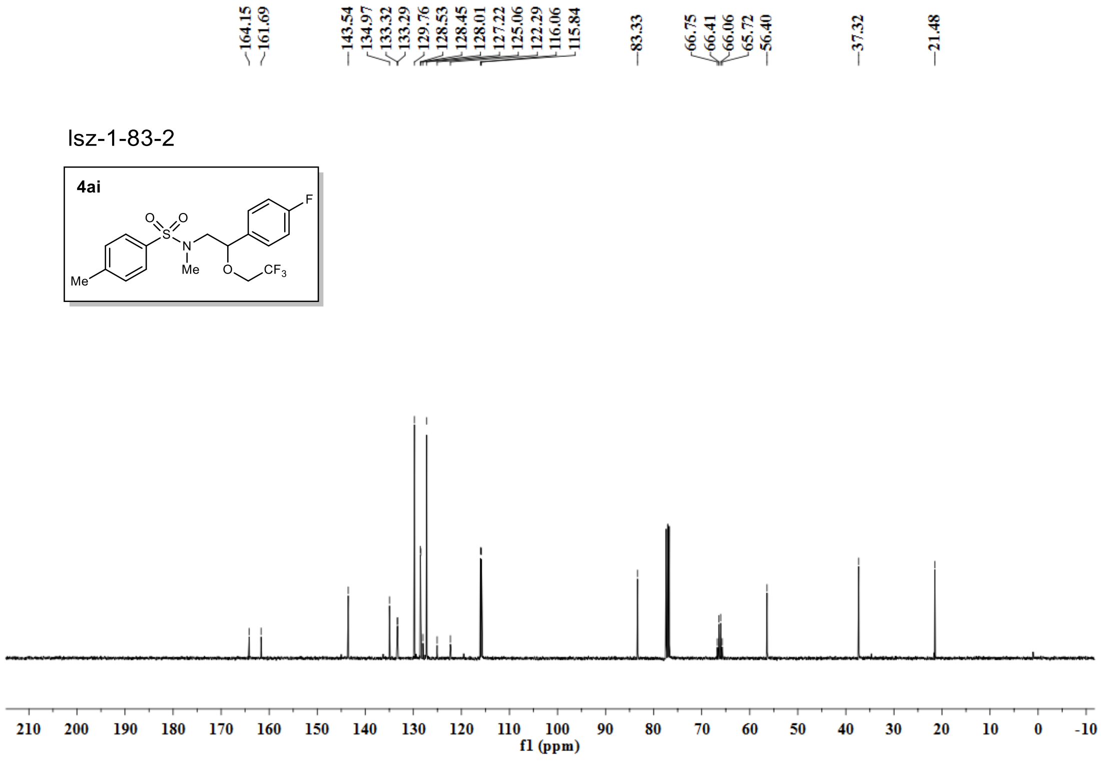

^{19}F NMR spectrum of 4ai (377 MHz, $CDCl_3$):

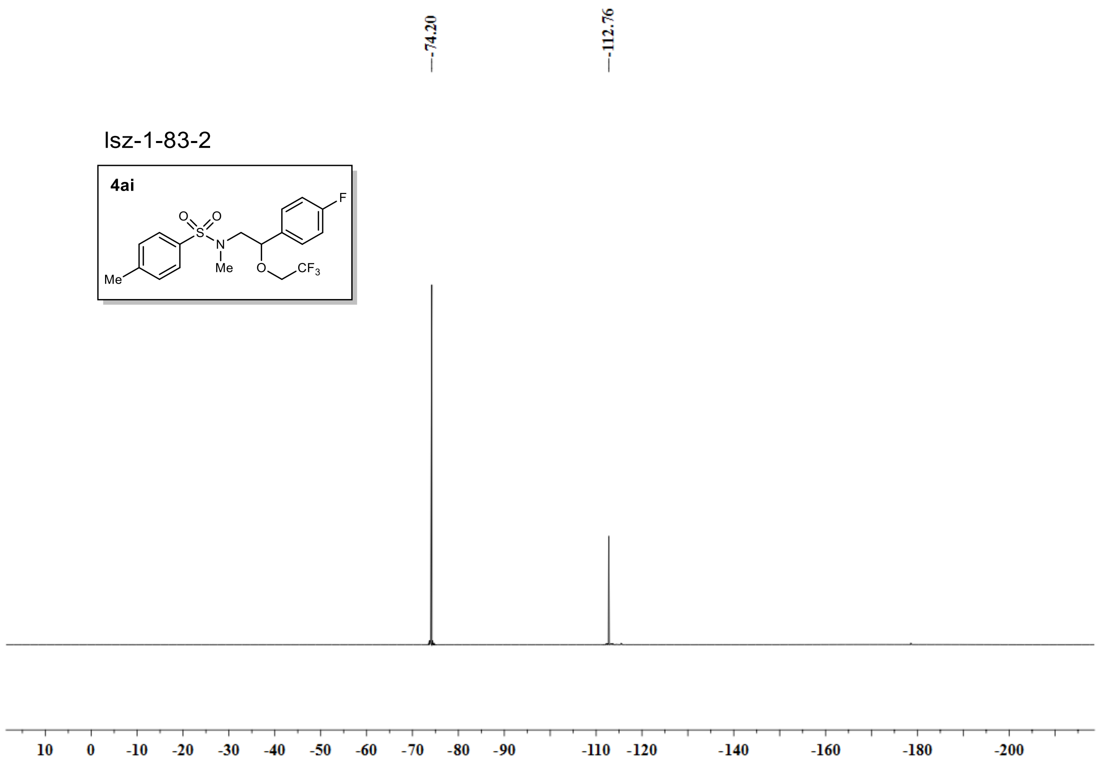

^{1}H NMR spectrum of 4aj (400 MHz, $CDCl_3$):

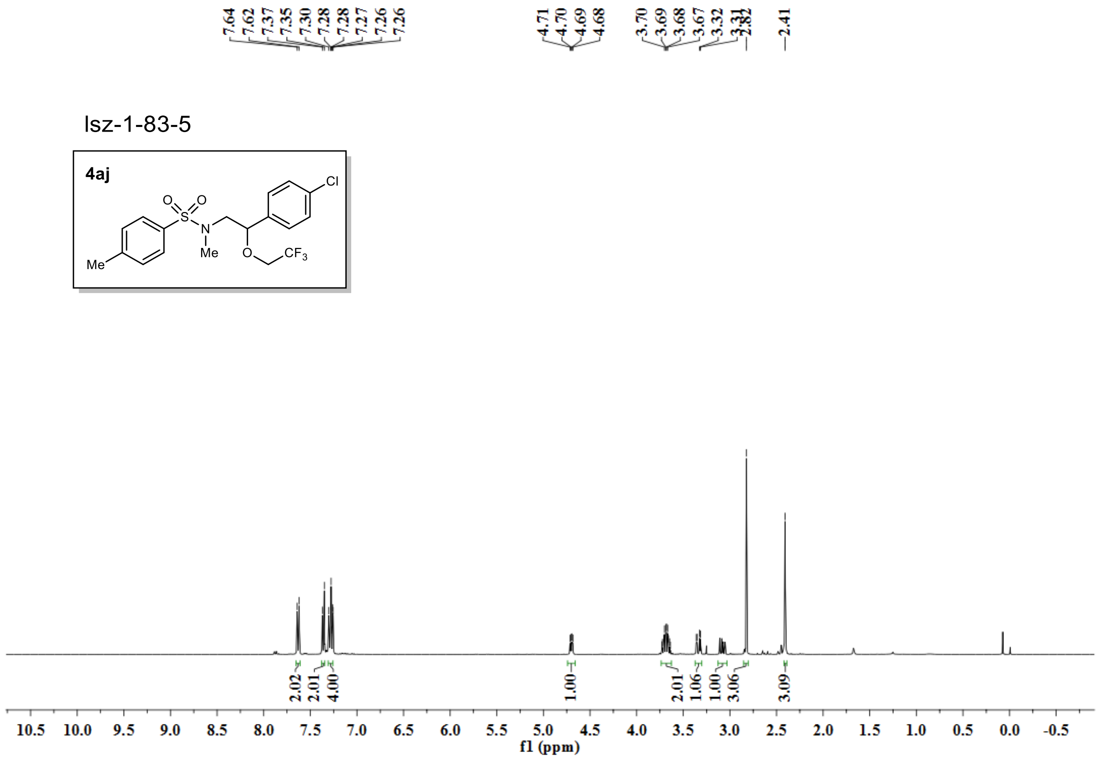

^{13}C NMR spectrum of 4aj (101 MHz, $CDCl_3$):

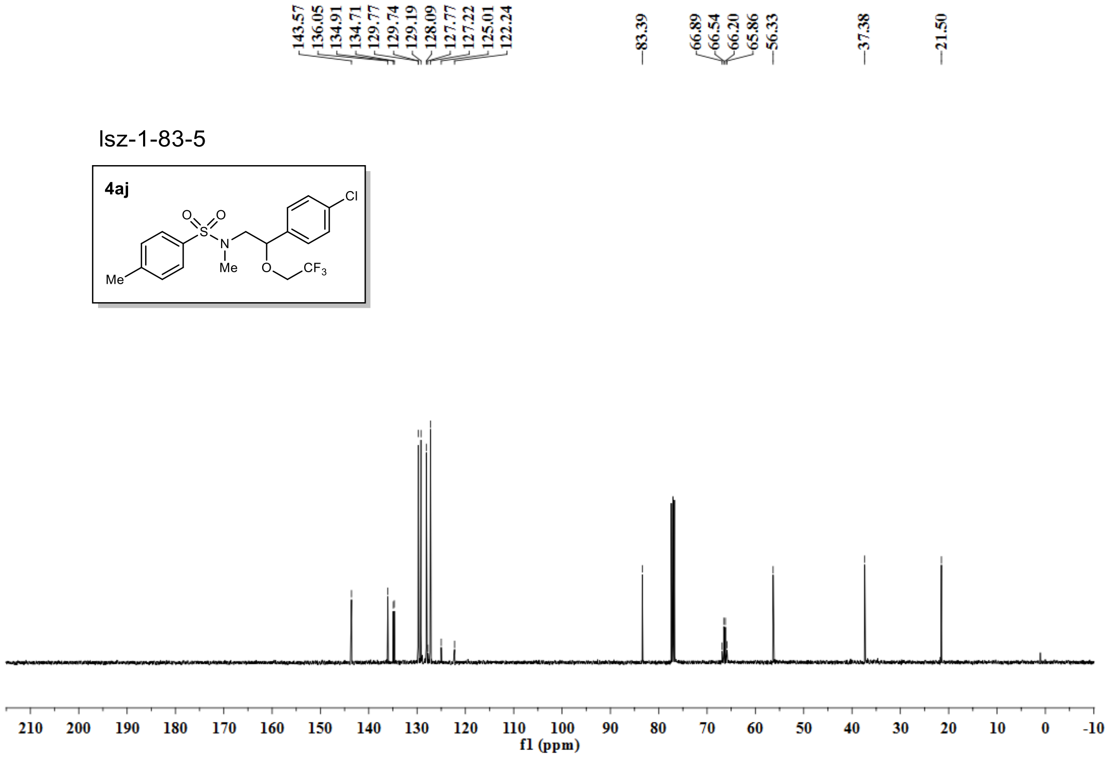

^{19}F NMR spectrum of 4aj (377 MHz, $CDCl_3$):

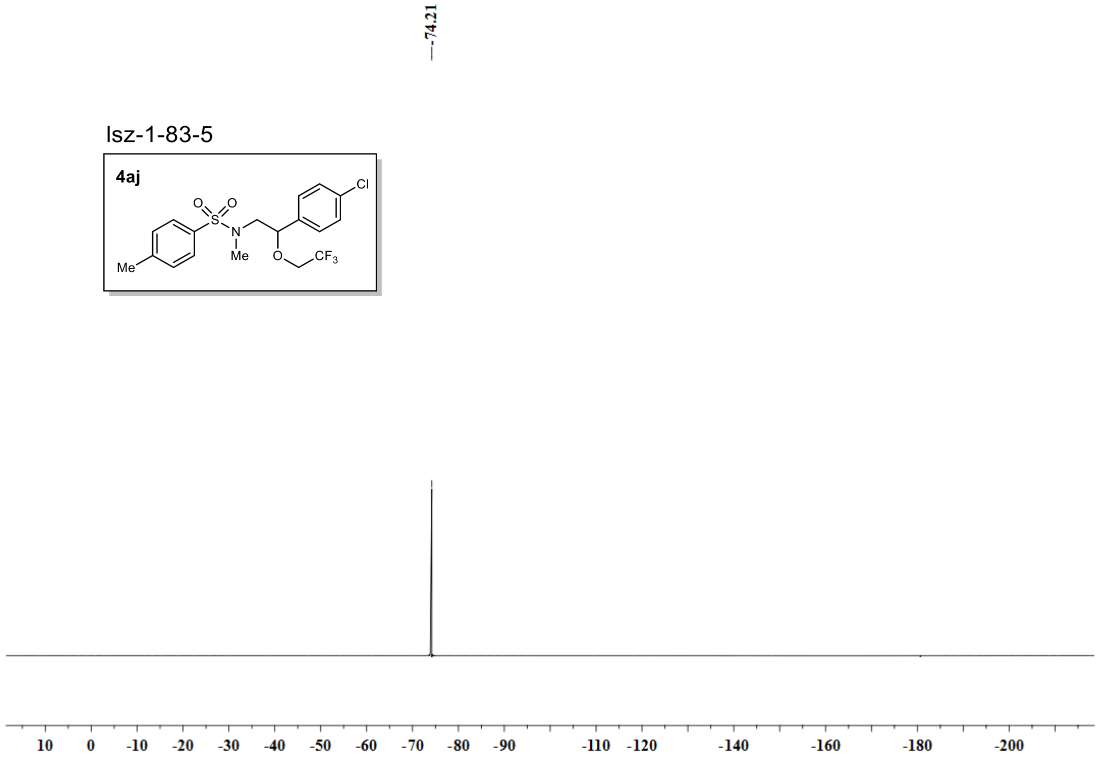

^{1}H NMR spectrum of 4ak (400 MHz, $CDCl_3$):

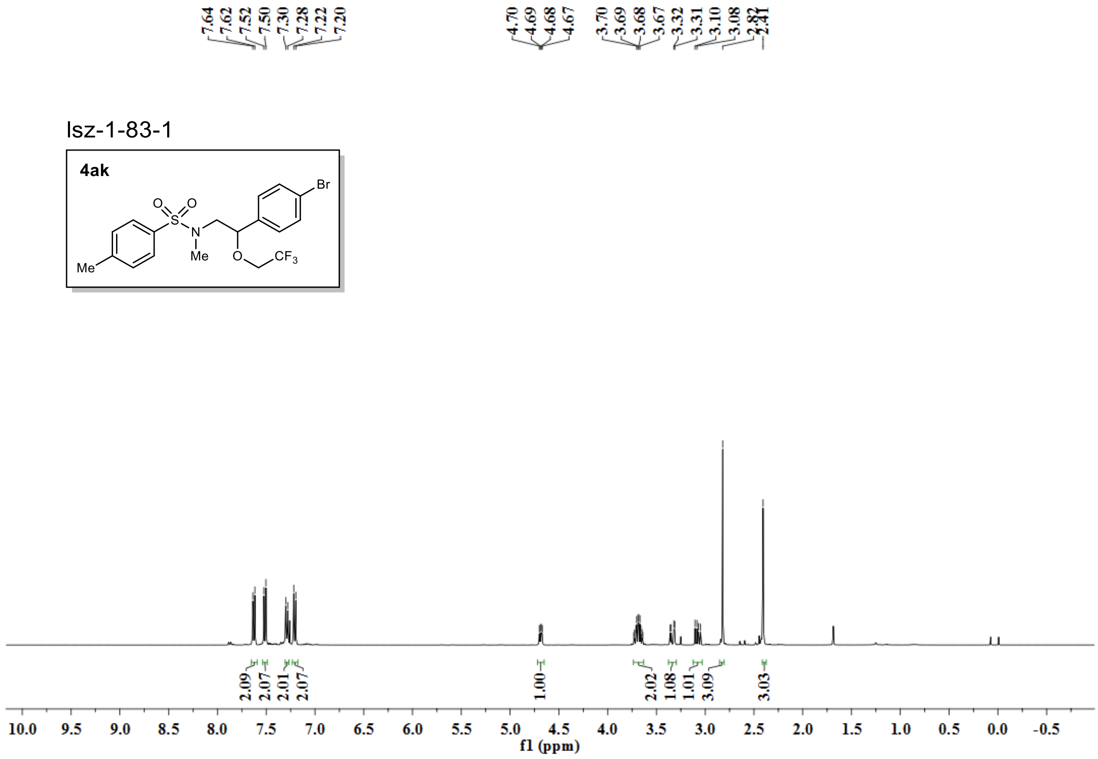

^{13}C NMR spectrum of 4ak (101 MHz, $CDCl_3$):

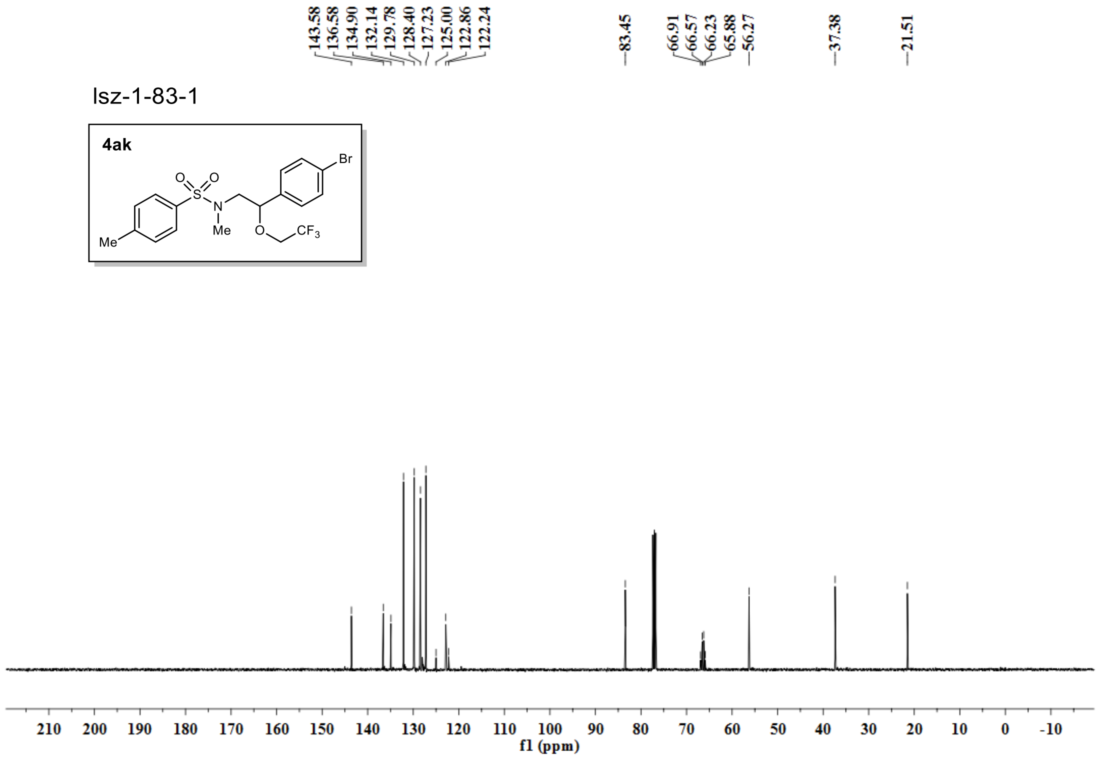

^{19}F NMR spectrum of 4ak (377 MHz, $CDCl_3$):

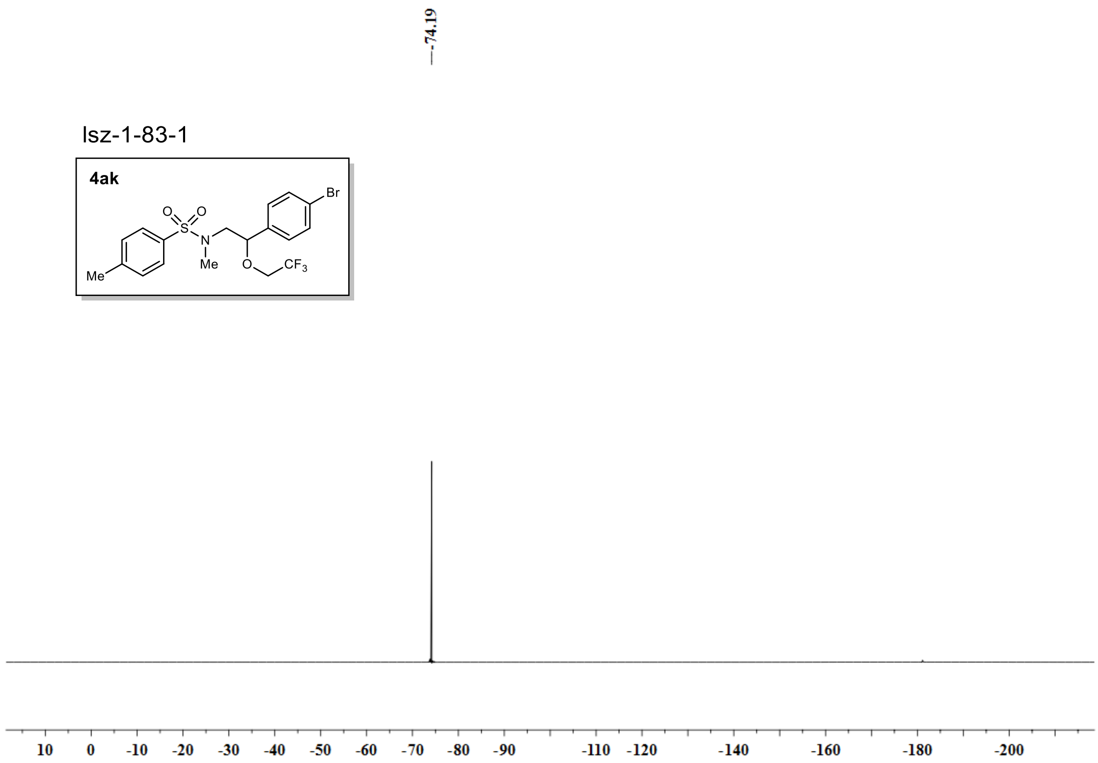

^{1}H NMR spectrum of 4al (400 MHz, $CDCl_3$):

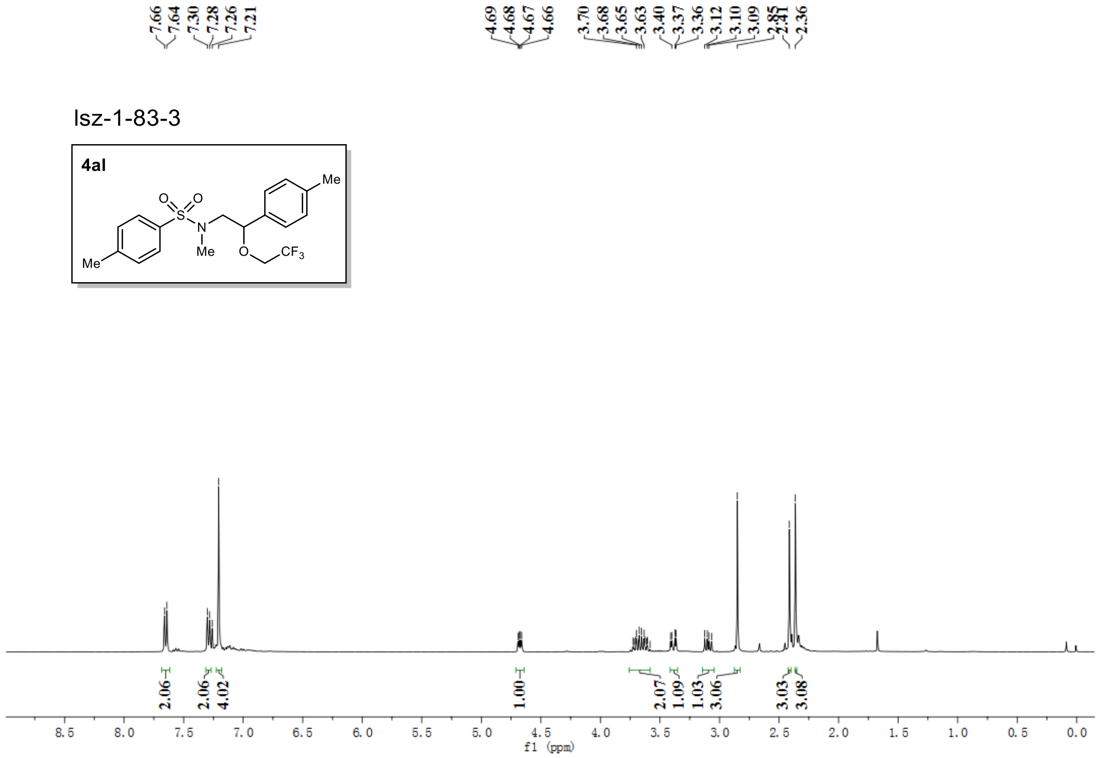

^{13}C NMR spectrum of 4al (101 MHz, $CDCl_3$):

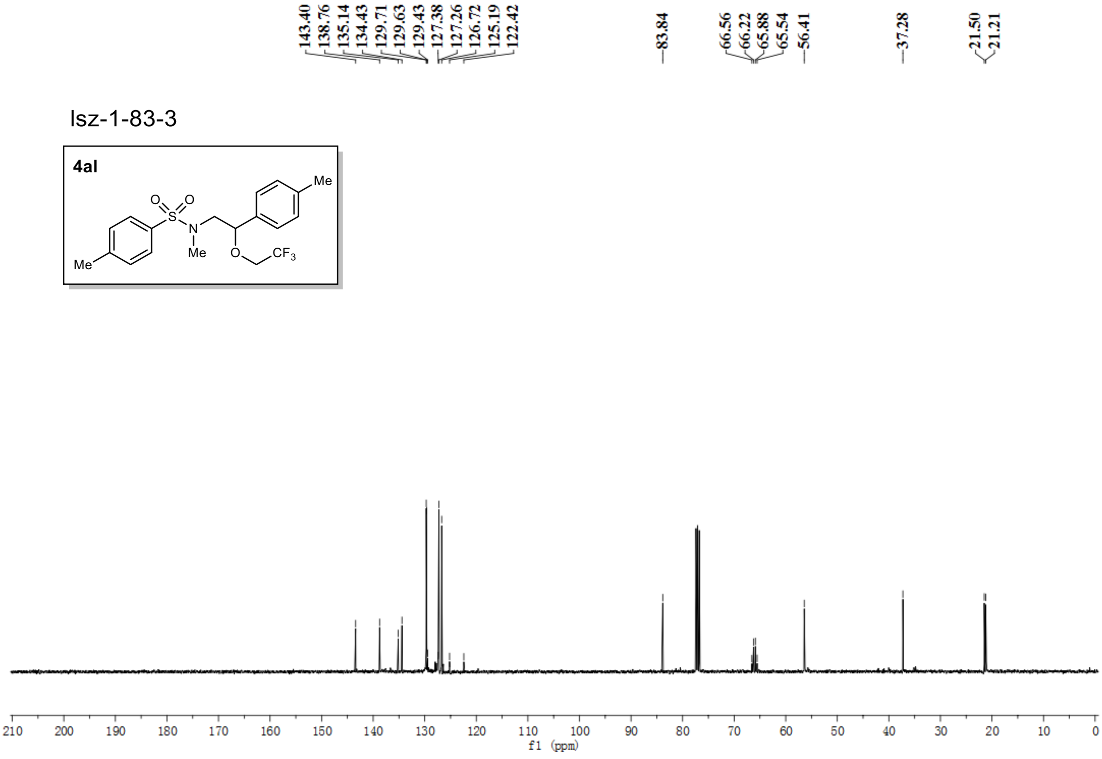

^{19}F NMR spectrum of 4al (377 MHz, $CDCl_3$):

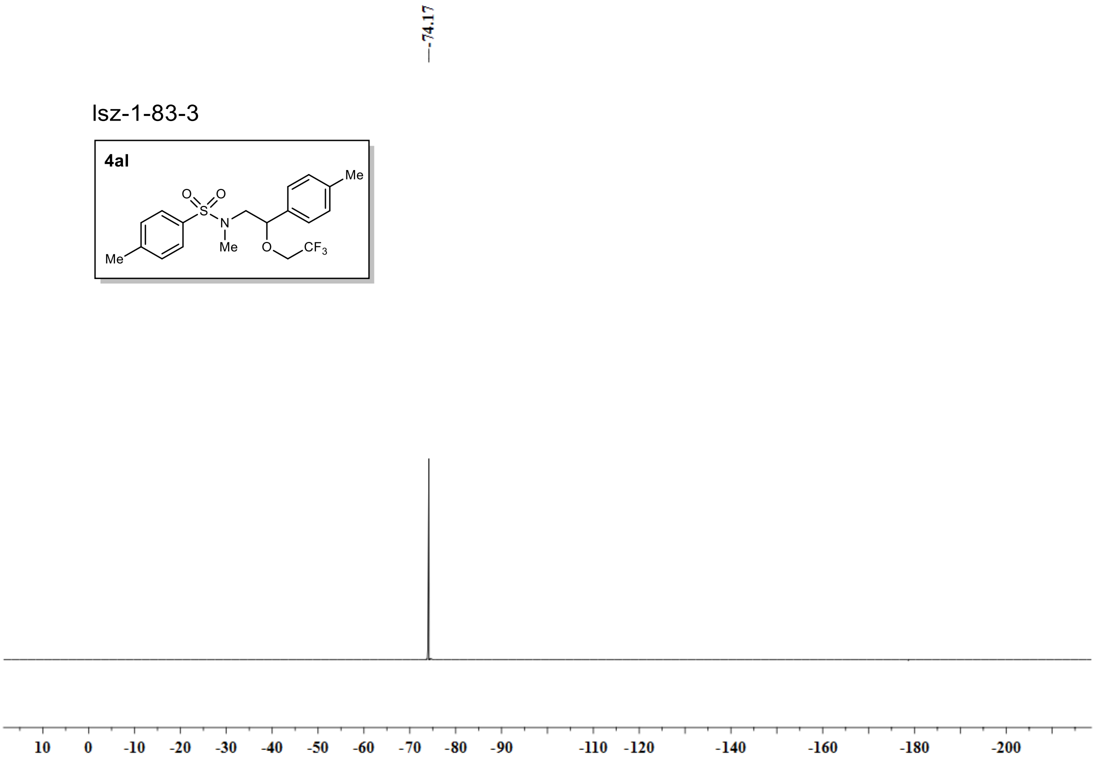

^{1}H NMR spectrum of 4am (400 MHz, CDCl$_3$):

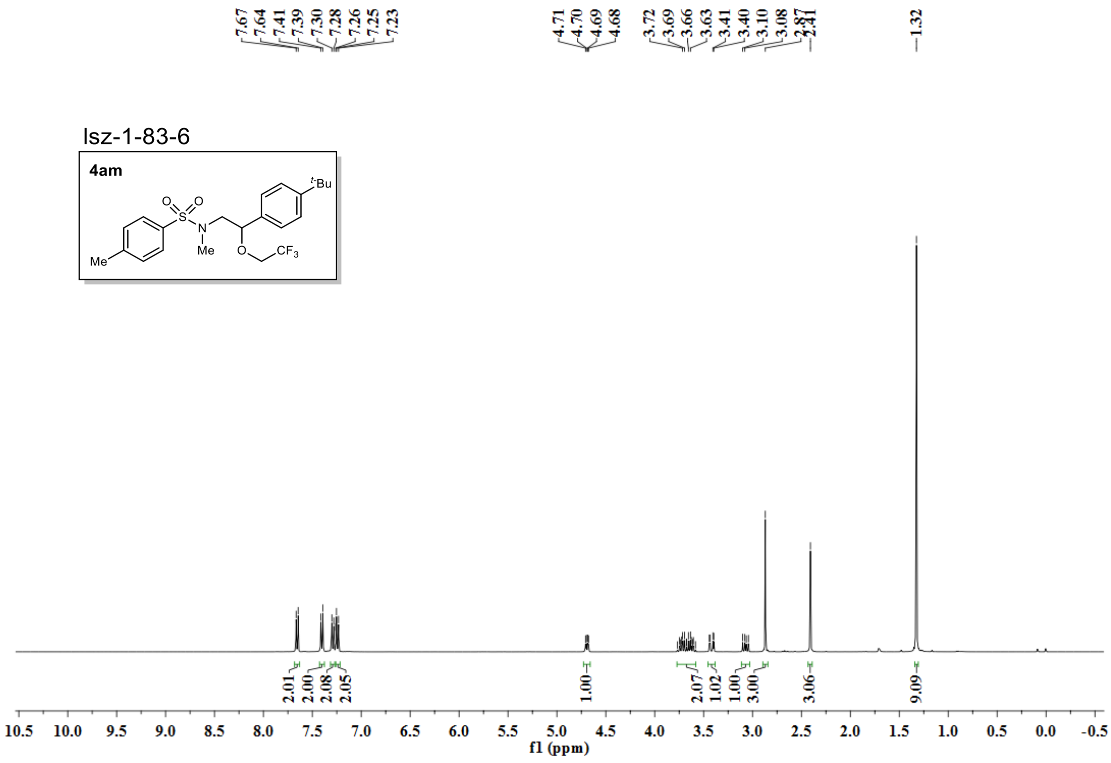

^{13}C NMR spectrum of 4am (101 MHz, CDCl$_3$):

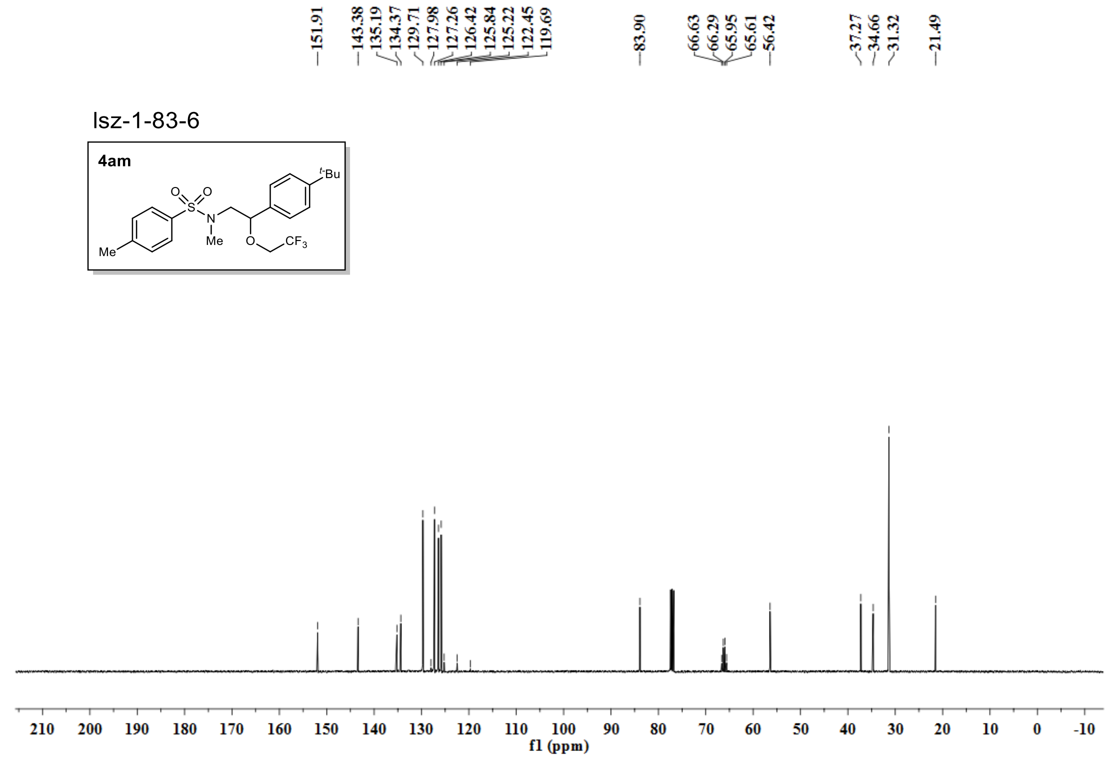

^{19}F NMR spectrum of 4am (377 MHz, $CDCl_3$):

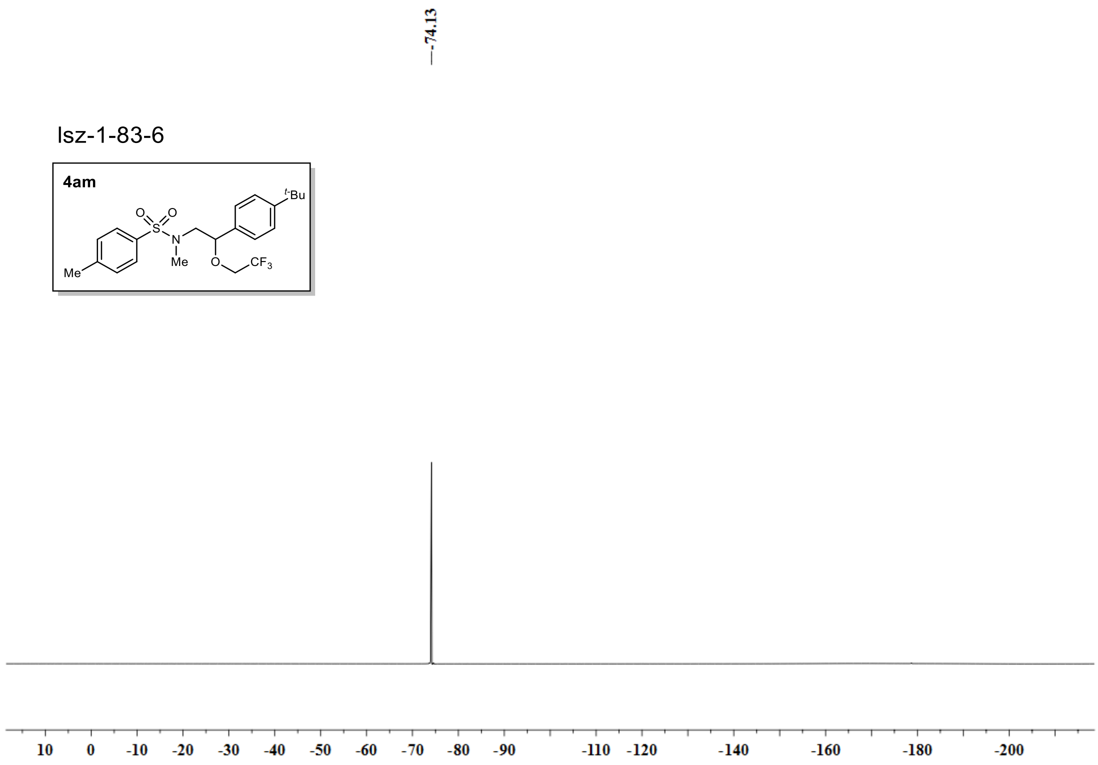

^{1}H NMR spectrum of 4an (400 MHz, $CDCl_3$):

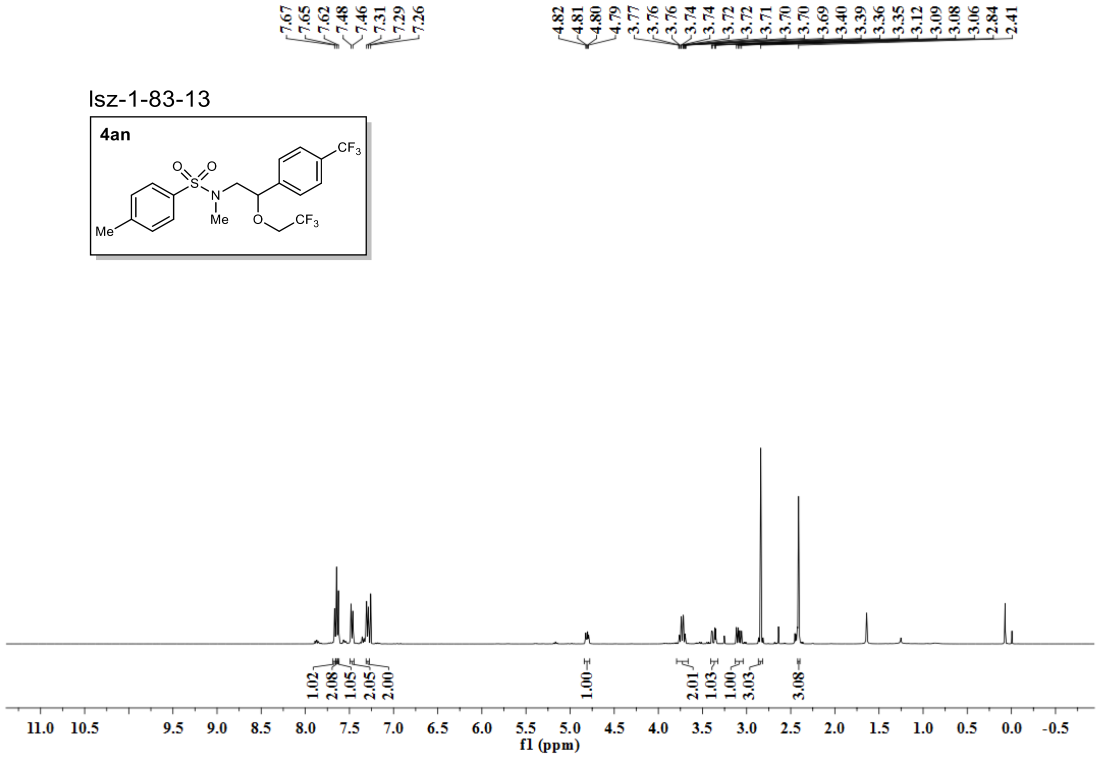

^{13}C NMR spectrum of 4an (101 MHz, $CDCl_3$):

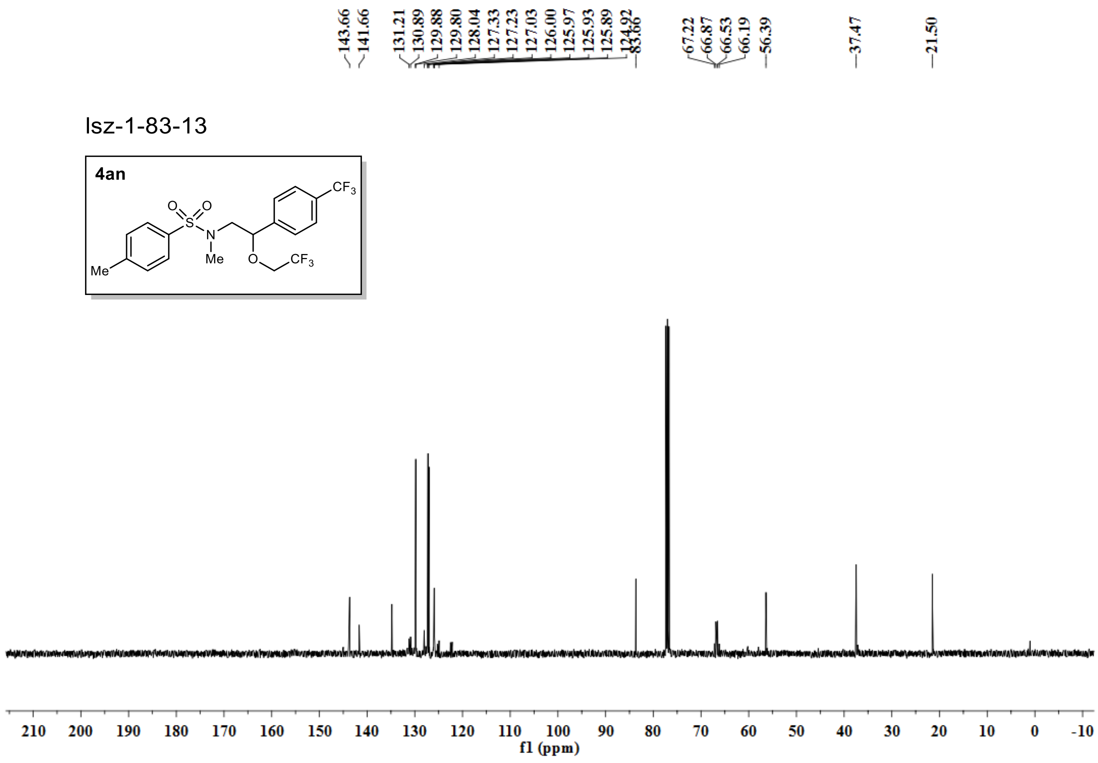

^{19}F NMR spectrum of 4an (377 MHz, $CDCl_3$):

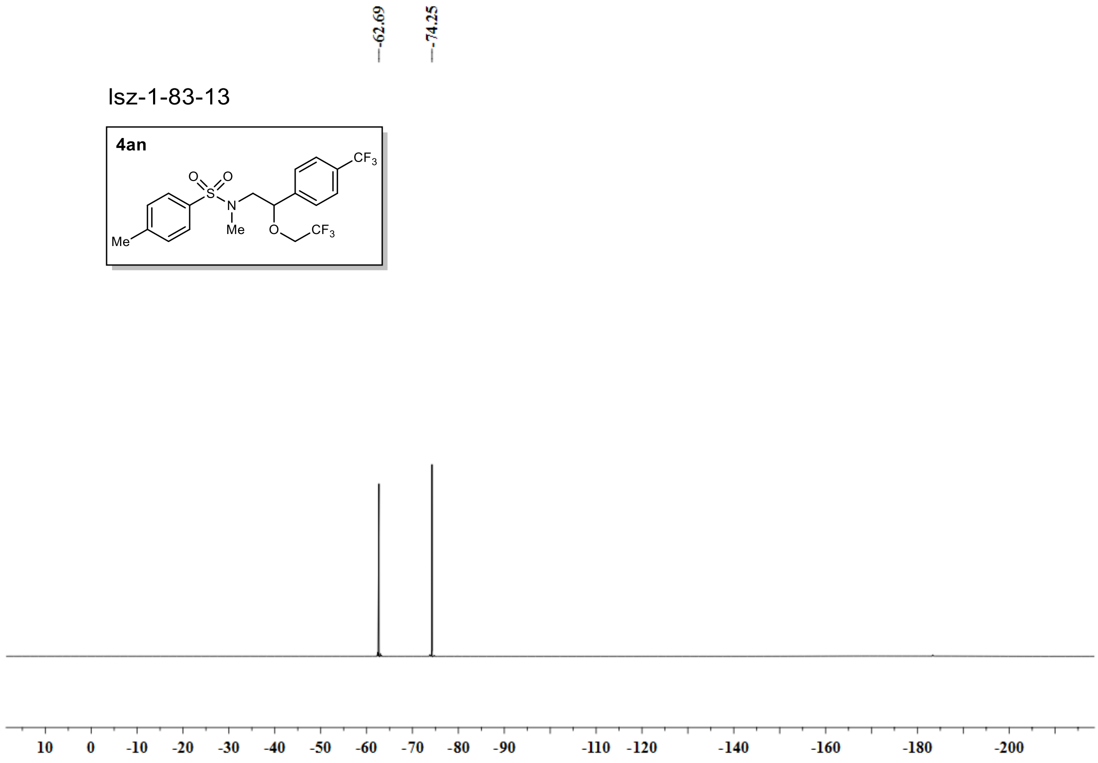

^{1}H NMR spectrum of 4ao (400 MHz, $CDCl_3$):

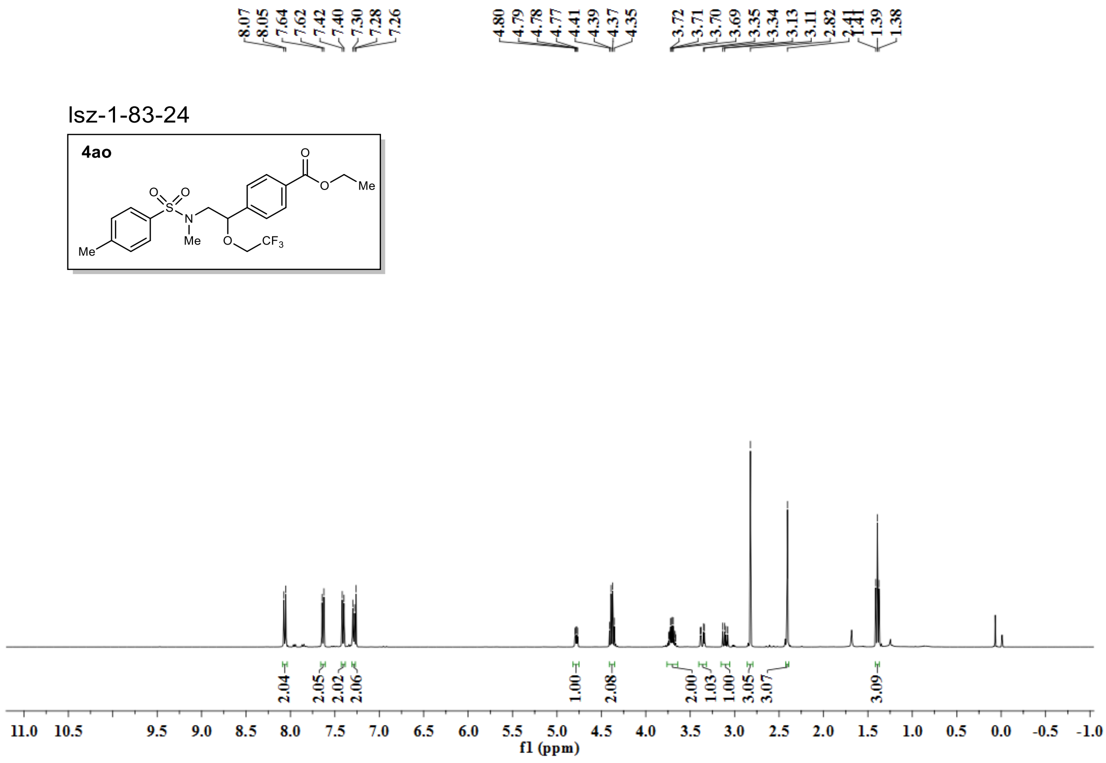

^{13}C NMR spectrum of 4ao (101 MHz, $CDCl_3$):

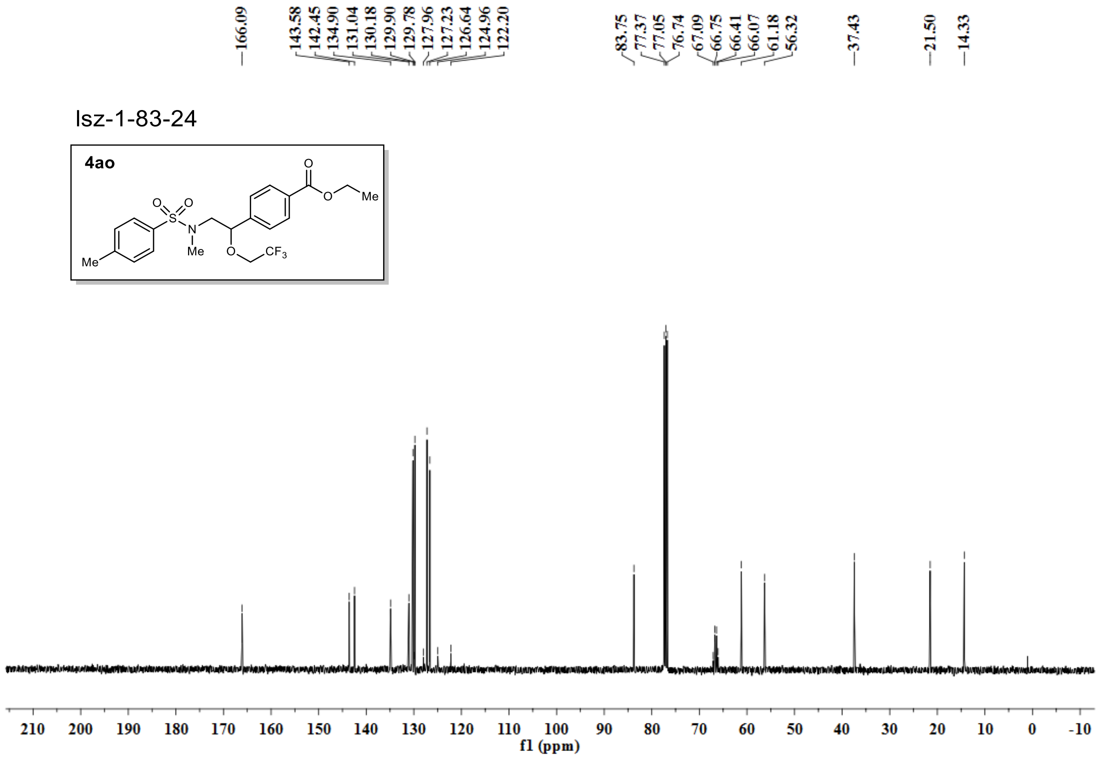

^{19}F NMR spectrum of 4ao (377 MHz, $CDCl_3$):

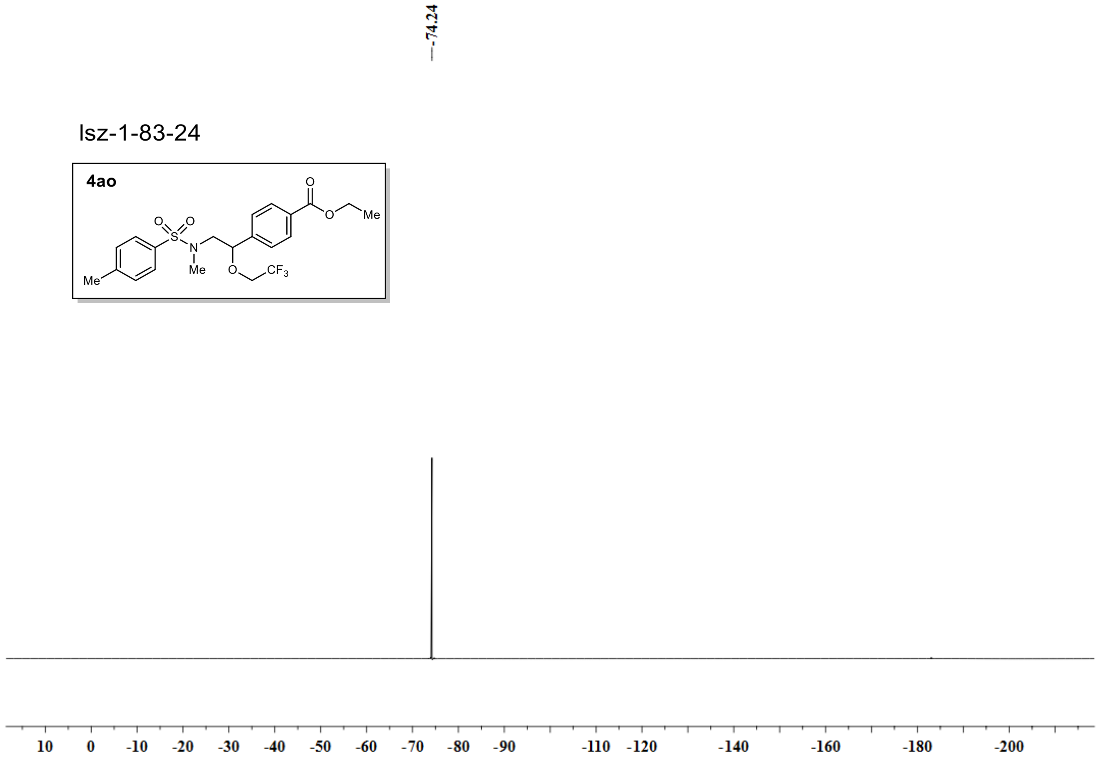

^{1}H NMR spectrum of 4ap (400 MHz, $CDCl_3$):

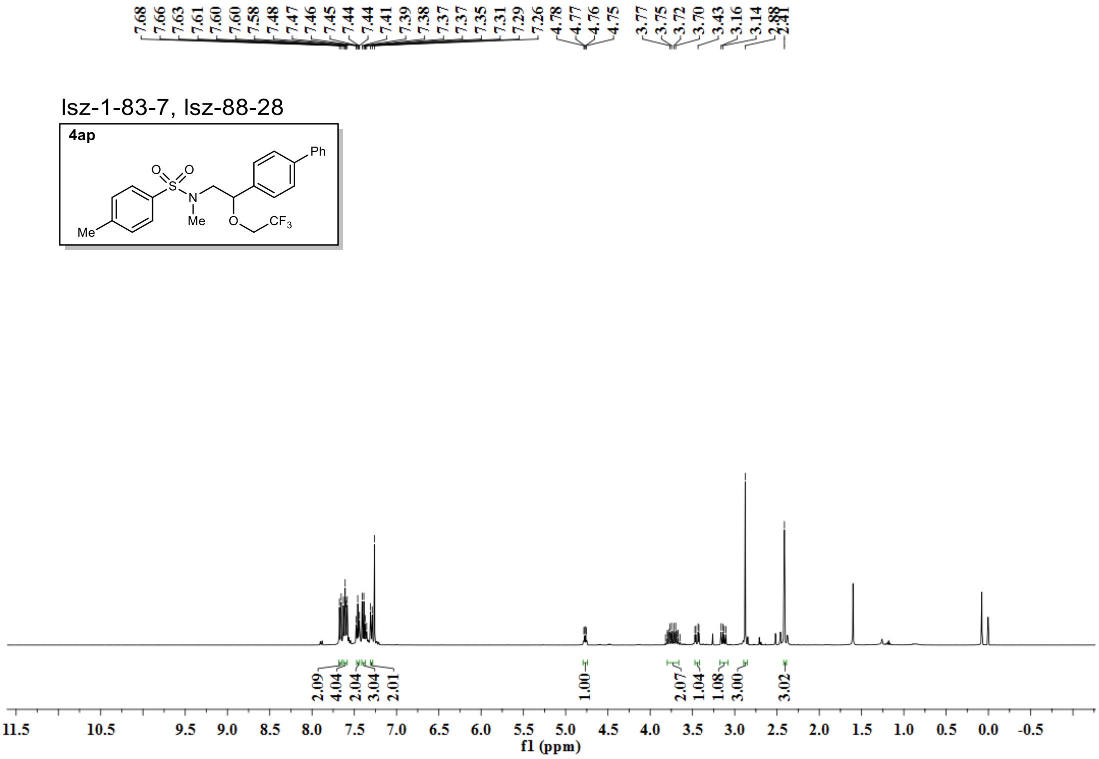

^{13}C NMR spectrum of 4ap (101 MHz, $CDCl_3$):

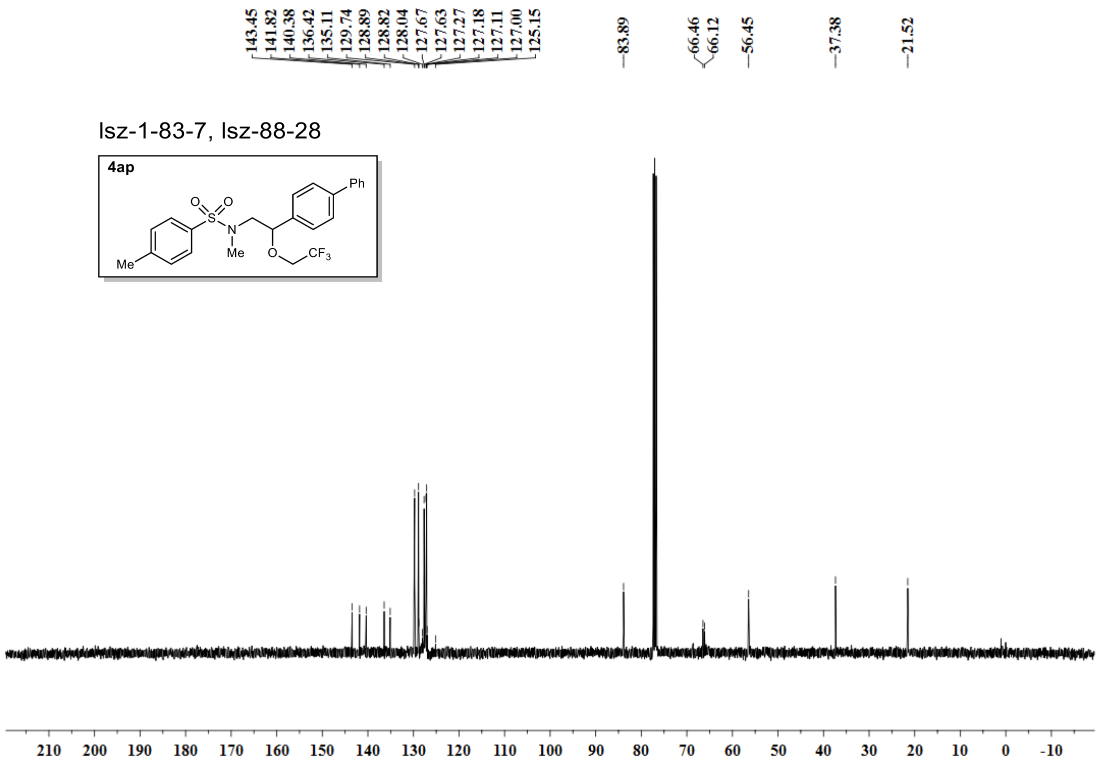

^{19}F NMR spectrum of 4ap (377 MHz, $CDCl_3$):

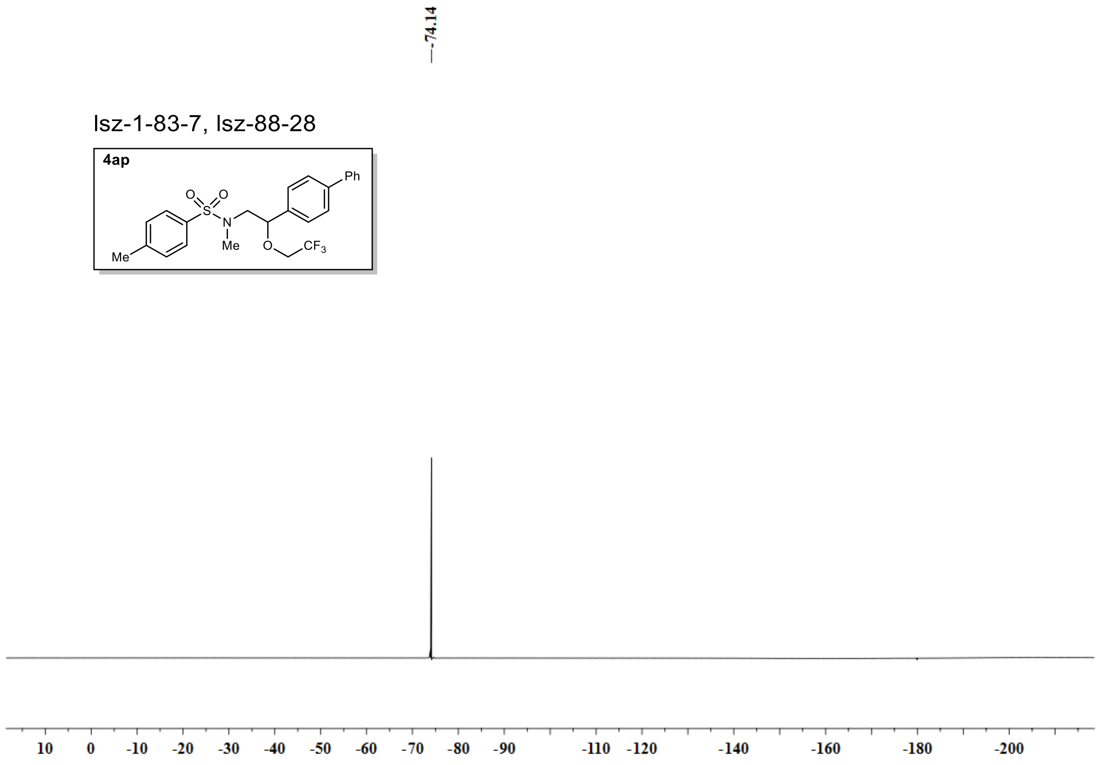

^{1}H NMR spectrum of 4aq (400 MHz, $CDCl_3$):

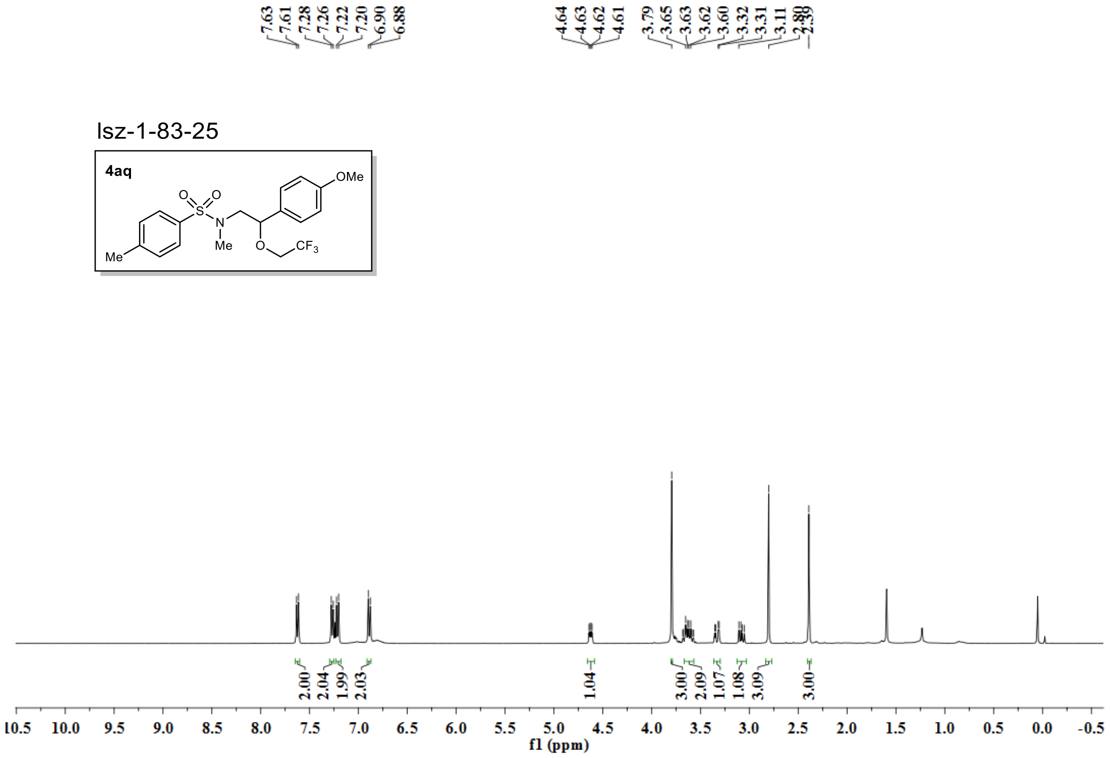

^{13}C NMR spectrum of 4aq (101 MHz, $CDCl_3$):

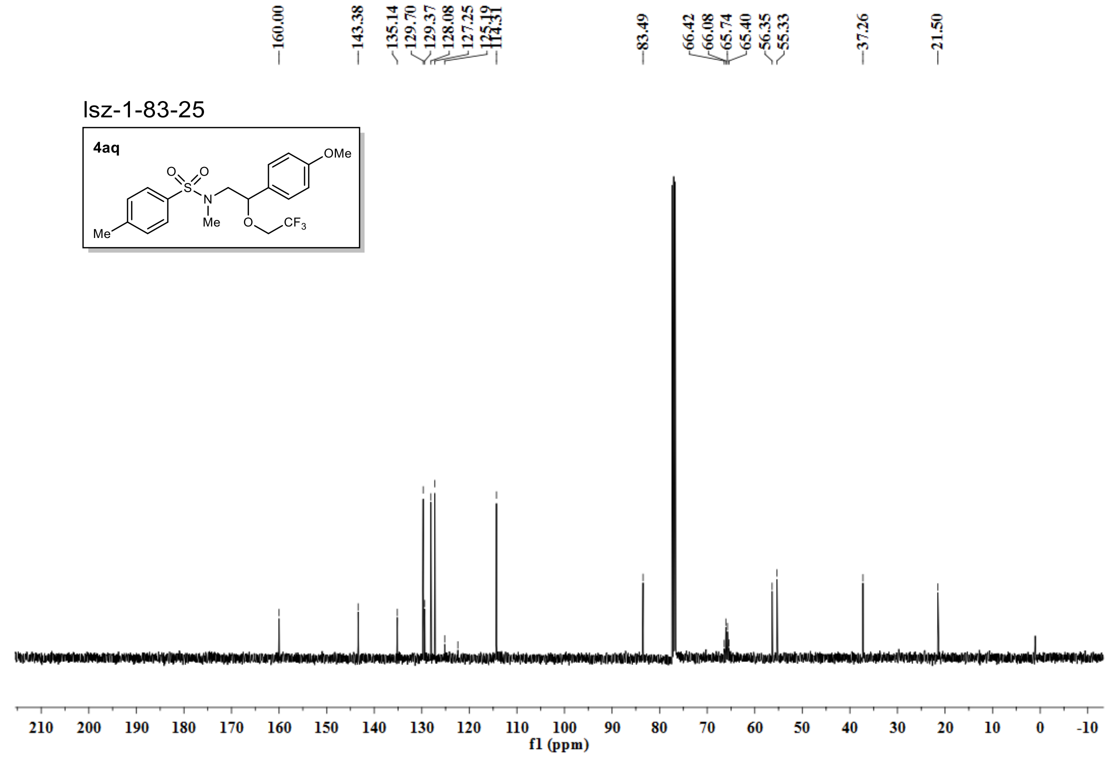

^{19}F NMR spectrum of 4aq (377 MHz, $CDCl_3$):

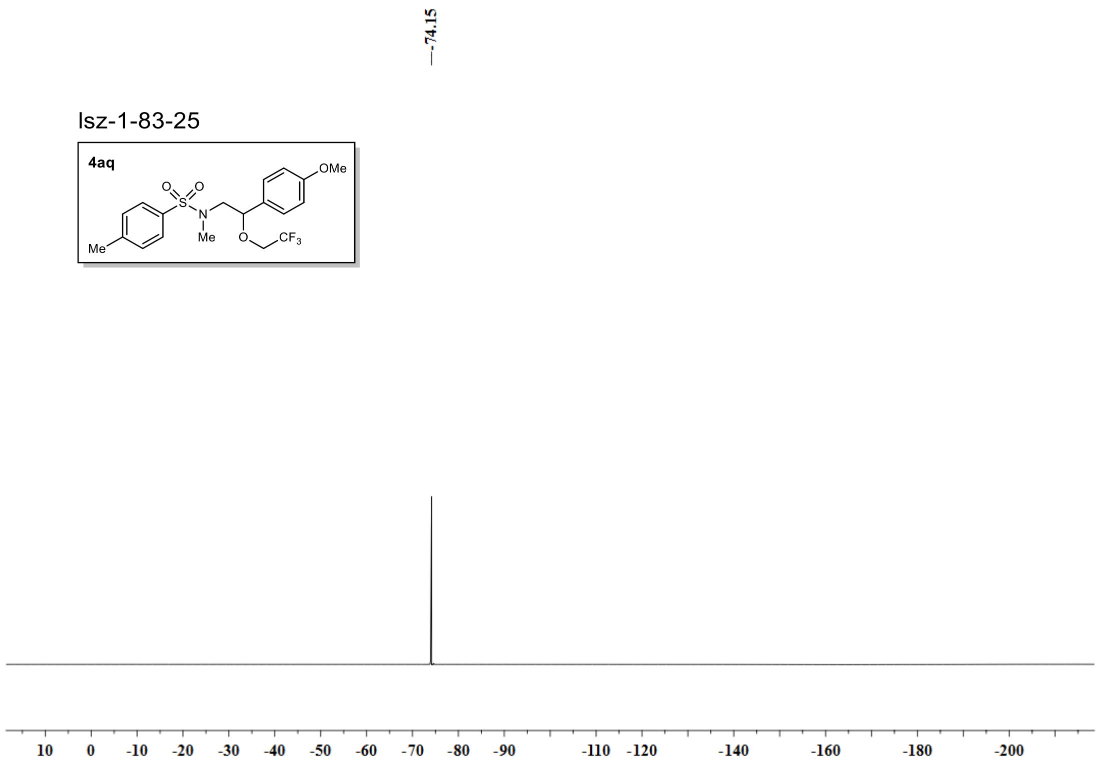

^{1}H NMR spectrum of 4ar (400 MHz, $CDCl_3$):

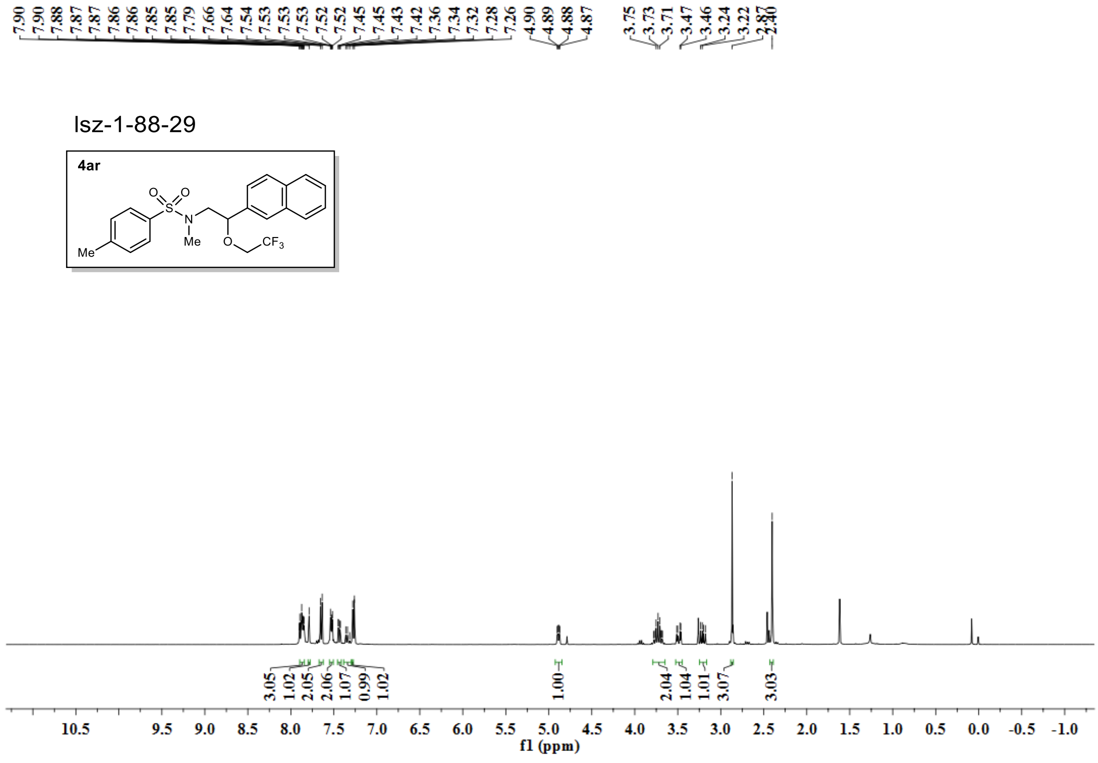

^{13}C NMR spectrum of 4ar (101 MHz, $CDCl_3$):

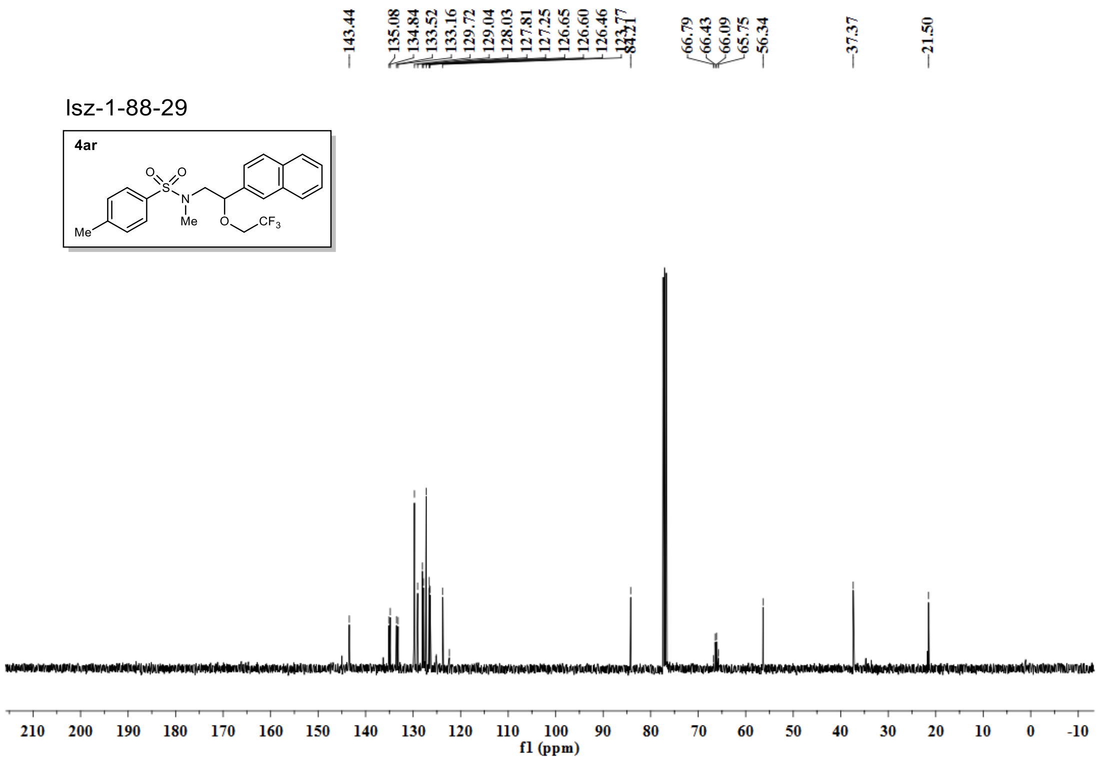

^{19}F NMR spectrum of 4ar (377 MHz, $CDCl_3$):

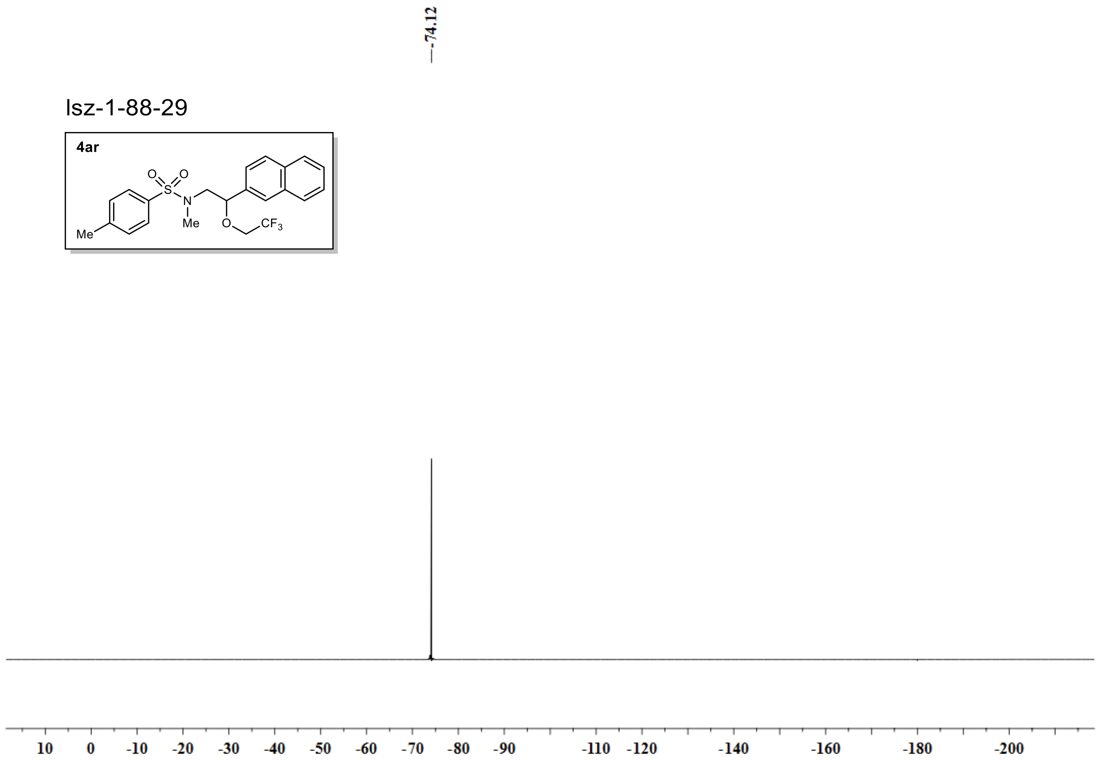

^{1}H NMR spectrum of 4as (400 MHz, $CDCl_3$):

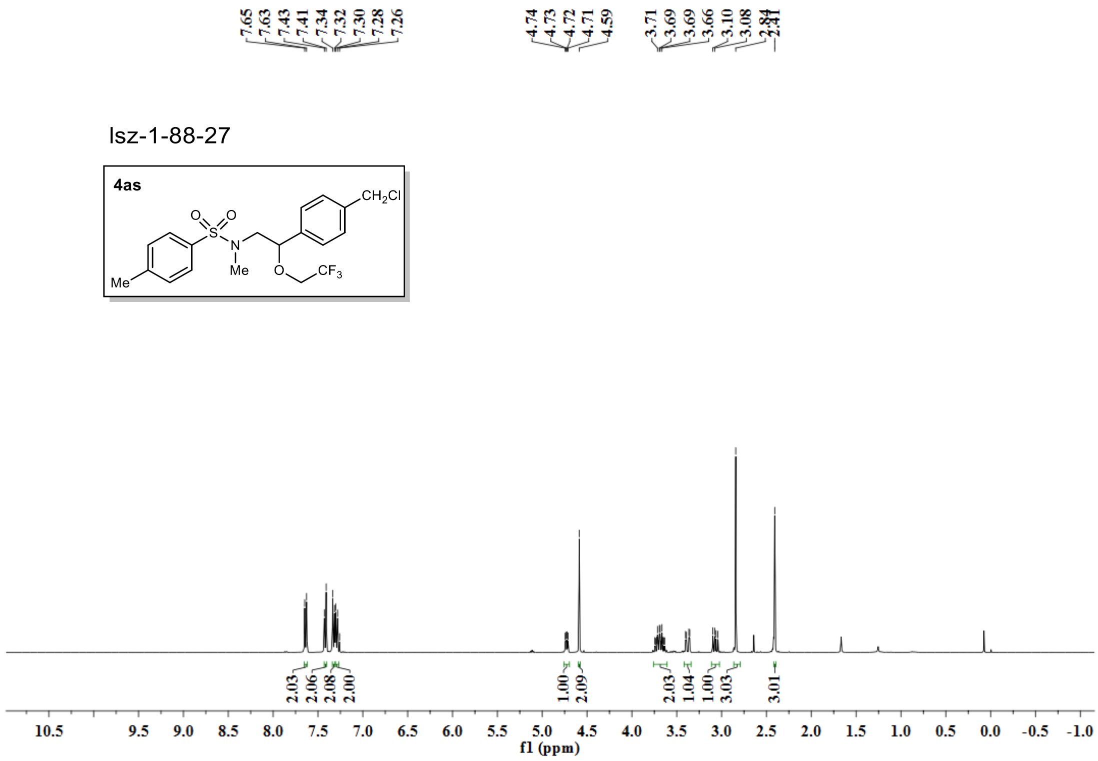

^{13}C NMR spectrum of 4as (101 MHz, $CDCl_3$):

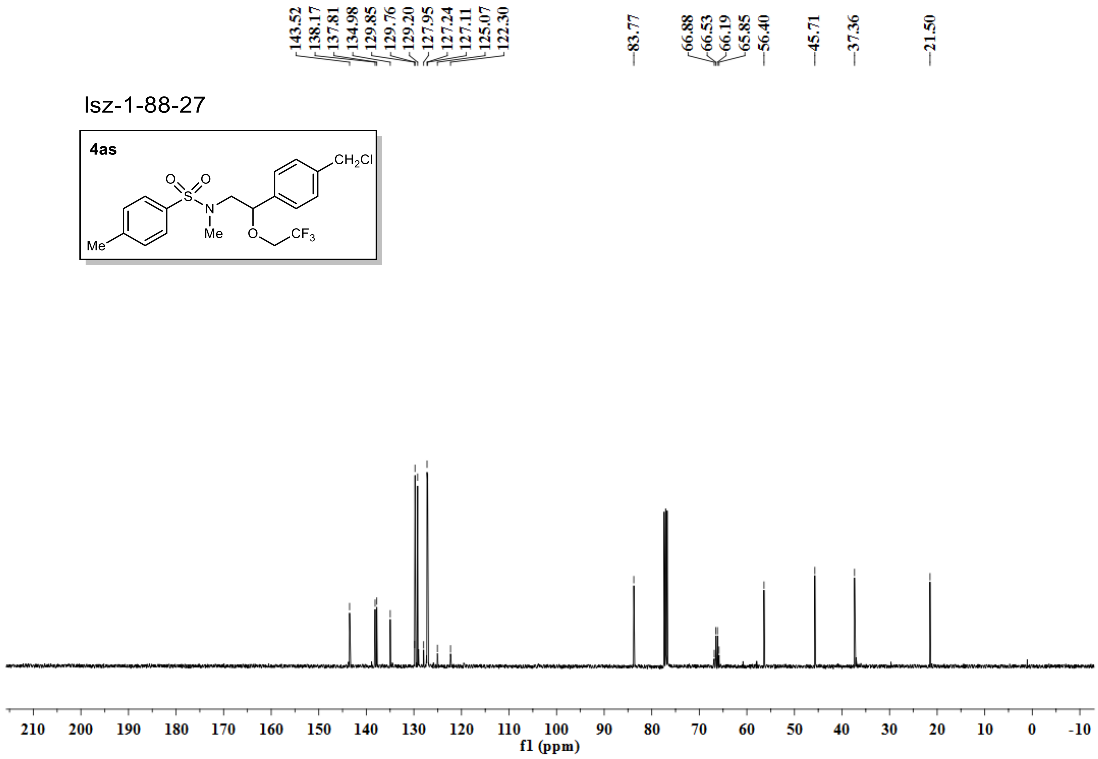

^{19}F NMR spectrum of 4as (377 MHz, $CDCl_3$):

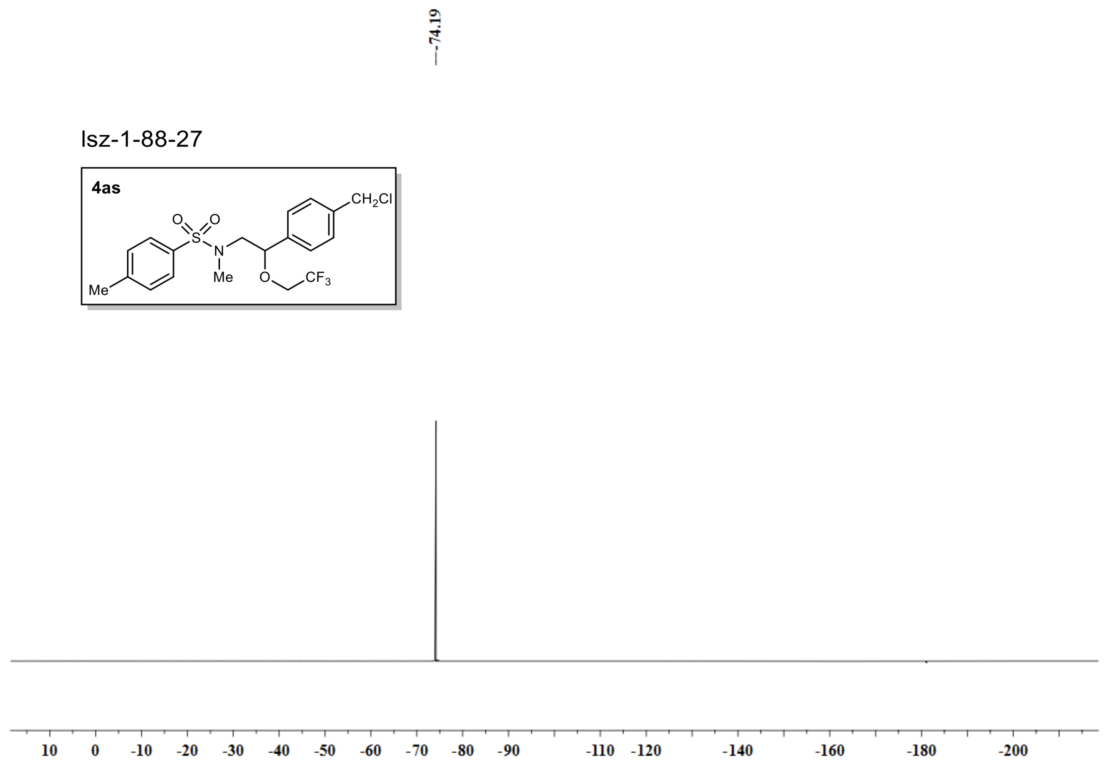

^{1}H NMR spectrum of 4at (400 MHz, $CDCl_3$):

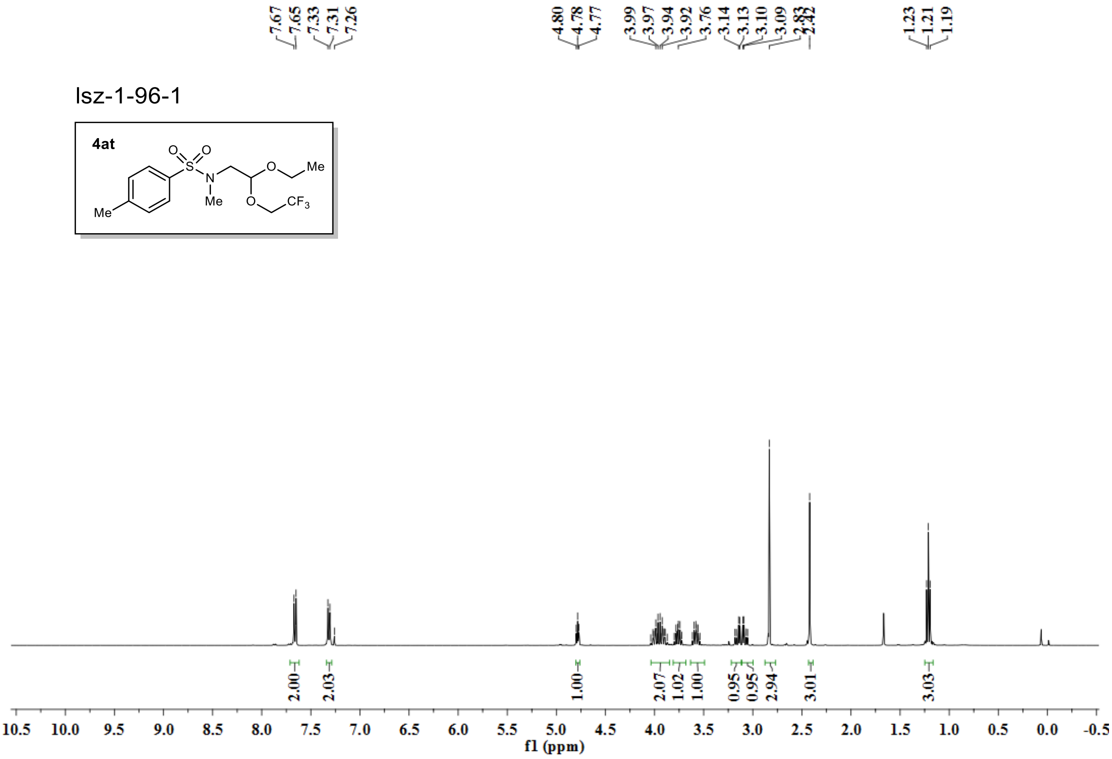

^{13}C NMR spectrum of 4at (101 MHz, $CDCl_3$):

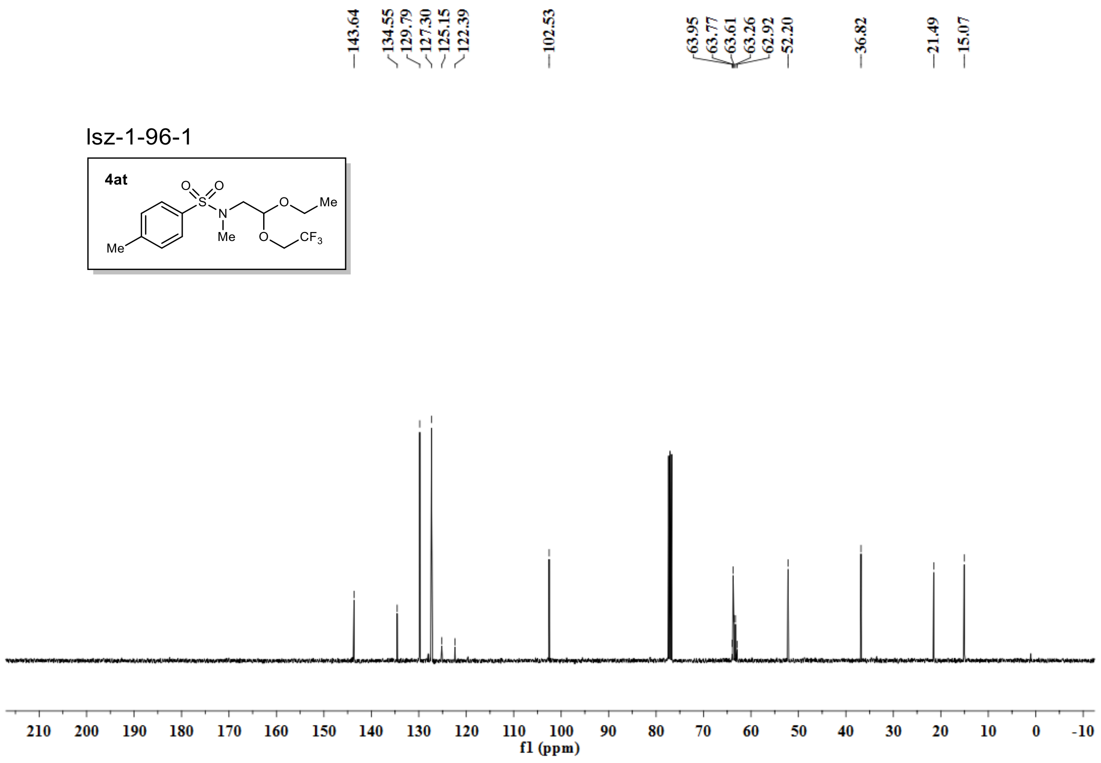

^{19}F NMR spectrum of 4at (377 MHz, $CDCl_3$):

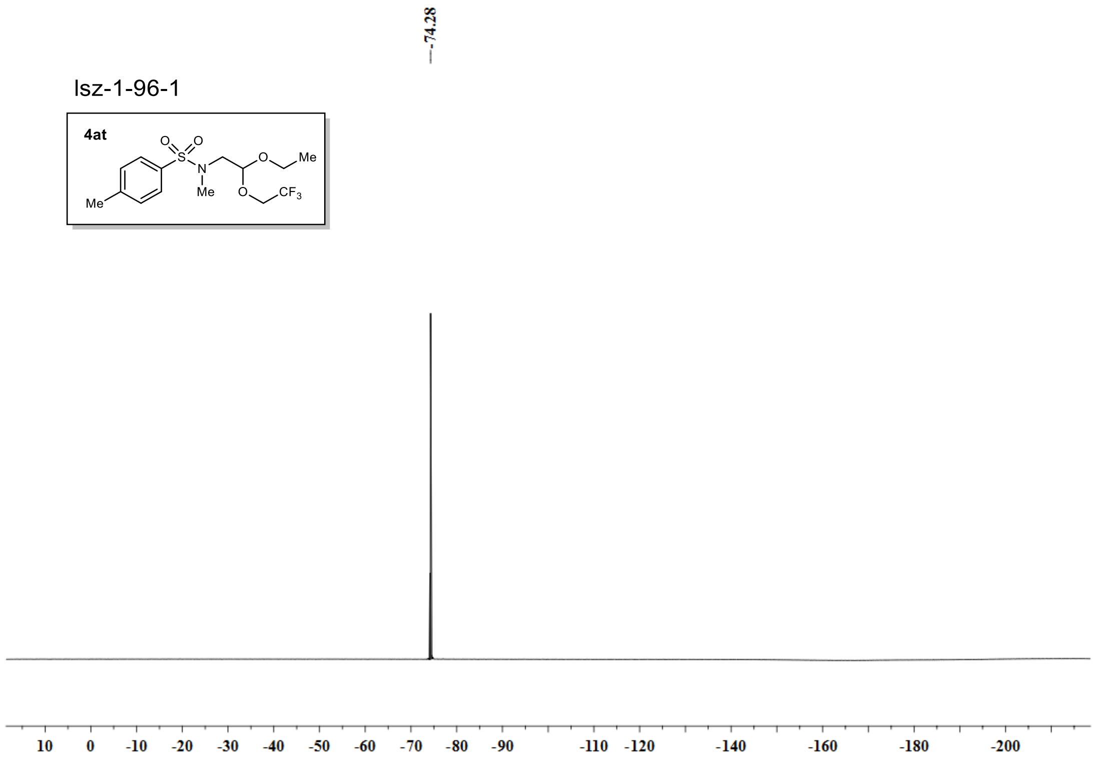

^{1}H NMR spectrum of 4au (400 MHz, $CDCl_3$):

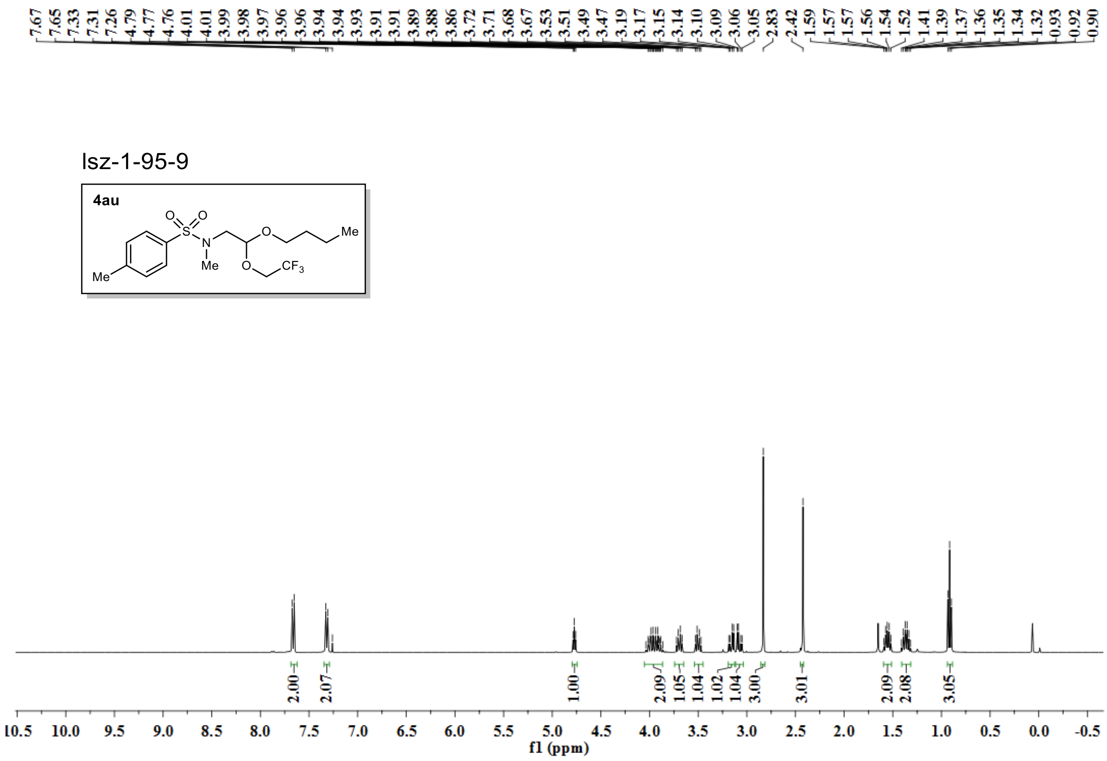

^{13}C NMR spectrum of 4au (101 MHz, $CDCl_3$):

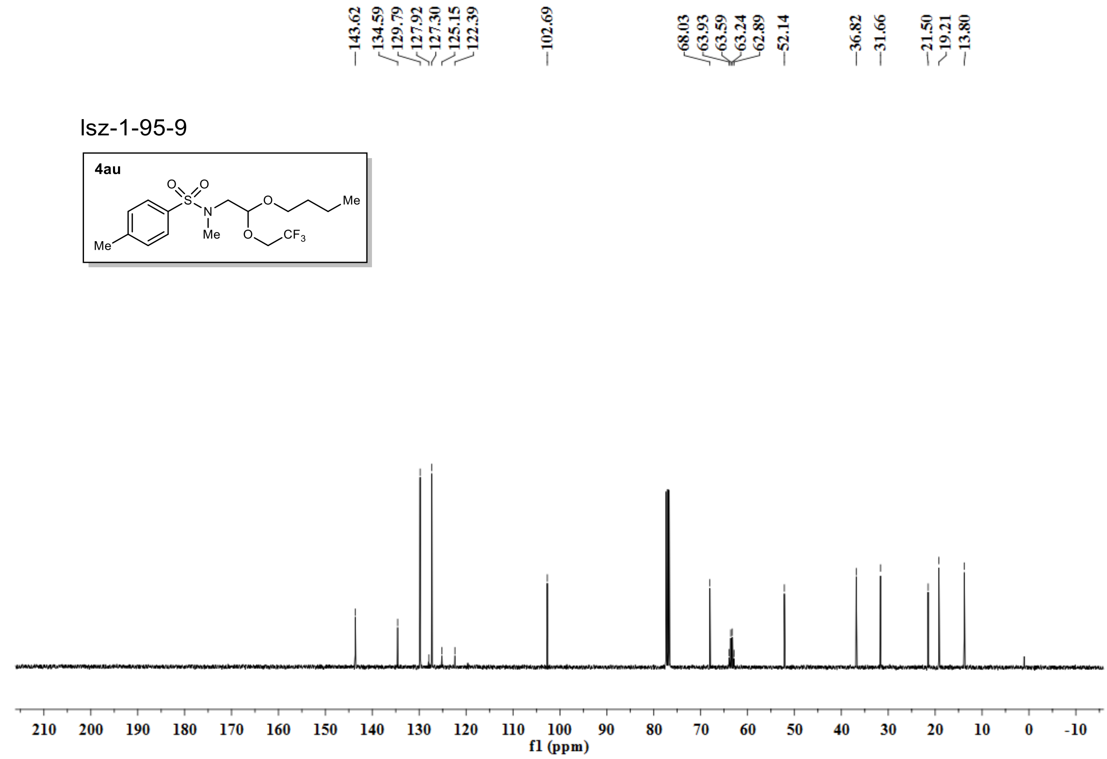

^{19}F NMR spectrum of 4au (377 MHz, $CDCl_3$):

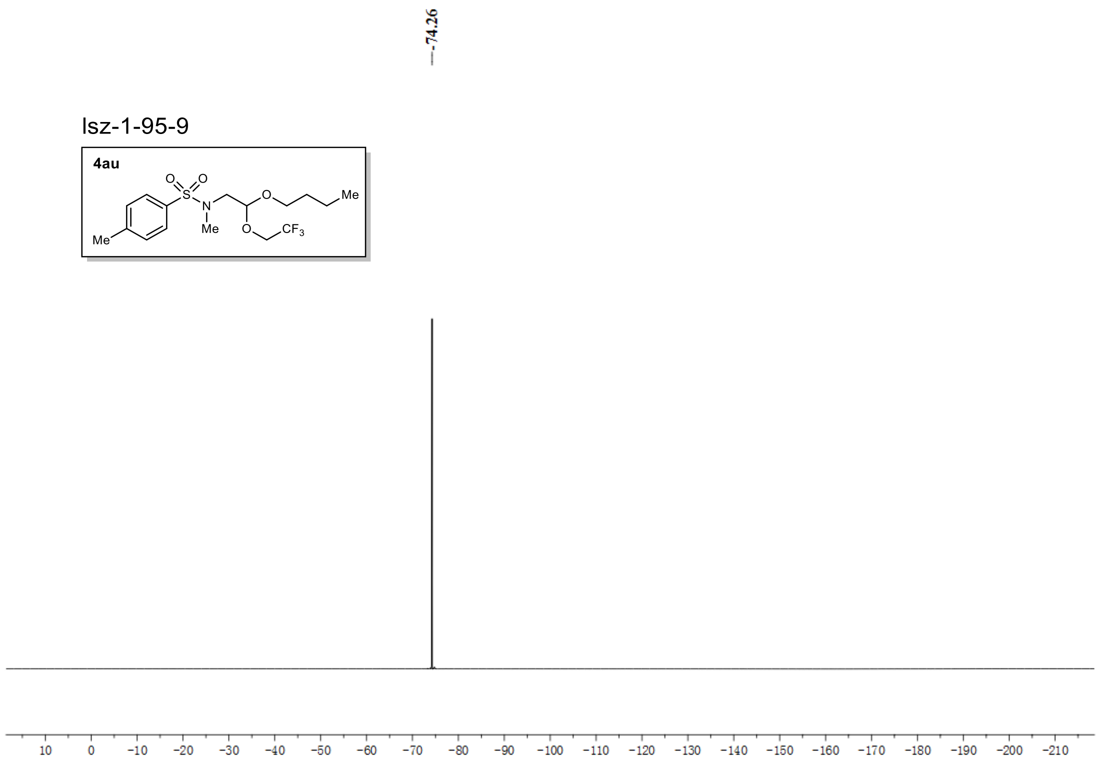

^{1}H NMR spectrum of 4av (400 MHz, $CDCl_3$):

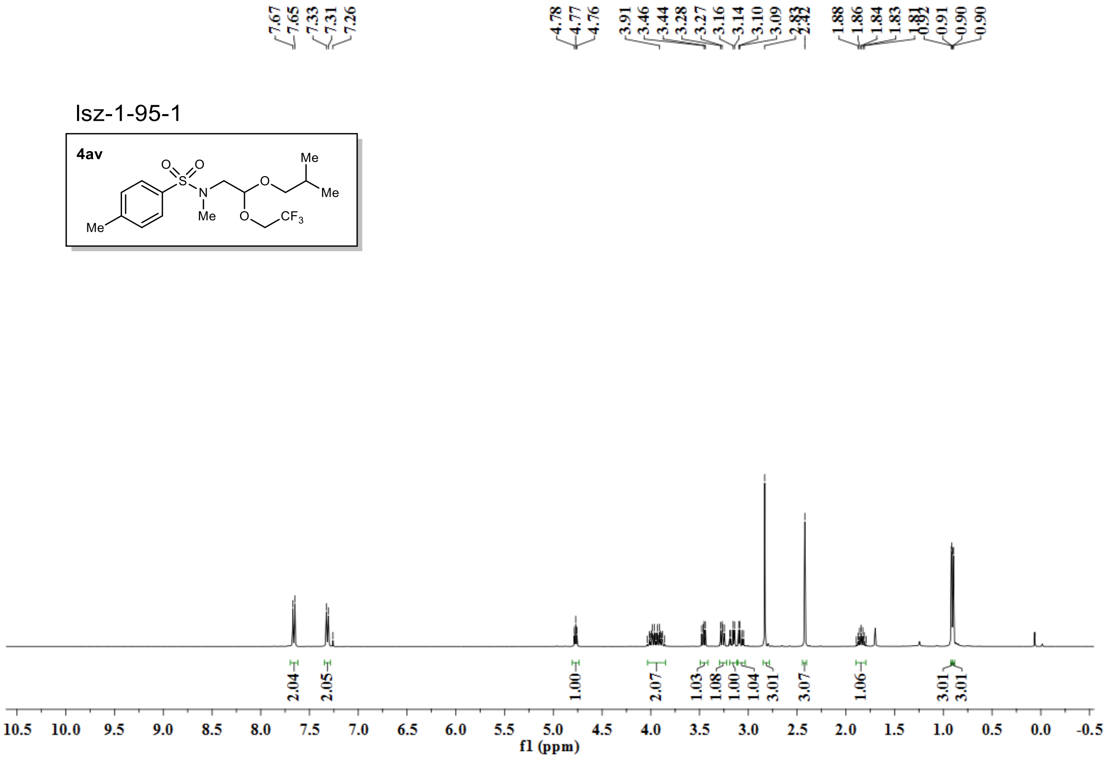

^{13}C NMR spectrum of 4av (101 MHz, $CDCl_3$):

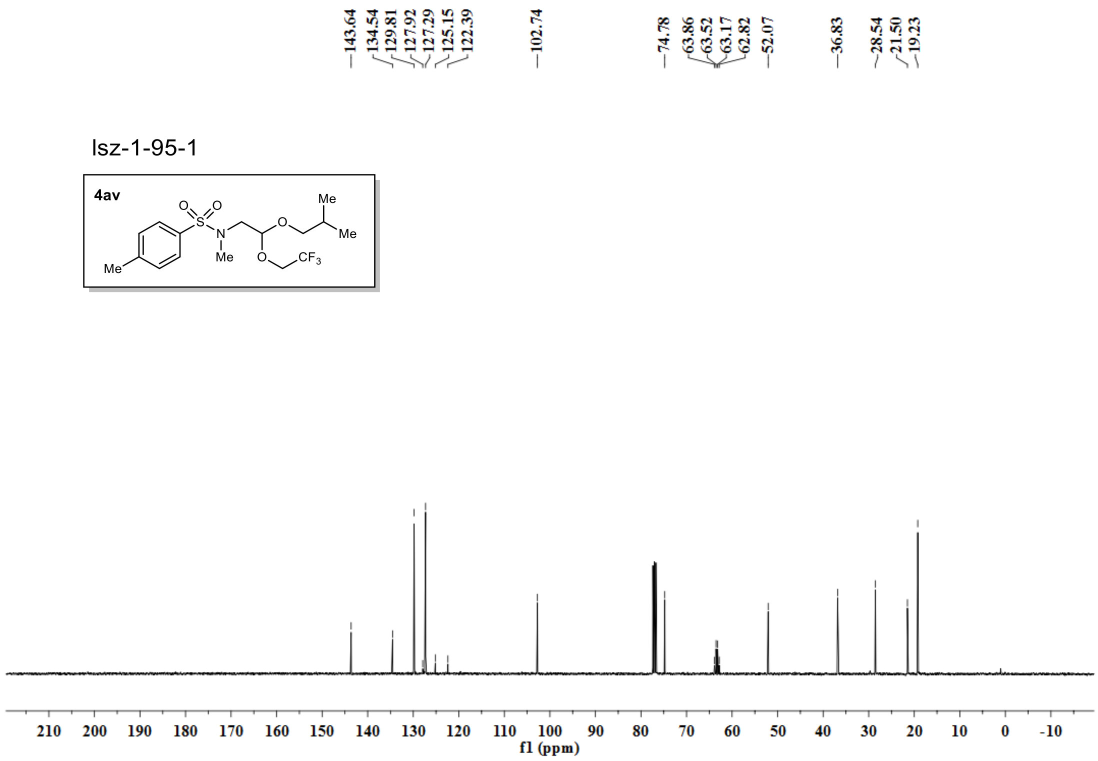

^{19}F NMR spectrum of 4av (377 MHz, $CDCl_3$):

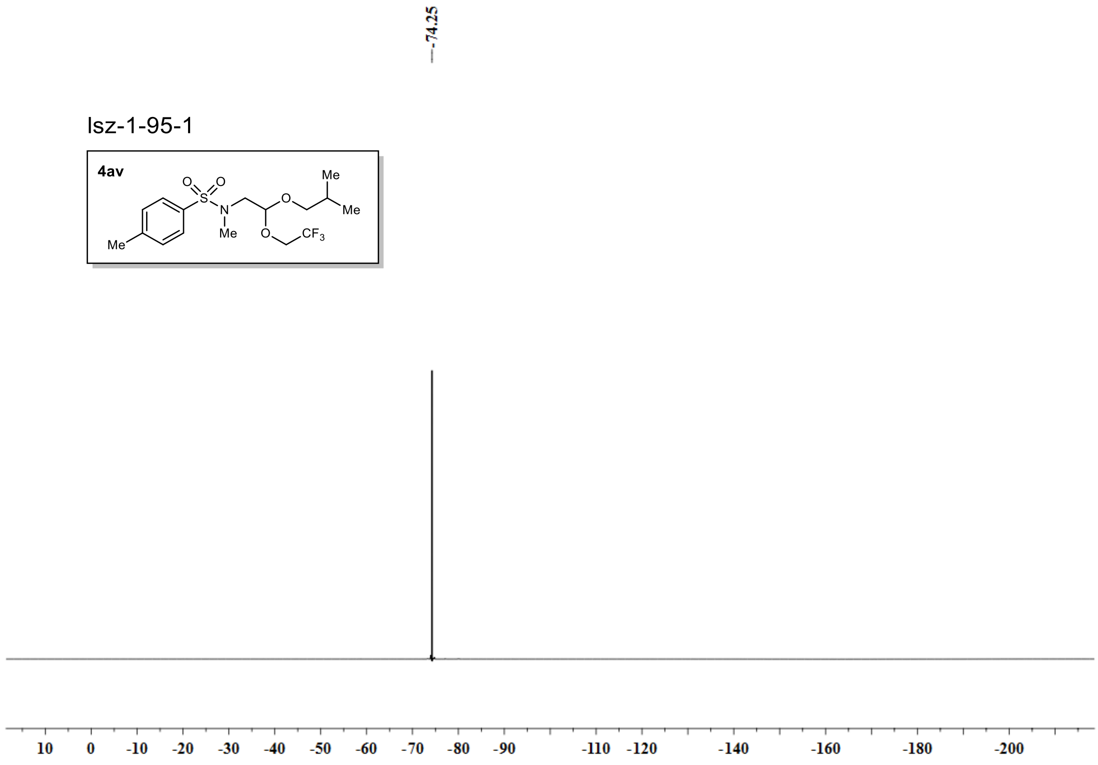

^{1}H NMR spectrum of 4aw (400 MHz, $CDCl_3$):

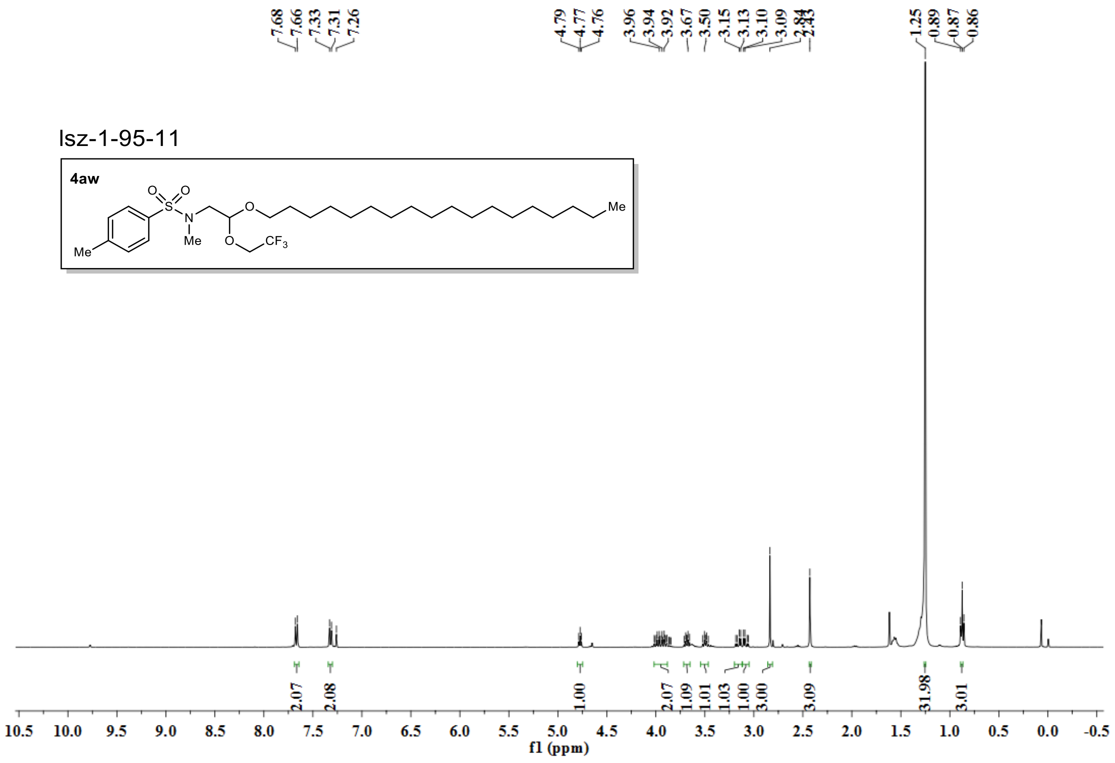

^{13}C NMR spectrum of 4aw (101 MHz, $CDCl_3$):

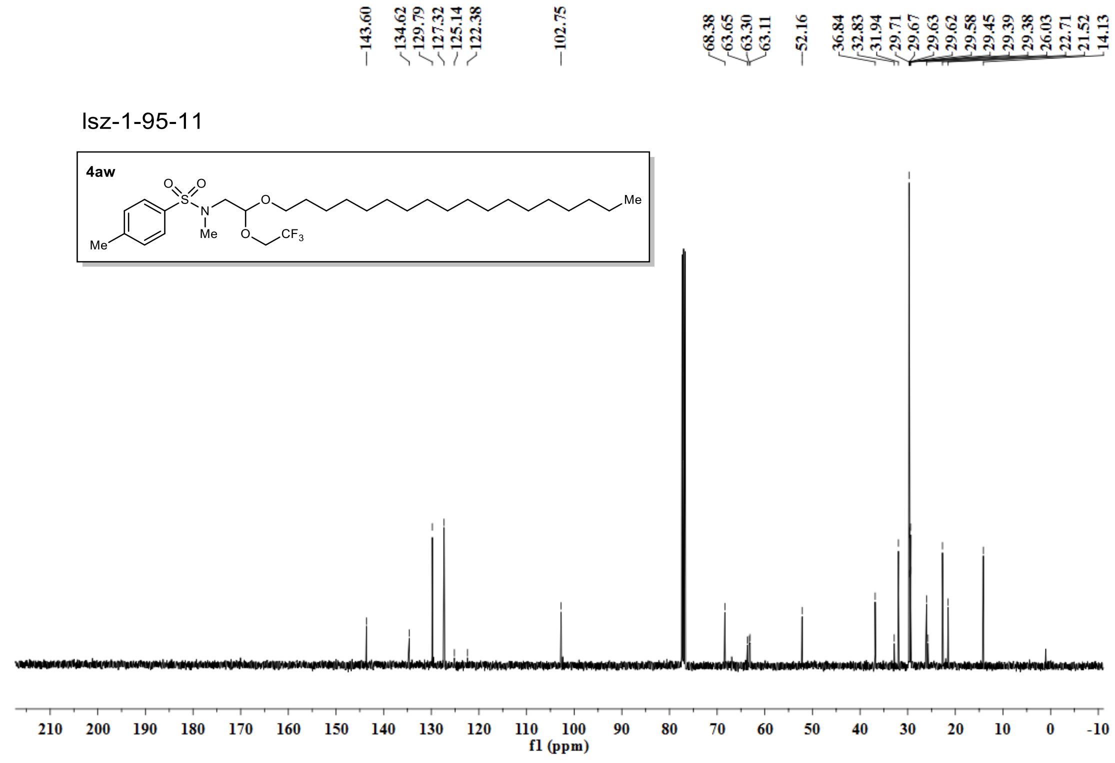

^{19}F NMR spectrum of 4aw (377 MHz, $CDCl_3$):

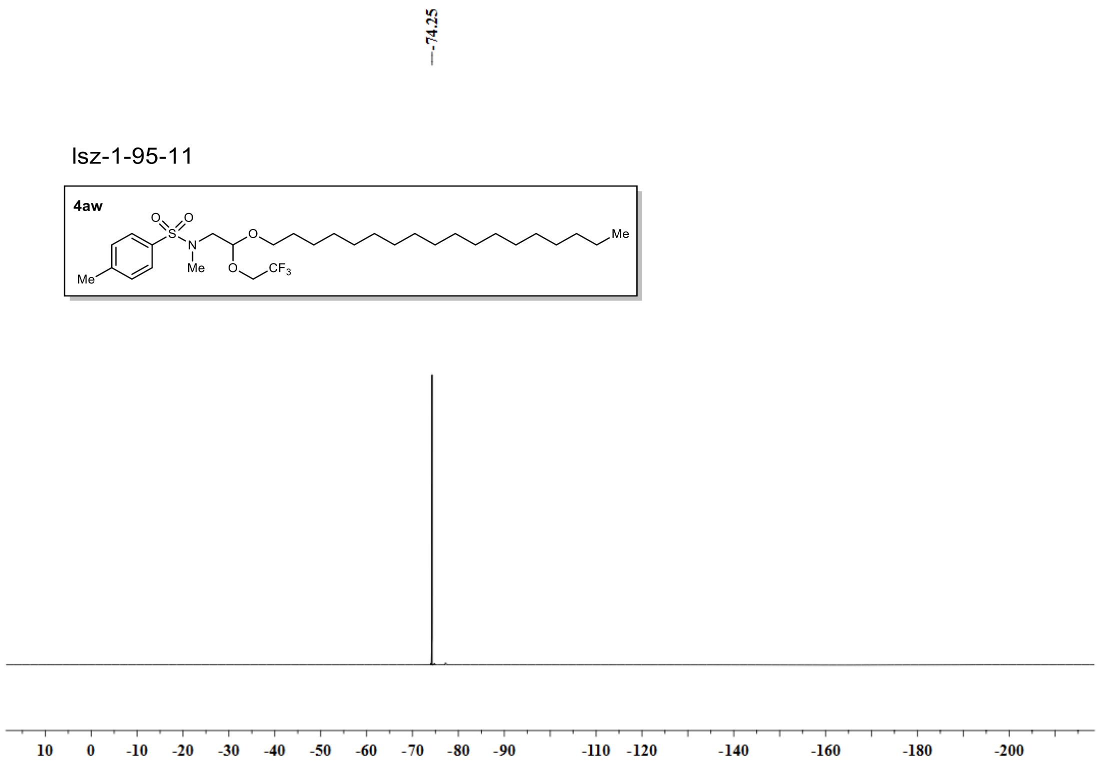

^{1}H NMR spectrum of 4ax (400 MHz, $CDCl_3$):

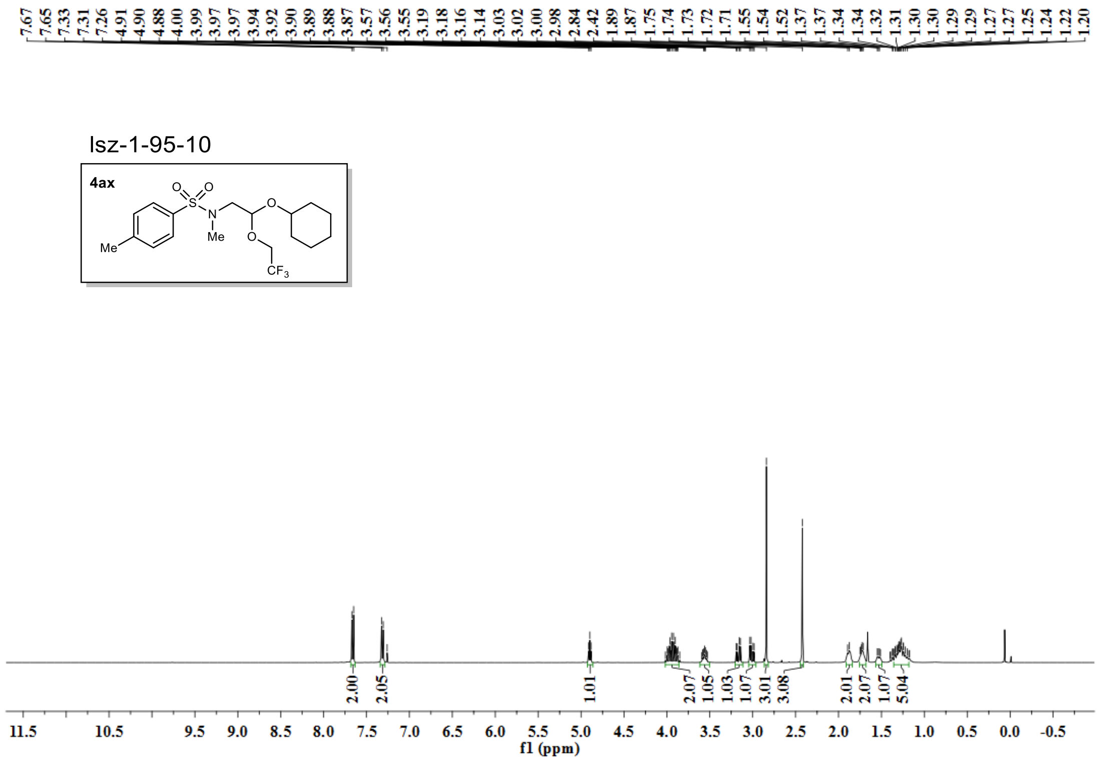

^{13}C NMR spectrum of 4ax (101 MHz, $CDCl_3$):

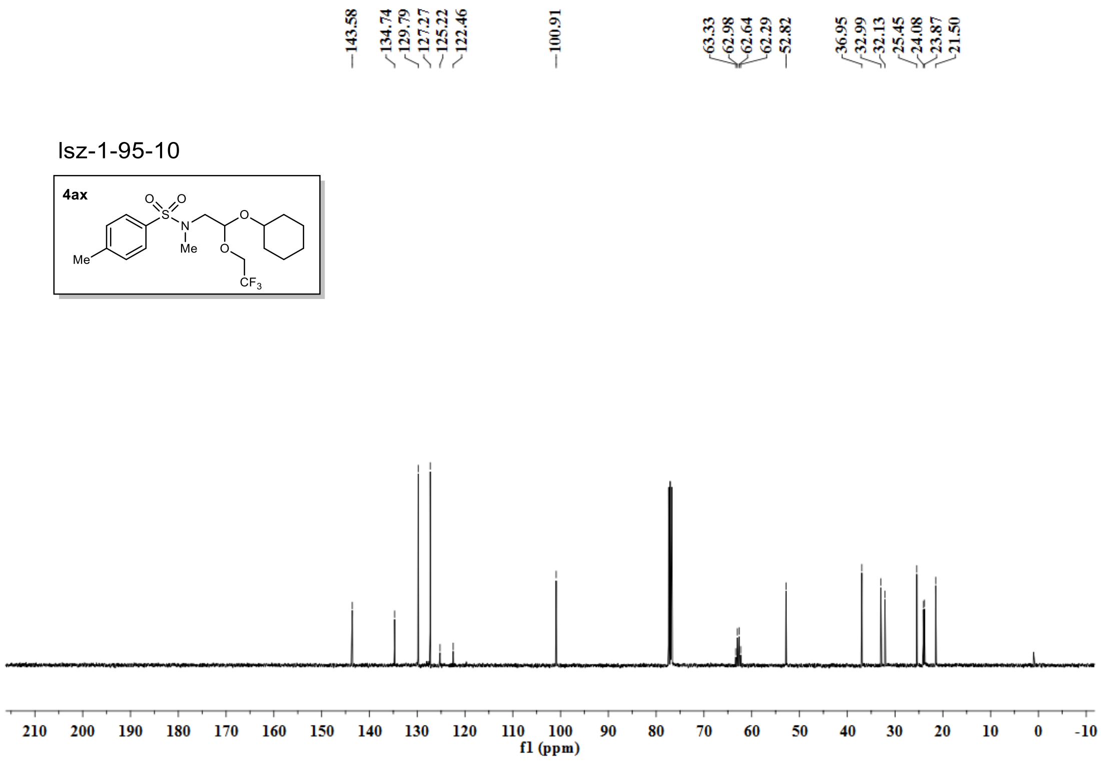

^{19}F NMR spectrum of 4ax (377 MHz, $CDCl_3$):

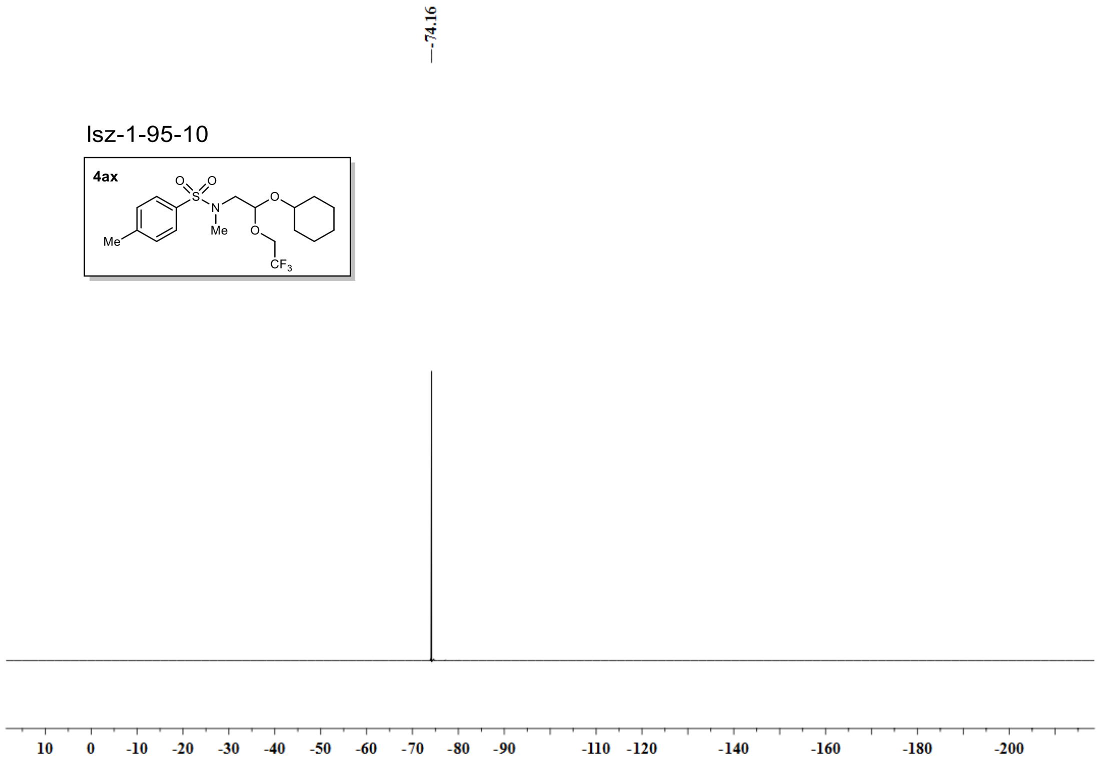

[1]**H NMR spectrum of 4ay** (400 MHz, $CDCl_3$):

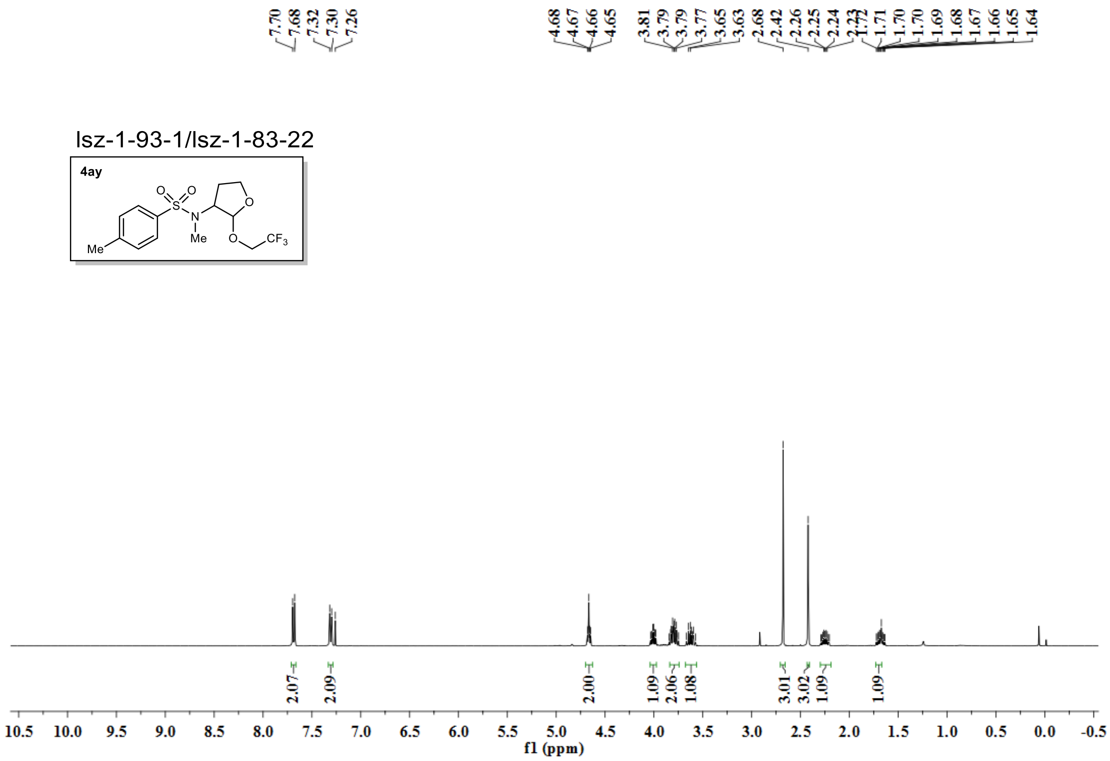

[13]**C NMR spectrum of 4ay** (101 MHz, $CDCl_3$):

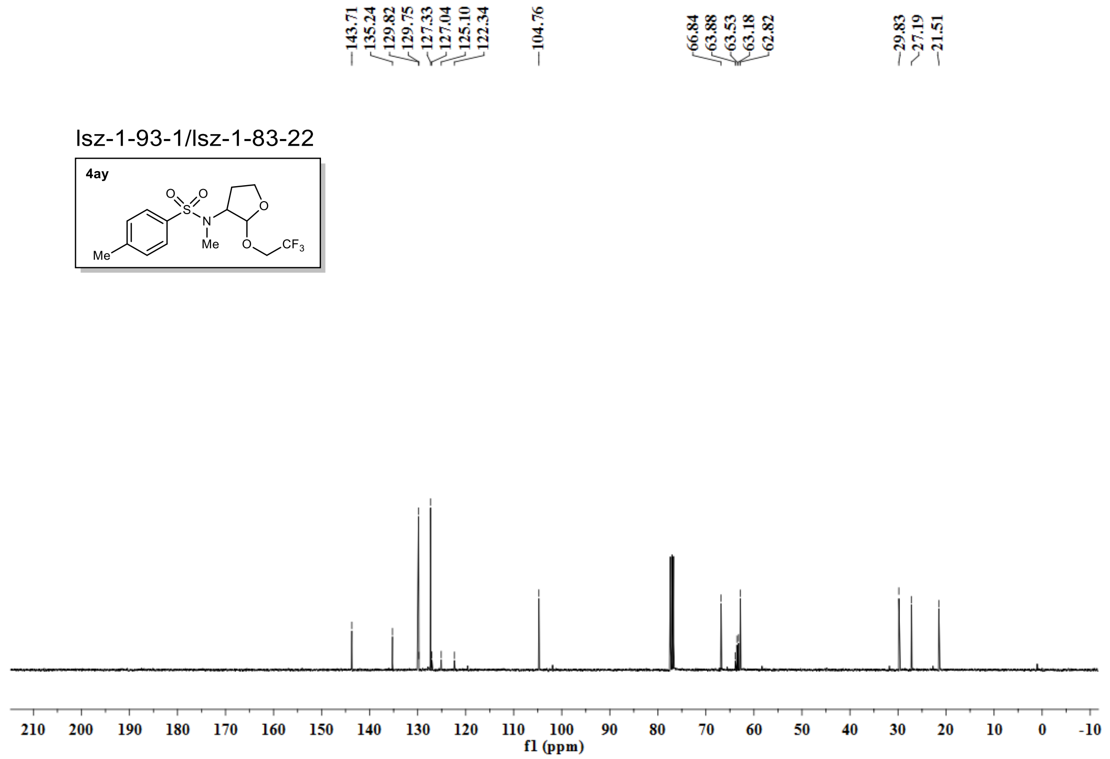

^{19}F NMR spectrum of 4ay (377 MHz, $CDCl_3$):

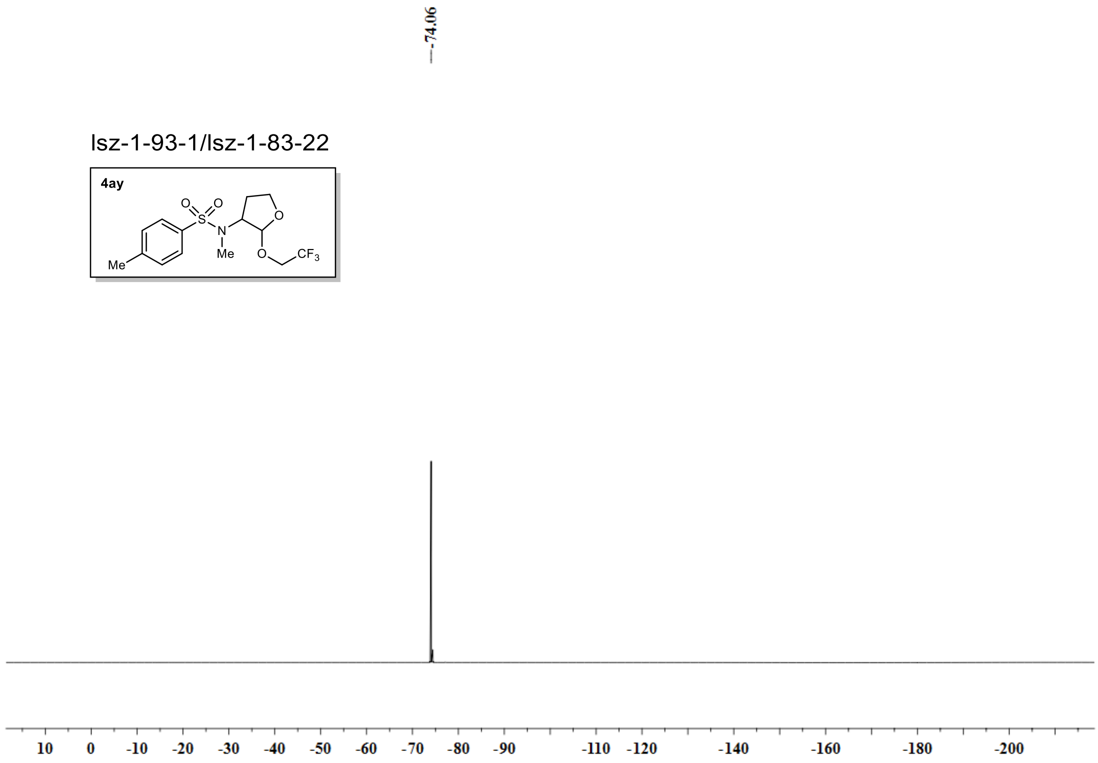

^{1}H NMR spectrum of 4az (400 MHz, $CDCl_3$):

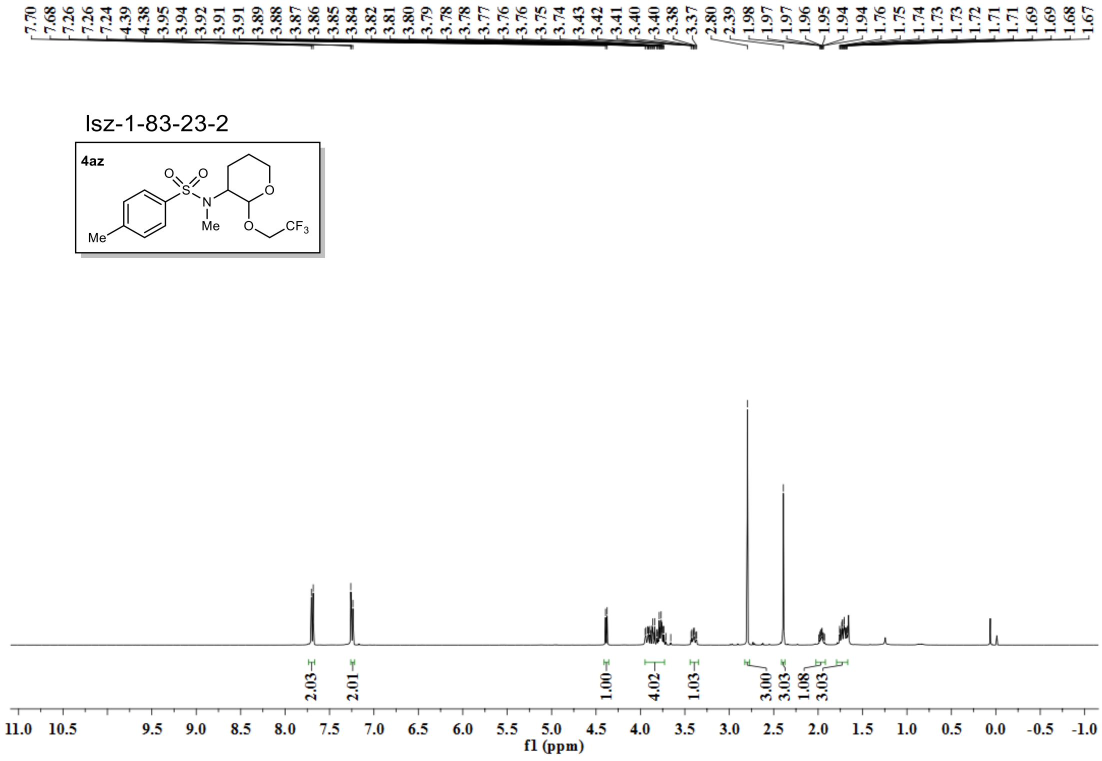

^{13}C NMR spectrum of 4az (101 MHz, $CDCl_3$):

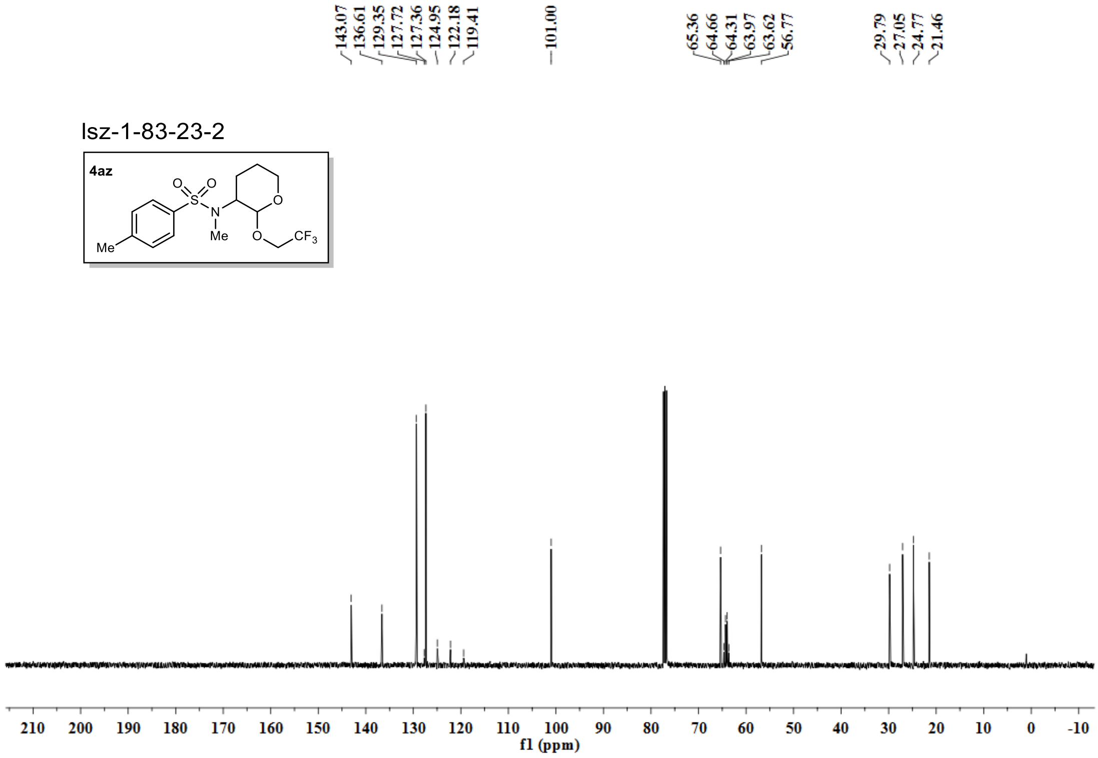

^{19}F NMR spectrum of 4az (377 MHz, $CDCl_3$):

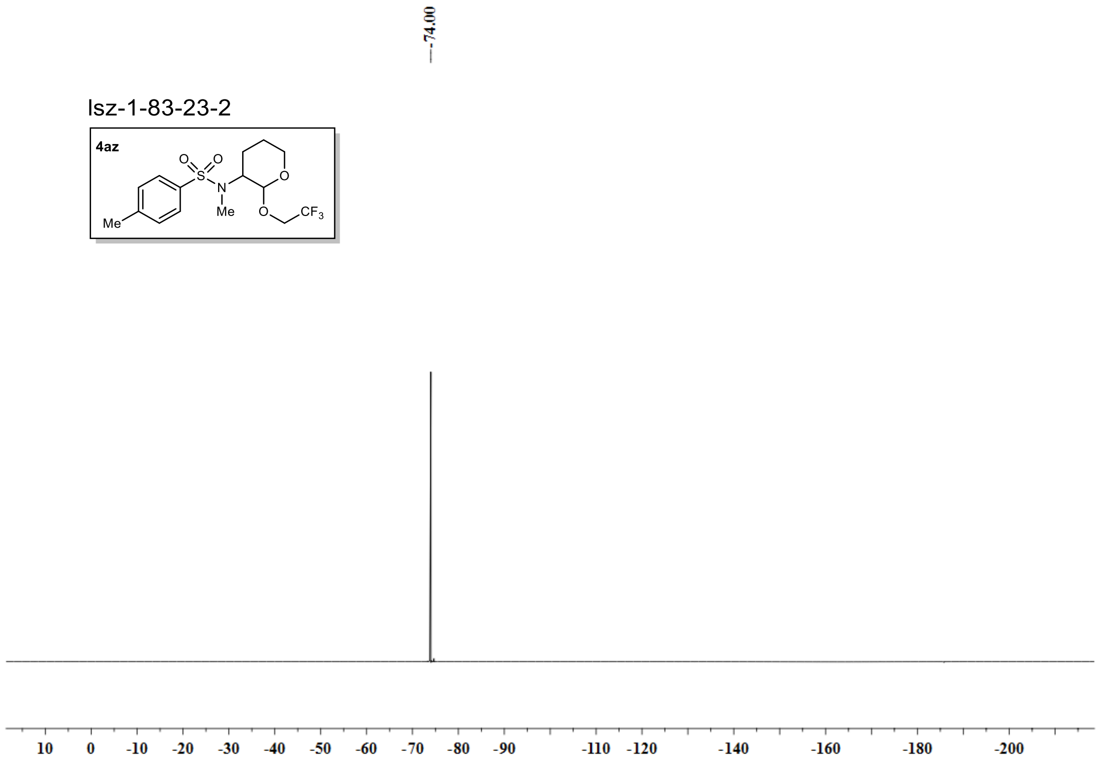

^{1}H NMR spectrum of 4ba (400 MHz, $CDCl_3$):

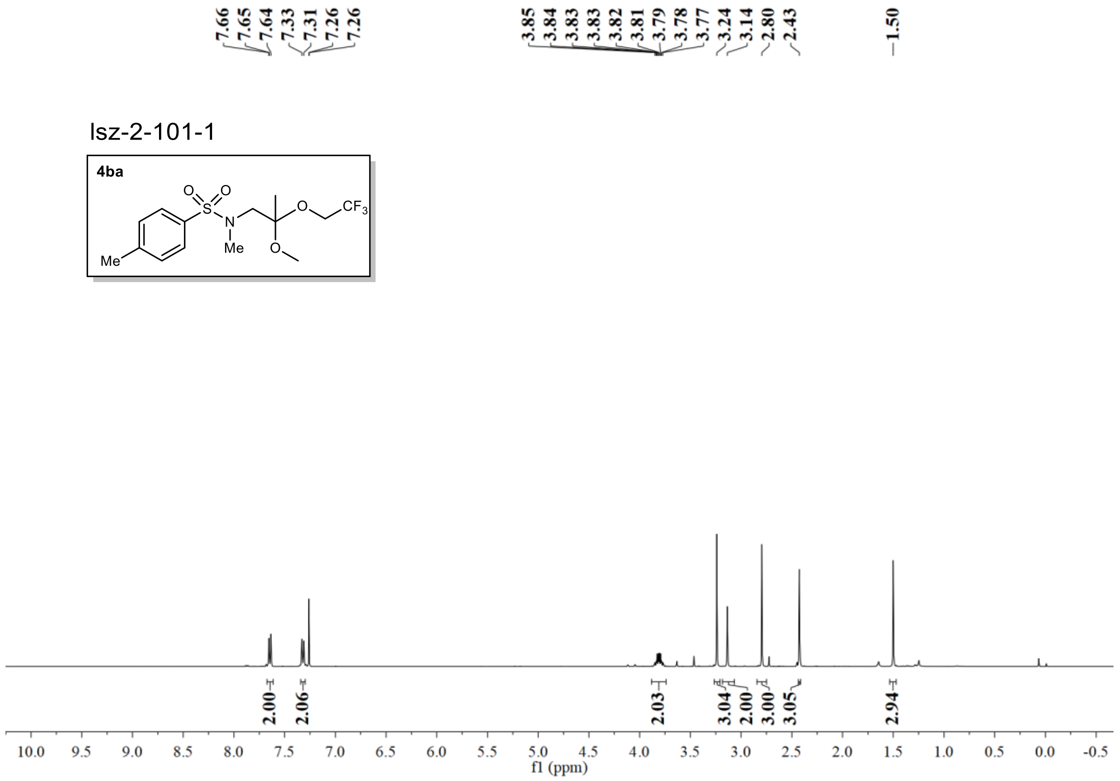

^{13}C NMR spectrum of 4ba (101 MHz, $CDCl_3$):

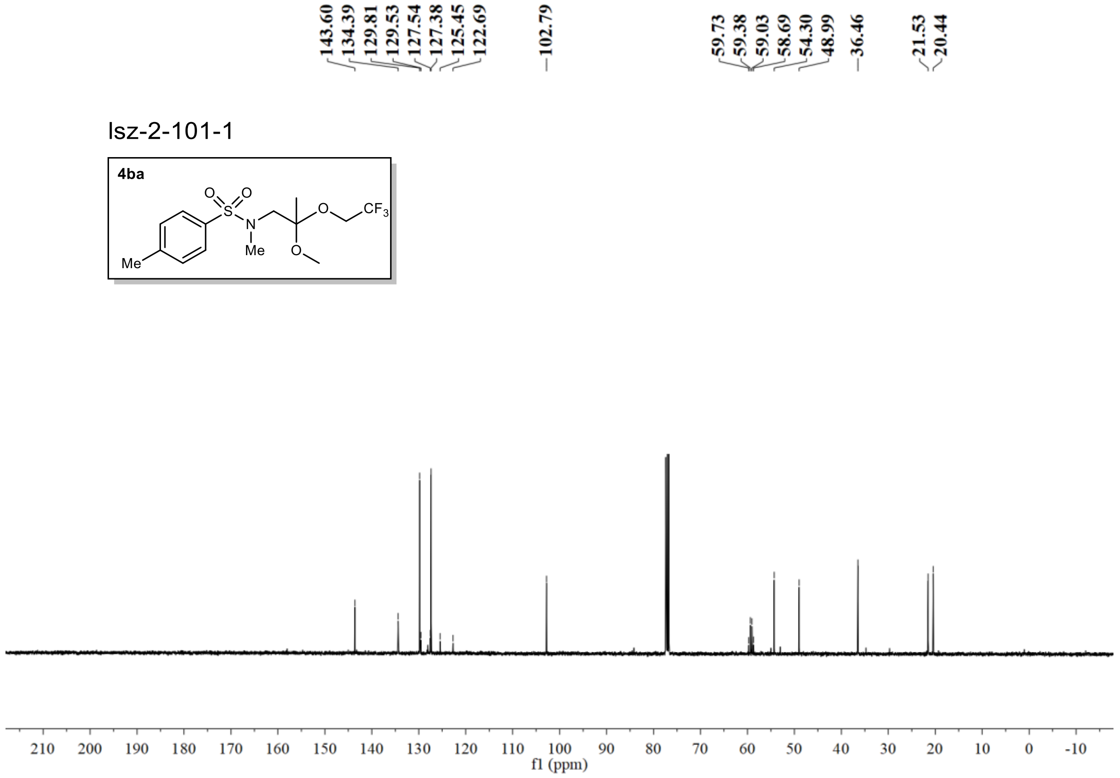

^{19}F NMR spectrum of 4ba (377 MHz, $CDCl_3$):

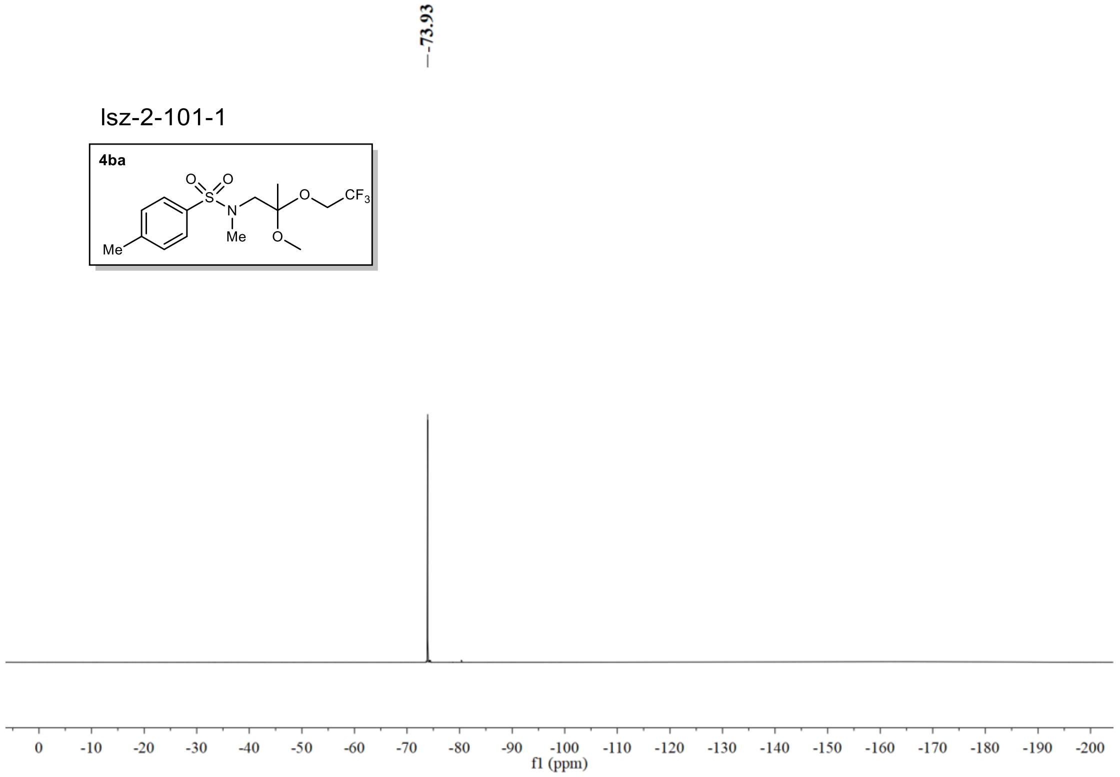

^{1}H NMR spectrum of 4bb (400 MHz, $CDCl_3$):

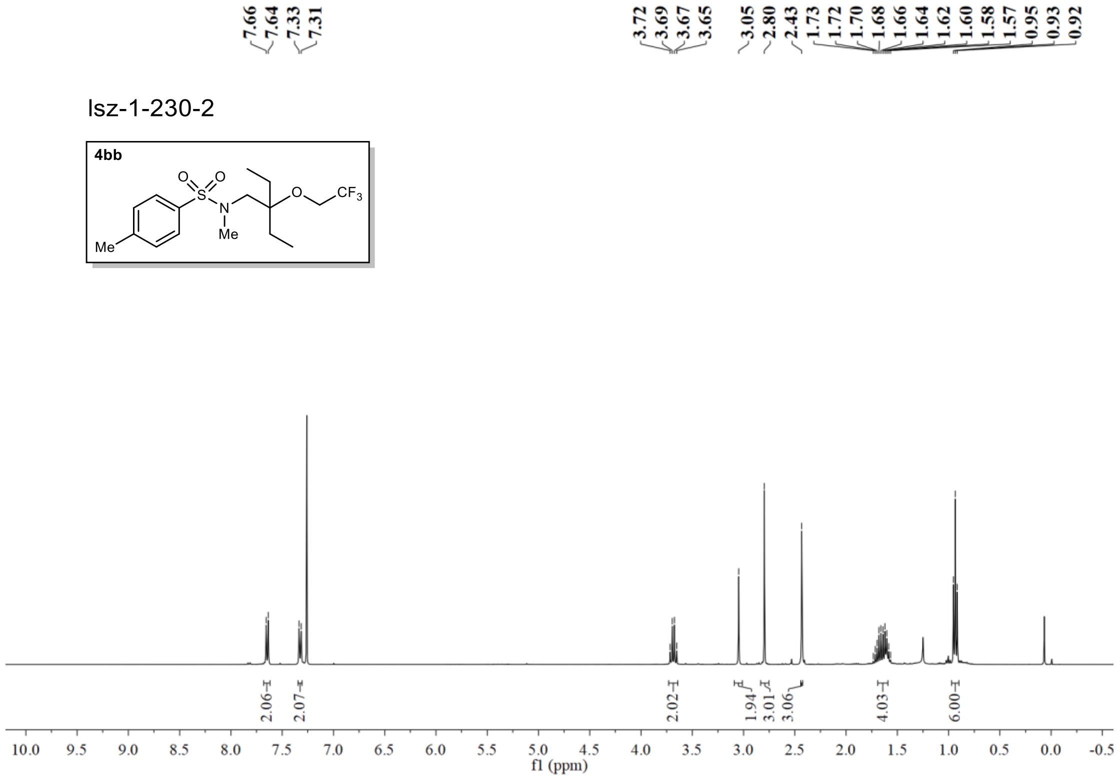

^{13}C NMR spectrum of 4bb (101 MHz, $CDCl_3$):

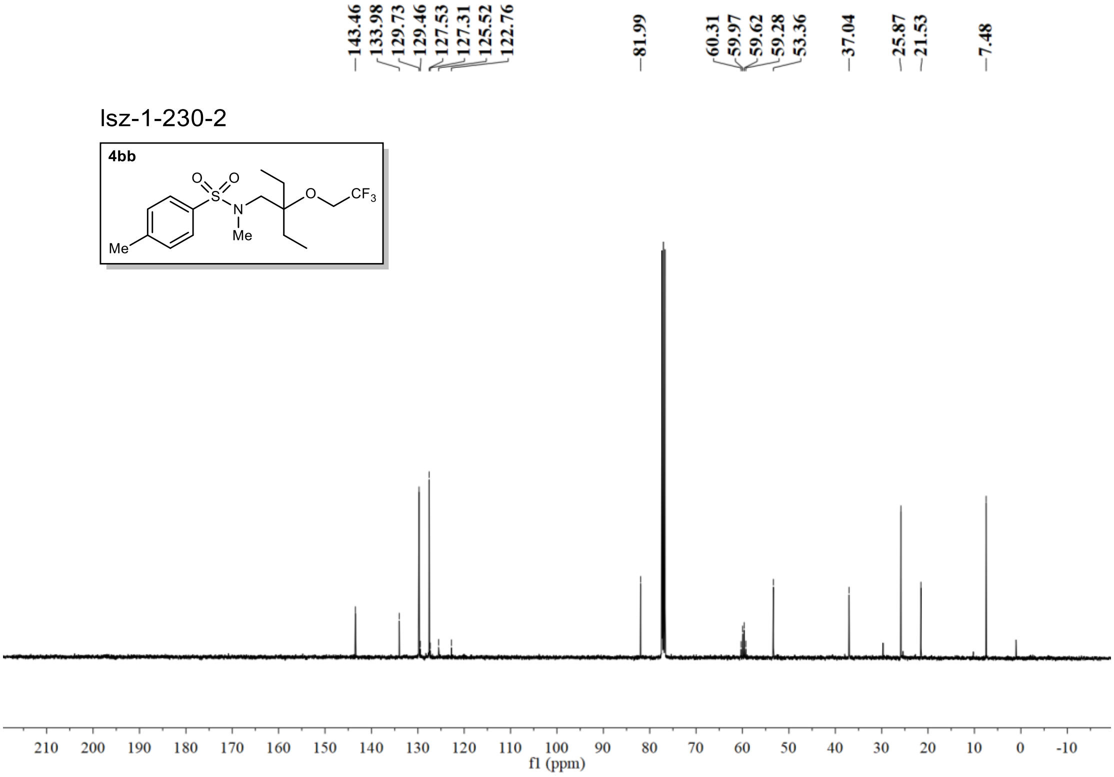

^{19}F NMR spectrum of 4bb (377 MHz, $CDCl_3$):

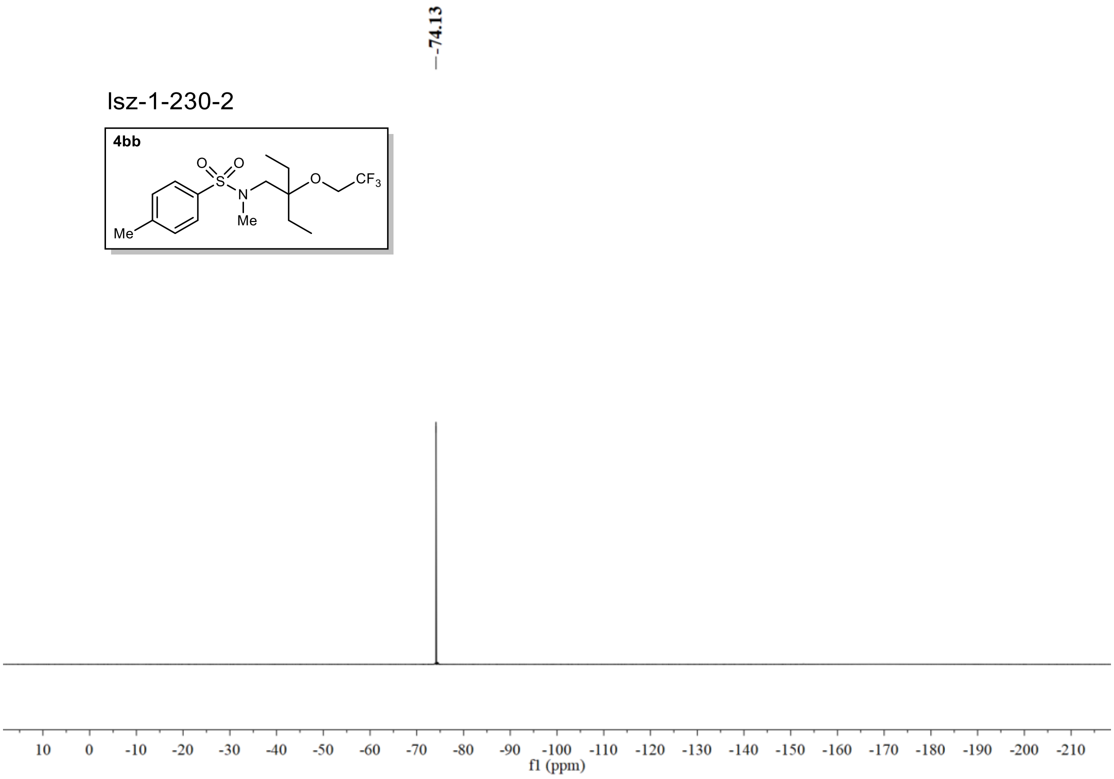

^{1}H NMR spectrum of 5a (400 MHz, $CDCl_3$):

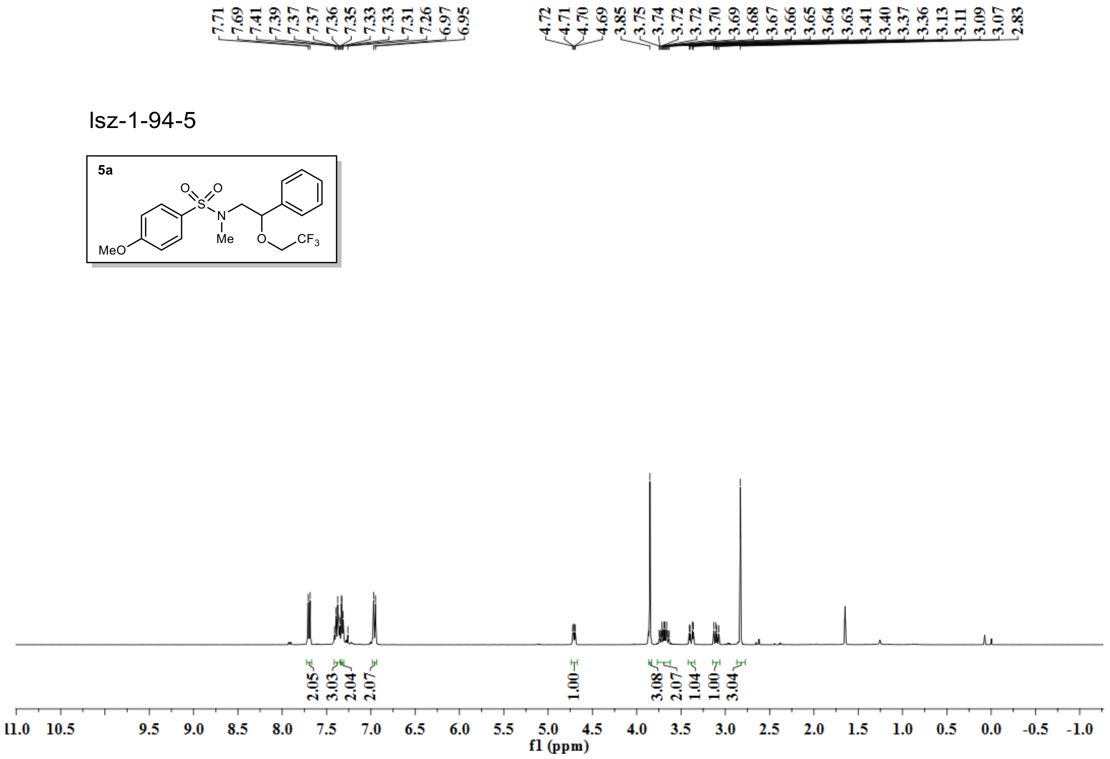

^{13}C NMR spectrum of 5a (101 MHz, $CDCl_3$):

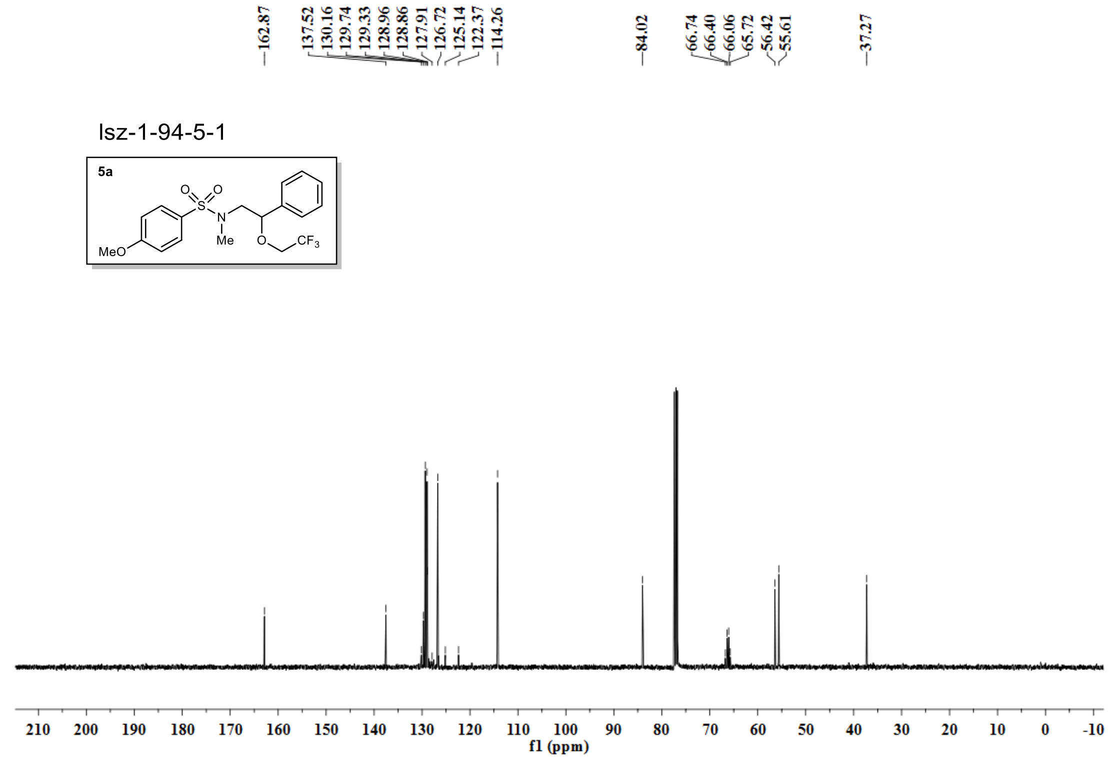

^{19}F NMR spectrum of 5a (377 MHz, $CDCl_3$):

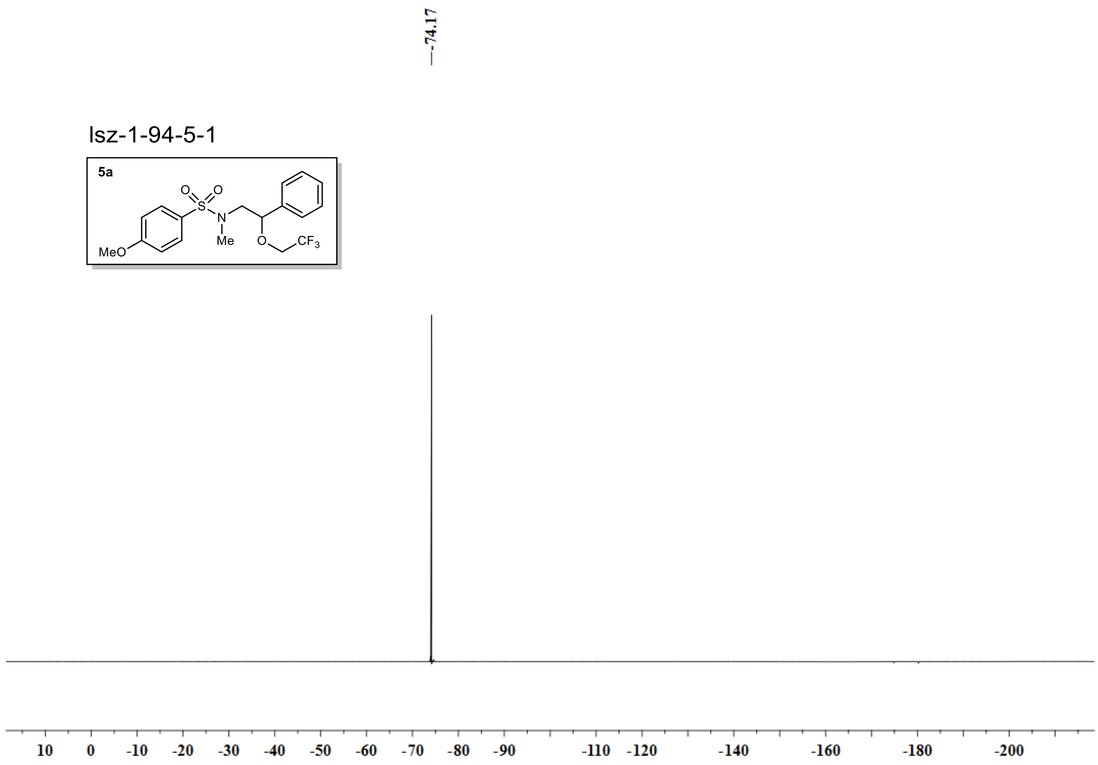

^{1}H NMR spectrum of 5b (400 MHz, $CDCl_3$):

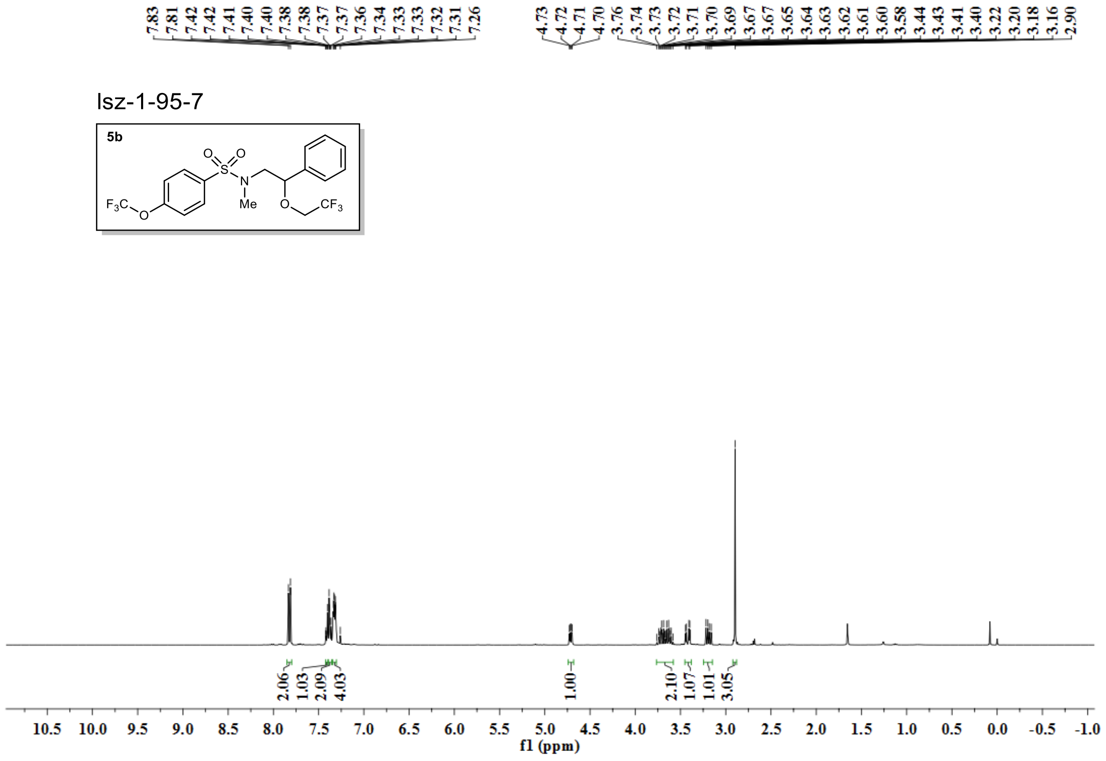

^{13}C NMR spectrum of 5b (101 MHz, $CDCl_3$):

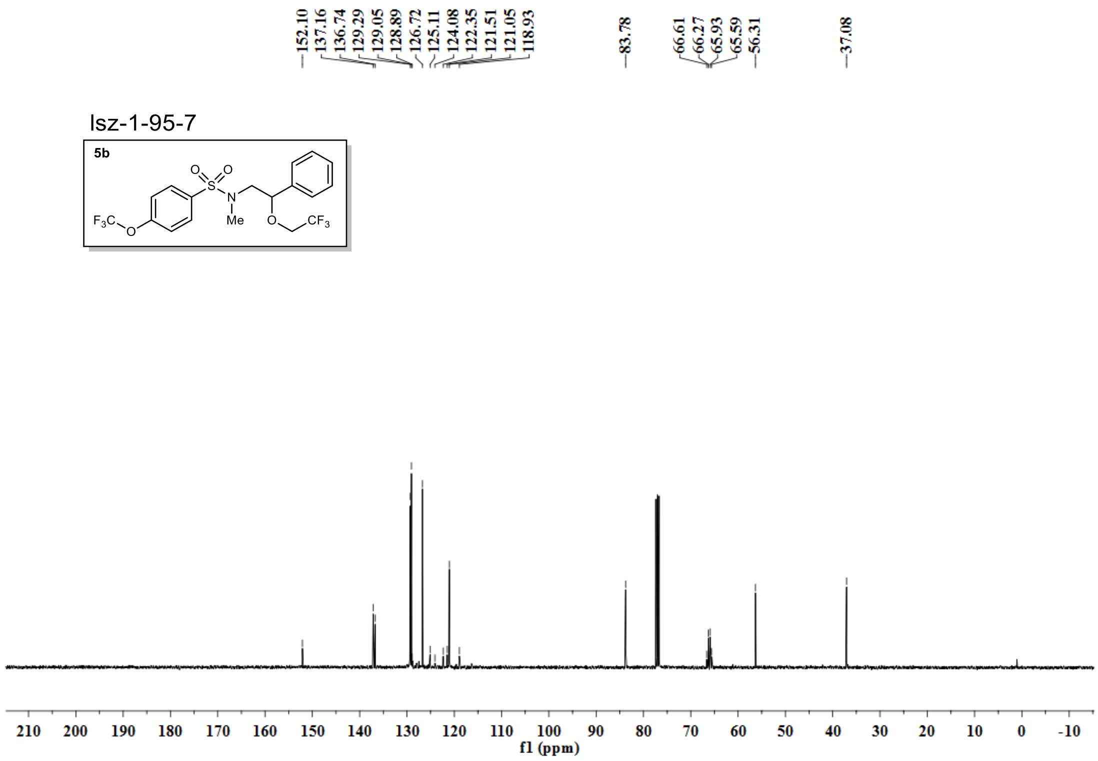

^{19}F NMR spectrum of 5b (377 MHz, $CDCl_3$):

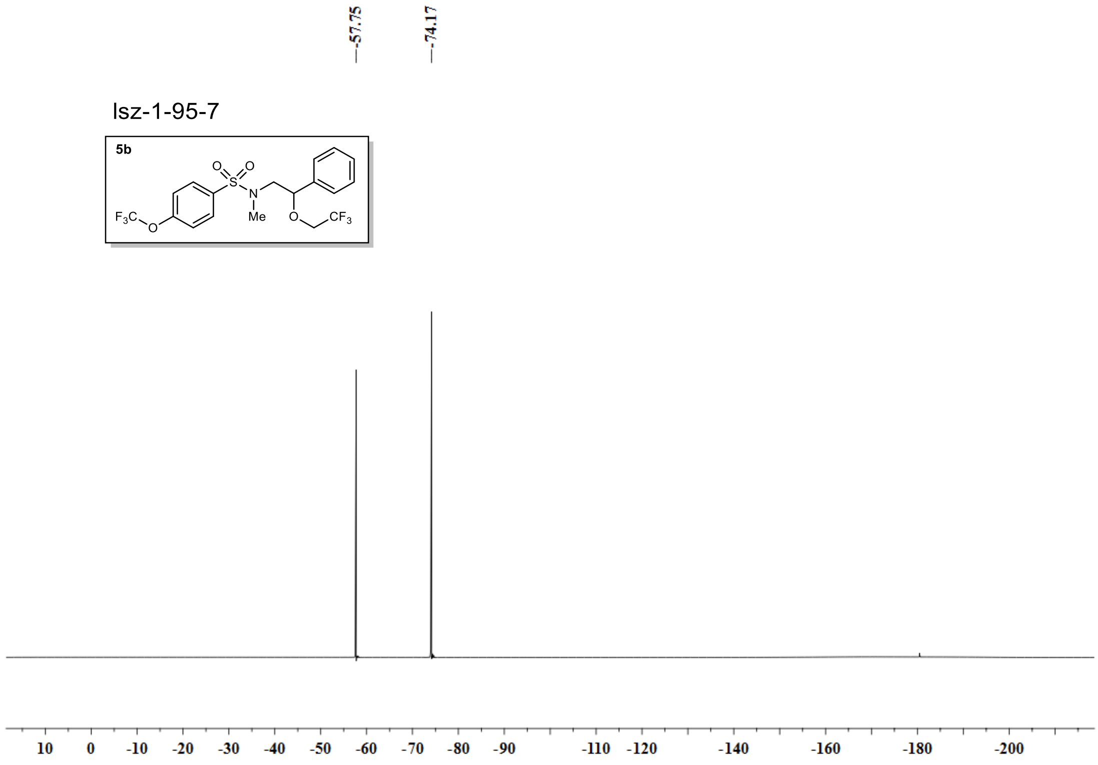

^{1}H NMR spectrum of 5c (400 MHz, $CDCl_3$):

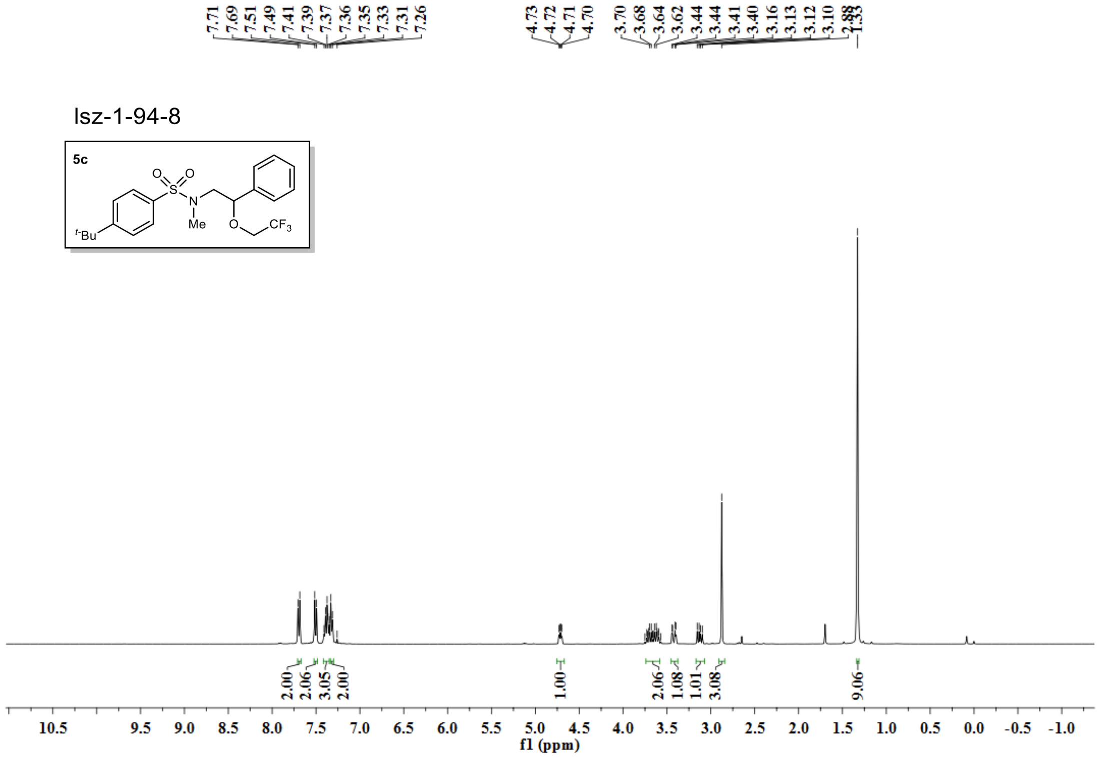

^{13}C NMR spectrum of 5c (101 MHz, $CDCl_3$):

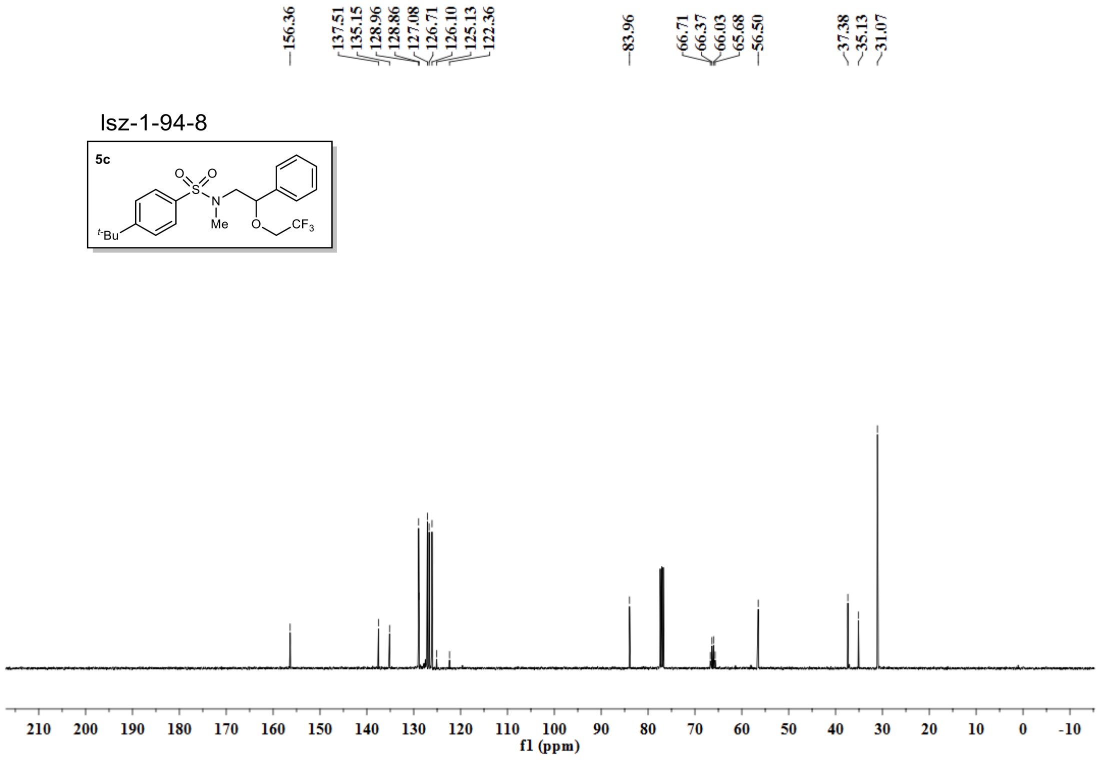

^{19}F NMR spectrum of 5c (377 MHz, $CDCl_3$):

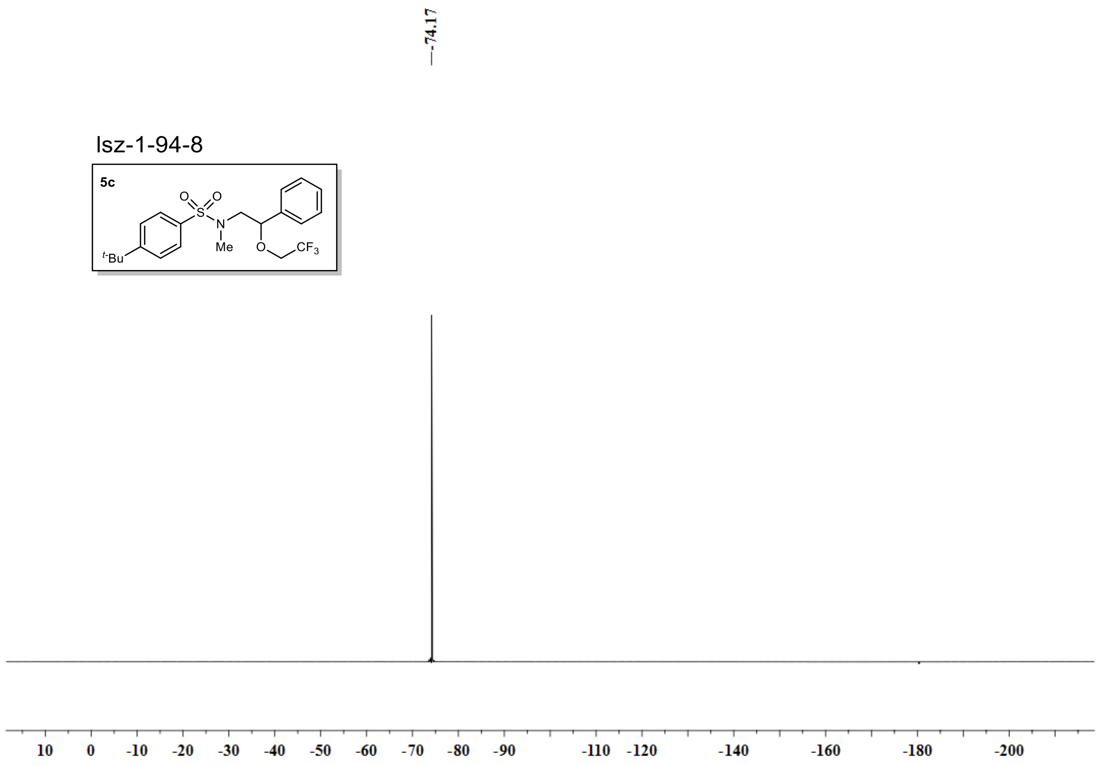

^{1}H NMR spectrum of 5d (400 MHz, $CDCl_3$):

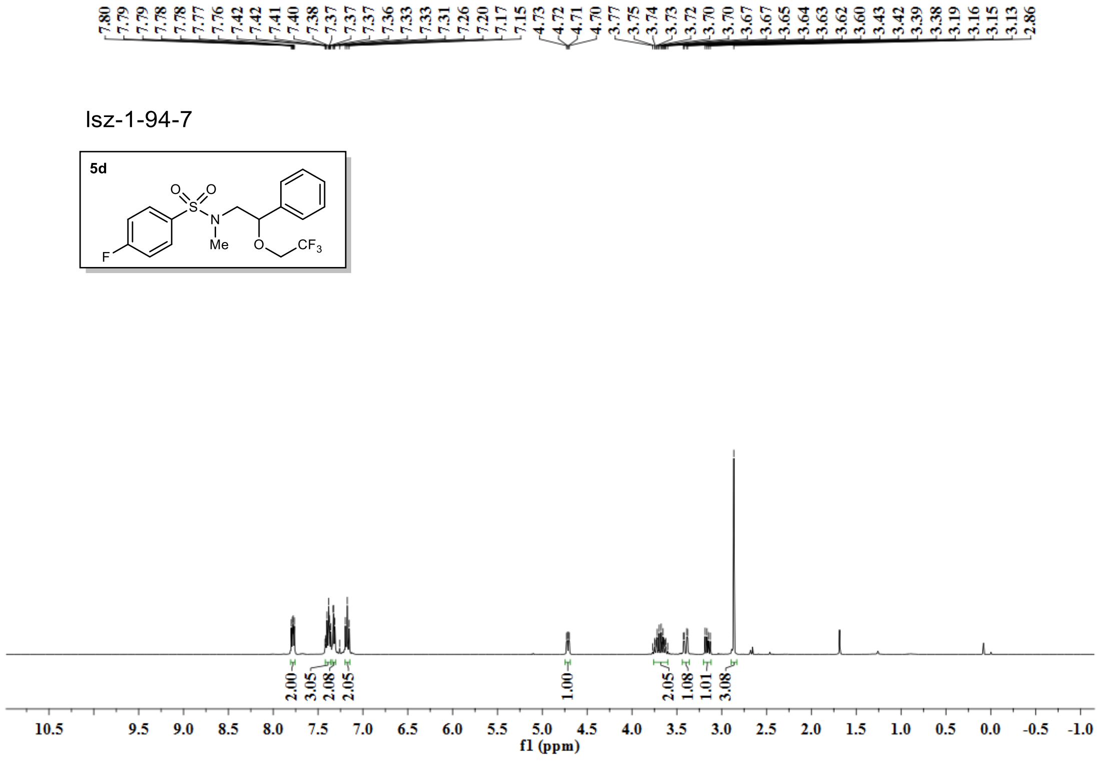

^{13}C NMR spectrum of 5d (101 MHz, $CDCl_3$):

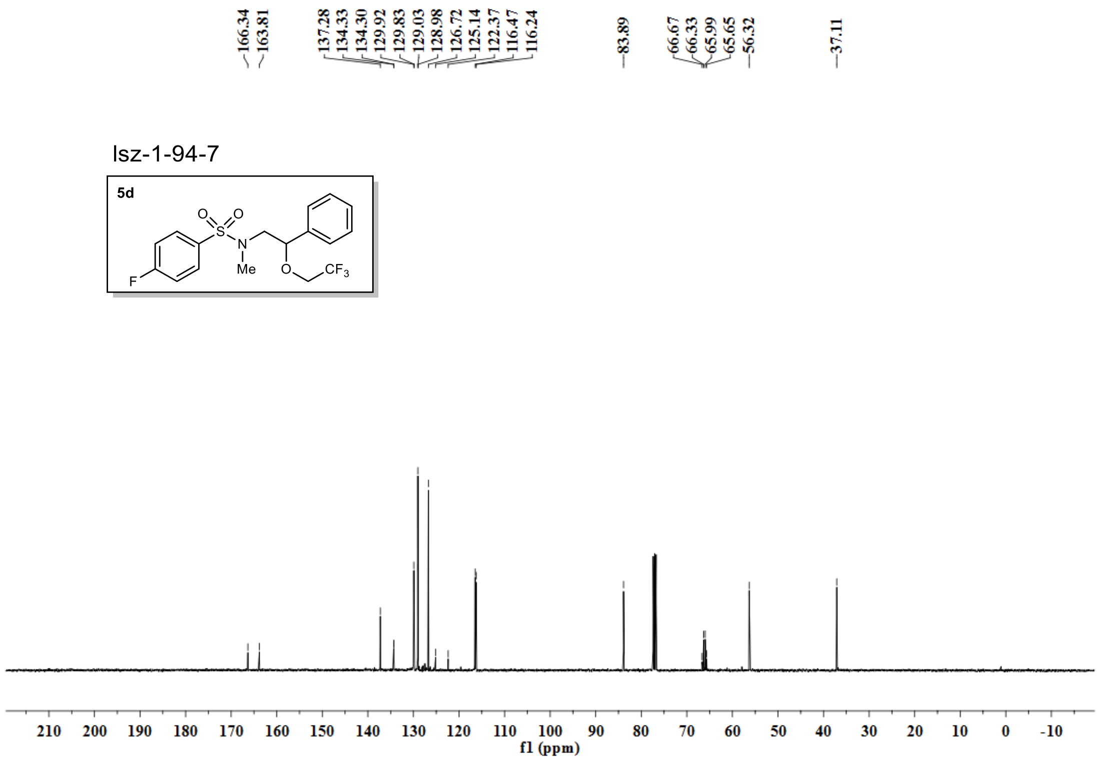

^{19}F NMR spectrum of 5d (377 MHz, $CDCl_3$):

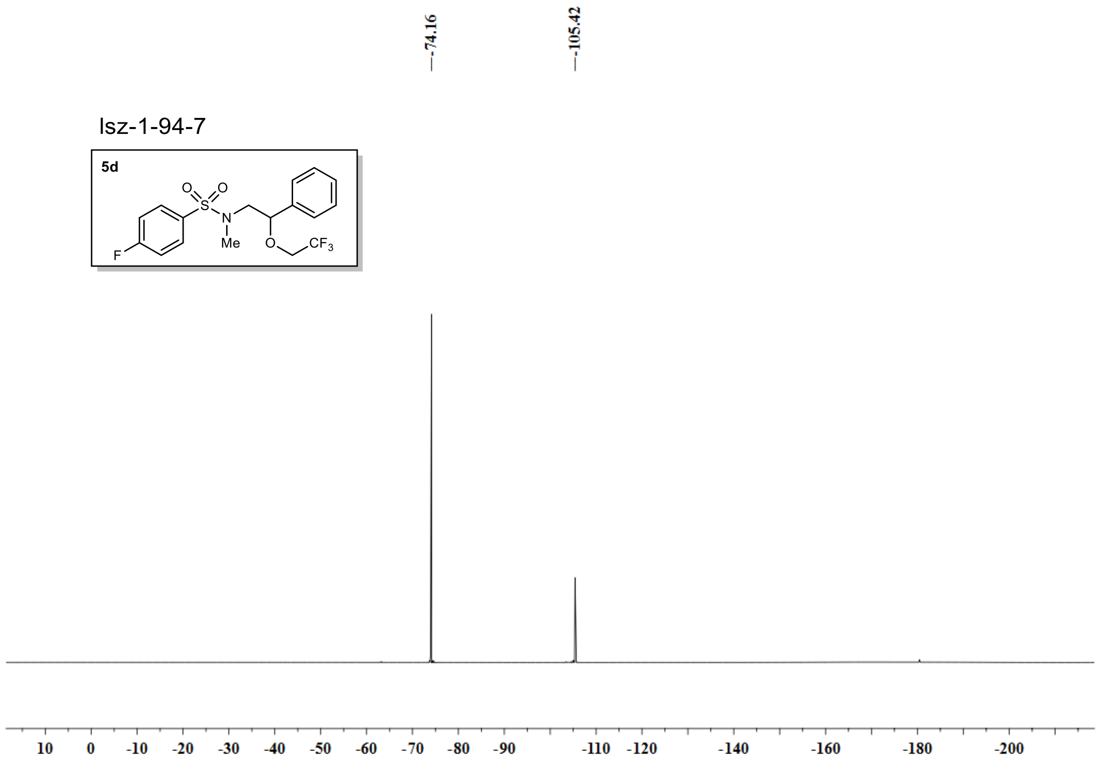

^{1}H NMR spectrum of 5e (400 MHz, $CDCl_3$):

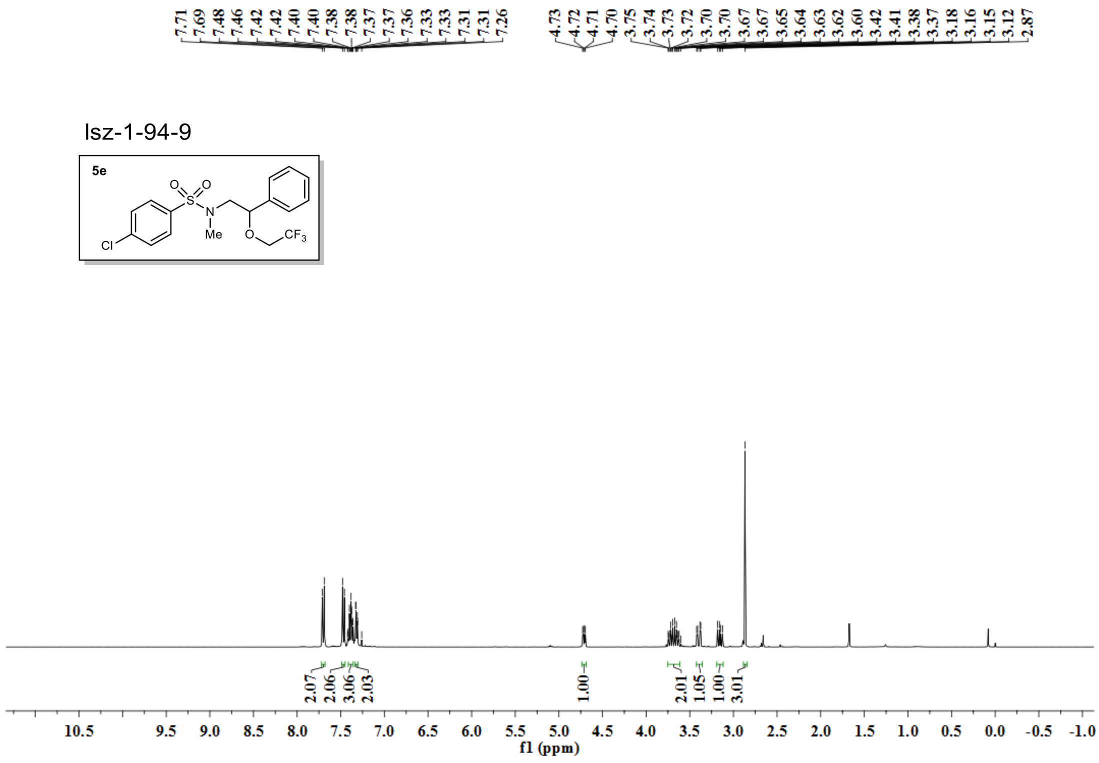

^{13}C NMR spectrum of 5e (101 MHz, $CDCl_3$):

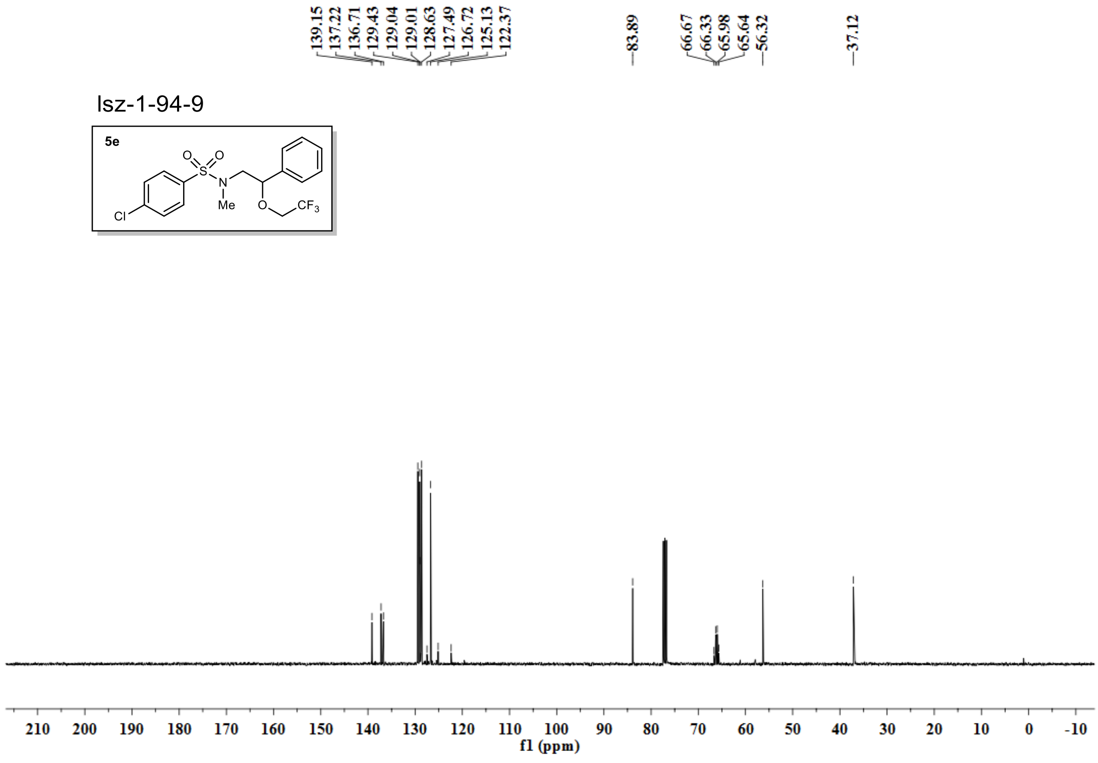

^{19}F NMR spectrum of 5e (377 MHz, $CDCl_3$):

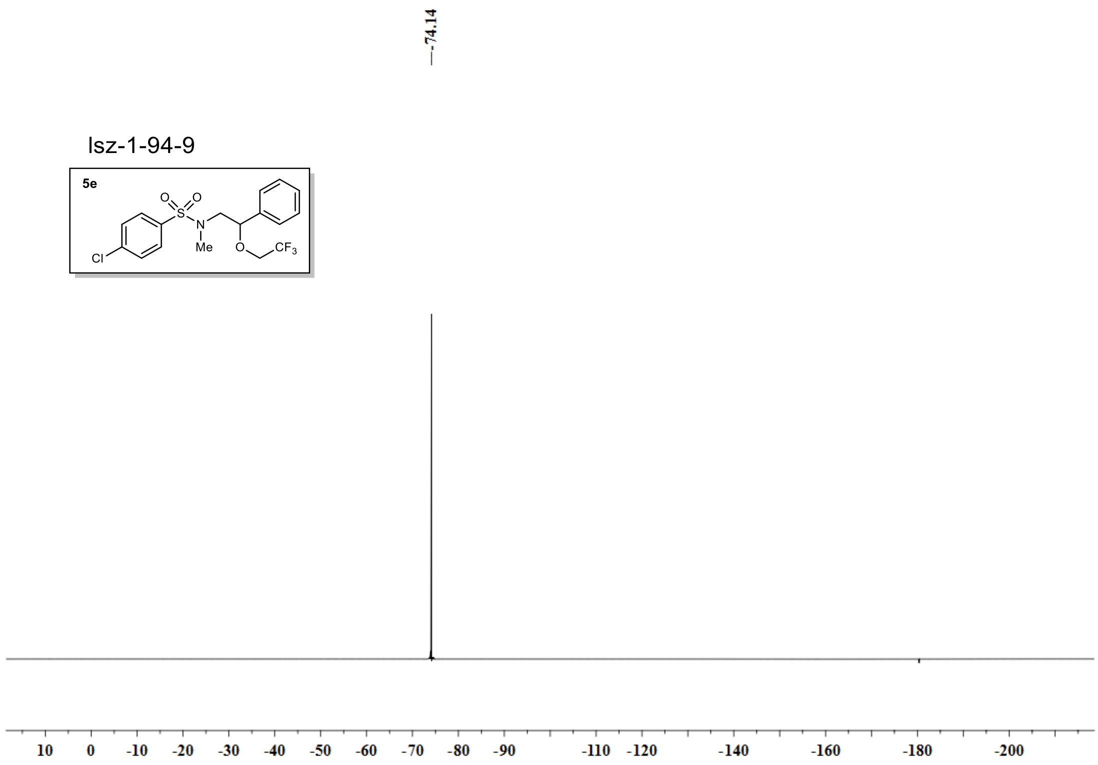

^{1}H NMR spectrum of 5f (400 MHz, $CDCl_3$):

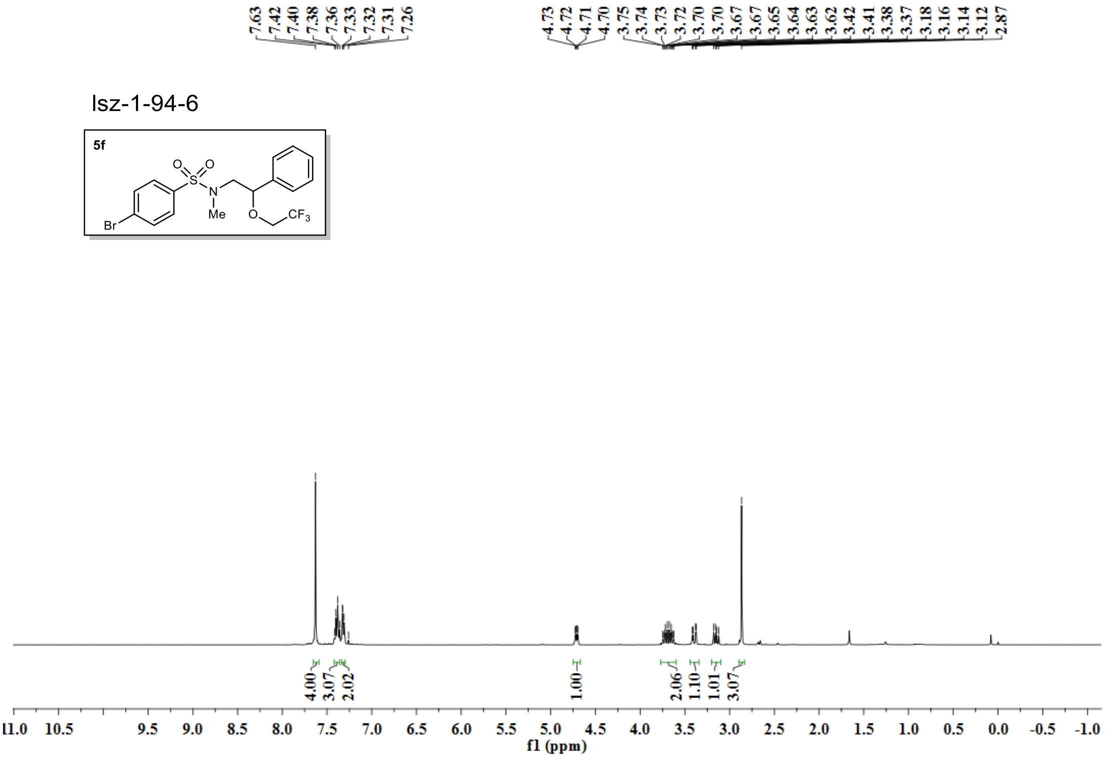

^{13}C NMR spectrum of 5f (101 MHz, $CDCl_3$):

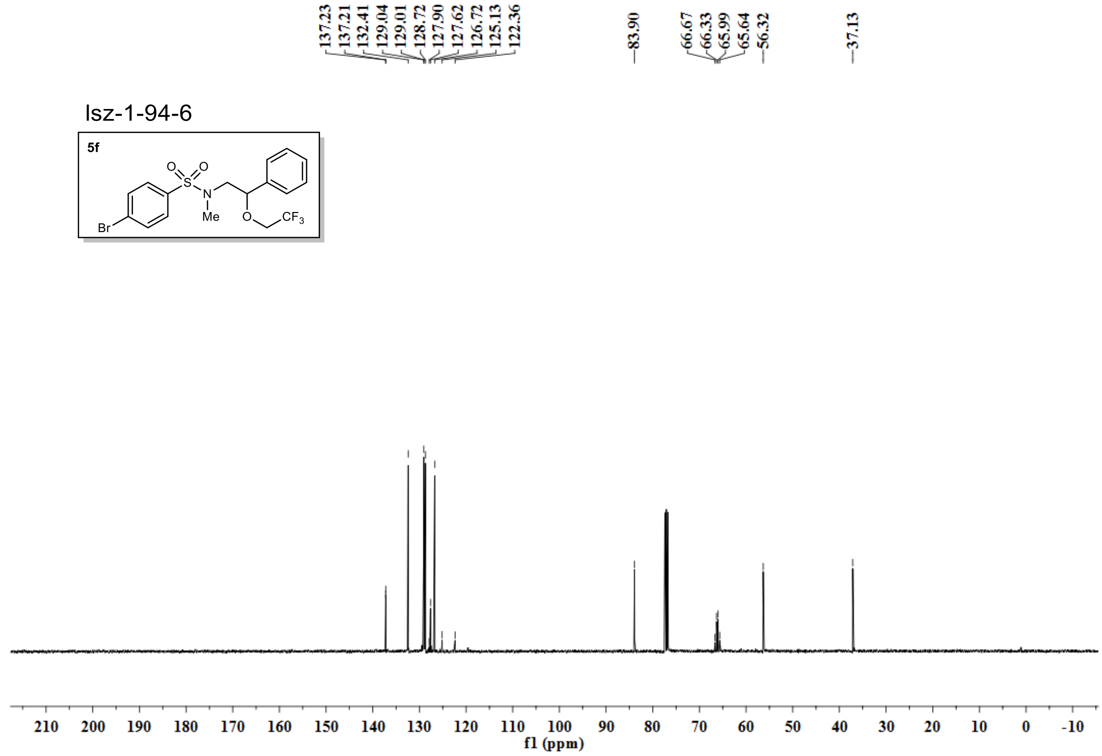

^{19}F NMR spectrum of 5f (377 MHz, $CDCl_3$):

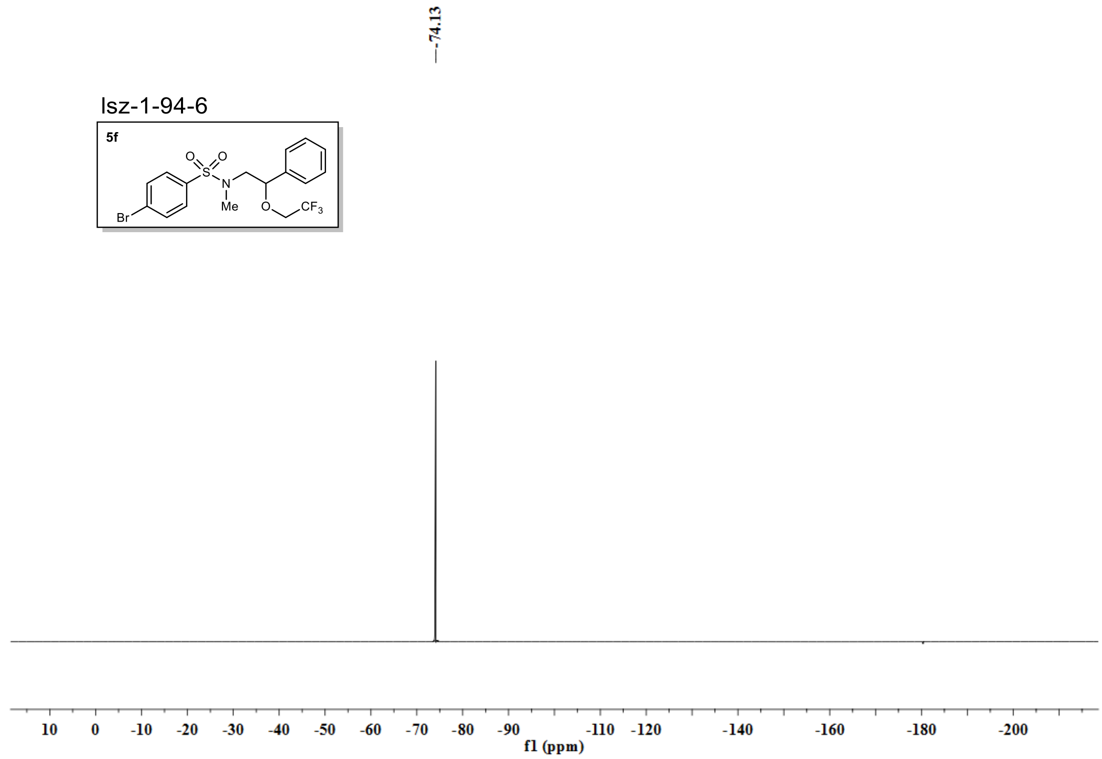

^{1}H NMR spectrum of 5g (400 MHz, $CDCl_3$):

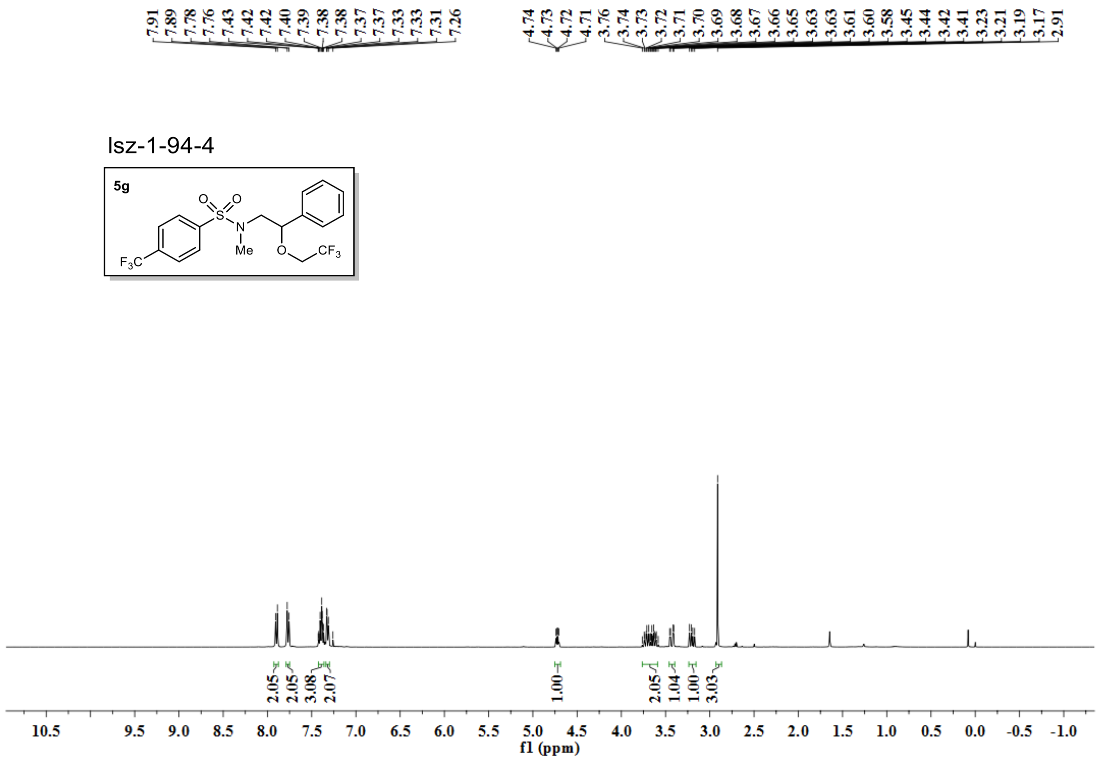

^{13}C NMR spectrum of 5g (101 MHz, $CDCl_3$):

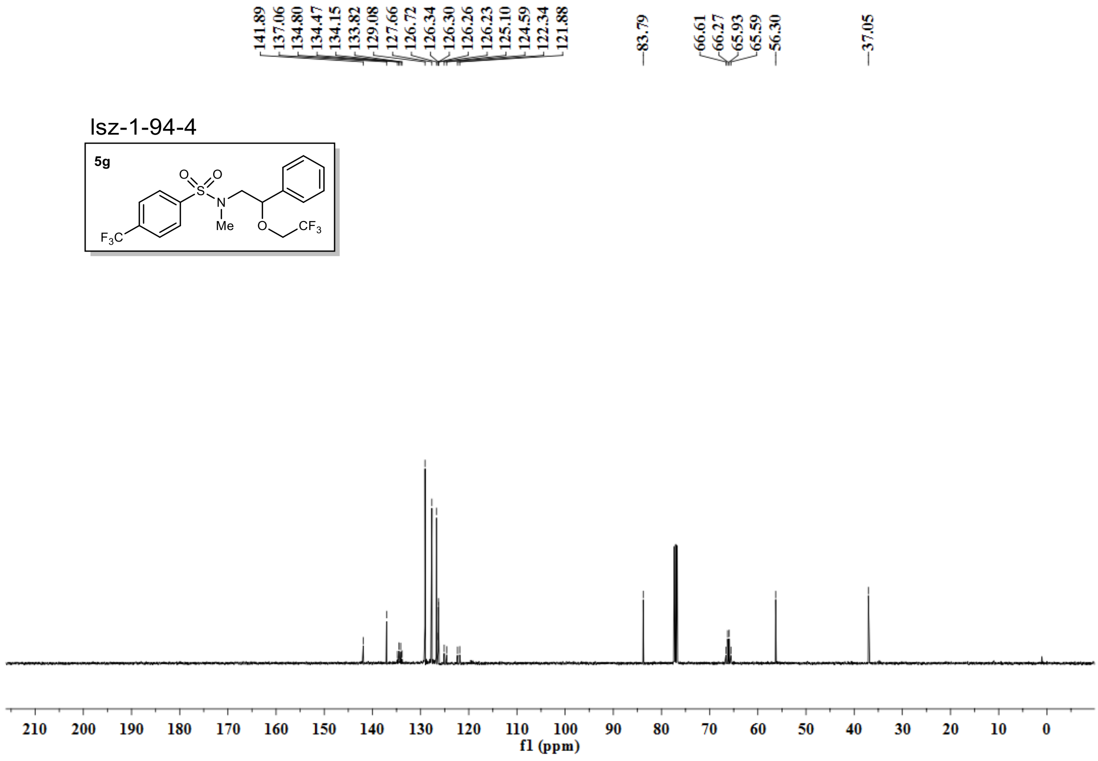

^{19}F NMR spectrum of 5g (377 MHz, $CDCl_3$):

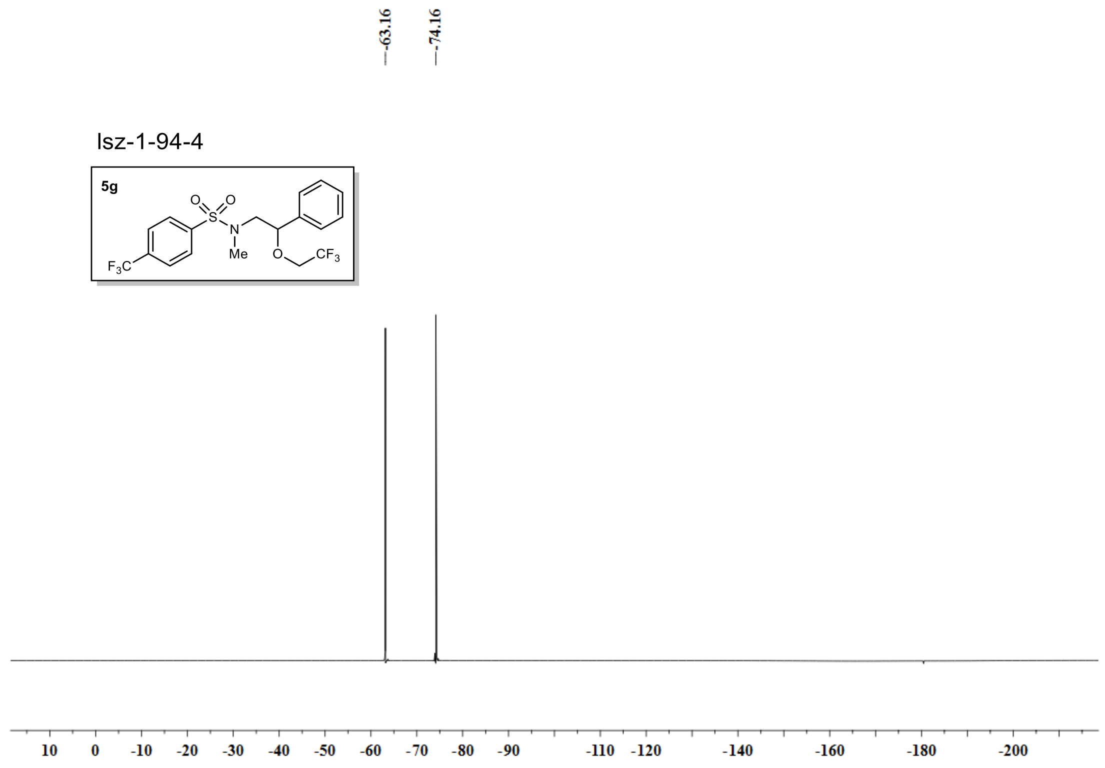

^{1}H NMR spectrum of 5h (400 MHz, $CDCl_3$):

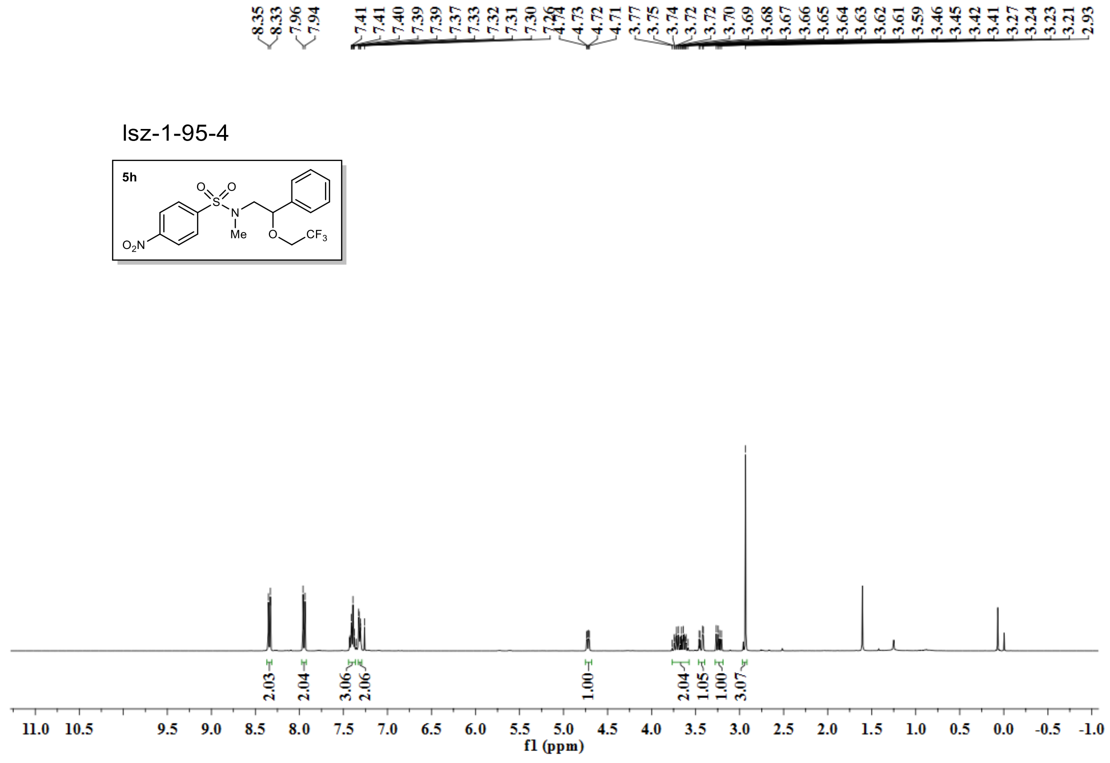

^{13}C NMR spectrum of 5h (101 MHz, $CDCl_3$):

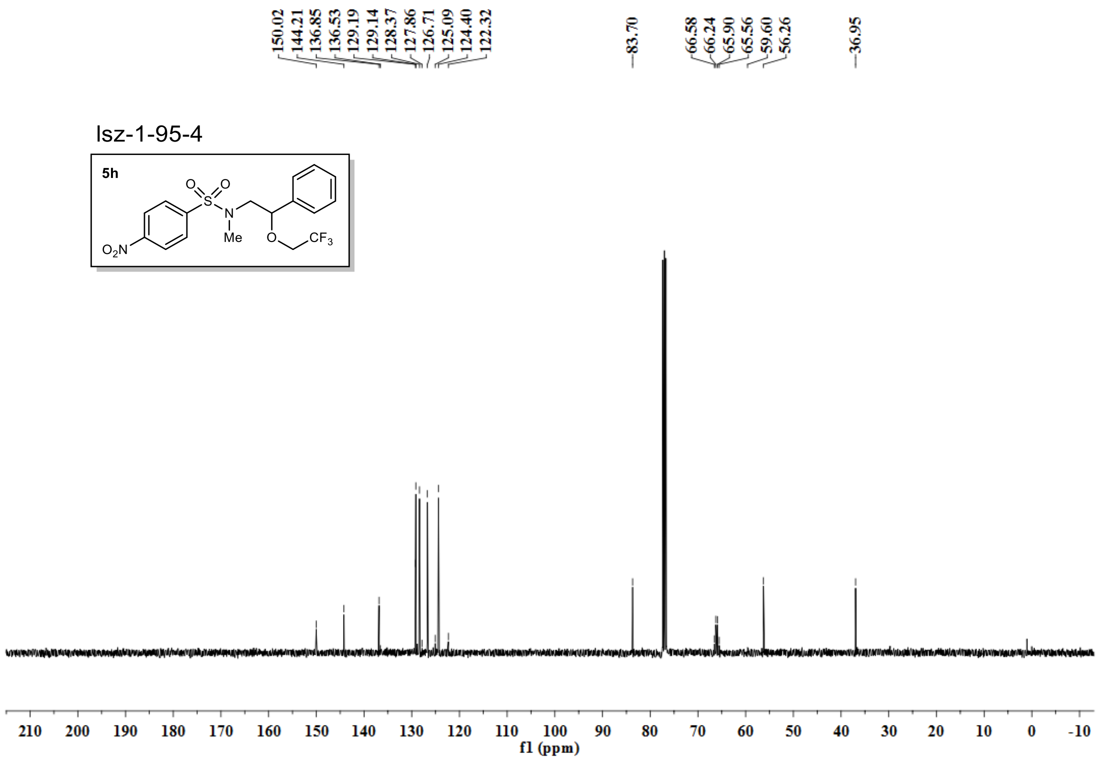

^{19}F NMR spectrum of 5h (377 MHz, $CDCl_3$):

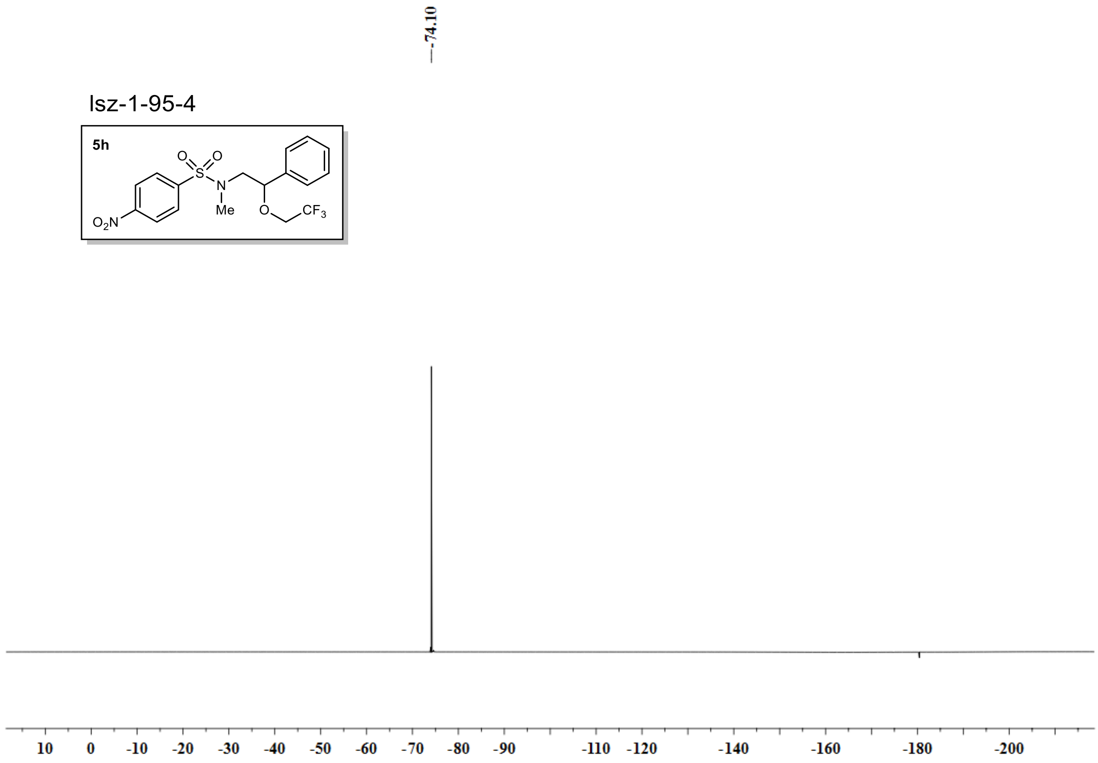

^{1}H NMR spectrum of 5i (400 MHz, $CDCl_3$):

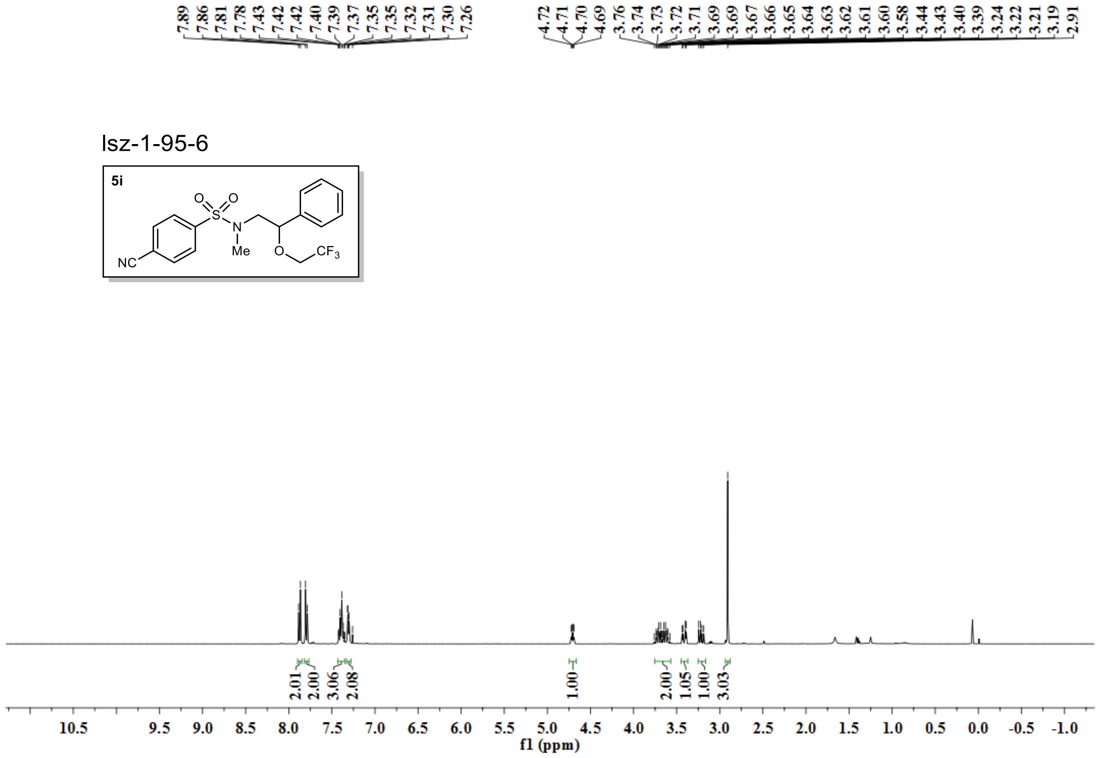

^{13}C NMR spectrum of 5i (101 MHz, $CDCl_3$):

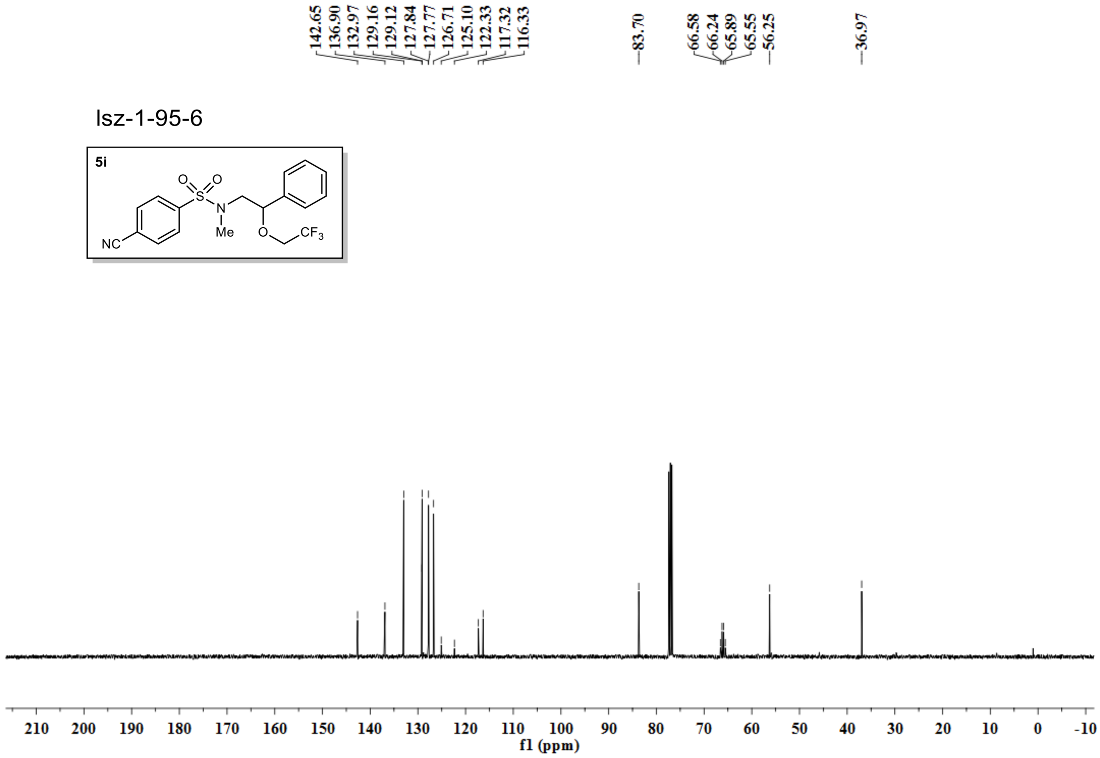

^{19}**F NMR spectrum of 5i** (377 MHz, $CDCl_3$):

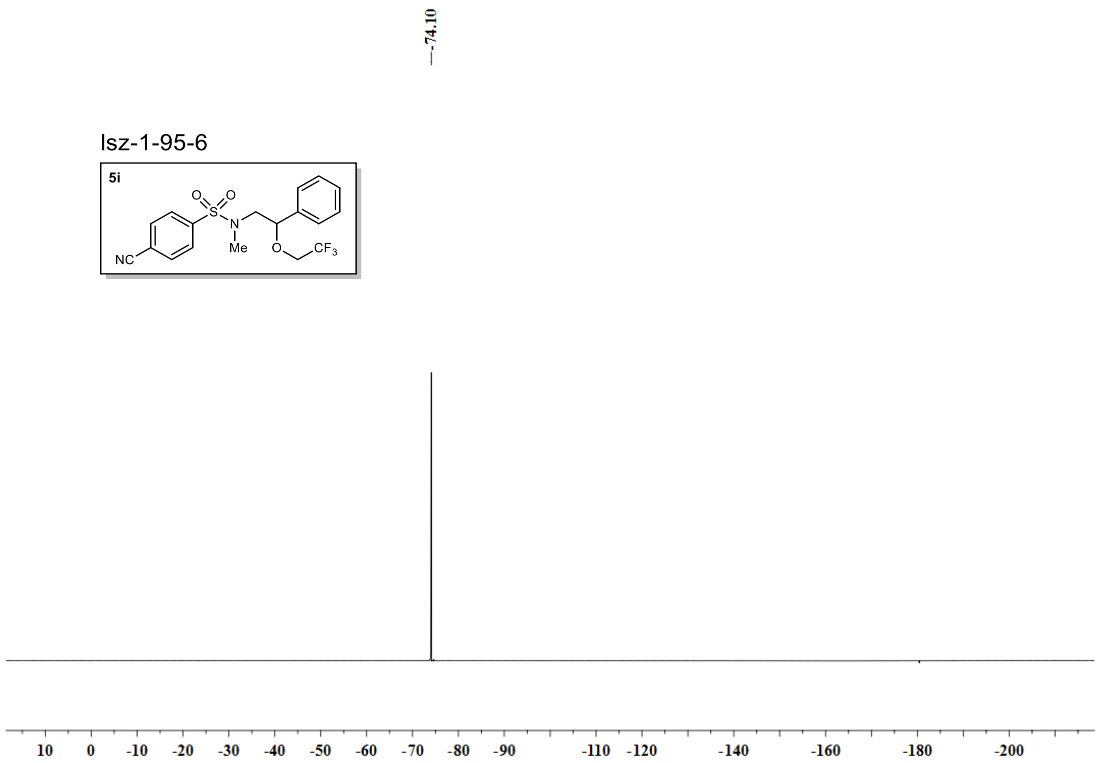

^{1}**H NMR spectrum of 5j** (400 MHz, $CDCl_3$):

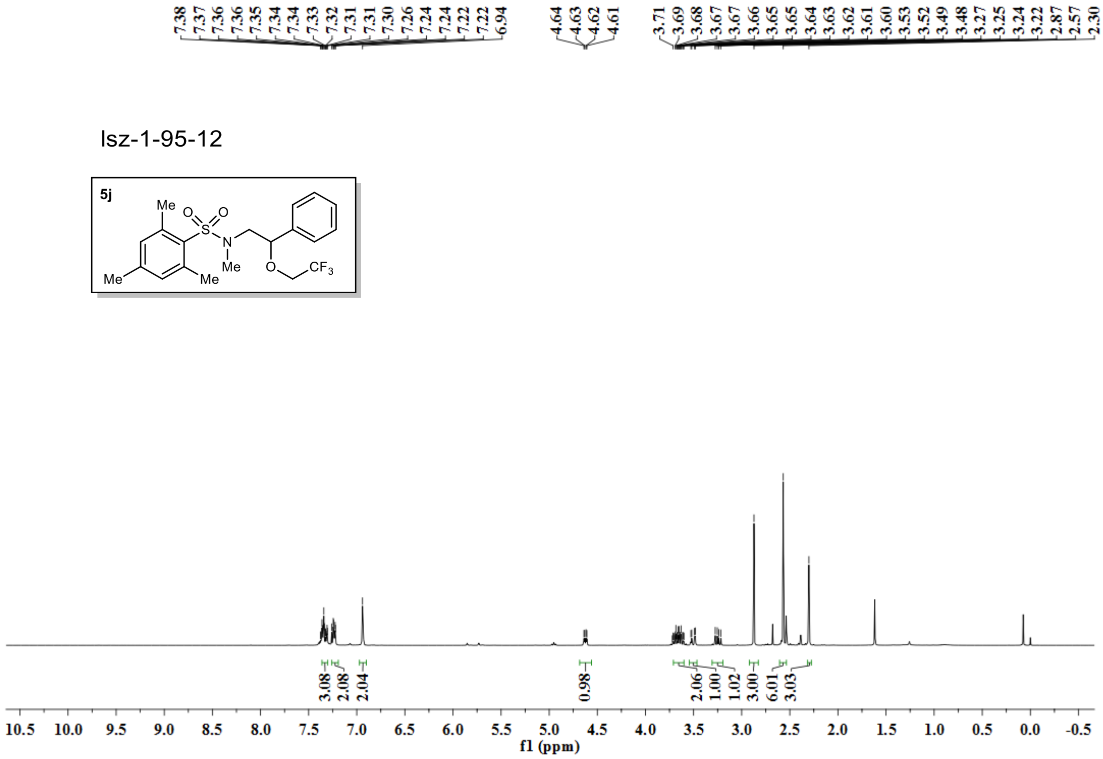

^{13}C NMR spectrum of 5j (101 MHz, $CDCl_3$):

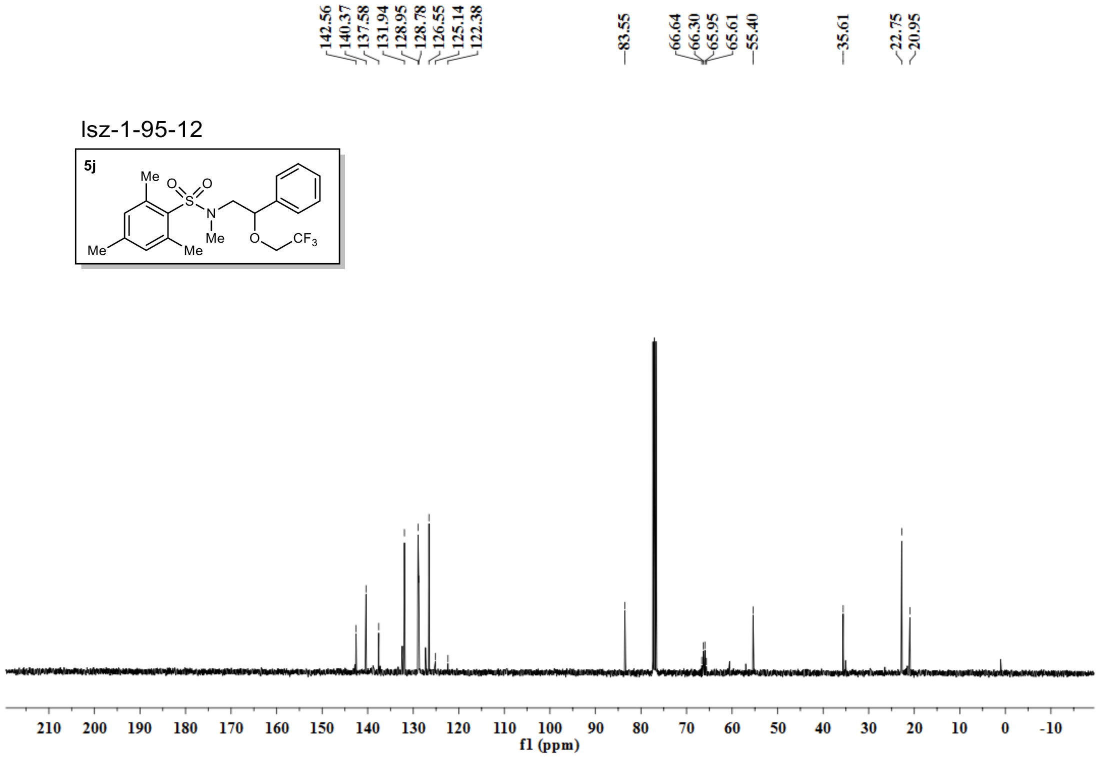

^{19}F NMR spectrum of 5j (377 MHz, $CDCl_3$):

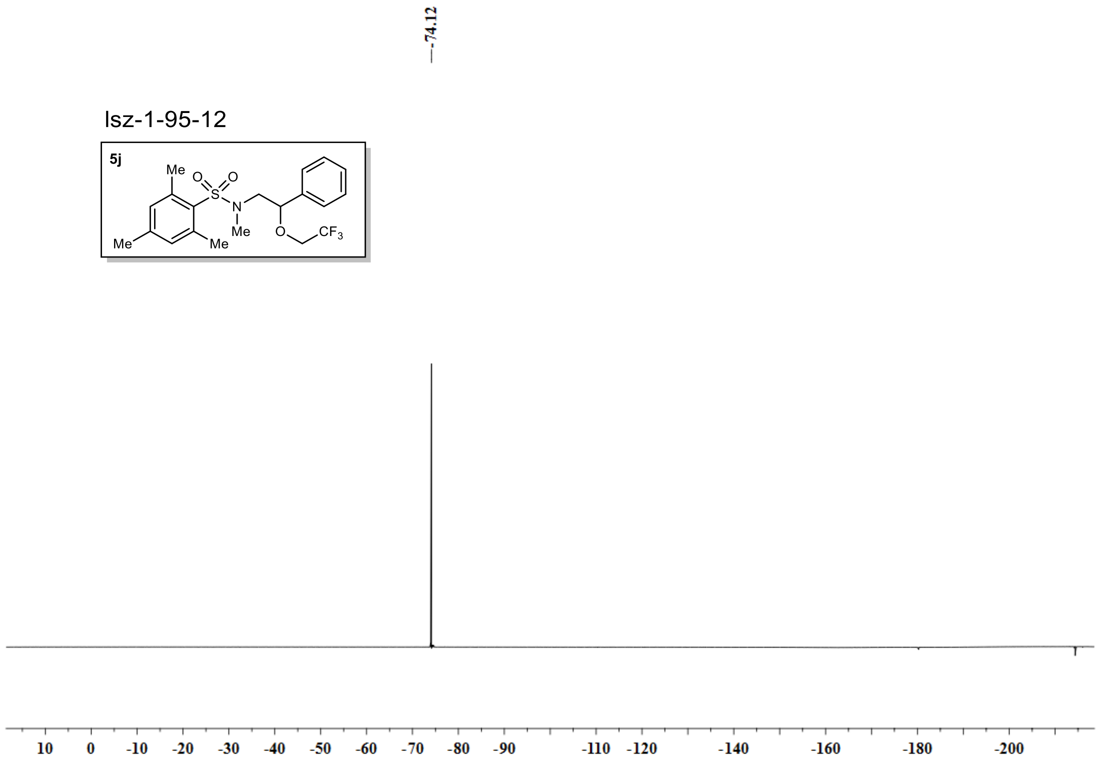

^{1}H NMR spectrum of 5k (400 MHz, $CDCl_3$):

lsz-1-95-5

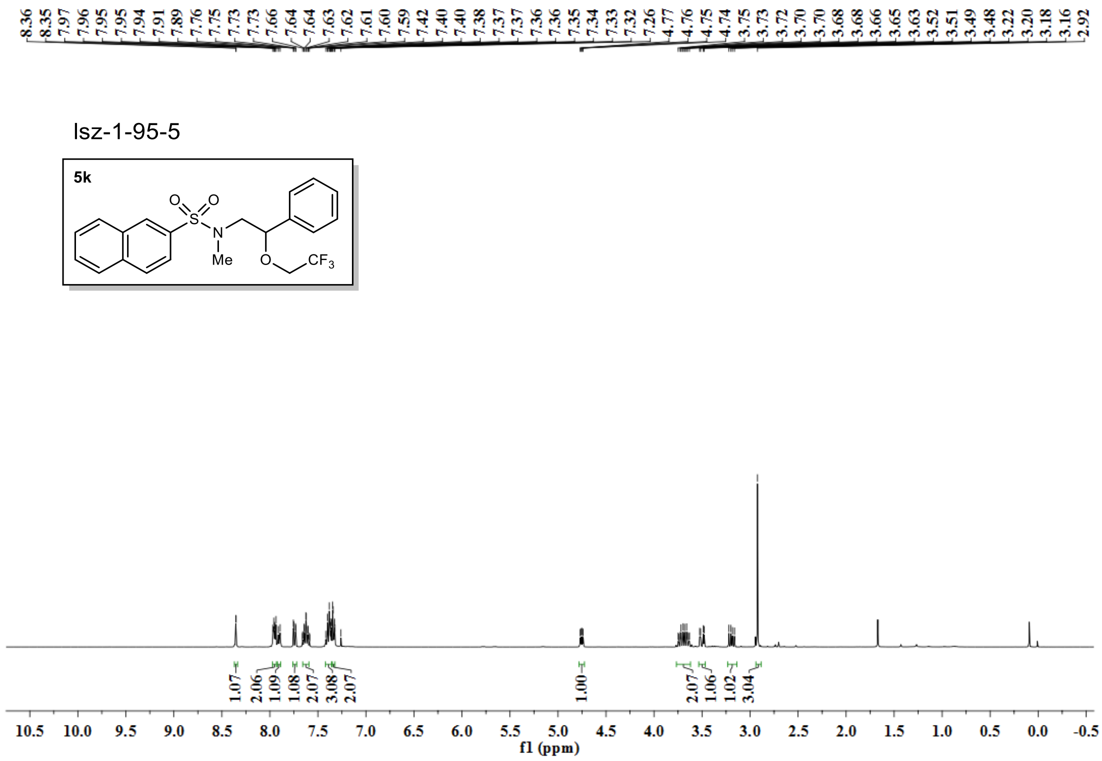

^{13}C NMR spectrum of 5k (101 MHz, $CDCl_3$):

lsz-1-95-5

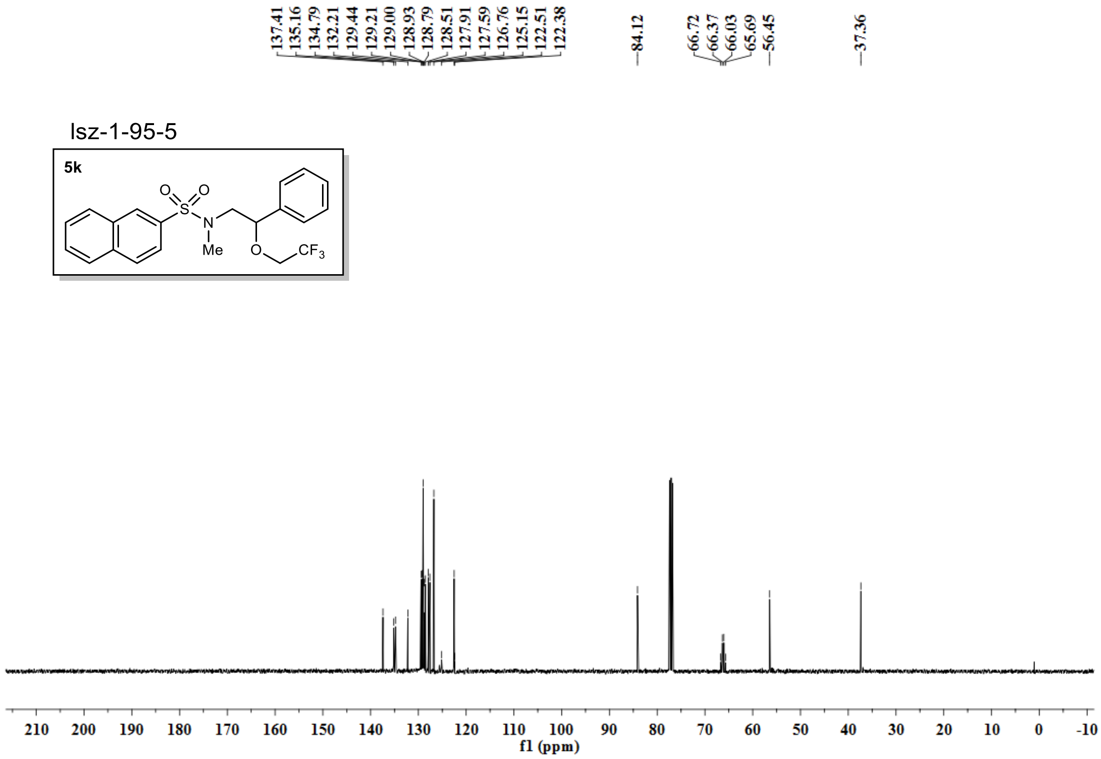

^{19}F NMR spectrum of 5k (377 MHz, $CDCl_3$):

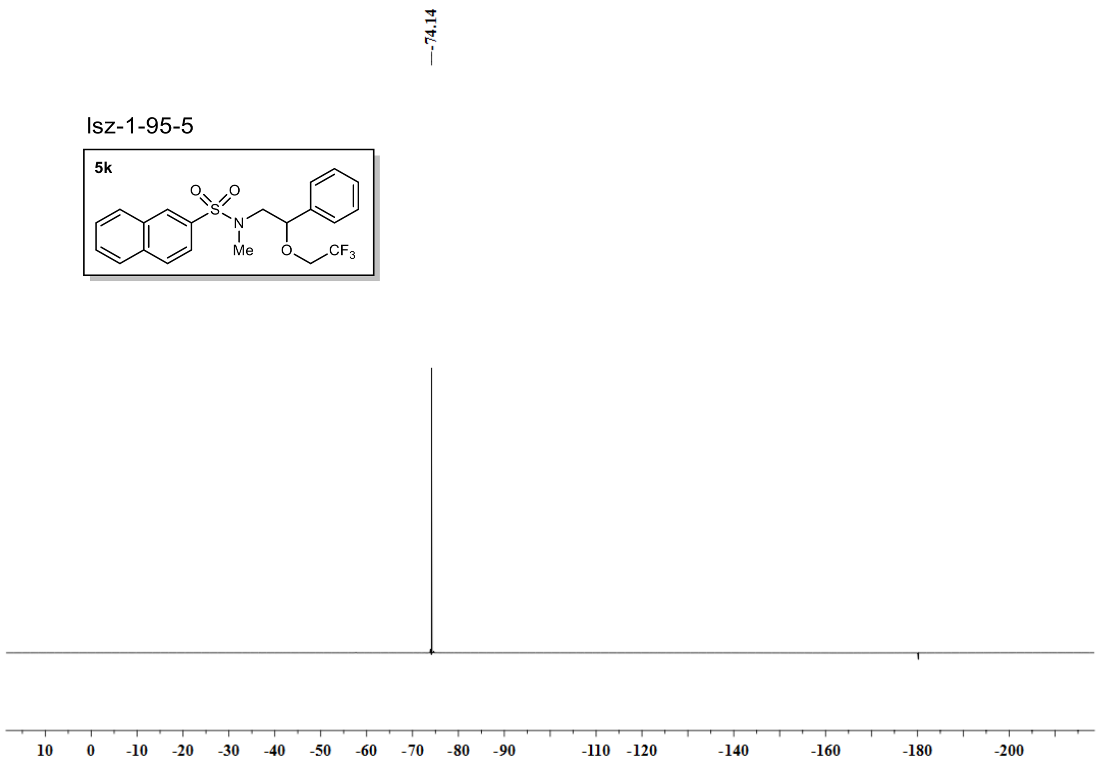

^{1}H NMR spectrum of 5l (400 MHz, $CDCl_3$):

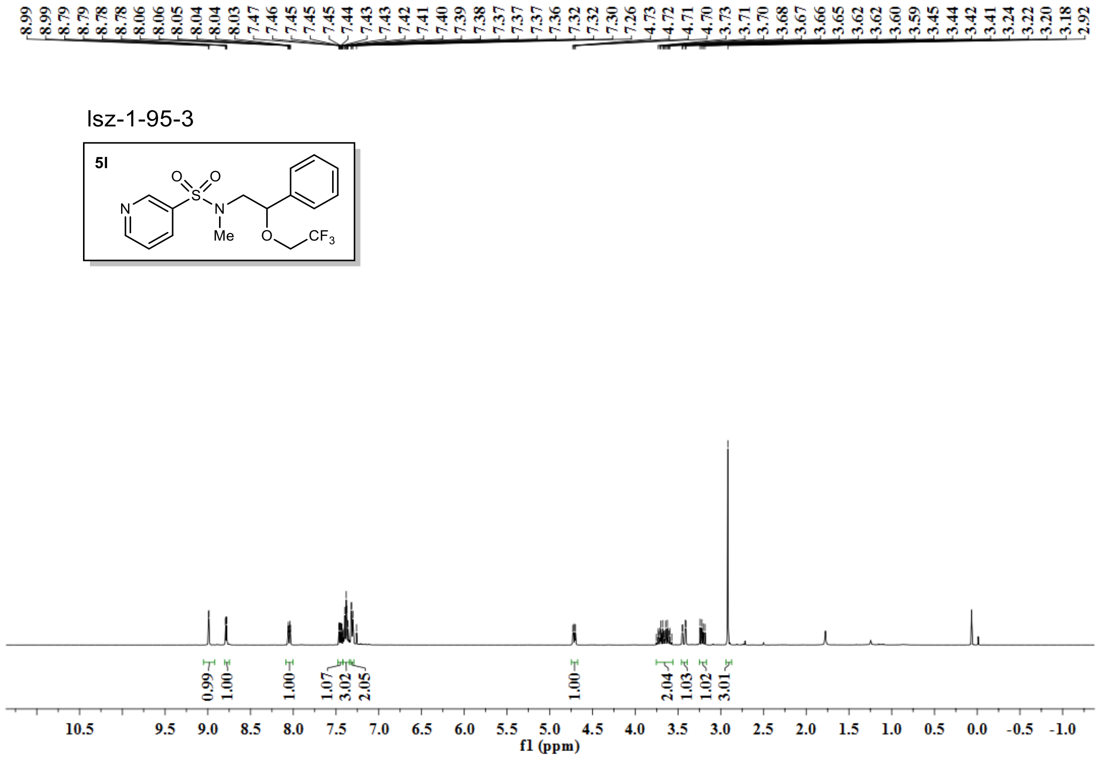

^{13}C NMR spectrum of 5l (101 MHz, $CDCl_3$):

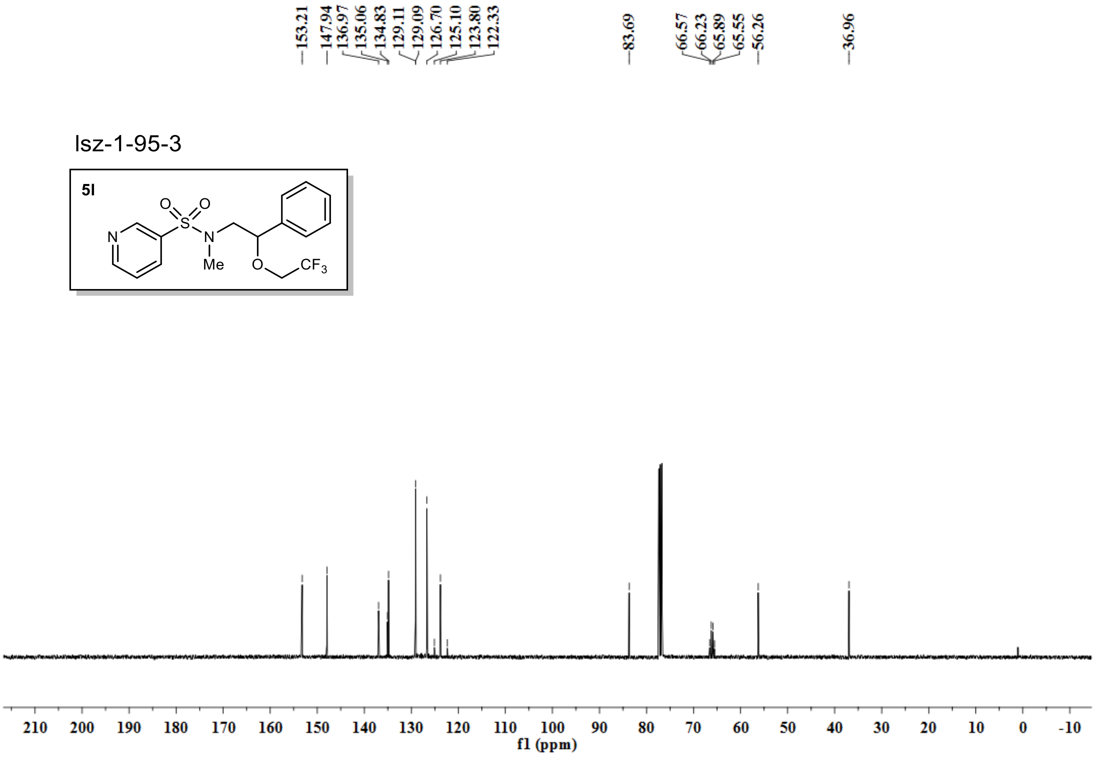

^{19}F NMR spectrum of 5l (377 MHz, $CDCl_3$):

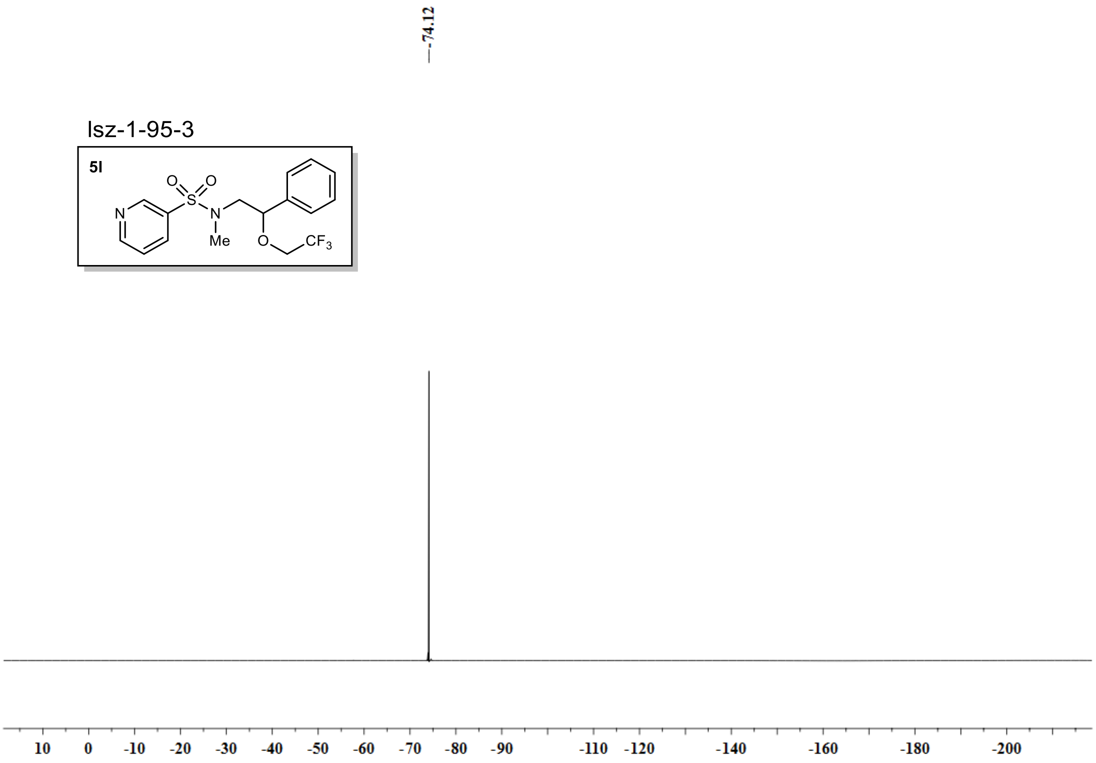

^{1}H NMR spectrum of 5m (400 MHz, $CDCl_3$):

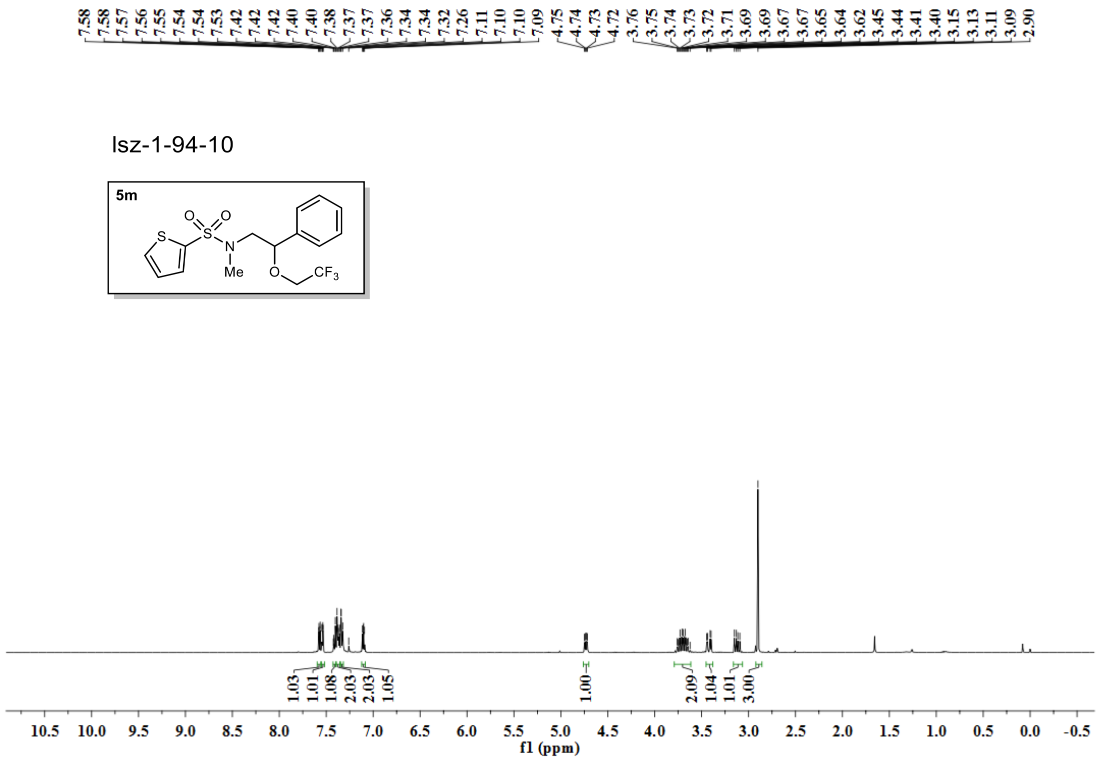

^{13}C NMR spectrum of 5m (101 MHz, $CDCl_3$):

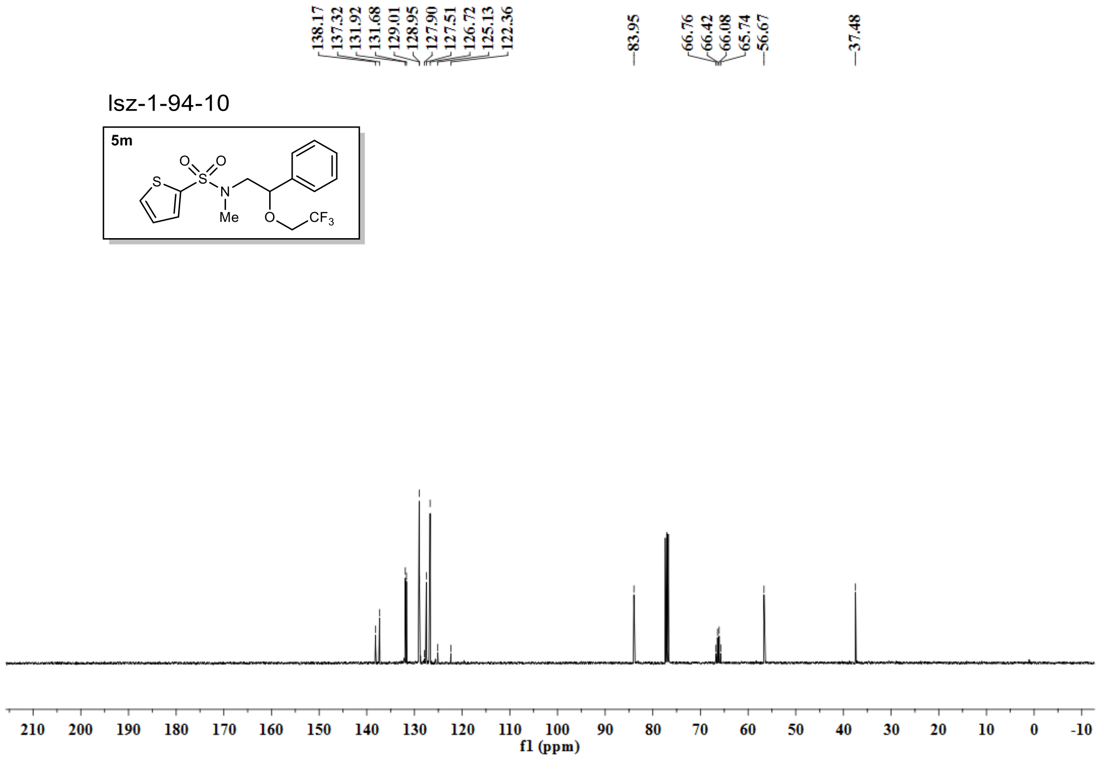

^{19}F NMR spectrum of 5m (377 MHz, $CDCl_3$):

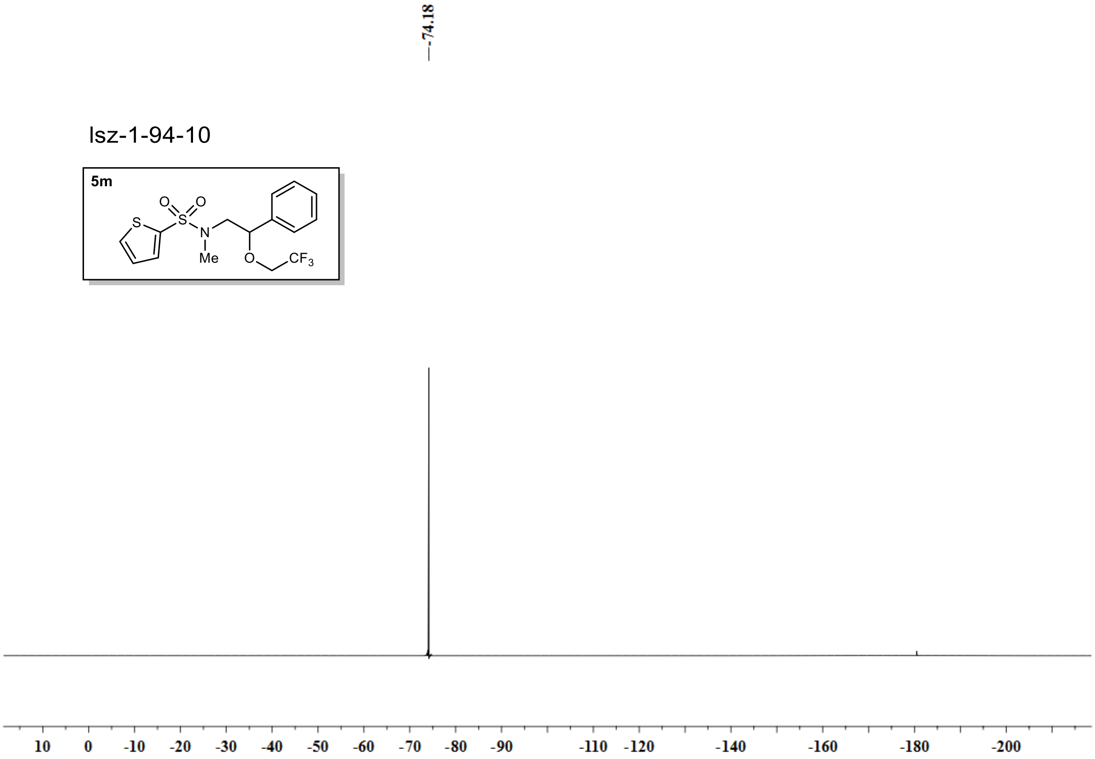

^{1}H NMR spectrum of 5n (400 MHz, $CDCl_3$):

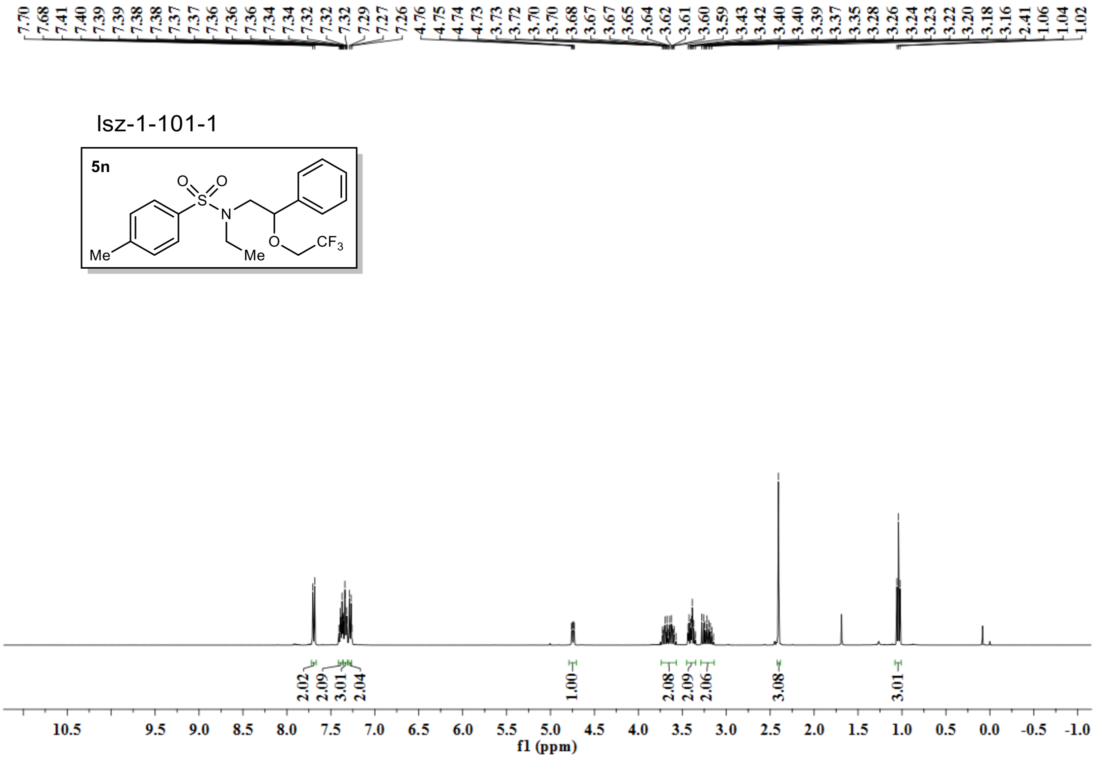

^{13}C NMR spectrum of 5n (101 MHz, $CDCl_3$):

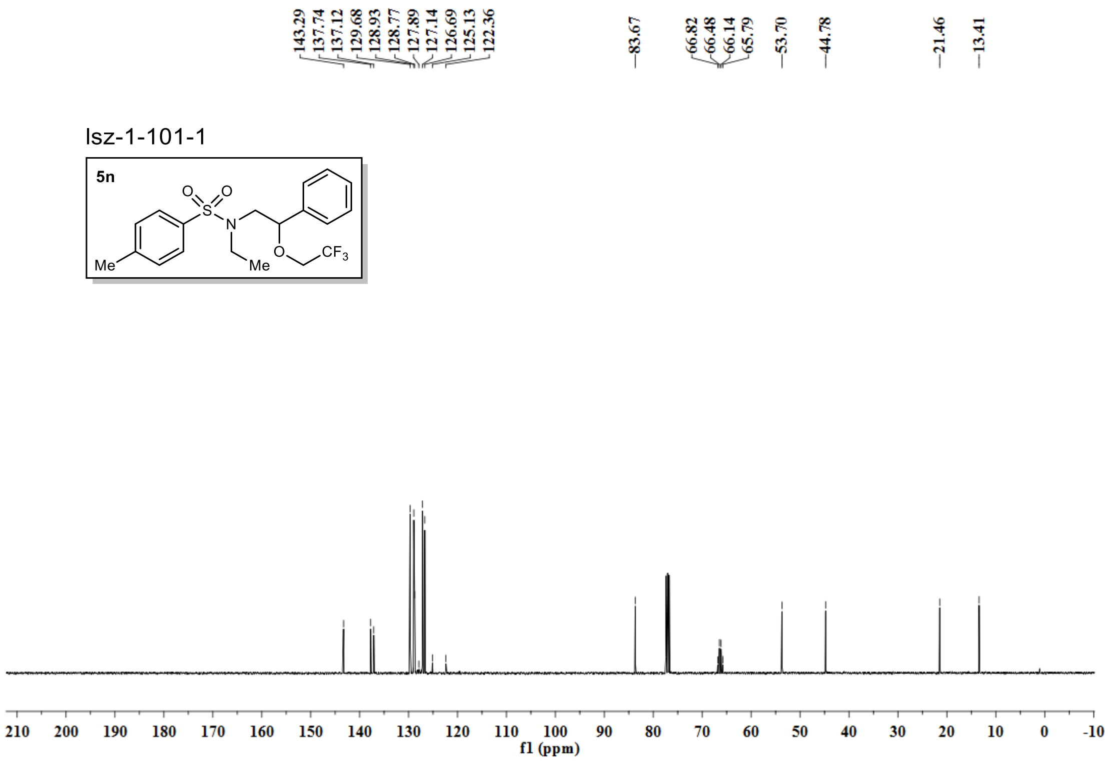

^{19}F NMR spectrum of 5n (377 MHz, $CDCl_3$):

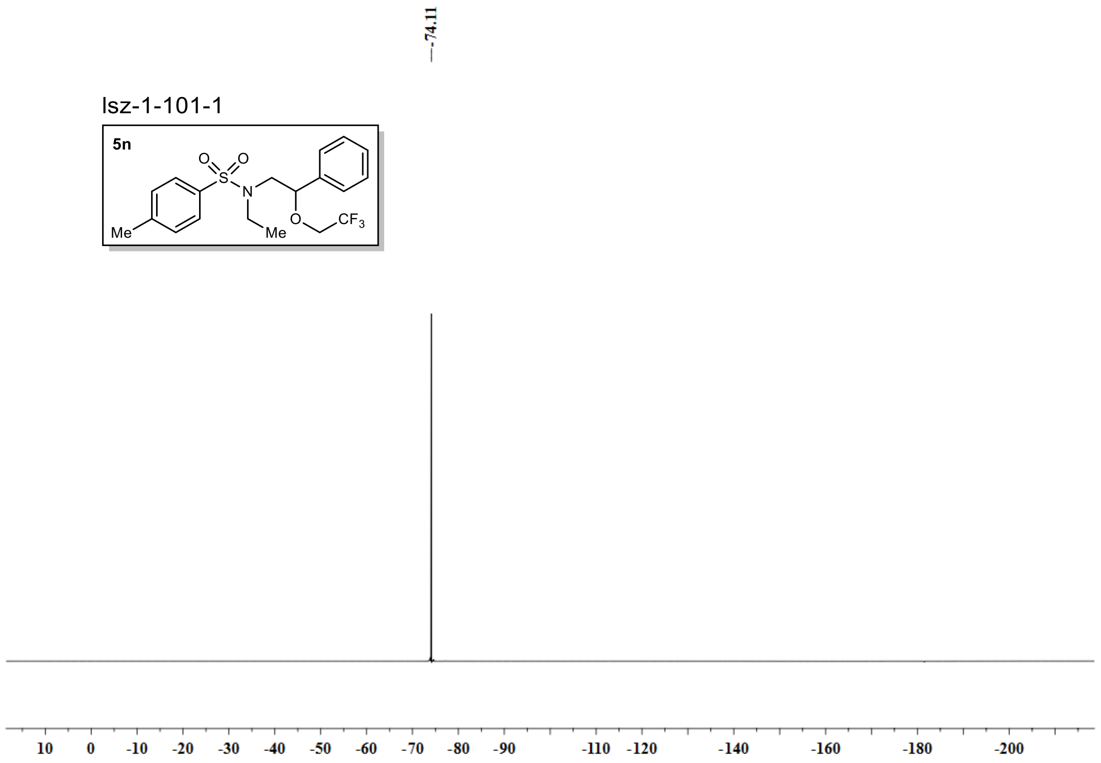

^{1}H NMR spectrum of 5o (400 MHz, $CDCl_3$):

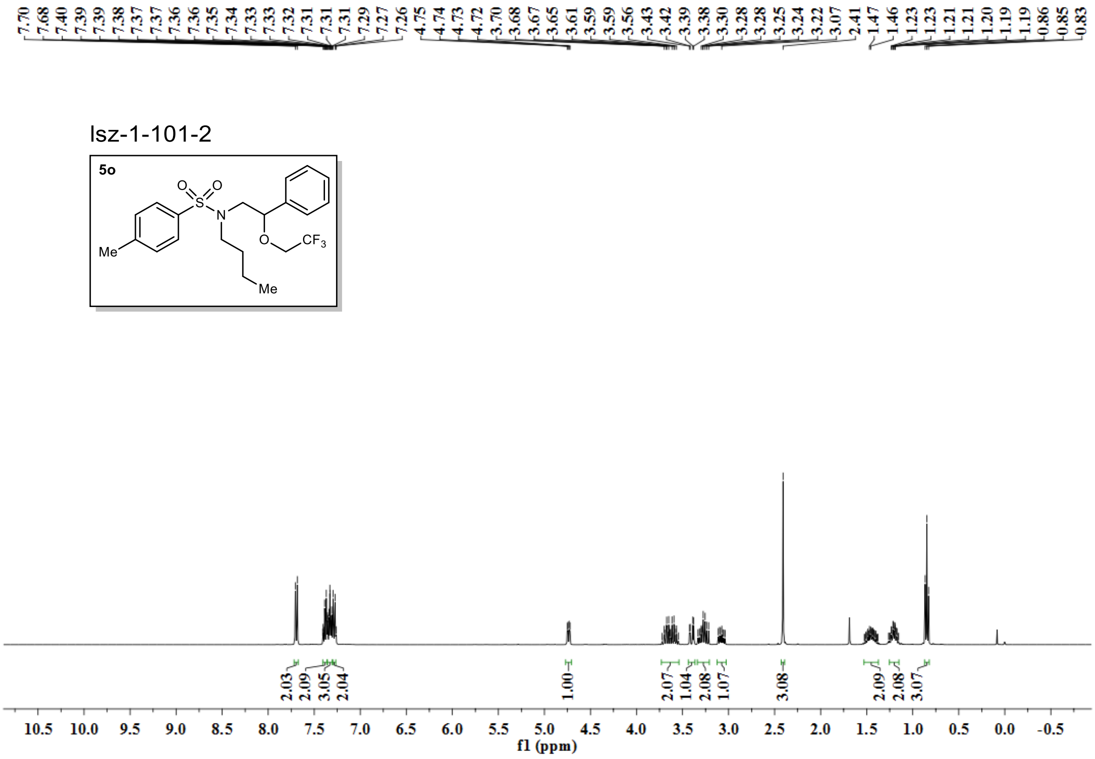

^{13}C NMR spectrum of 5o (101 MHz, $CDCl_3$):

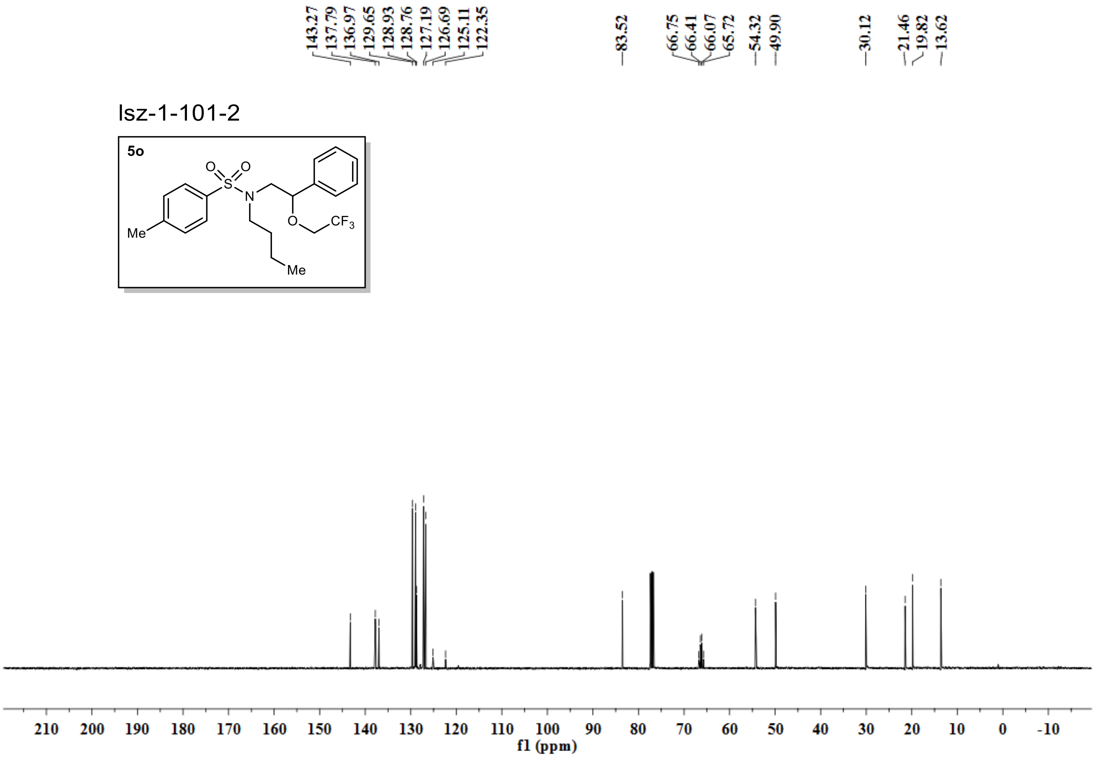

^{19}F NMR spectrum of 5o (377 MHz, $CDCl_3$):

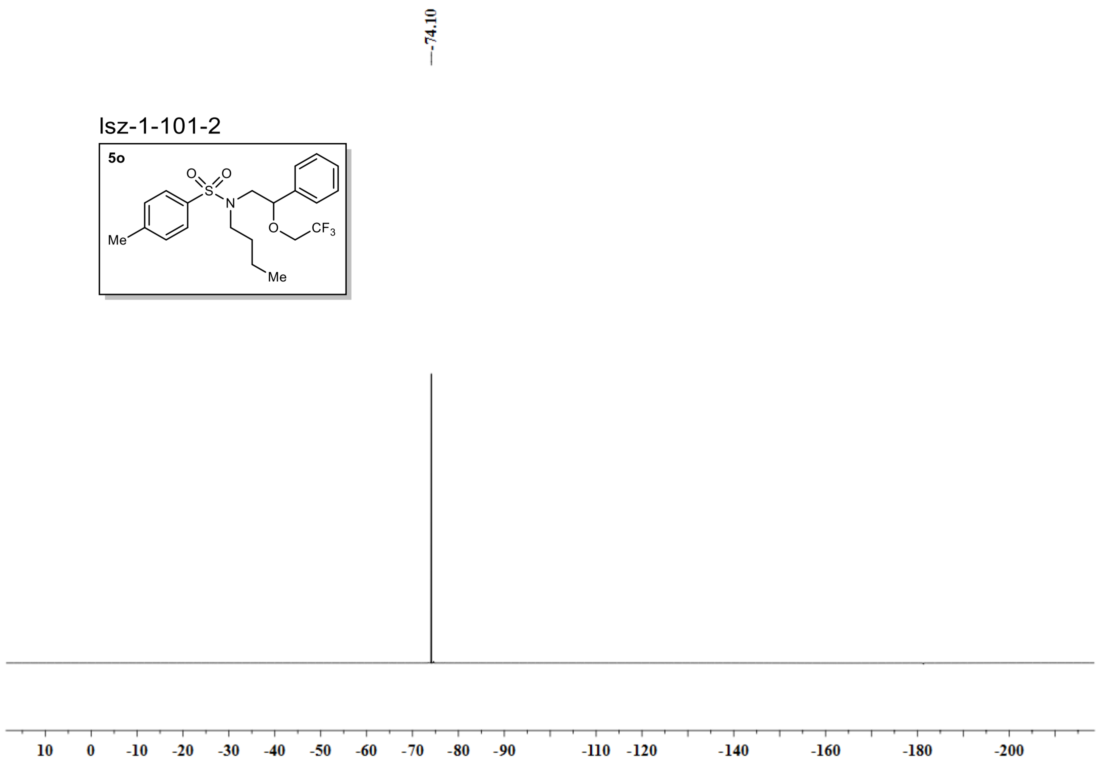

^{1}H NMR spectrum of 5p (400 MHz, $CDCl_3$):

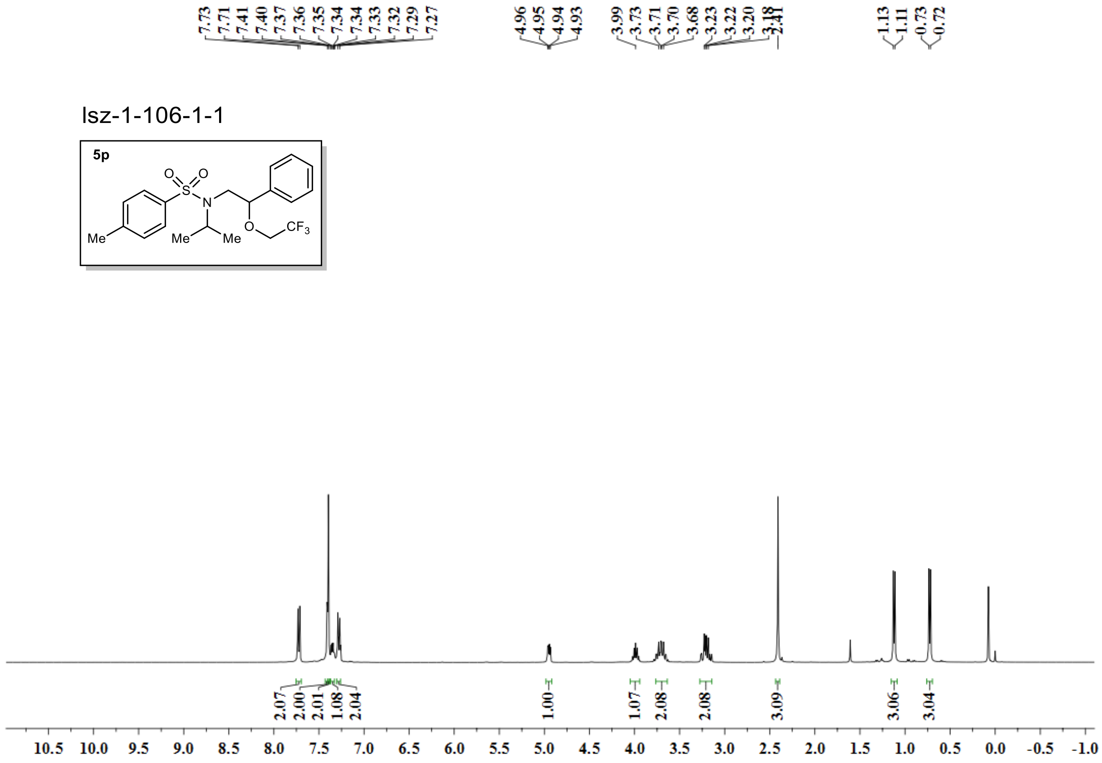

^{13}**C NMR spectrum of 5p** (101 MHz, $CDCl_3$):

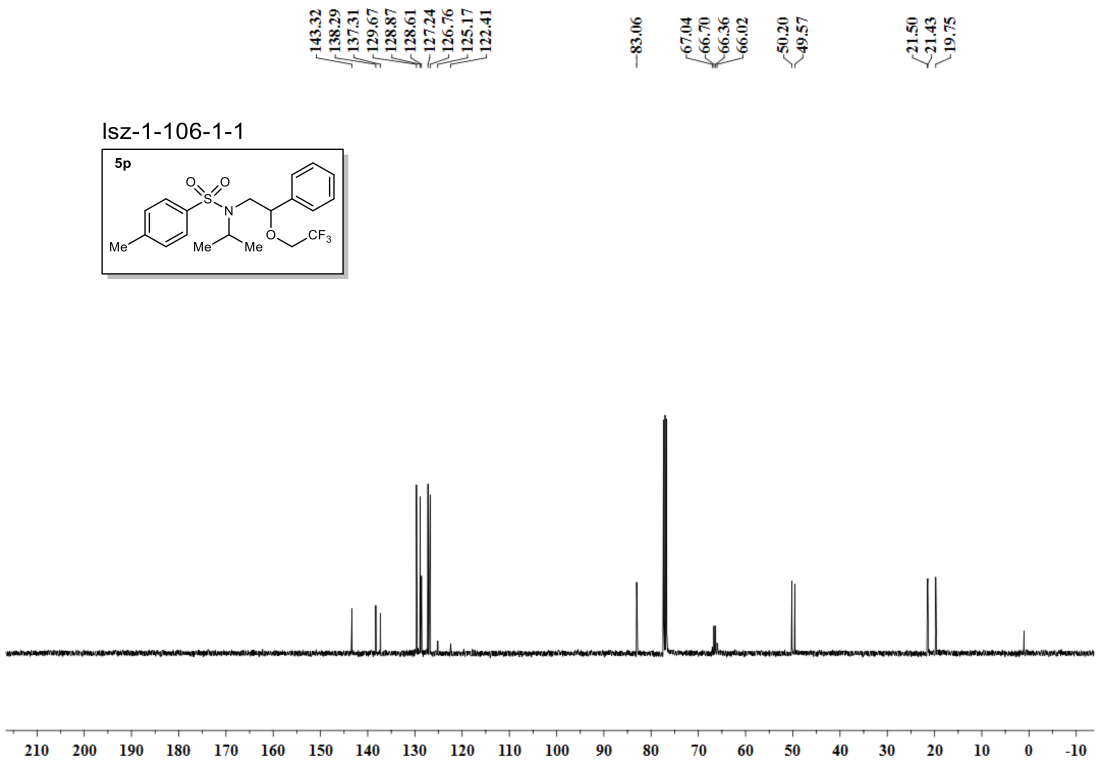

^{19}**F NMR spectrum of 5p** (377 MHz, $CDCl_3$):

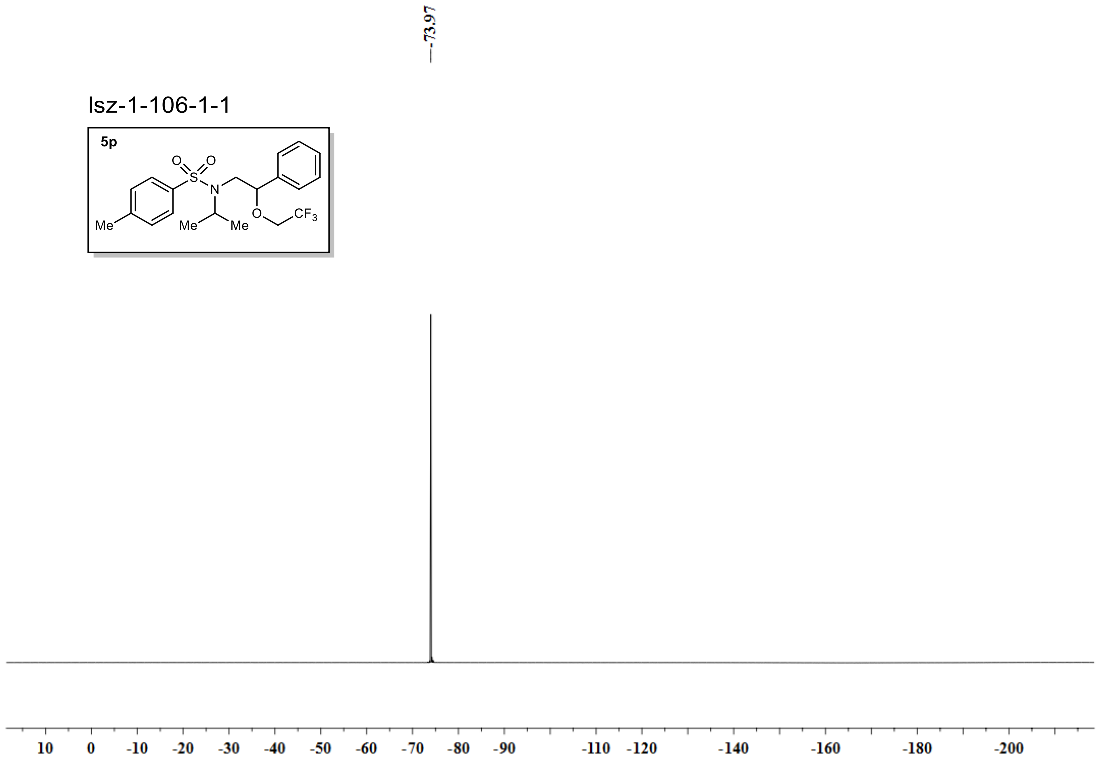

^{1}H NMR spectrum of 5q (400 MHz, $CDCl_3$):

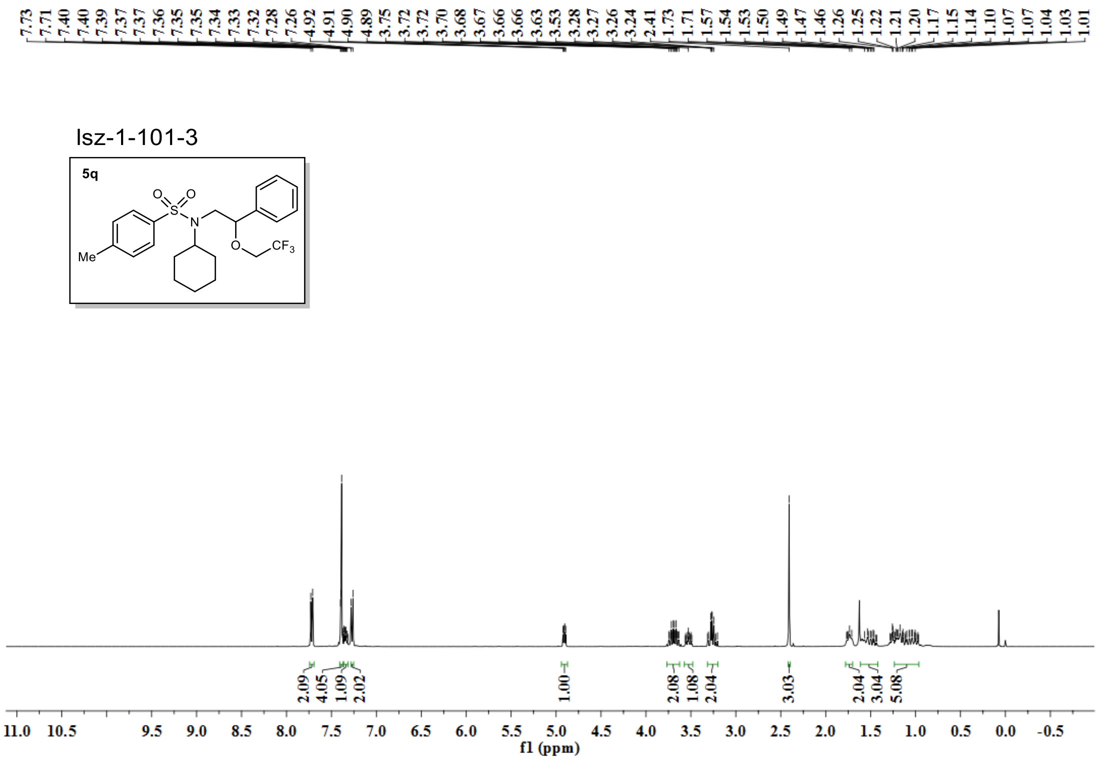

^{13}C NMR spectrum of 5q (101 MHz, $CDCl_3$):

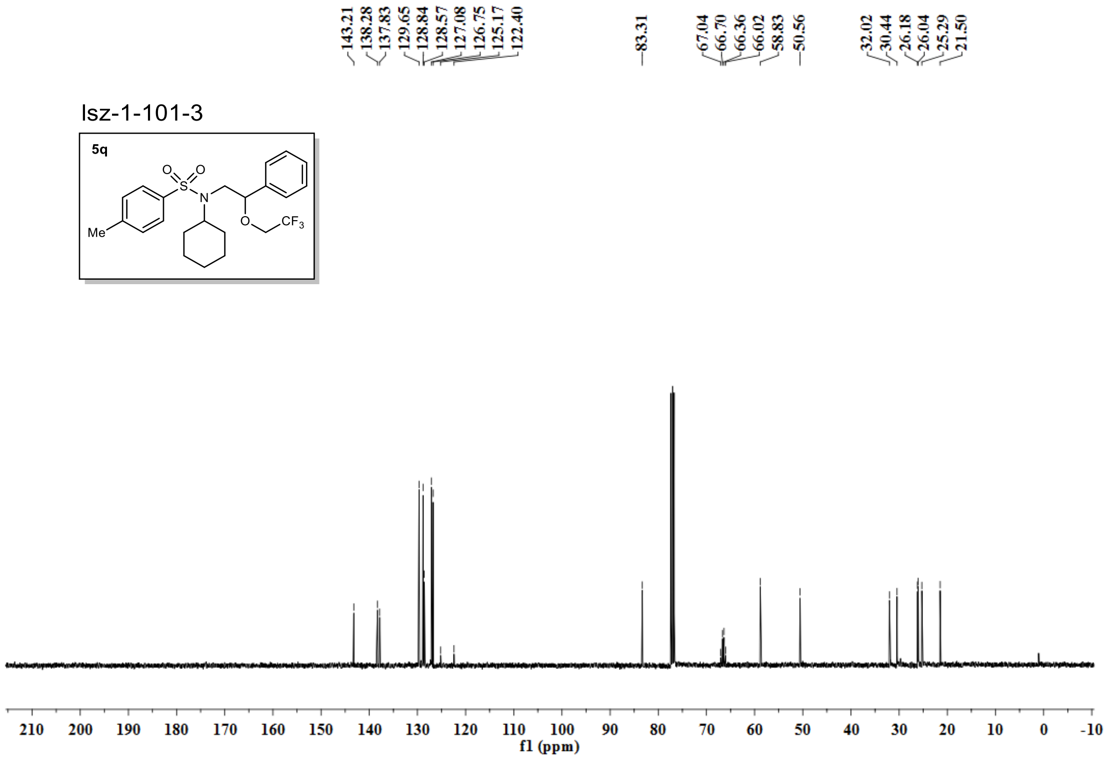

^{19}F NMR spectrum of 5q (377 MHz, $CDCl_3$):

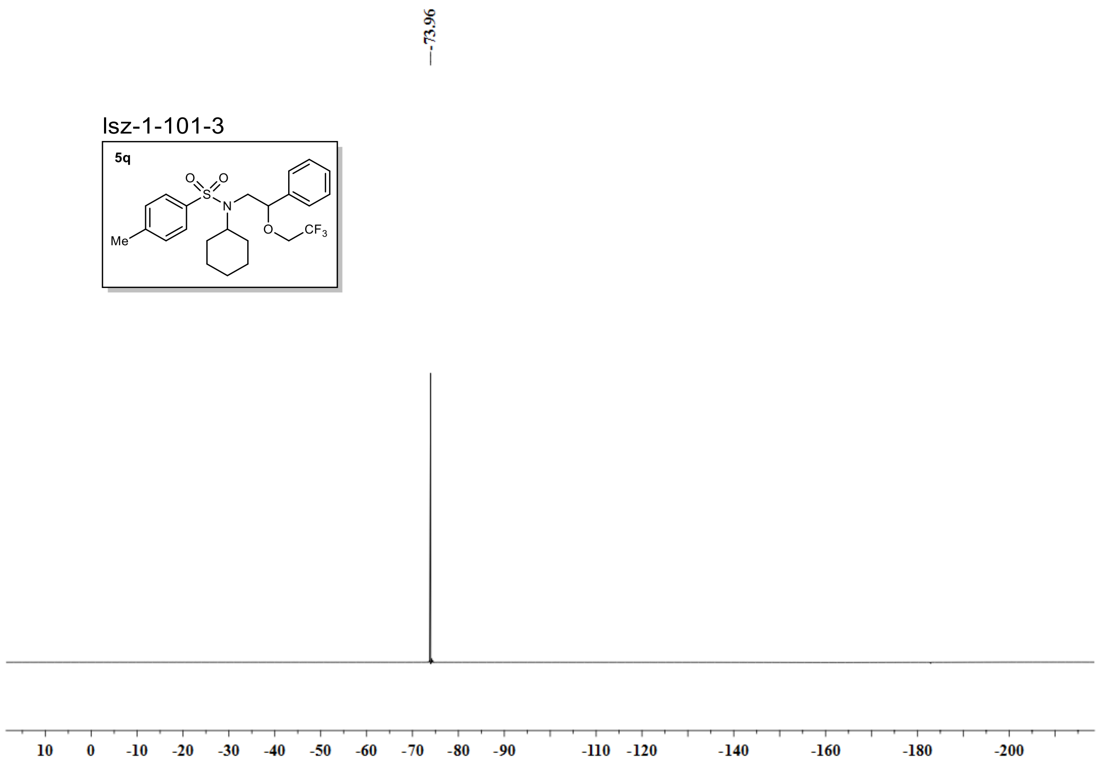

^{1}H NMR spectrum of 5r (400 MHz, $CDCl_3$):

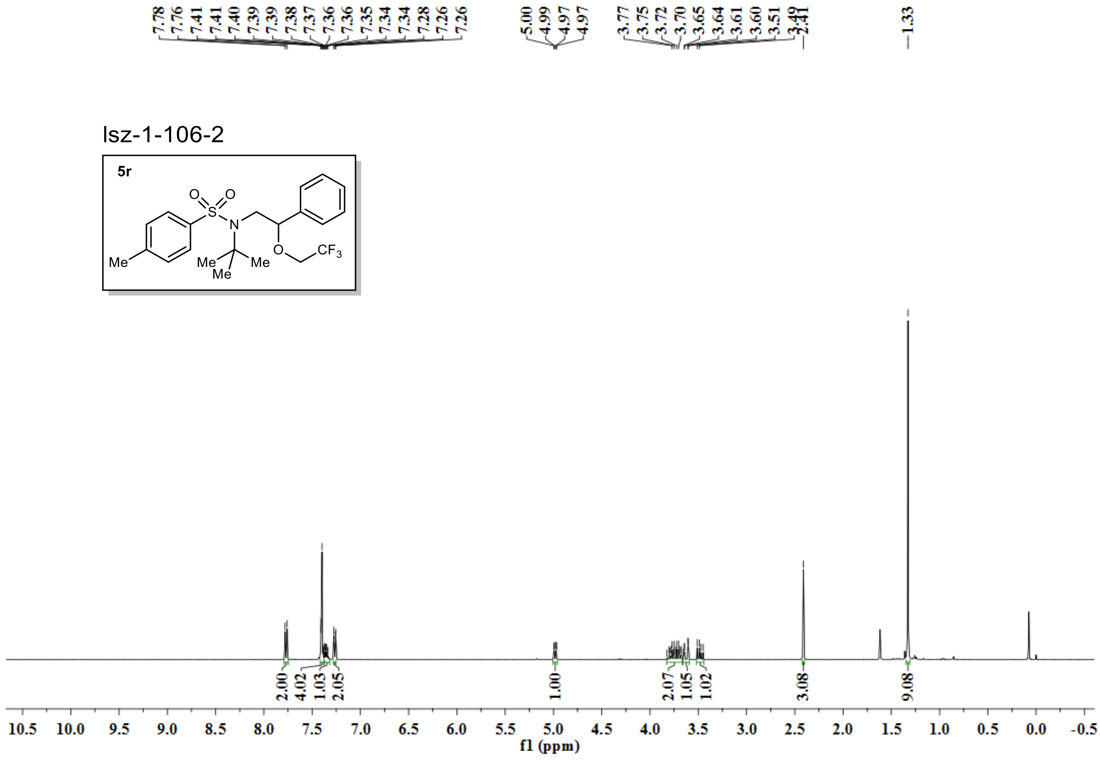

^{13}C NMR spectrum of 5r (101 MHz, $CDCl_3$):

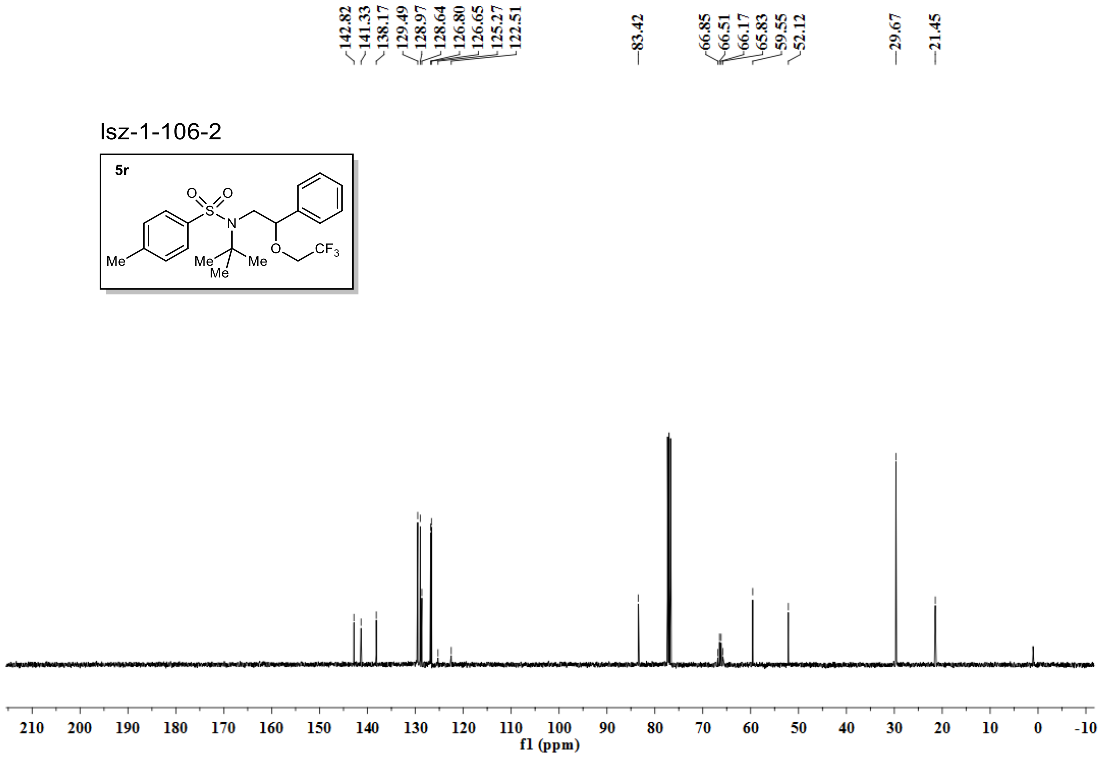

^{19}F NMR spectrum of 5r (377 MHz, $CDCl_3$):

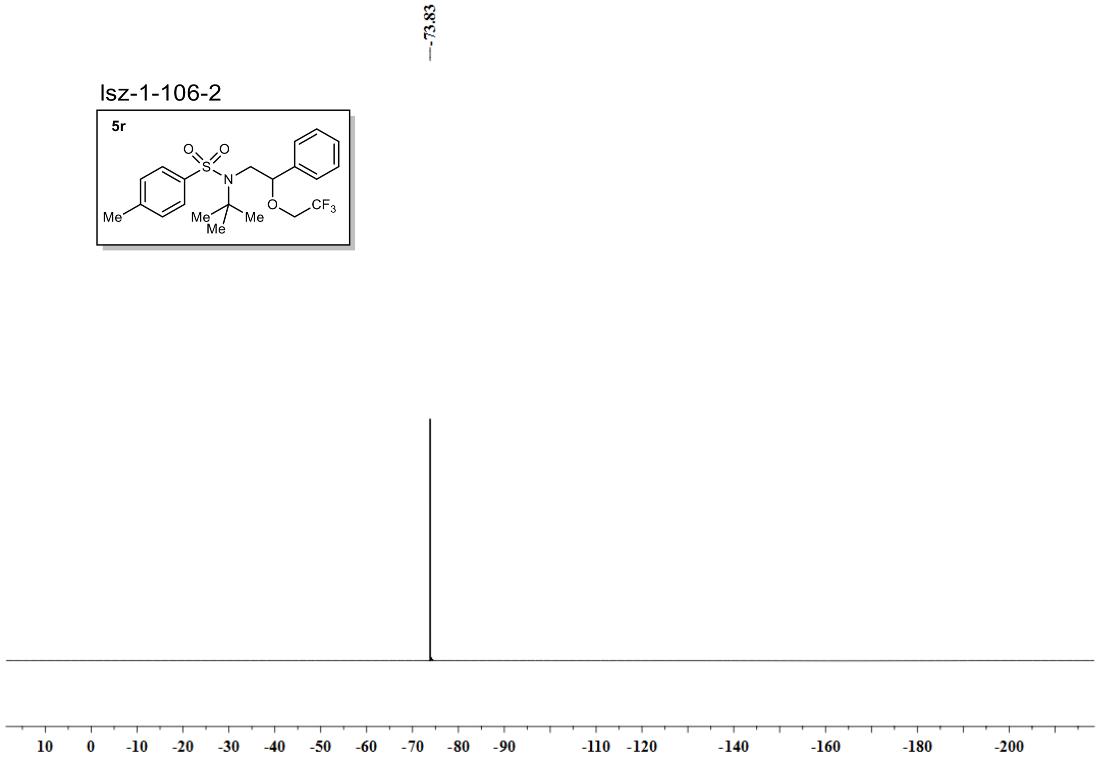

^{1}H NMR spectrum of 5s (400 MHz, $CDCl_3$):

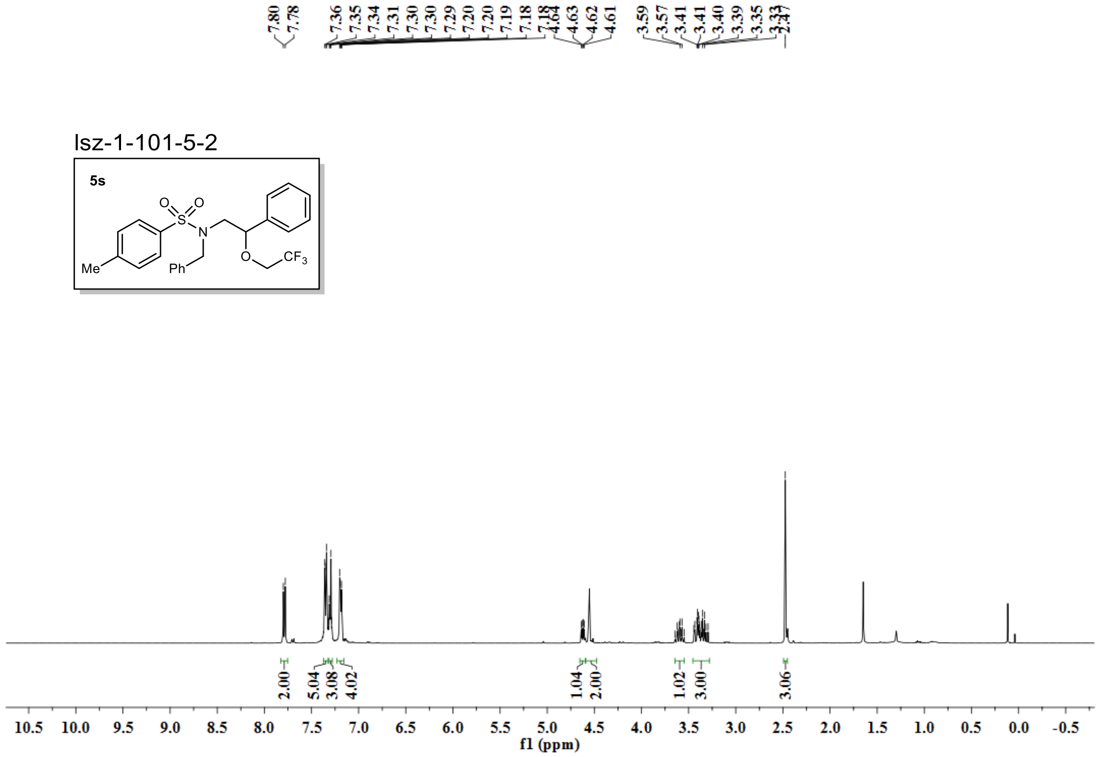

^{13}C NMR spectrum of 5s (101 MHz, $CDCl_3$):

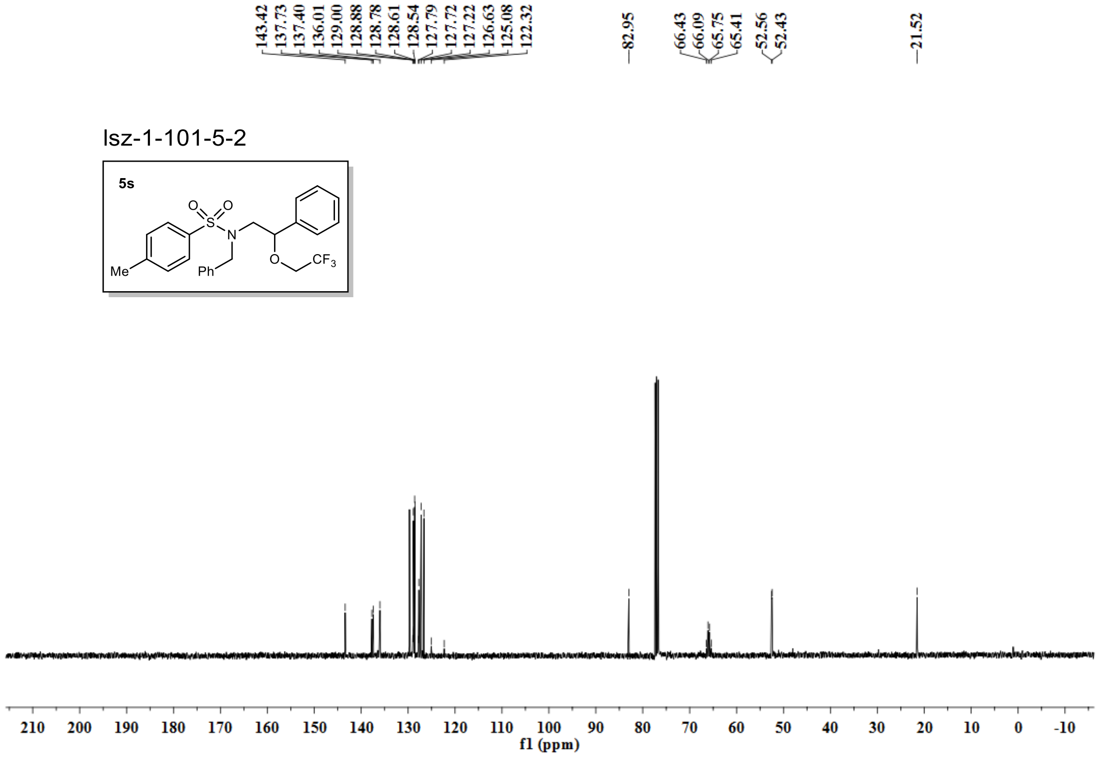

^{19}F NMR spectrum of 5s (377 MHz, $CDCl_3$):

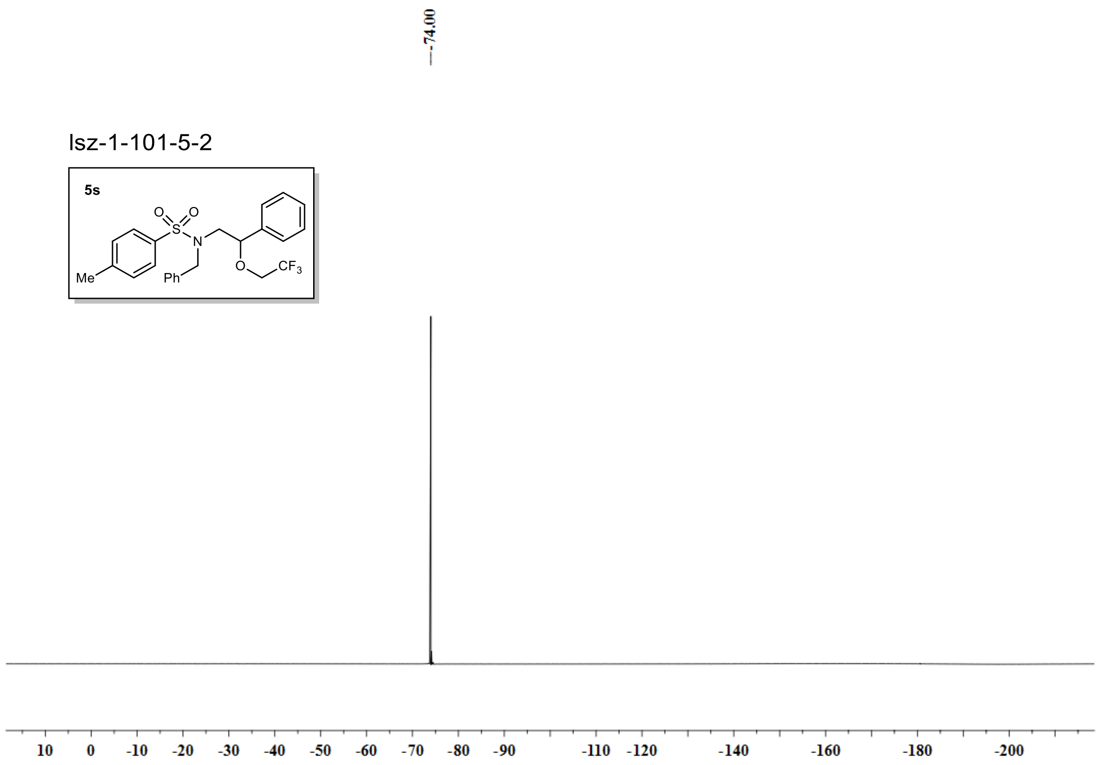

^{1}H NMR spectrum of 5t (400 MHz, $CDCl_3$):

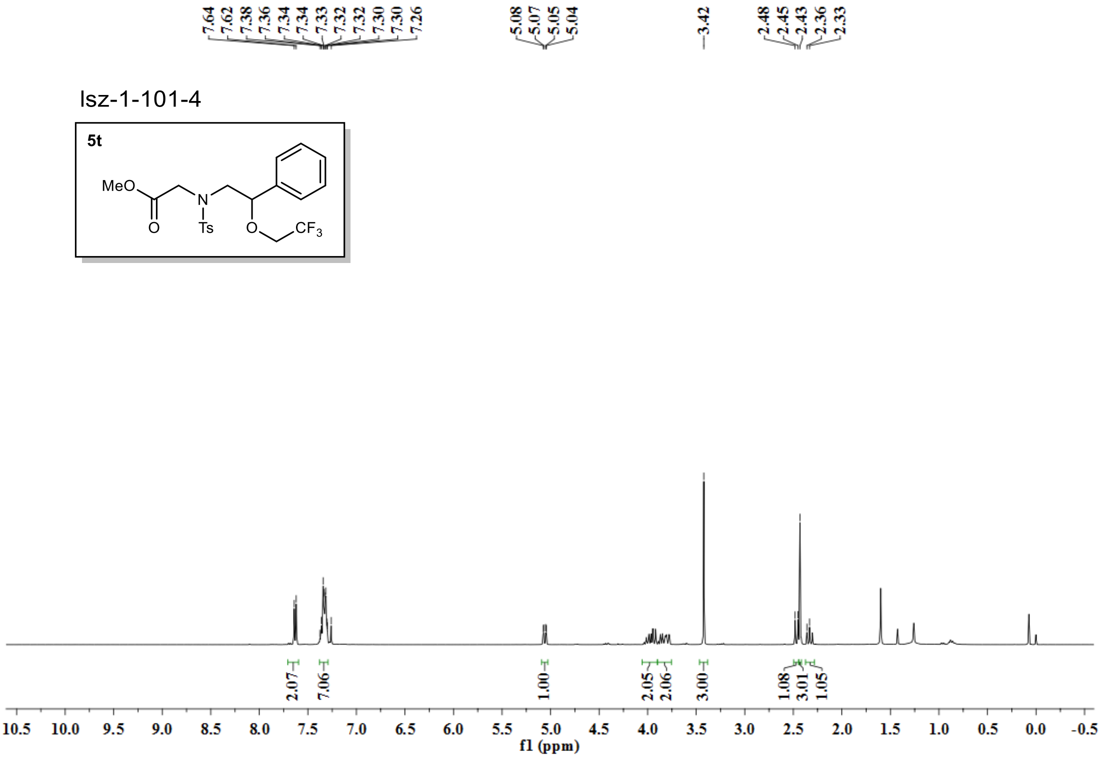

^{13}C NMR spectrum of 5t (101 MHz, $CDCl_3$):

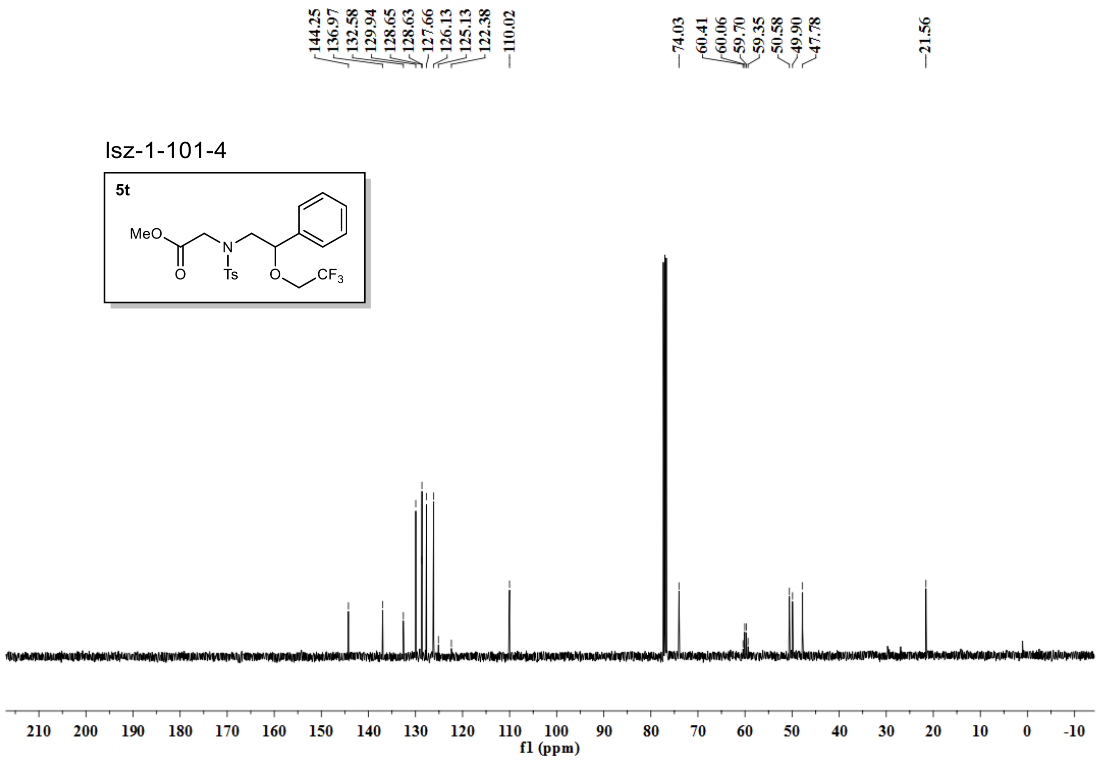

^{19}F NMR spectrum of 5t (377 MHz, $CDCl_3$):

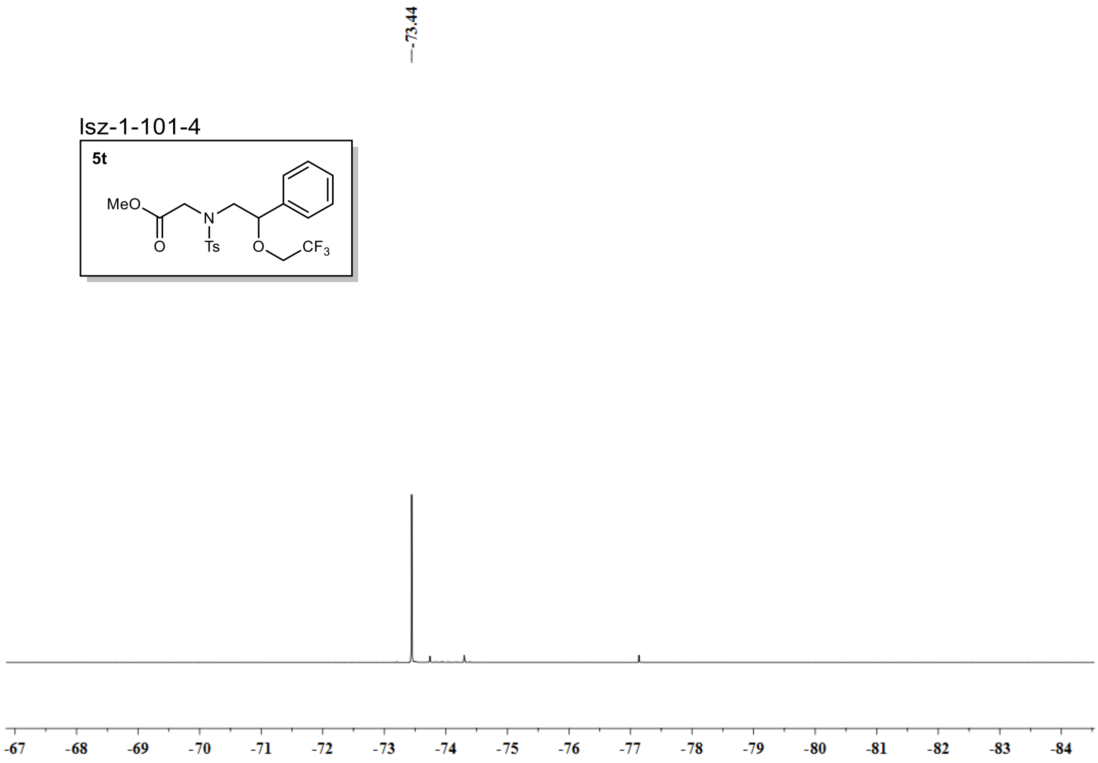

^{1}H NMR spectrum of 6a (400 MHz, $CDCl_3$):

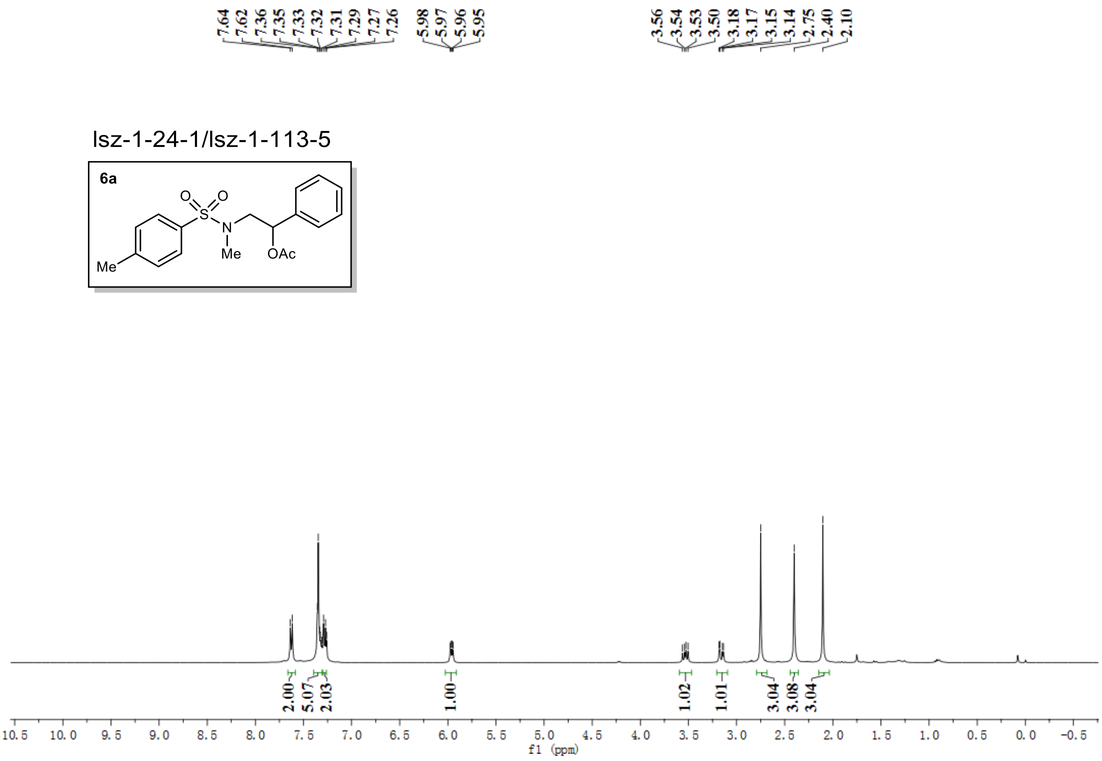

^{13}C NMR spectrum of 6a (101 MHz, $CDCl_3$):

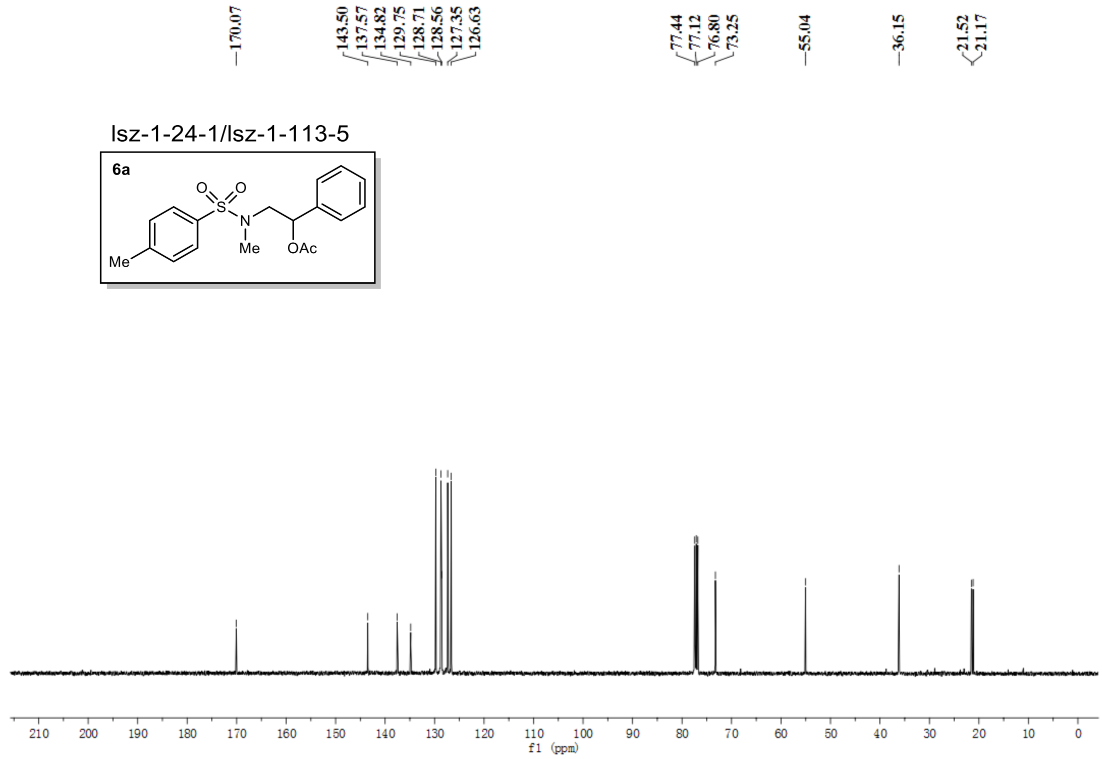

^{1}H NMR spectrum of 6b (400 MHz, $CDCl_3$):

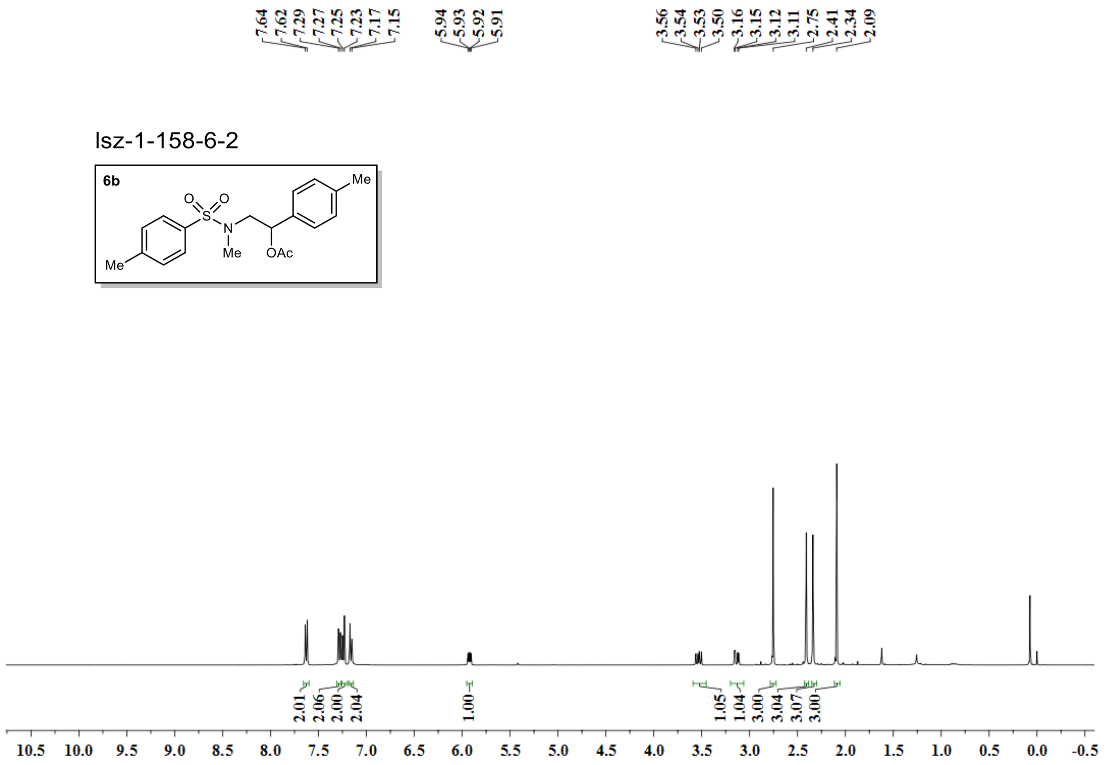

^{13}C NMR spectrum of 6b (101 MHz, $CDCl_3$):

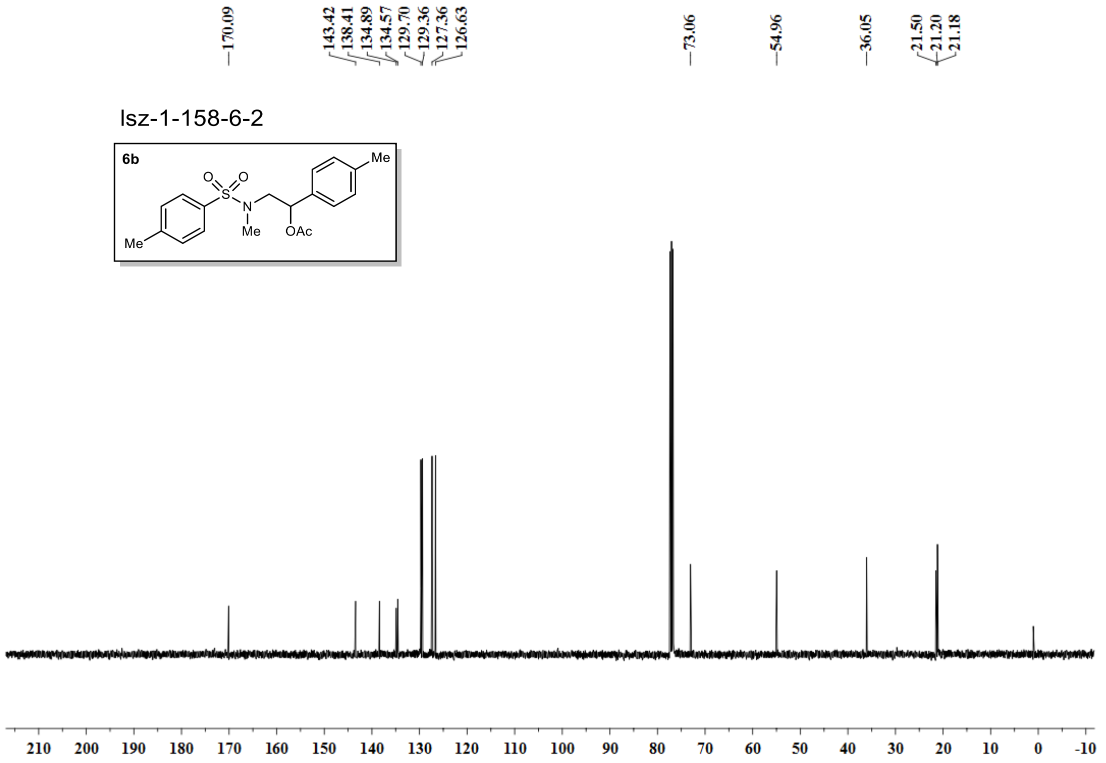

^{1}H NMR spectrum of 6c (400 MHz, $CDCl_3$):

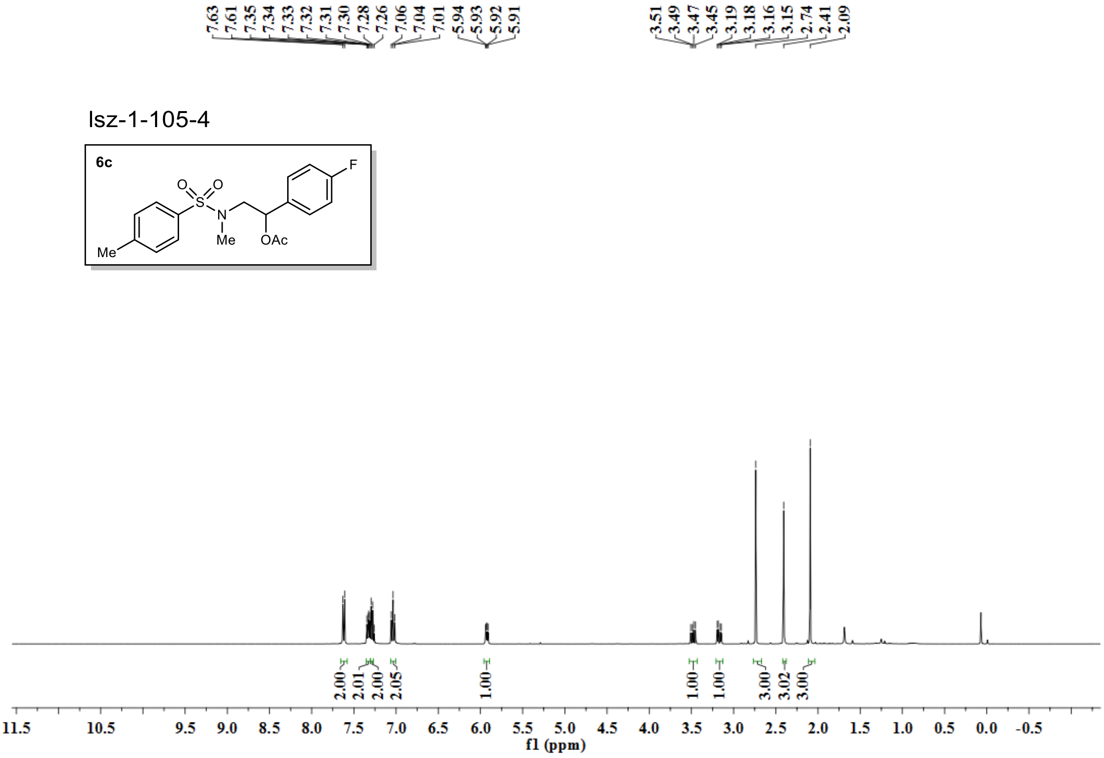

^{13}C NMR spectrum of 6c (101 MHz, $CDCl_3$):

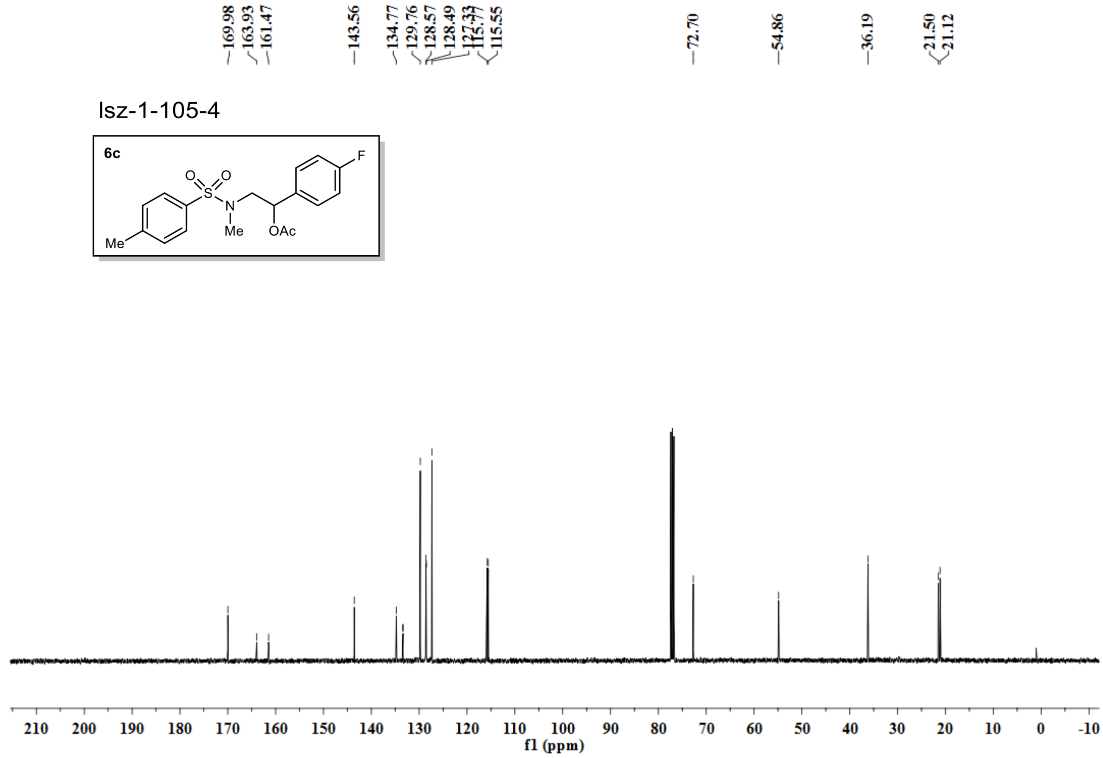

^{19}C NMR spectrum of 6c (377 MHz, $CDCl_3$):

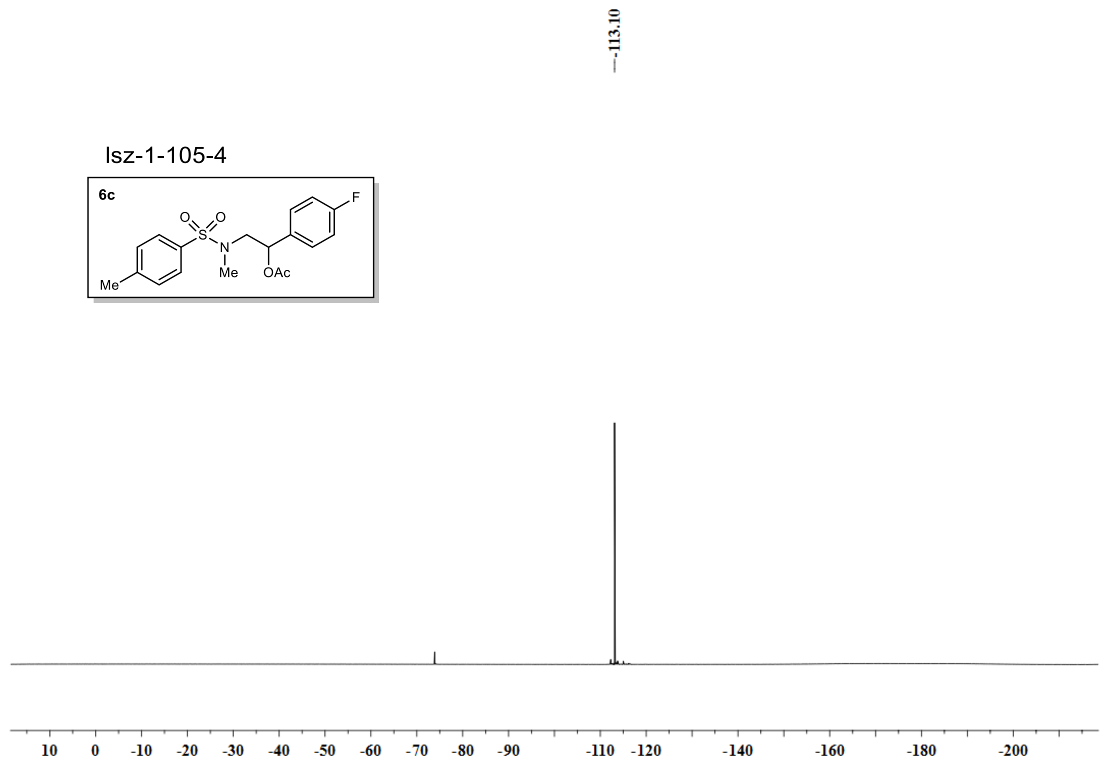

^{1}H NMR spectrum of 6d (400 MHz, $CDCl_3$):

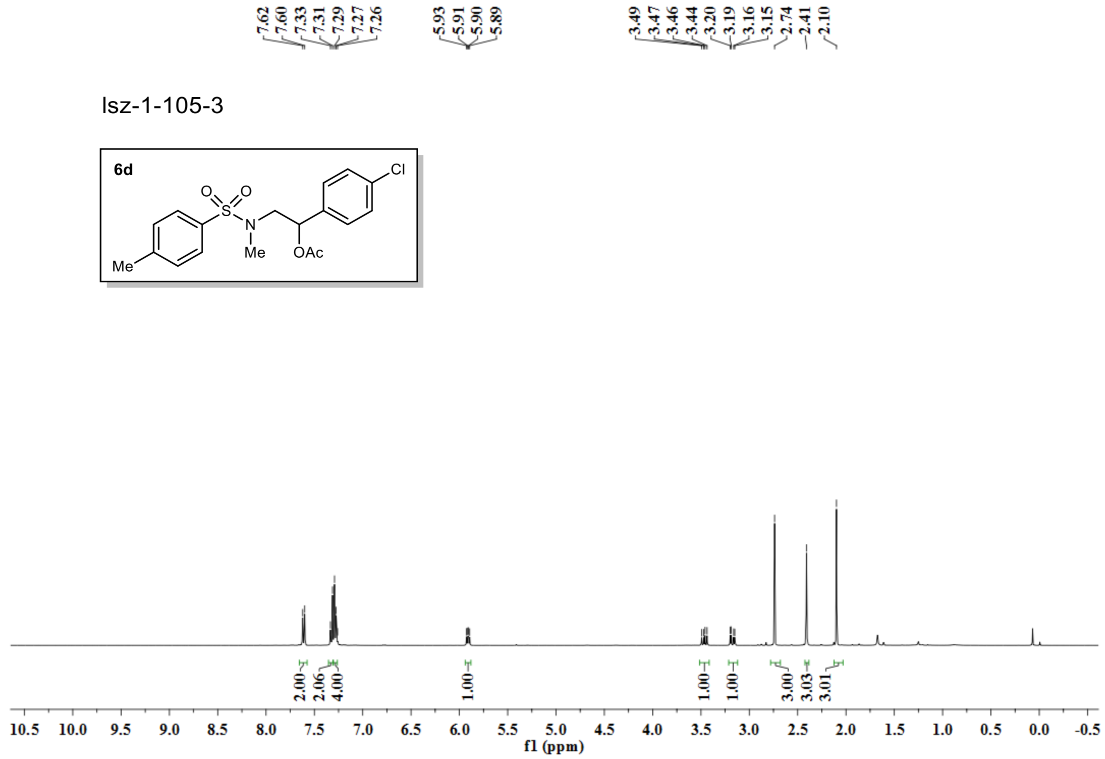

^{13}C NMR spectrum of 6d (101 MHz, $CDCl_3$):

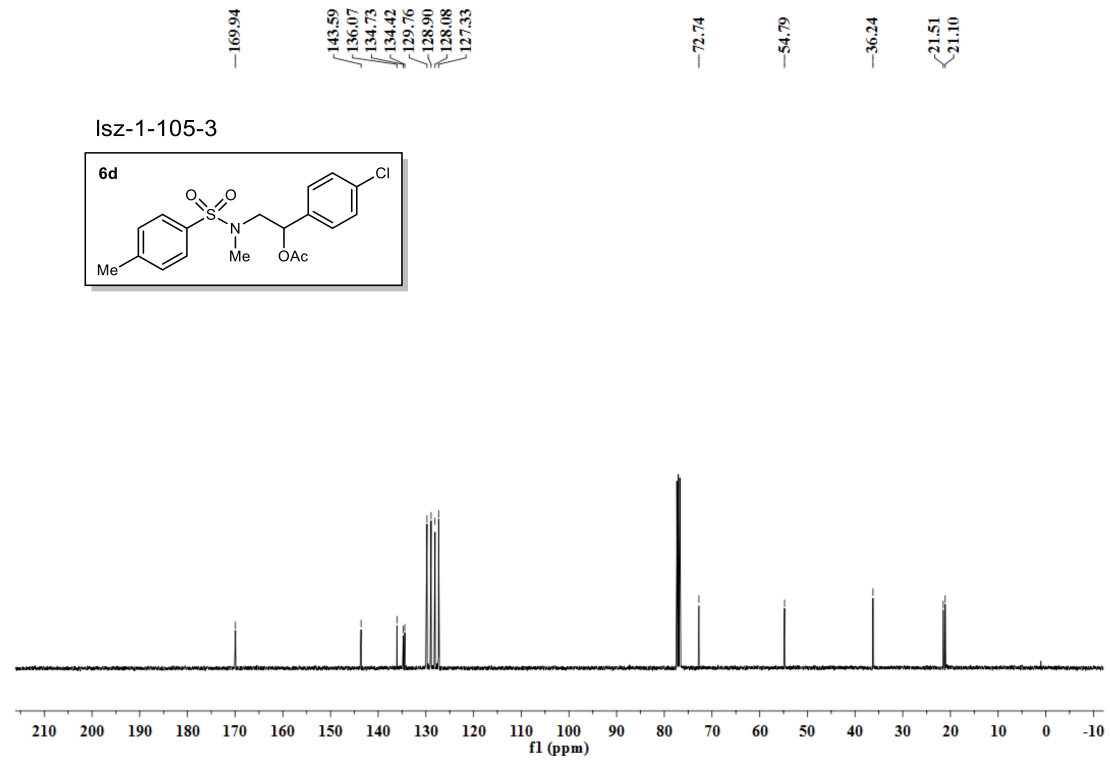

^{1}H NMR spectrum of 6e (400 MHz, $CDCl_3$):

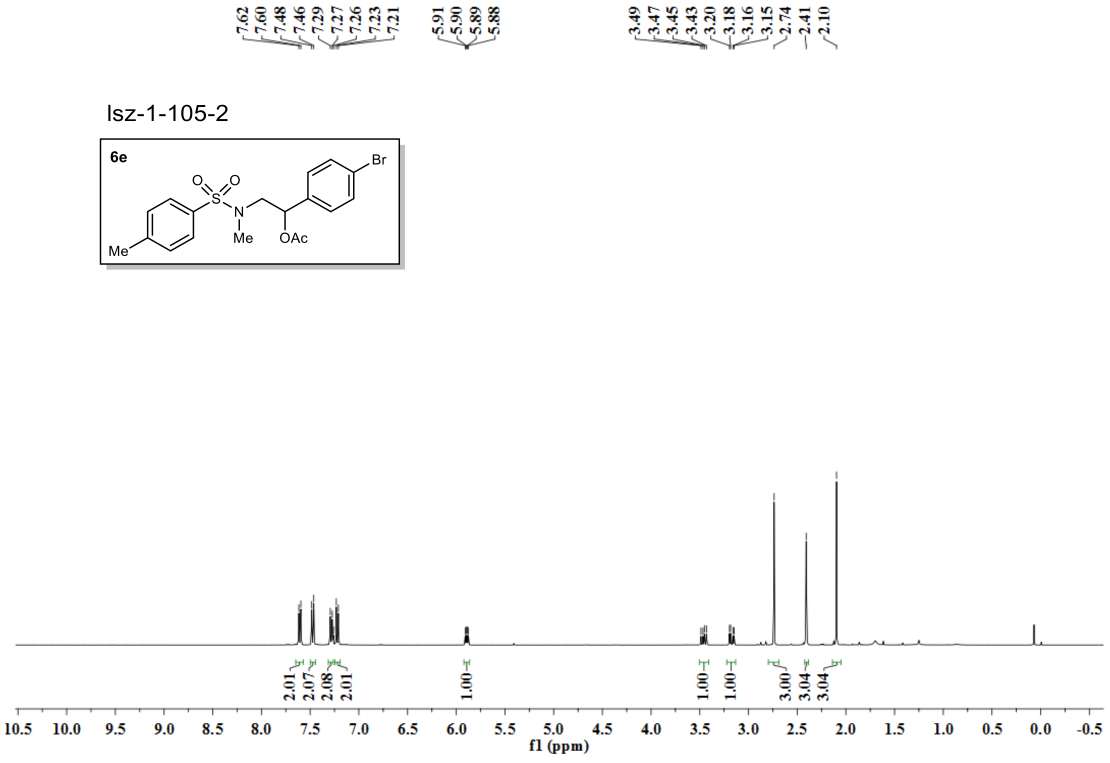

^{13}C NMR spectrum of 6e (101 MHz, $CDCl_3$):

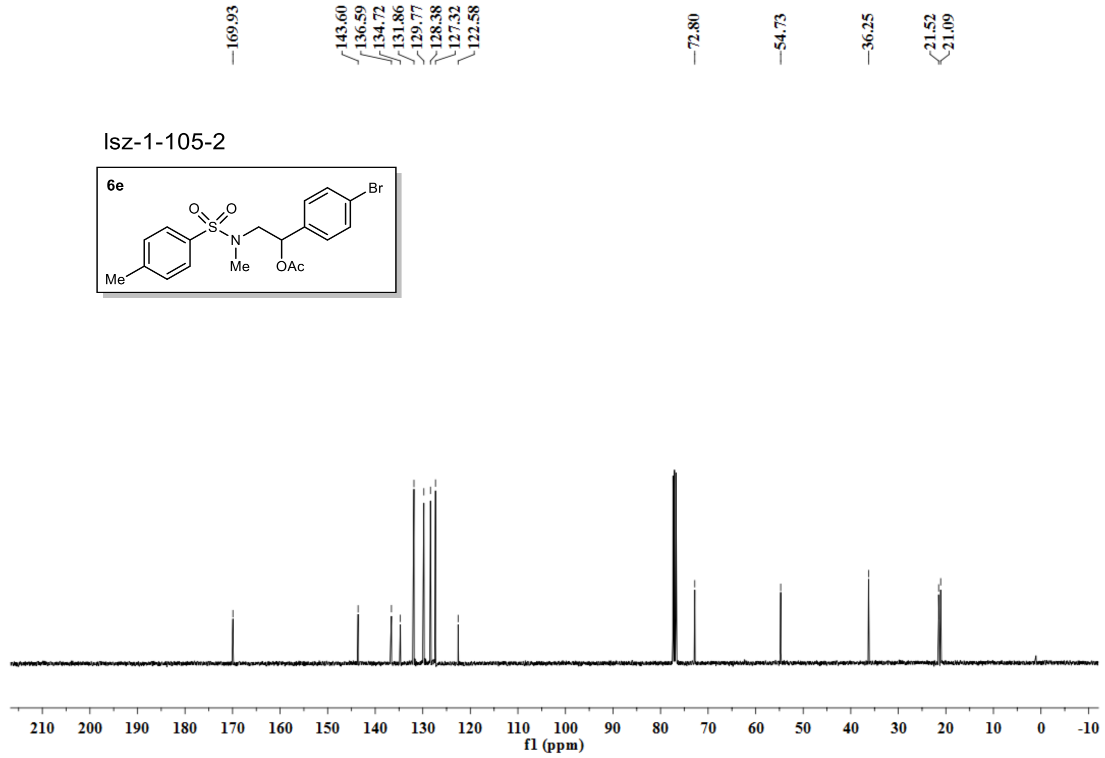

^{1}H NMR spectrum of 6f (400 MHz, $CDCl_3$):

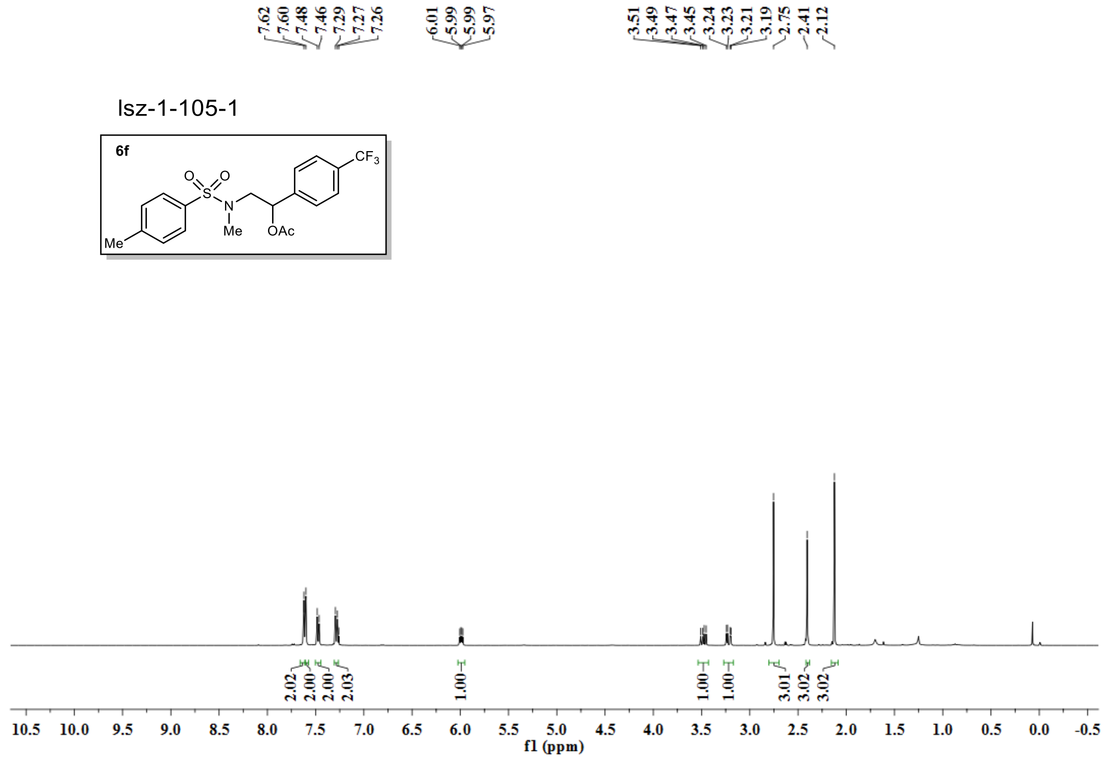

^{13}C NMR spectrum of 6f (101 MHz, $CDCl_3$):

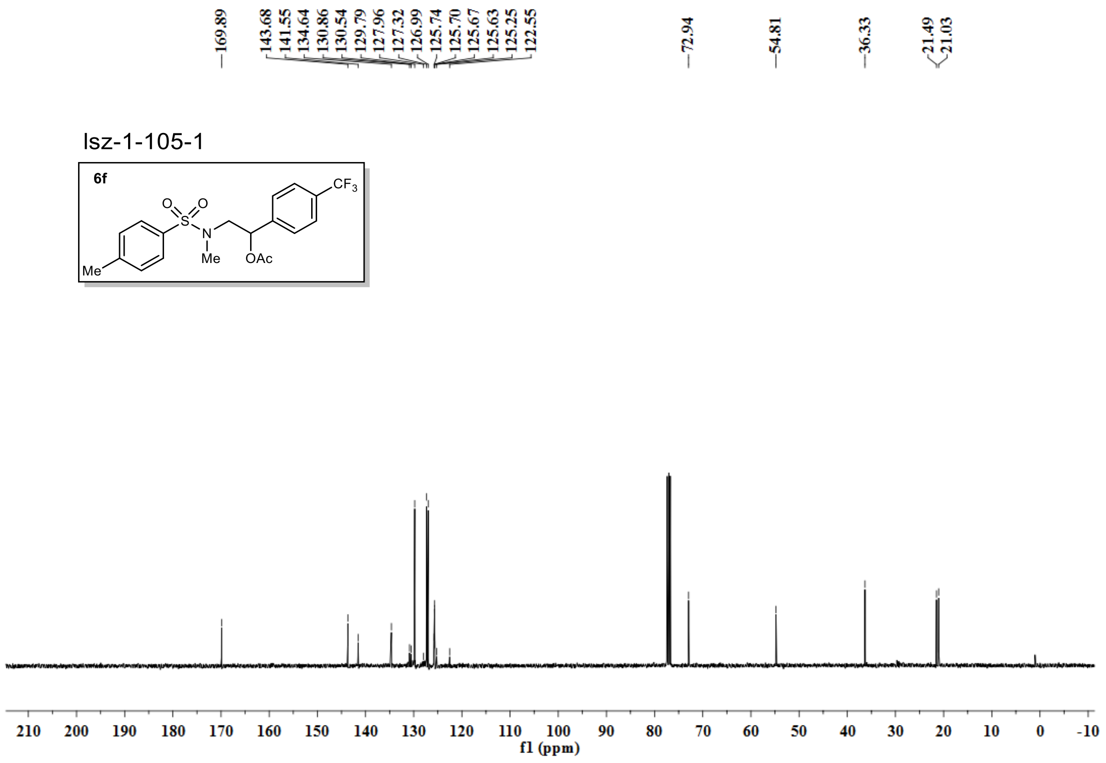

^{19}F NMR spectrum of 6f (377 MHz, $CDCl_3$):

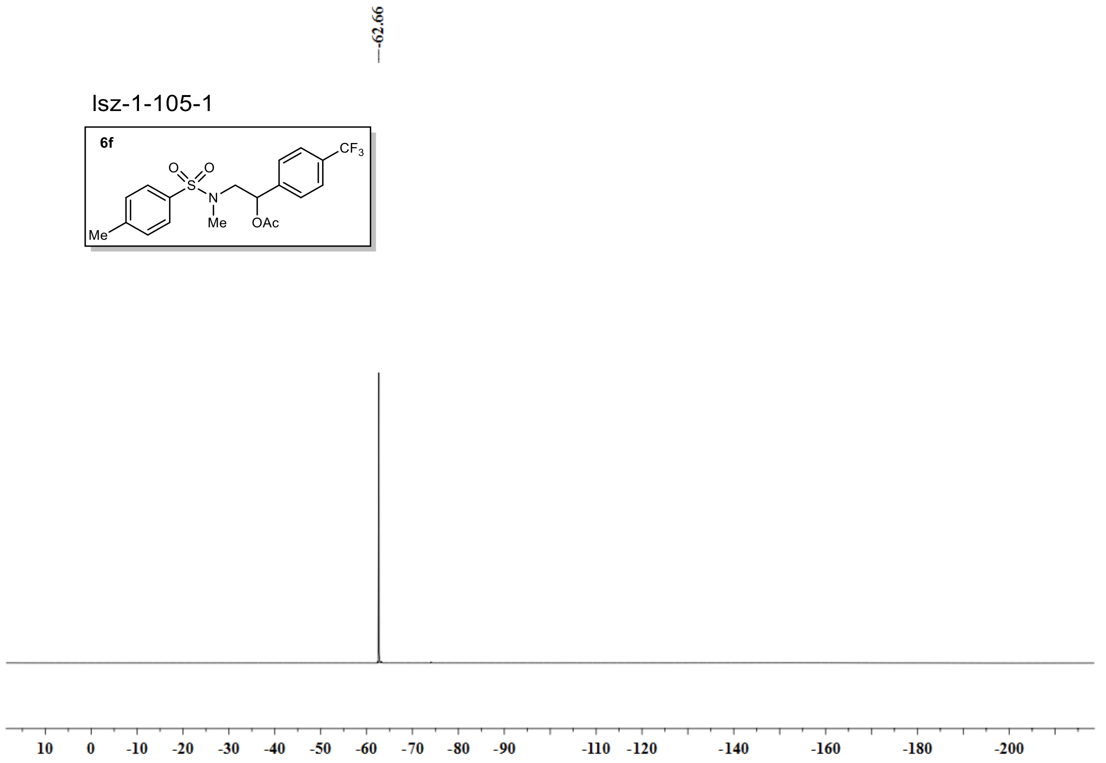

^{1}**H NMR spectrum of 6g** (400 MHz, $CDCl_3$):

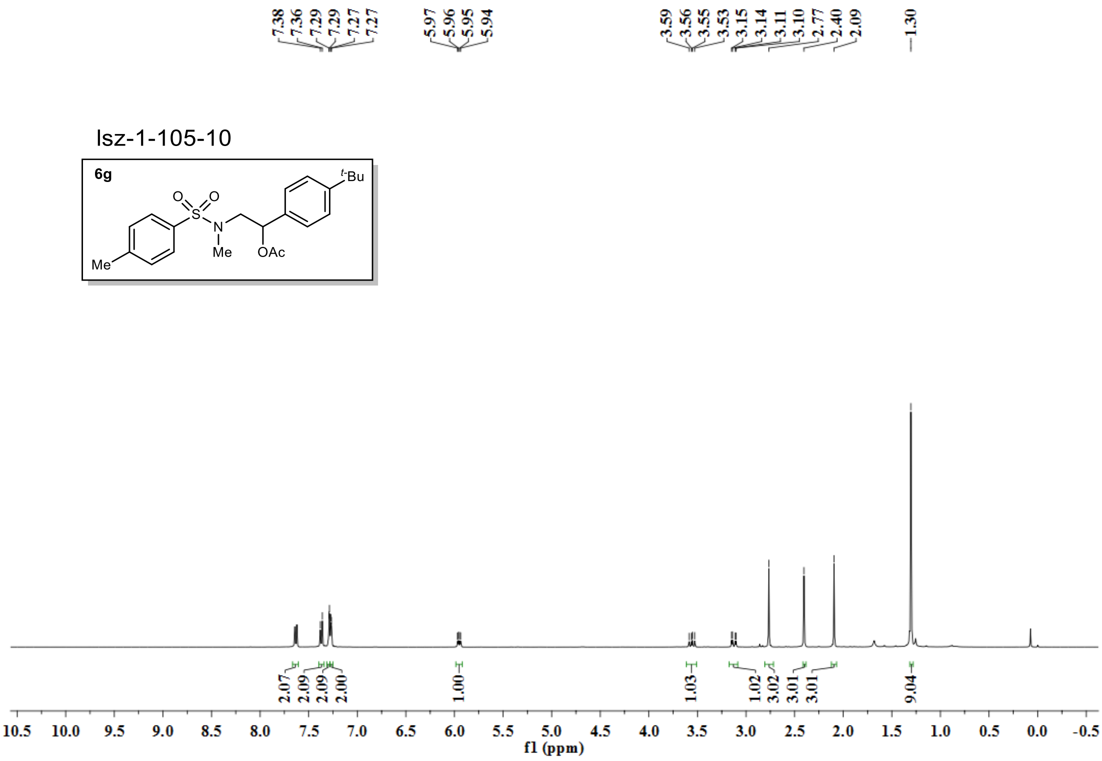

^{13}**C NMR spectrum of 6g** (101 MHz, $CDCl_3$):

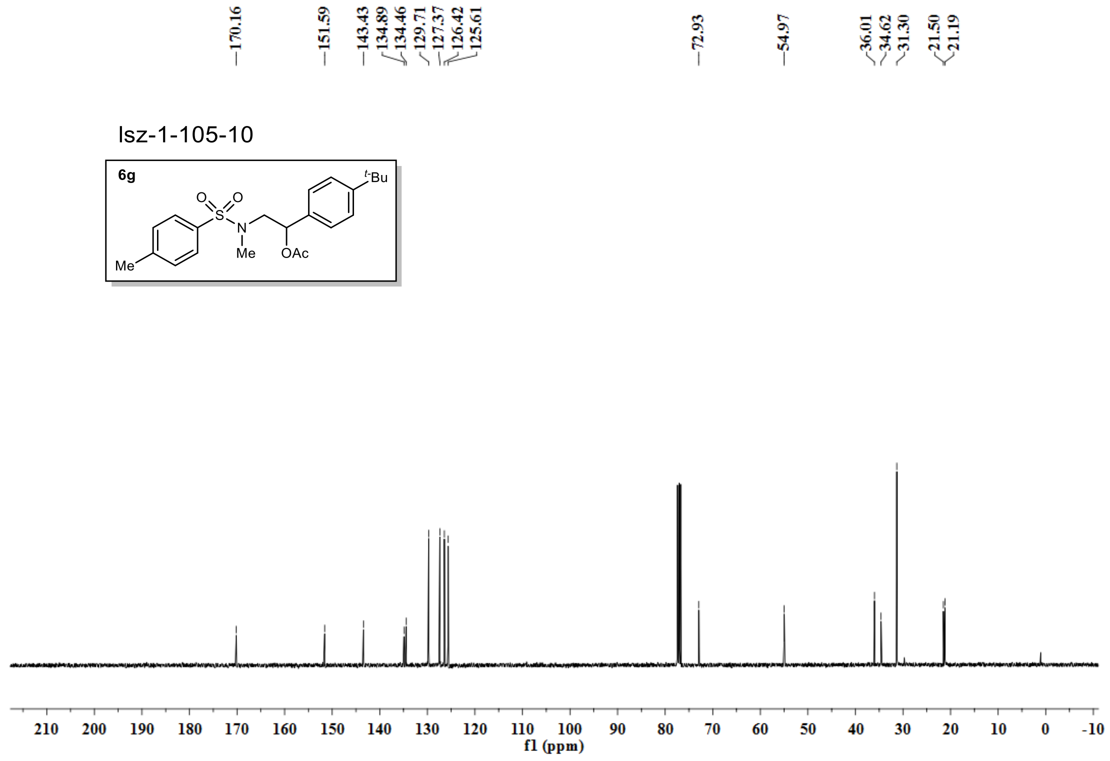

^{1}H NMR spectrum of 6h (400 MHz, $CDCl_3$):

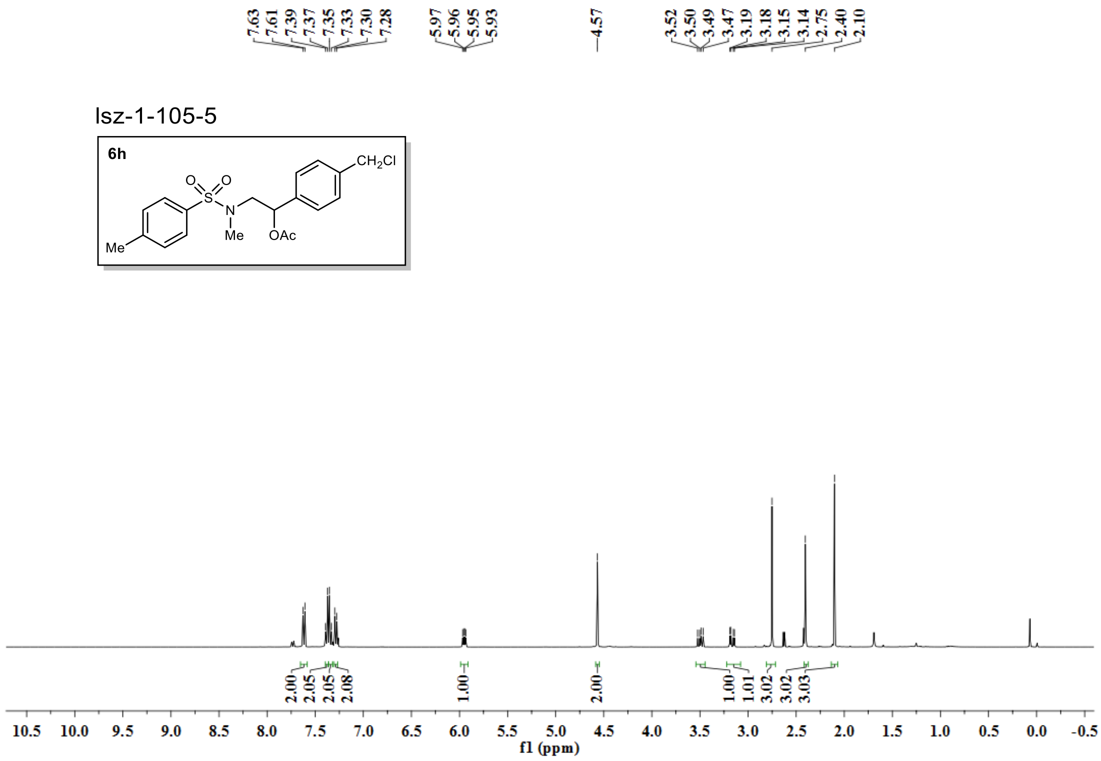

^{13}C NMR spectrum of 6h (101 MHz, $CDCl_3$):

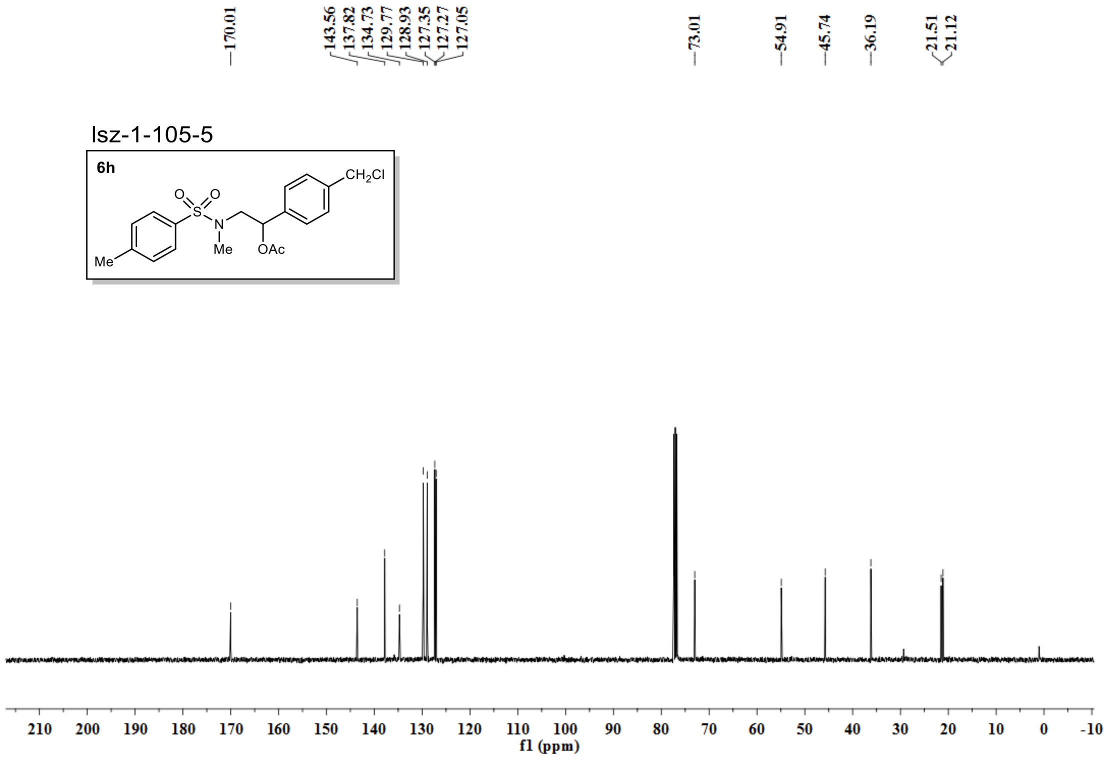

^{1}H NMR spectrum of 6i (400 MHz, $CDCl_3$):

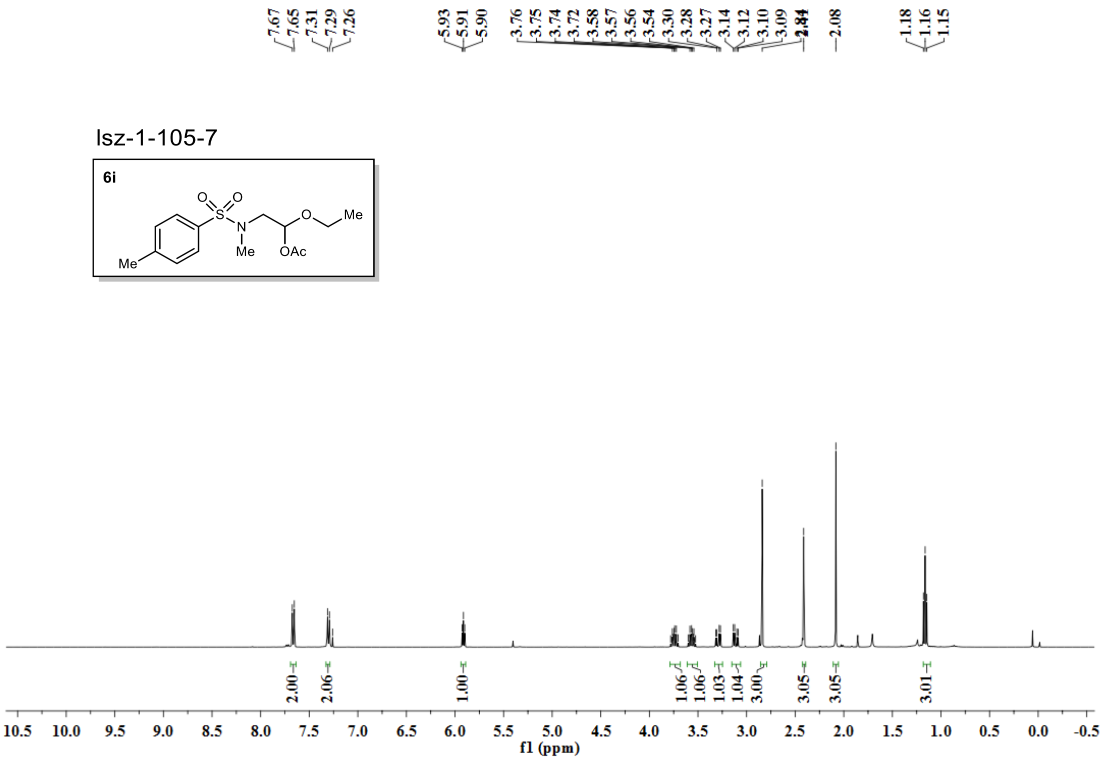

^{13}C NMR spectrum of 6i (101 MHz, $CDCl_3$):

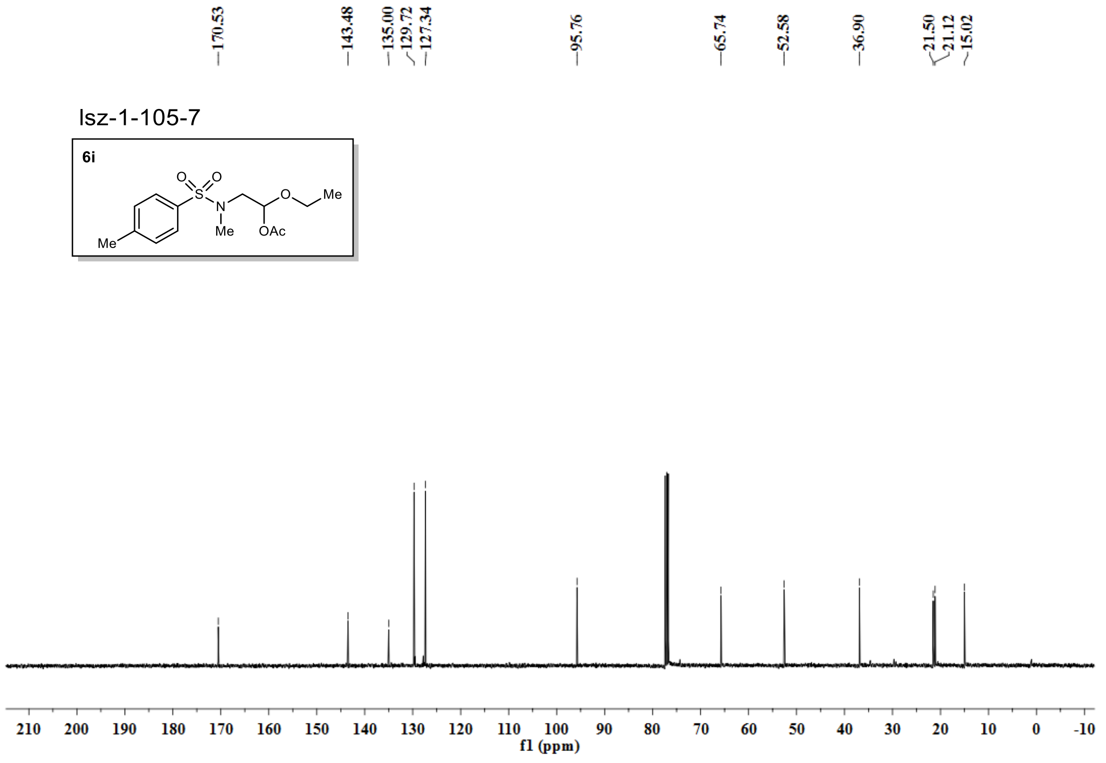

^{1}H NMR spectrum of 6j (400 MHz, $CDCl_3$):

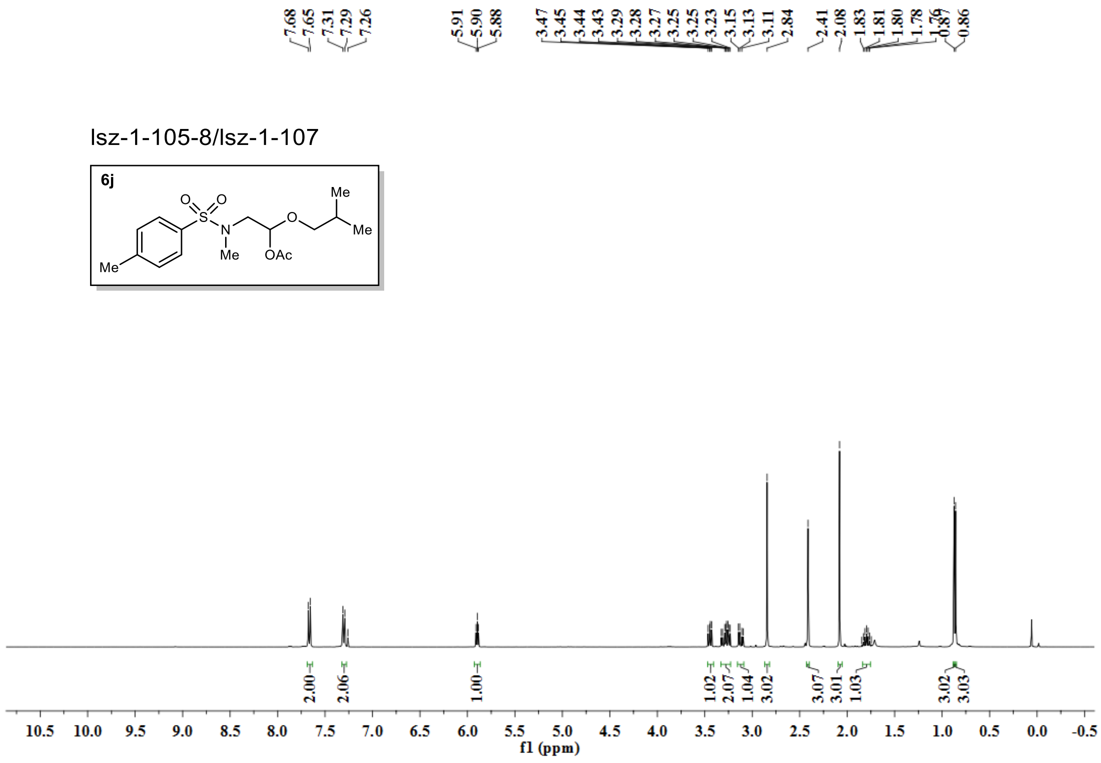

^{13}C NMR spectrum of 6j (101 MHz, $CDCl_3$):

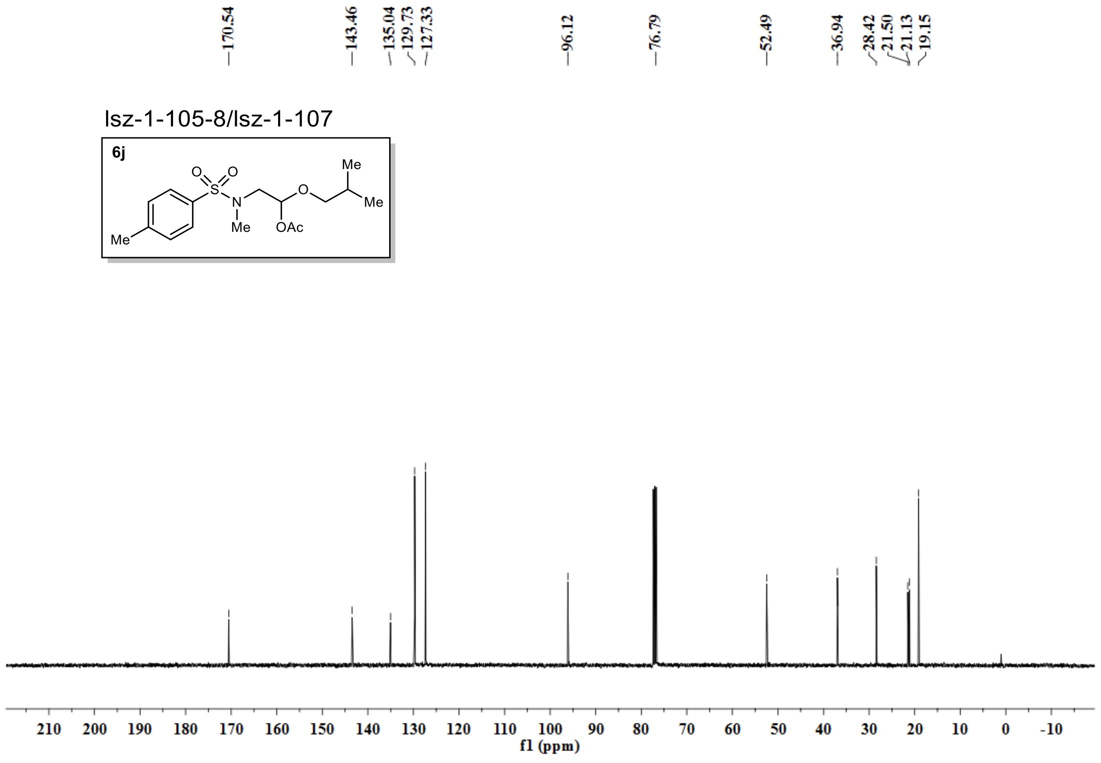

^{1}H NMR spectrum of 6k (400 MHz, $CDCl_3$):

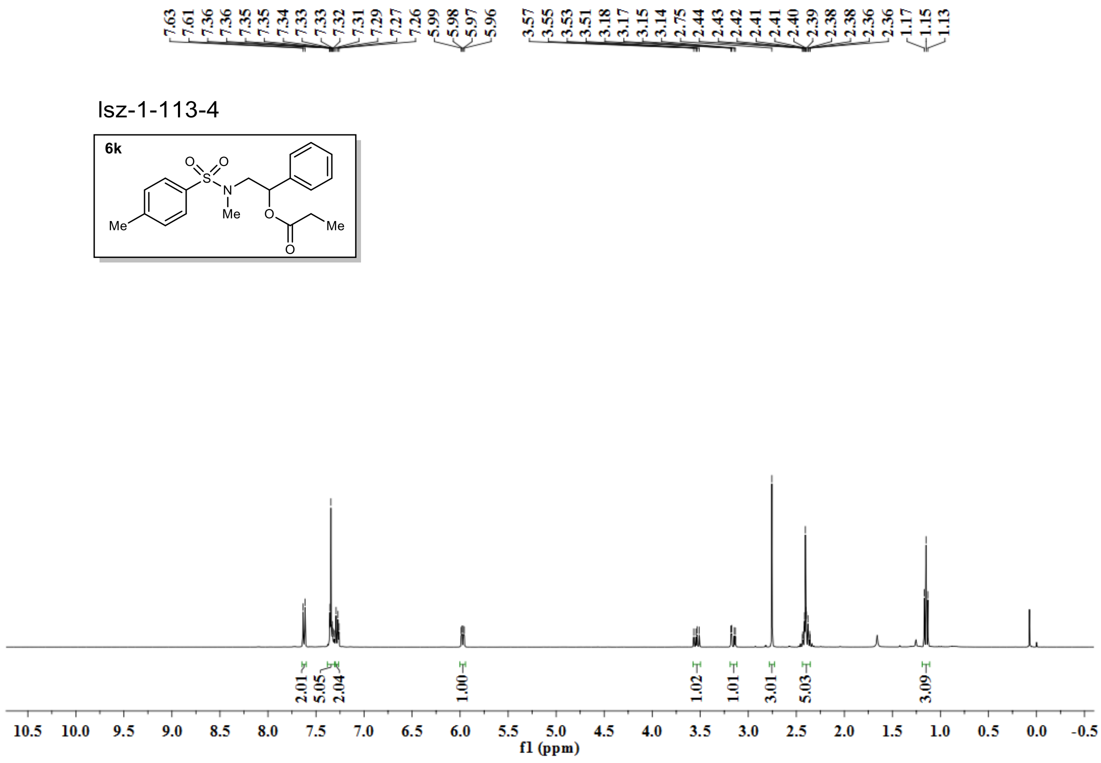

^{13}C NMR spectrum of 6k (101 MHz, $CDCl_3$):

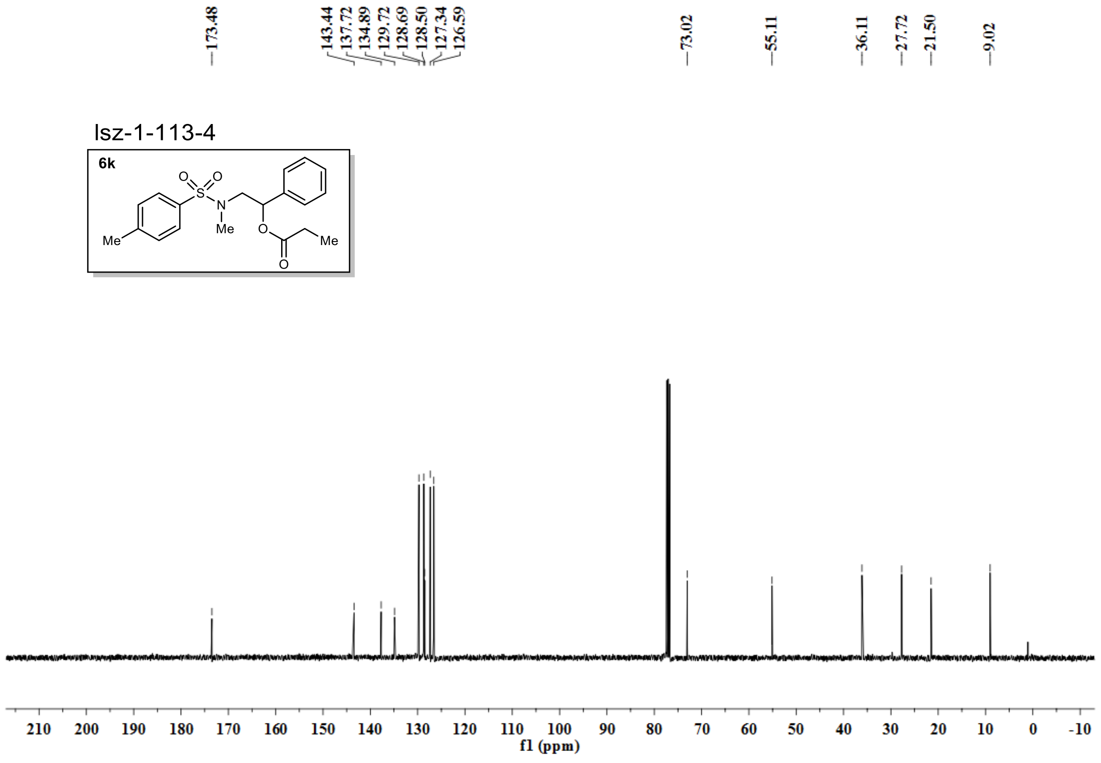

^{1}H NMR spectrum of 6l (400 MHz, $CDCl_3$):

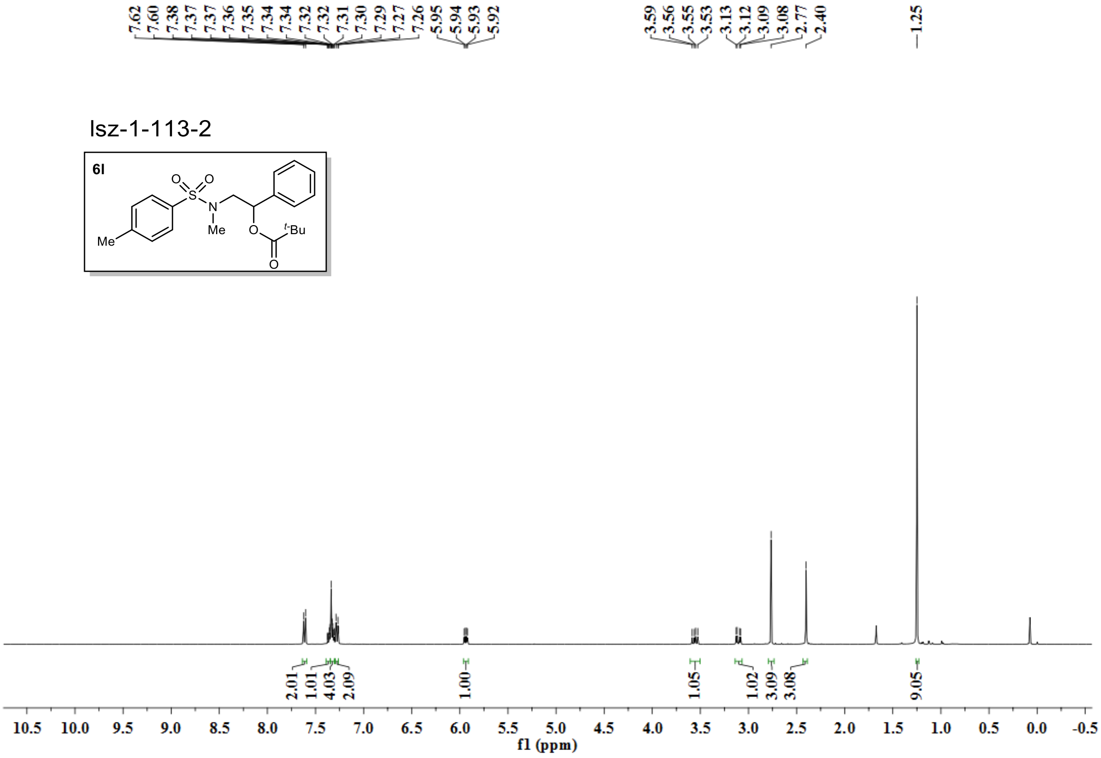

^{13}C NMR spectrum of 6l (101 MHz, $CDCl_3$):

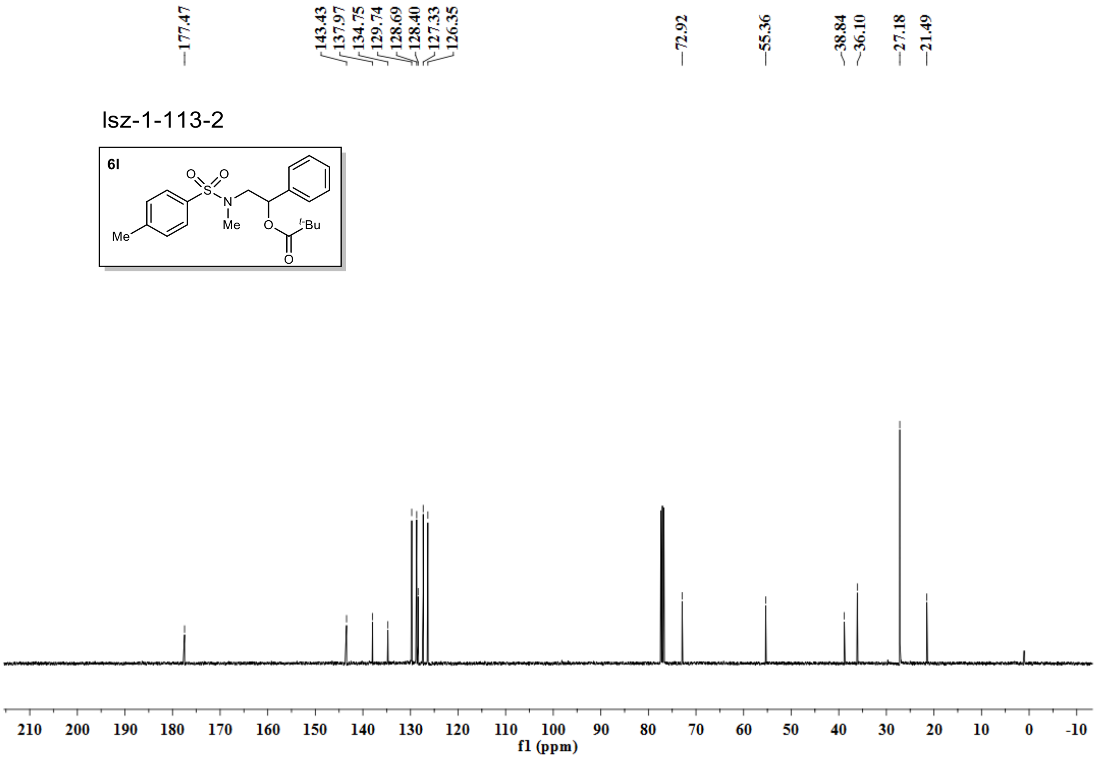

^{1}H NMR spectrum of 6m (400 MHz, $CDCl_3$):

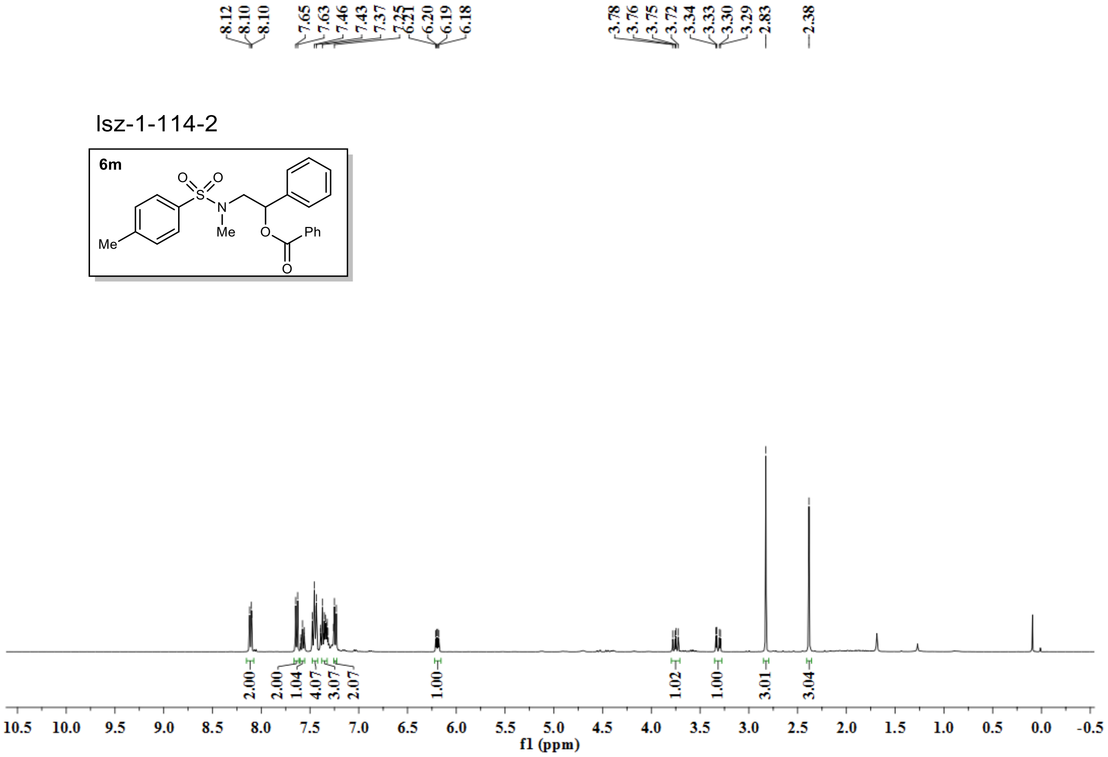

^{13}C NMR spectrum of 6m (101 MHz, $CDCl_3$):

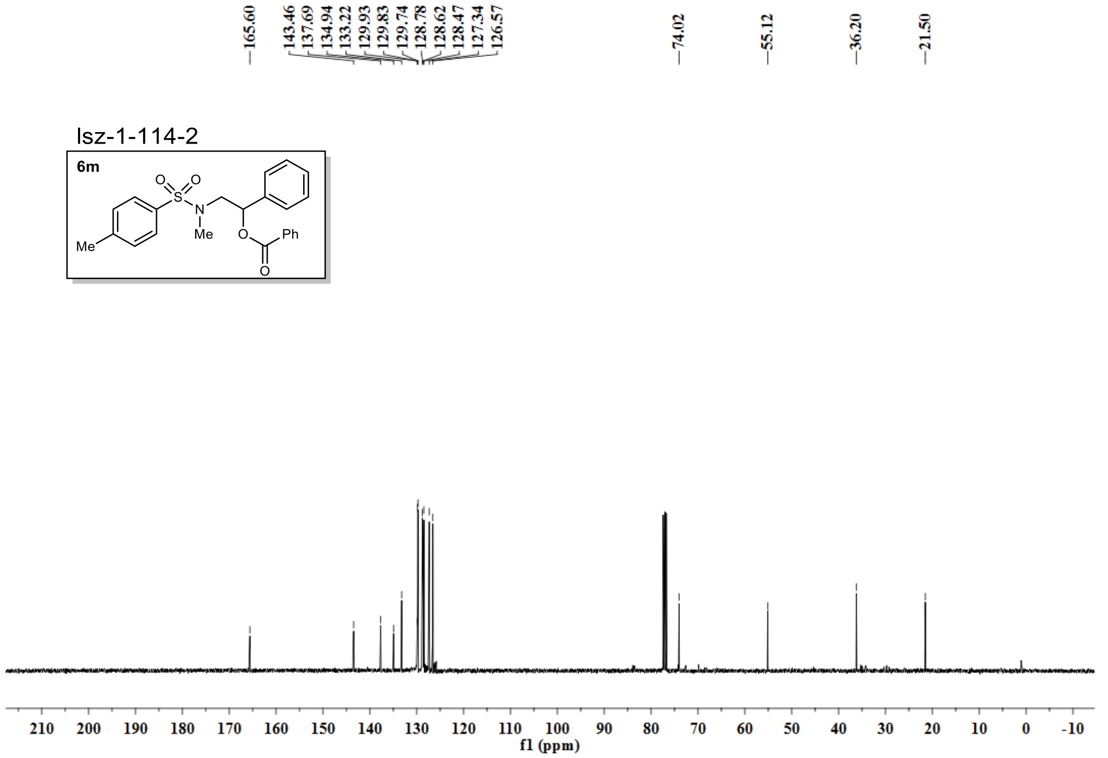

^{1}H NMR spectrum of 6n (400 MHz, $CDCl_3$):

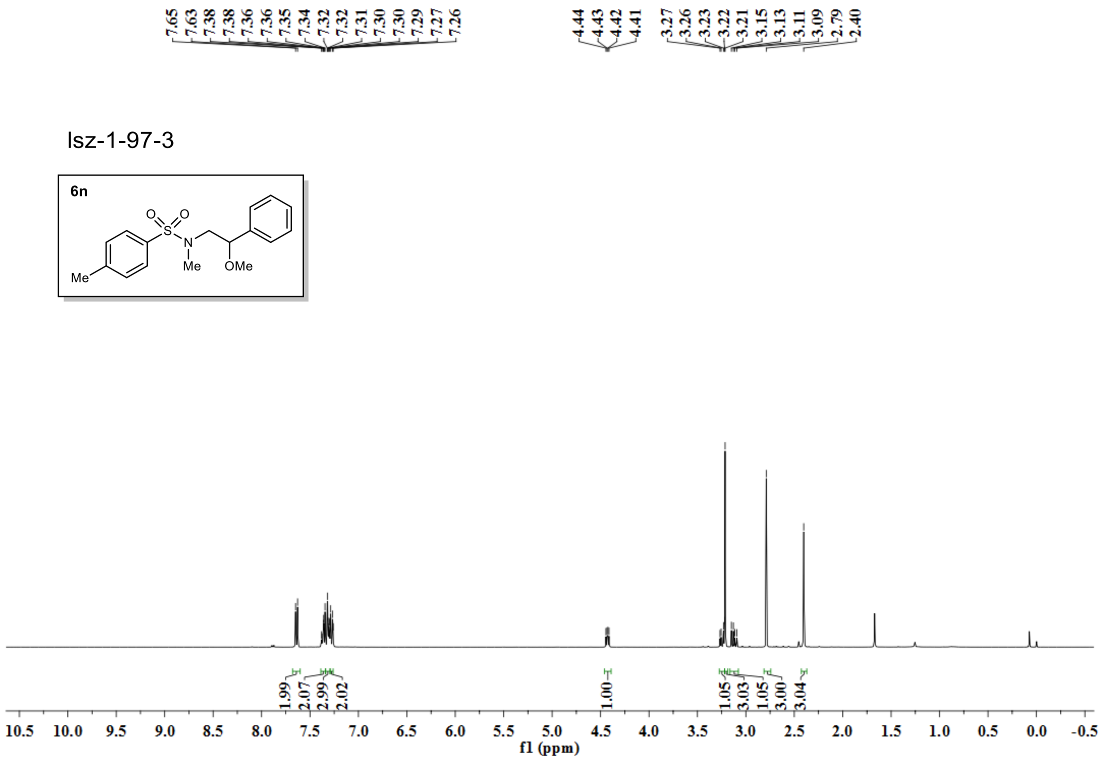

^{13}C NMR spectrum of 6n (101 MHz, $CDCl_3$):

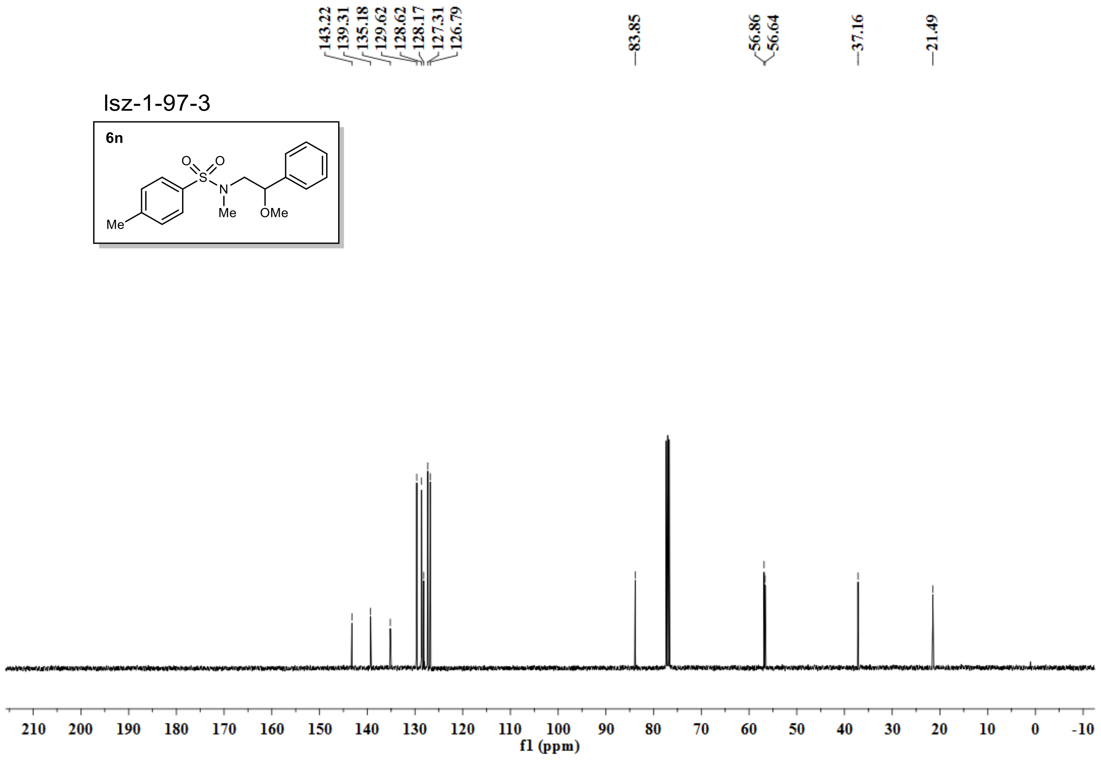

^{1}H NMR spectrum of 6o (400 MHz, $CDCl_3$):

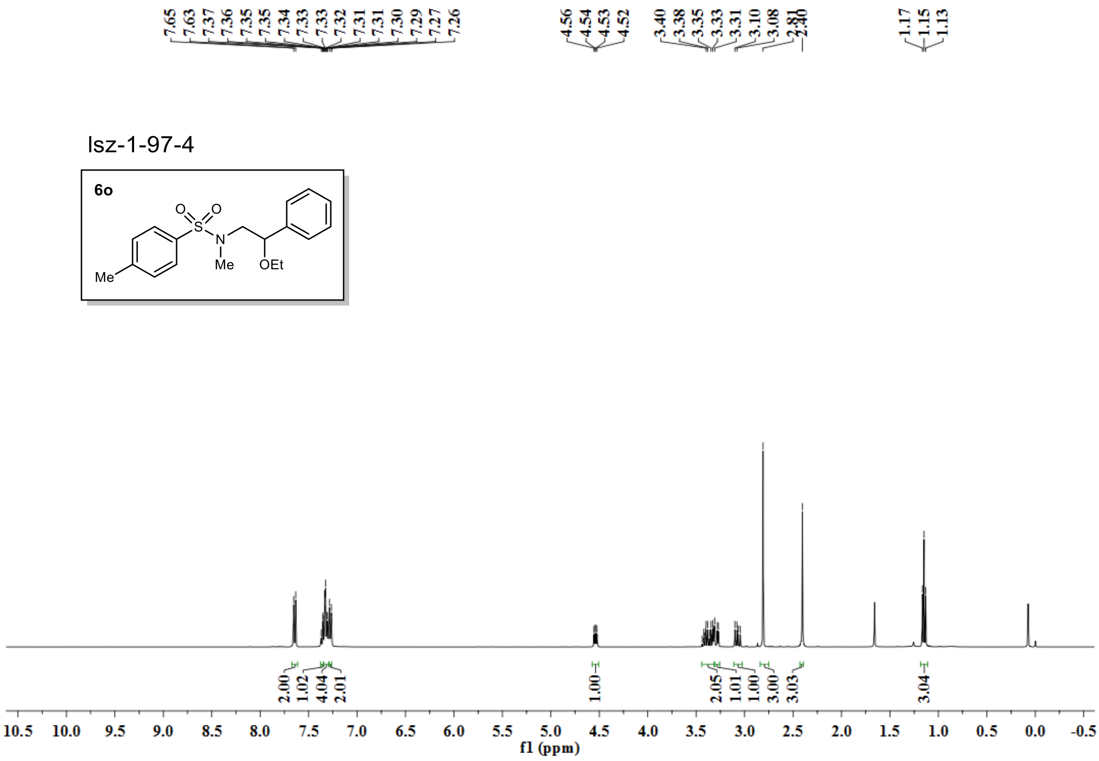

^{13}C NMR spectrum of 6o (101 MHz, $CDCl_3$):

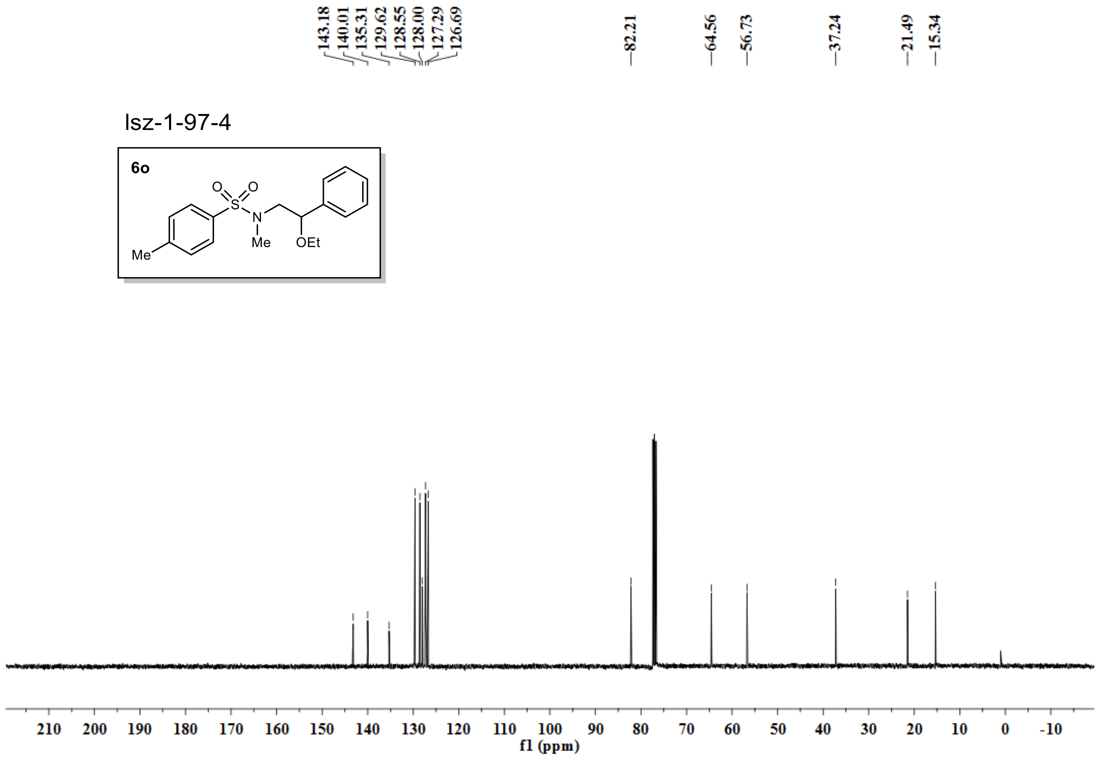

^{1}H NMR spectrum of 6p (400 MHz, $CDCl_3$):

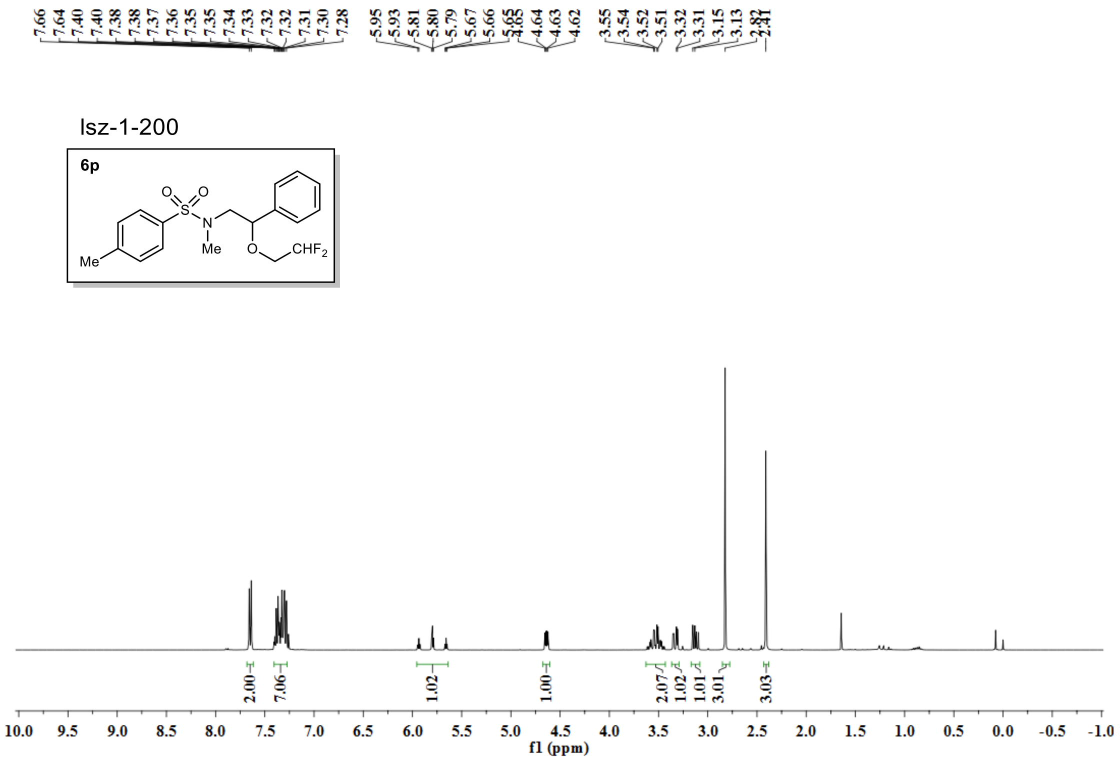

^{13}C NMR spectrum of 6p (101 MHz, $CDCl_3$):

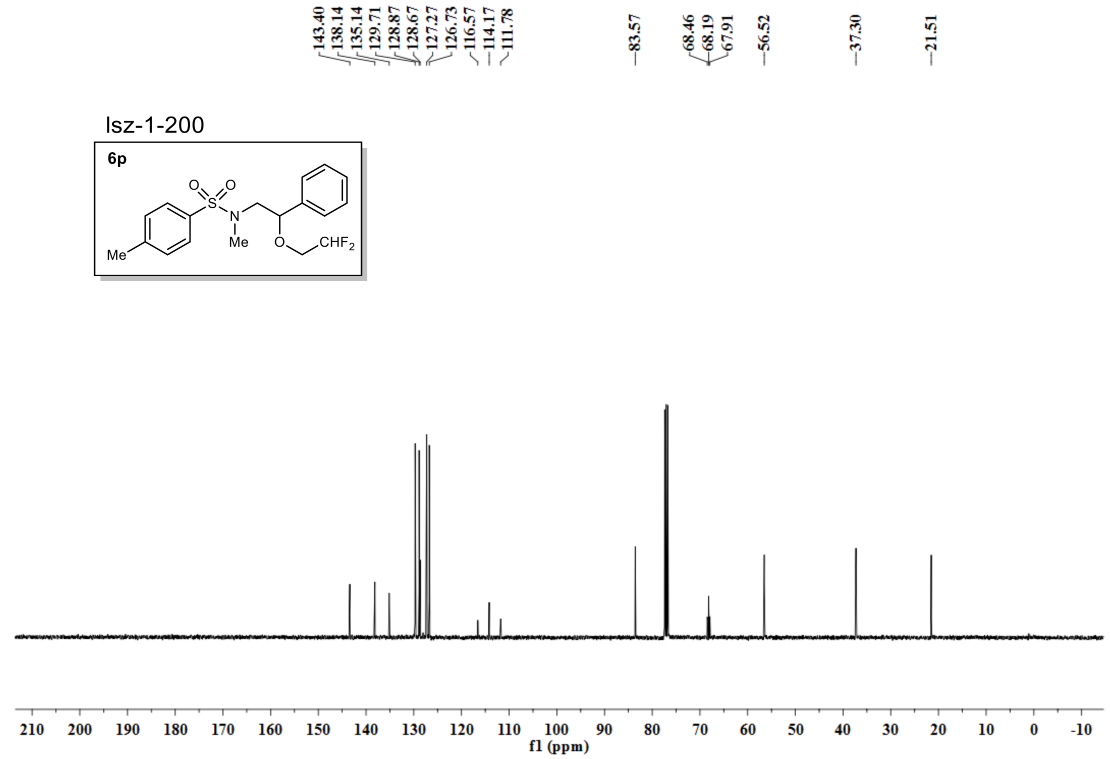

^{19}**F NMR spectrum of 6p** (377 MHz, $CDCl_3$):

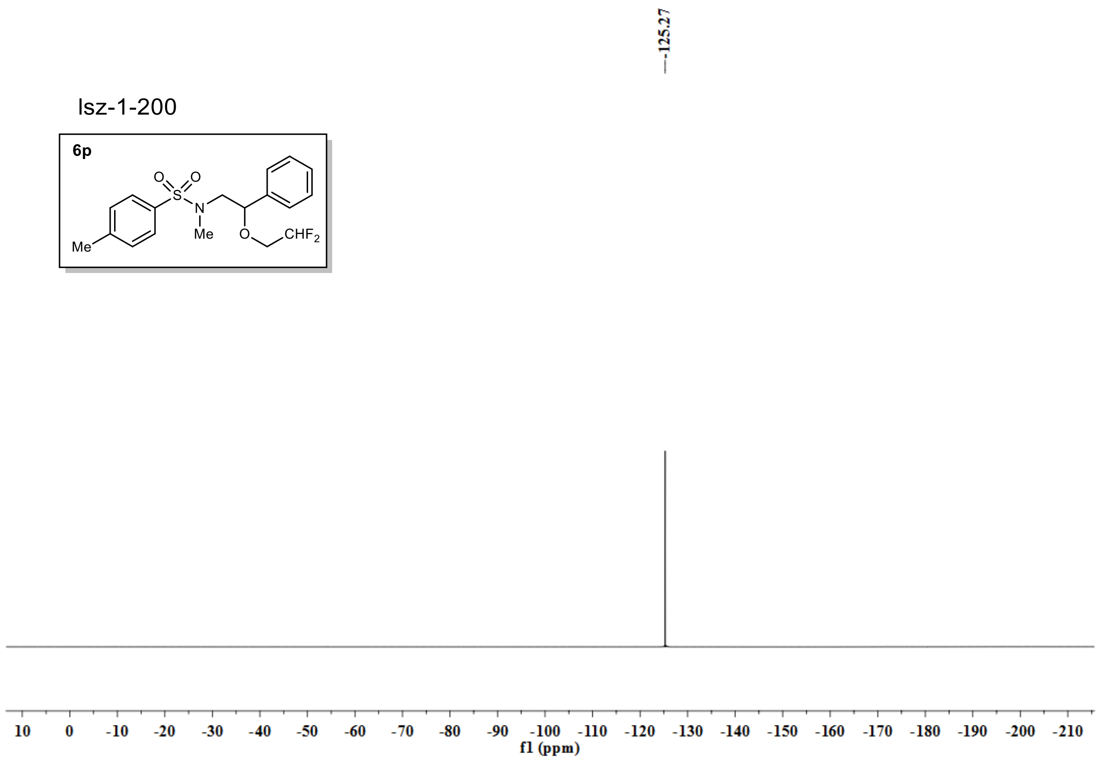

^{1}**H NMR spectrum of 6q** (400 MHz, $CDCl_3$):

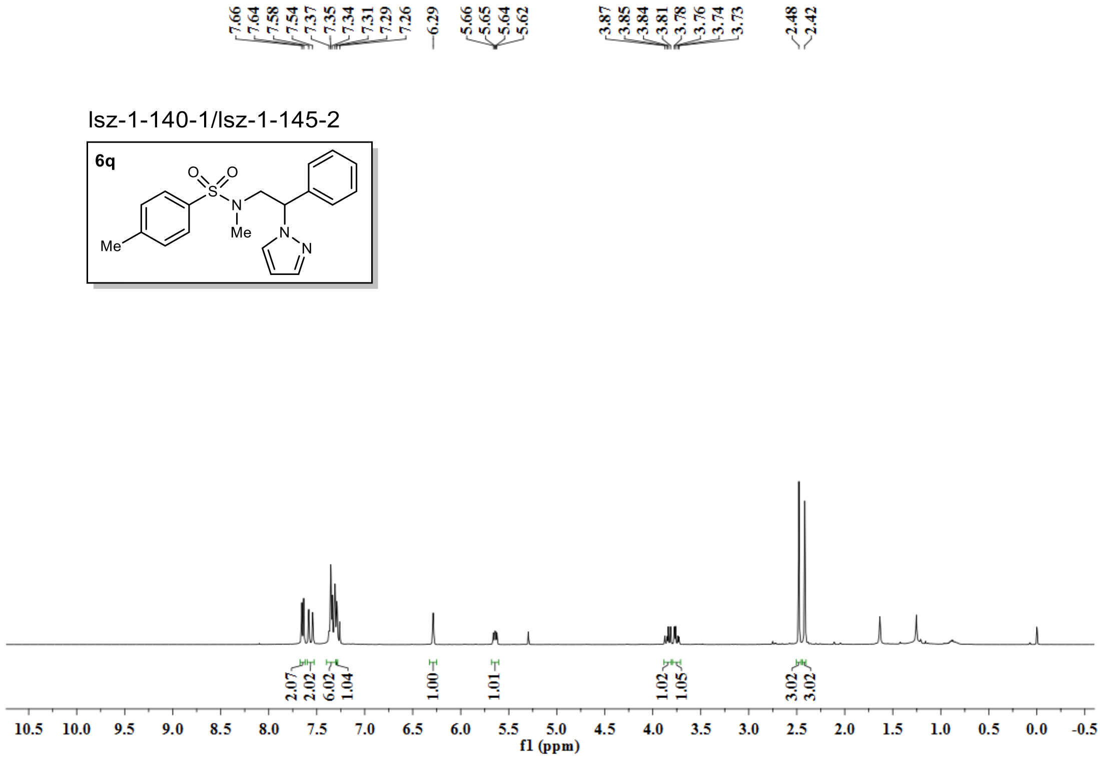

^{13}C NMR spectrum of 6q (101 MHz, $CDCl_3$):

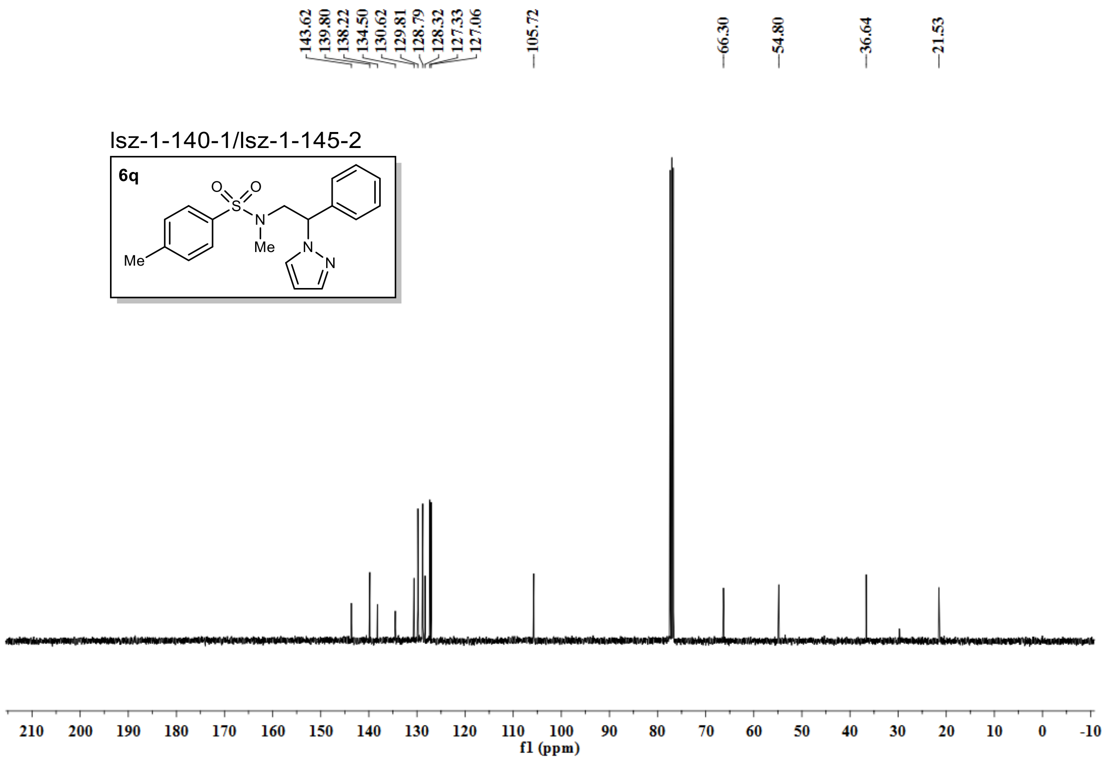

^{1}H NMR spectrum of 6r (400 MHz, $CDCl_3$):

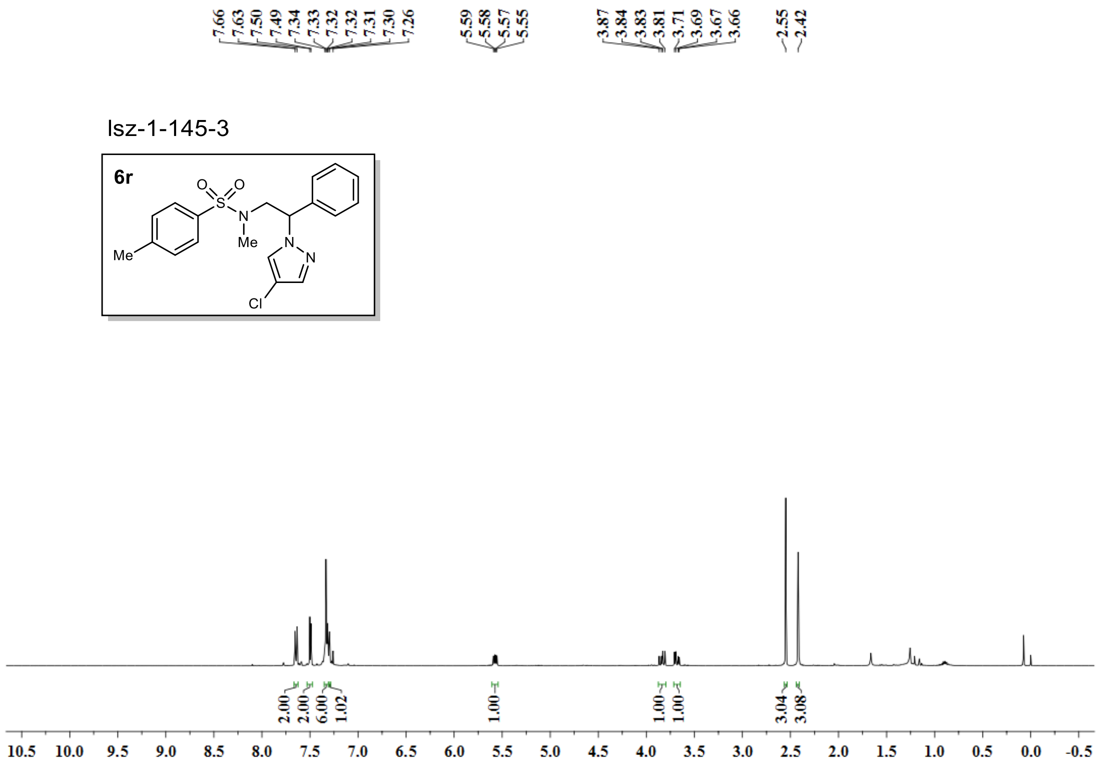

^{13}C NMR spectrum of 6r (101 MHz, $CDCl_3$):

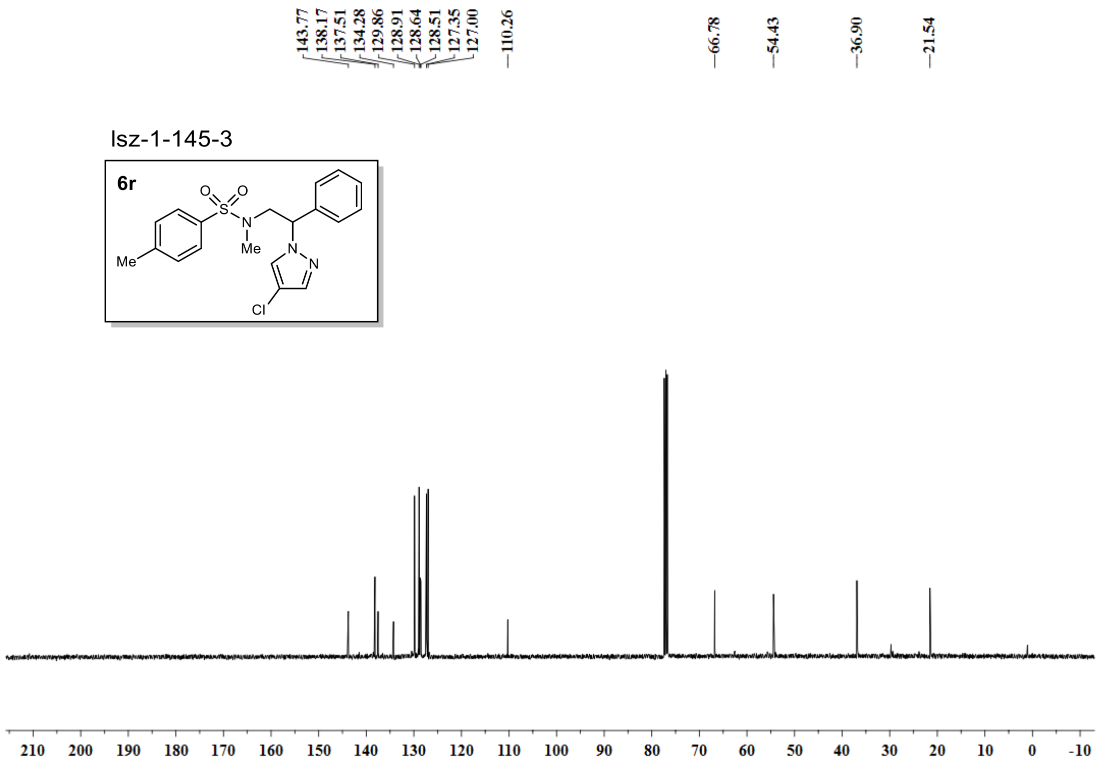

^{1}H NMR spectrum of 6s (400 MHz, $CDCl_3$):

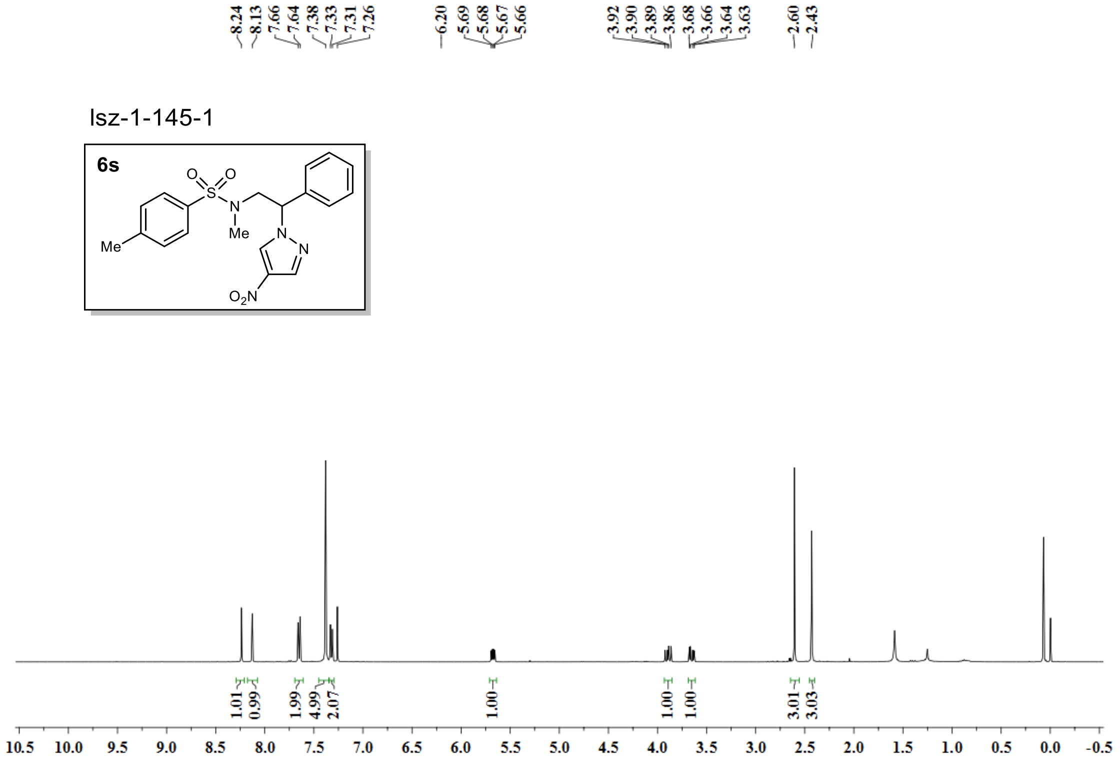

^{13}C NMR spectrum of 6s (101 MHz, $CDCl_3$):

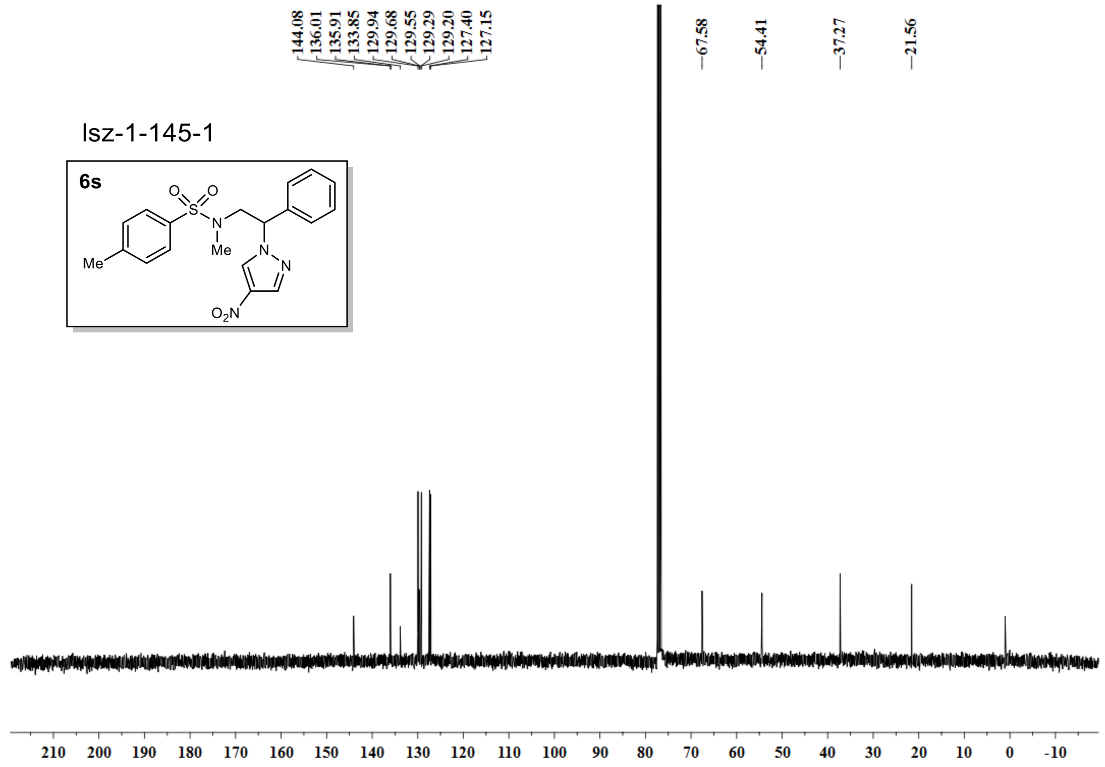

^{1}H NMR spectrum of 8 (400 MHz, $CDCl_3$):

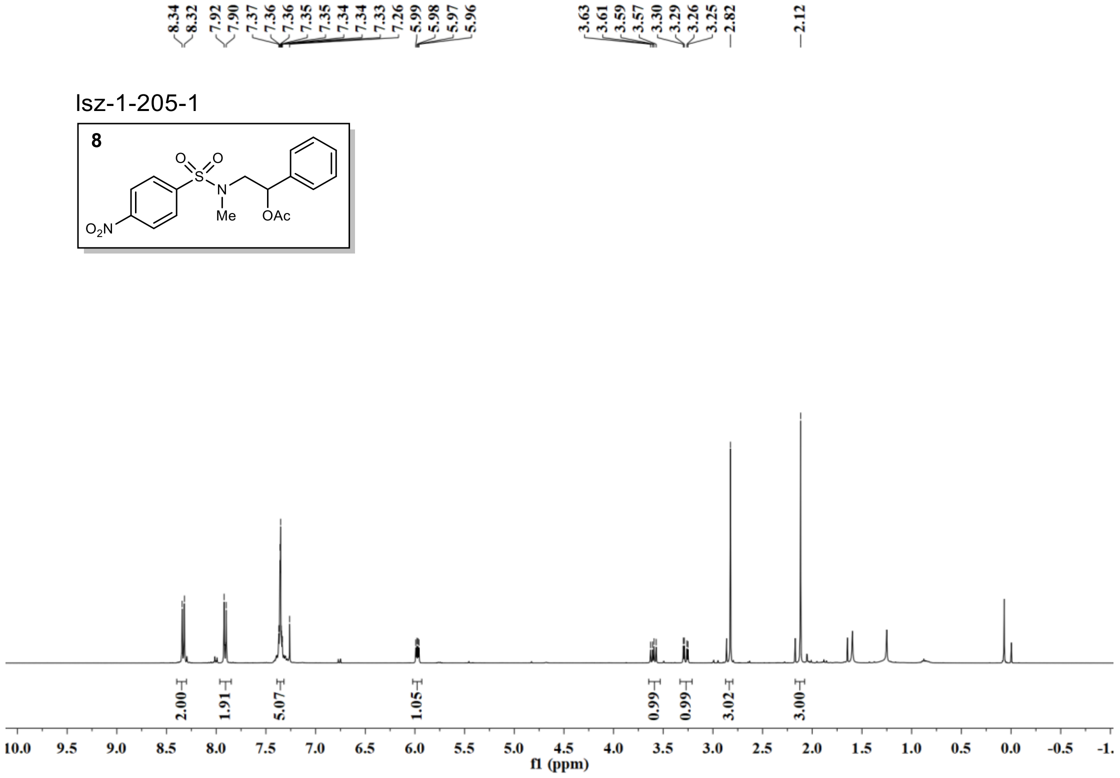

^{13}C NMR spectrum of 8 (101 MHz, $CDCl_3$):

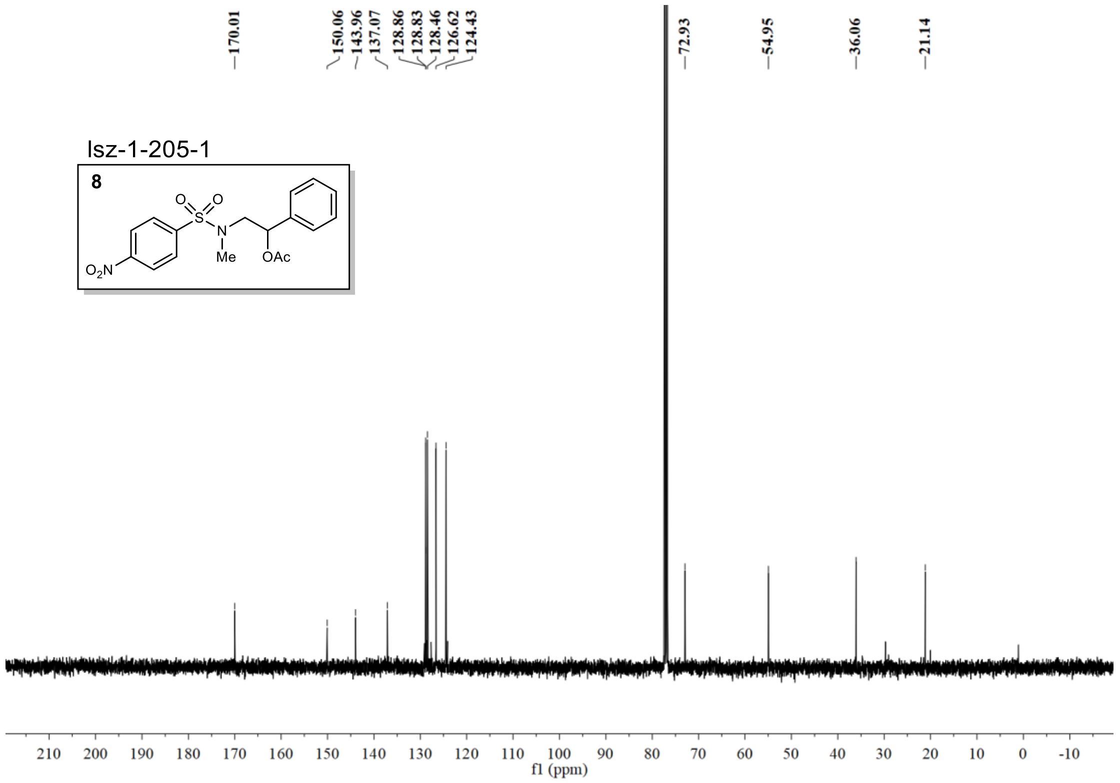

^{1}H NMR spectrum of 9 (400 MHz, $CDCl_3$):

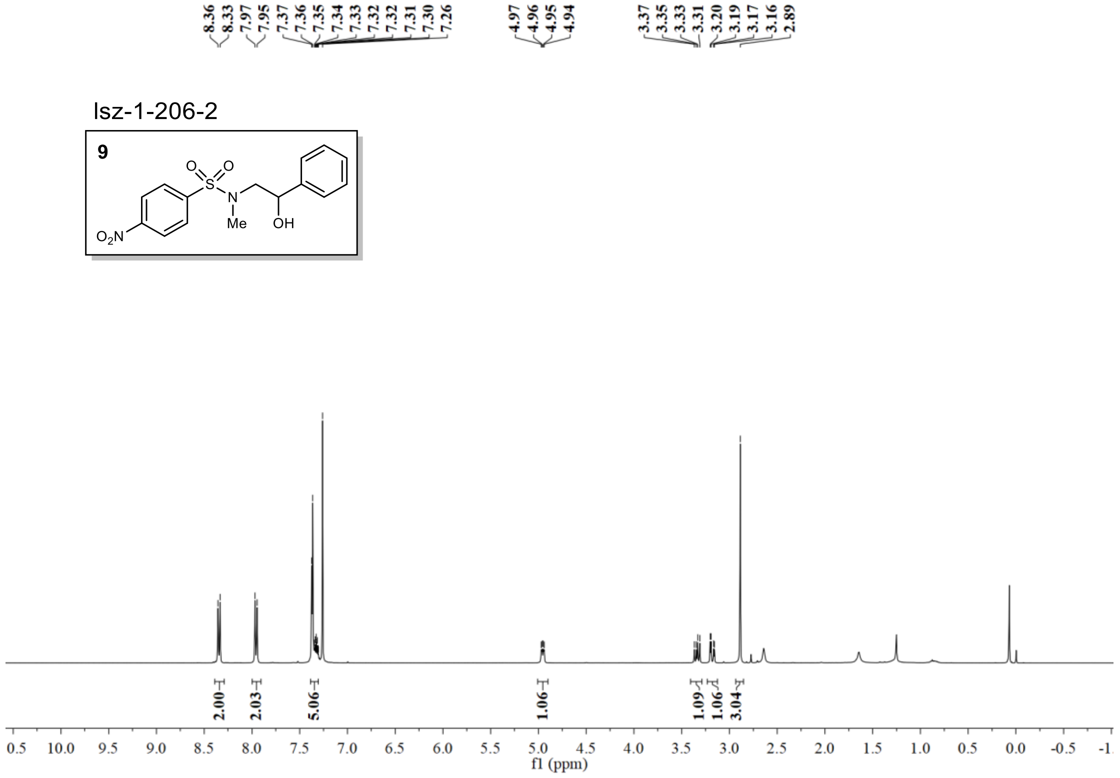

^{13}C NMR spectrum of 9 (101 MHz, $CDCl_3$):

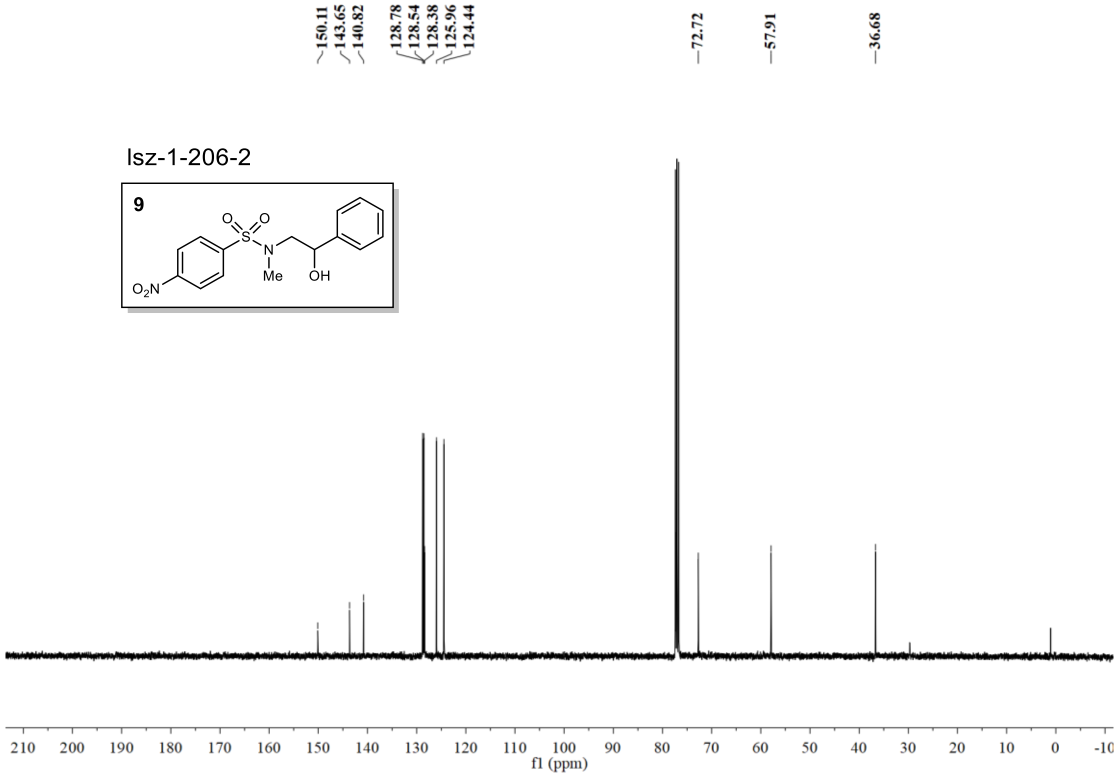

^{1}H NMR spectrum of 10a (400 MHz, $CDCl_3$):

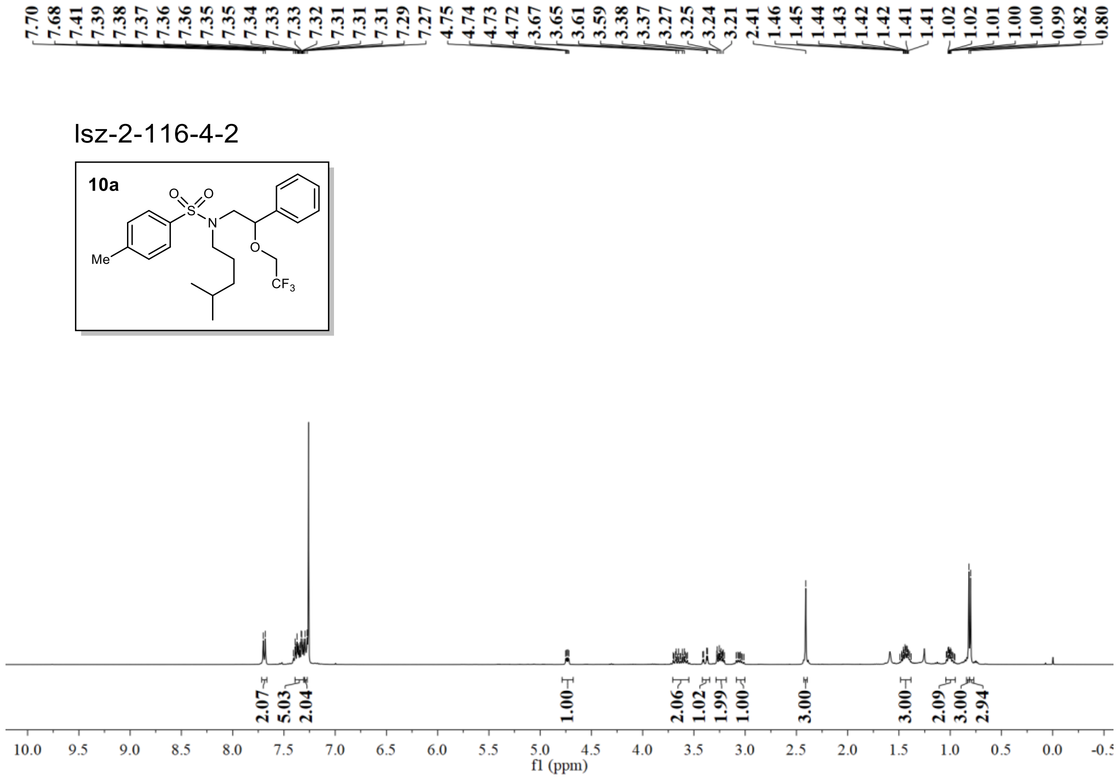

^{13}C NMR spectrum of 10a (101 MHz, $CDCl_3$):

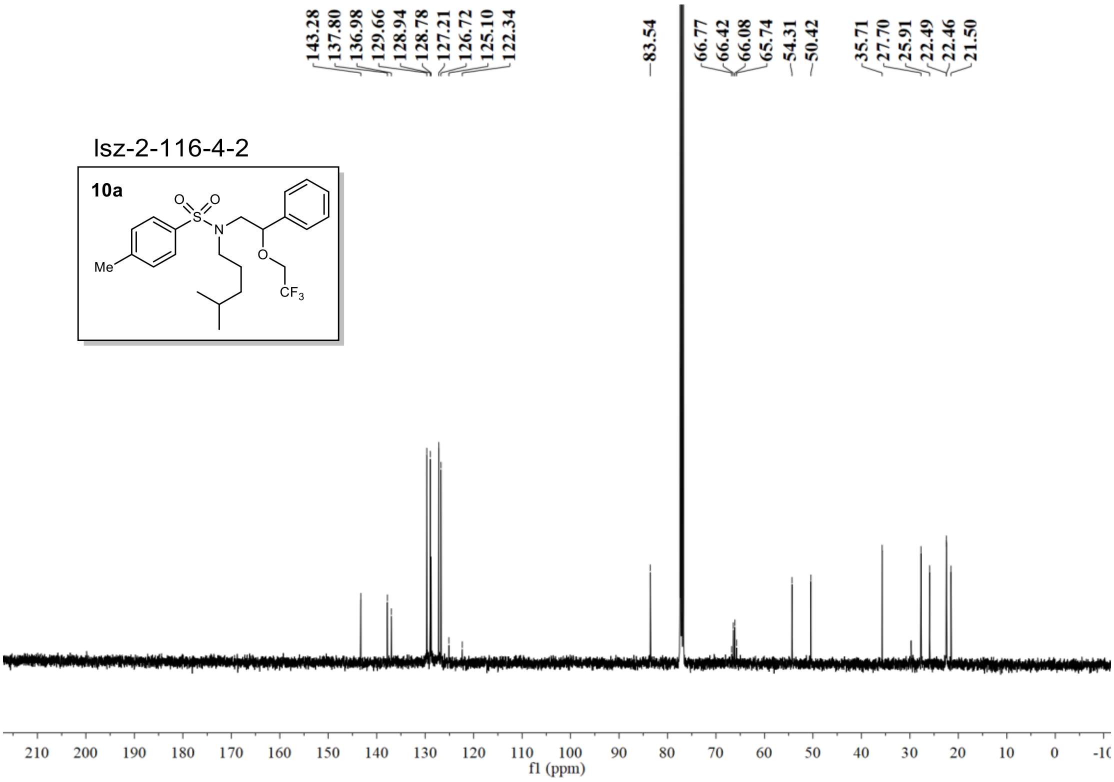

^{19}F NMR spectrum of 10a (377 MHz, $CDCl_3$):

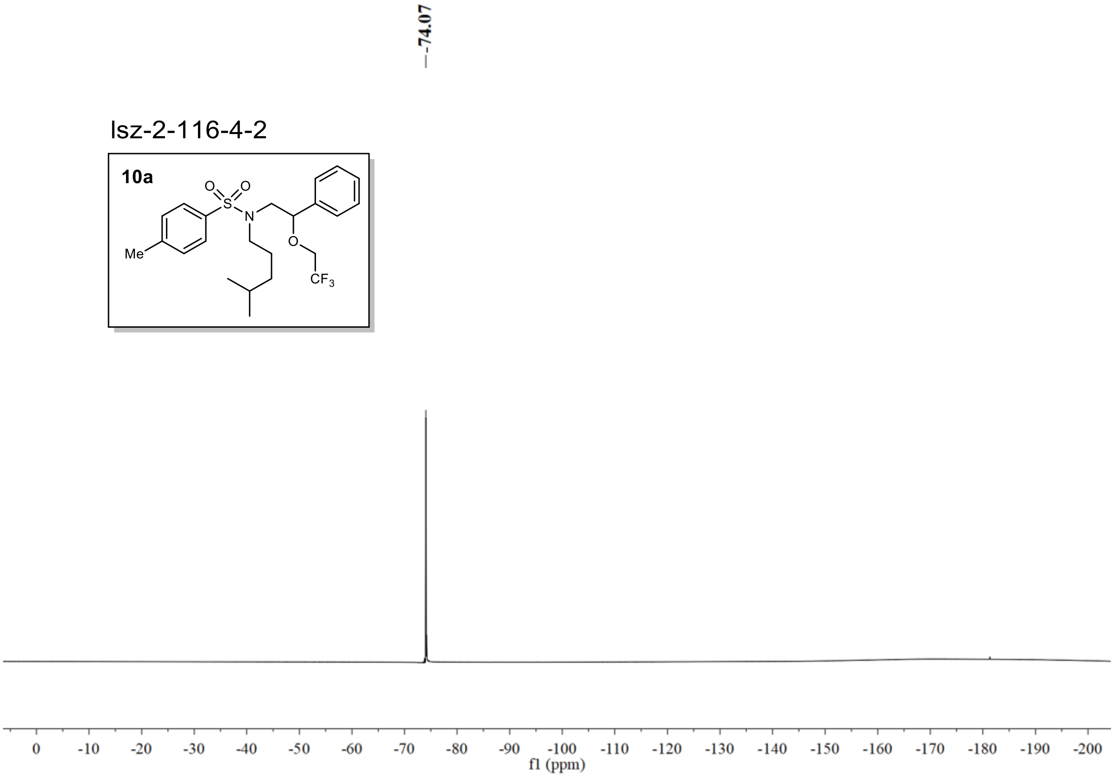

^{1}H NMR spectrum of 10b (400 MHz, $CDCl_3$):

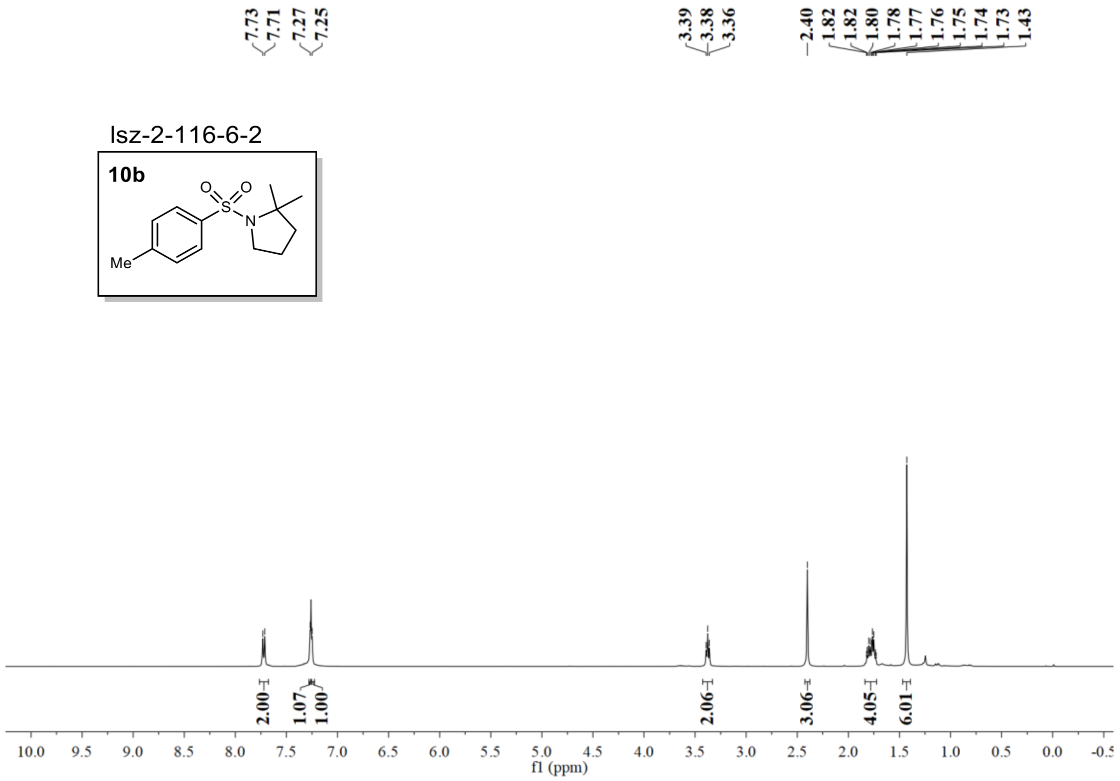

^{13}C NMR spectrum of 10b (101 MHz, $CDCl_3$):

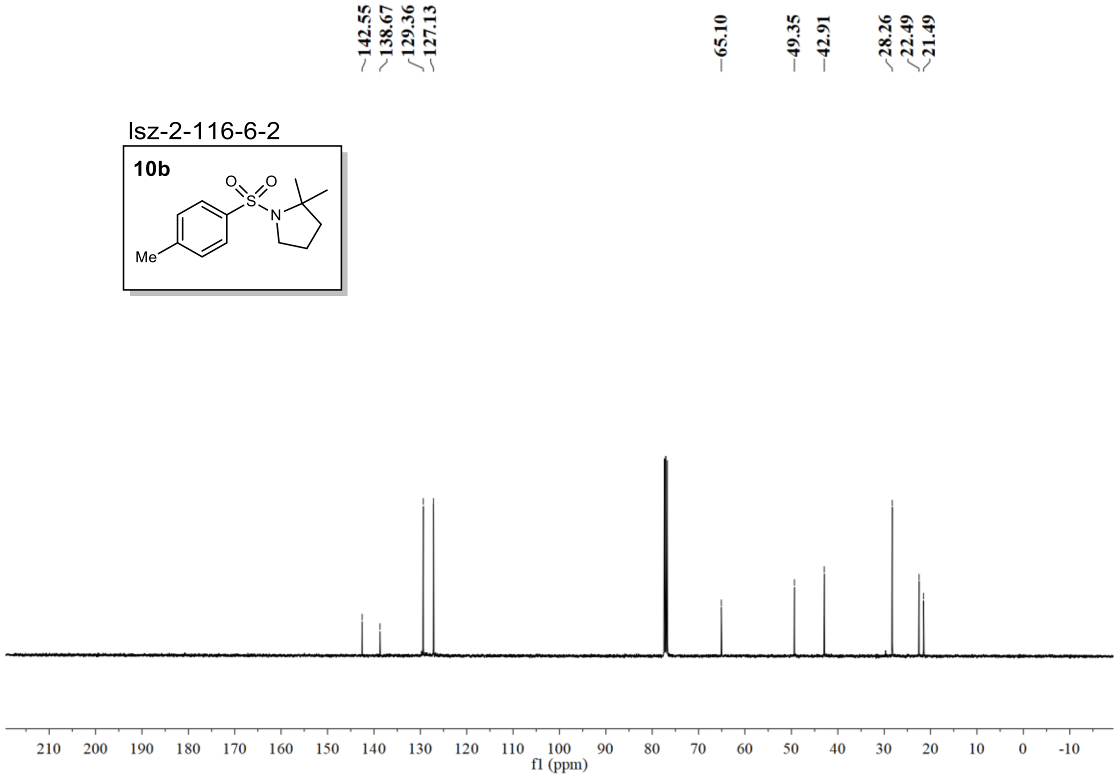

^{1}H NMR spectrum of 11a (400 MHz, $CDCl_3$):

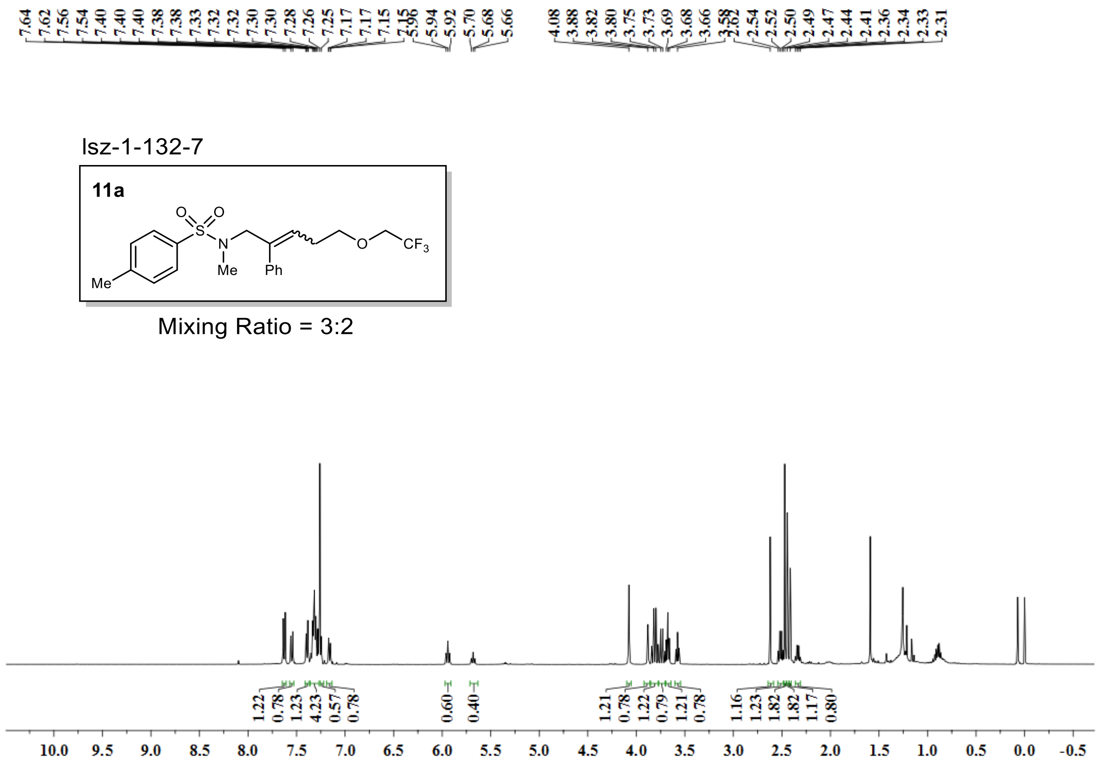

^{13}C NMR spectrum of 11a (101 MHz, $CDCl_3$):

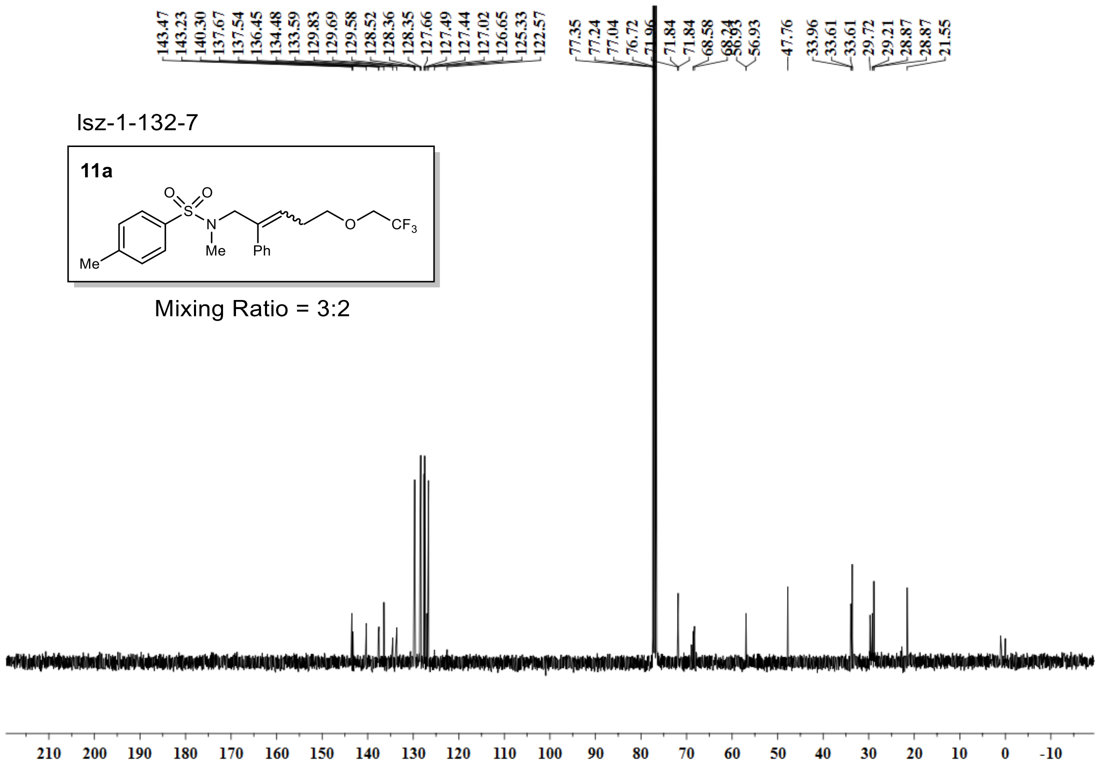

^{19}F NMR spectrum of 11a (377 MHz, $CDCl_3$):

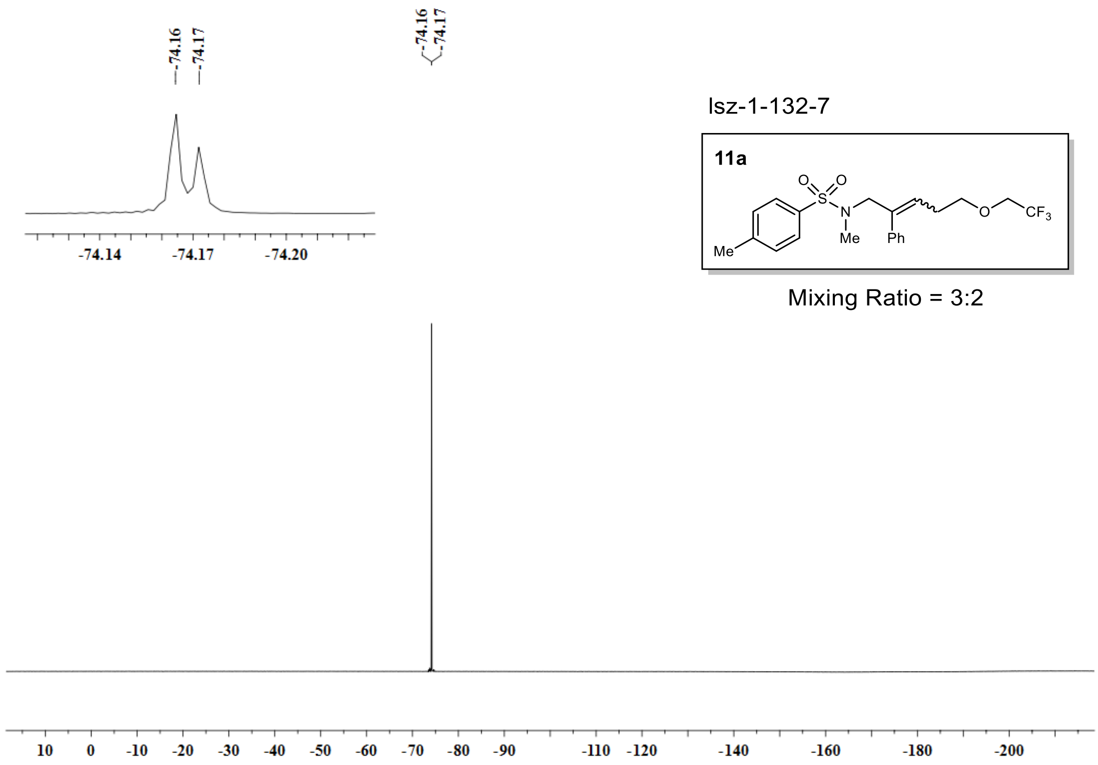

^{1}H NMR spectrum of 11b (400 MHz, $CDCl_3$):

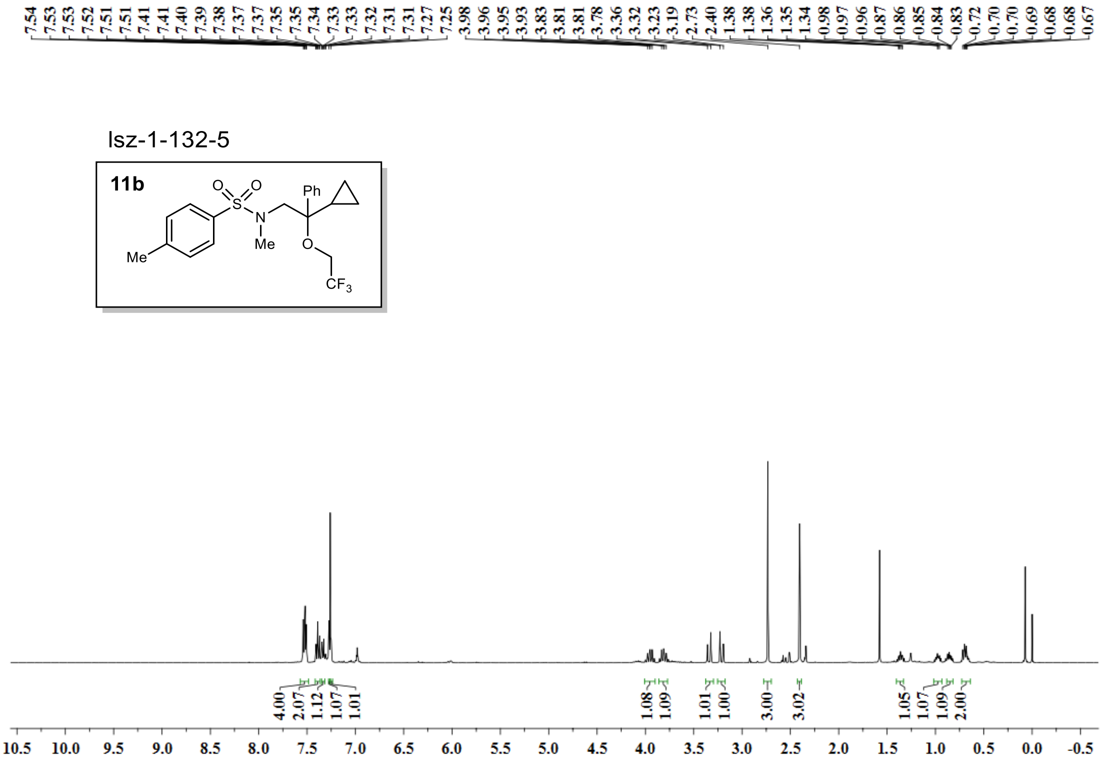

^{13}C NMR spectrum of 11b (101 MHz, $CDCl_3$):

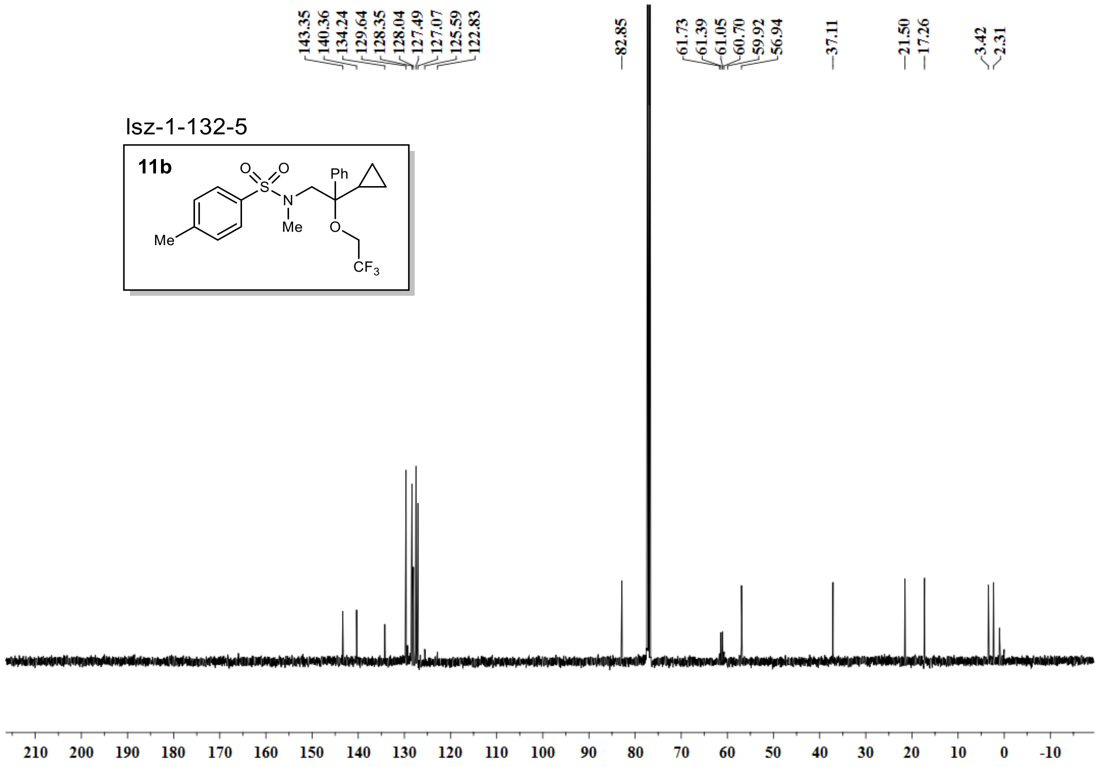

^{19}F NMR spectrum of 11b (377 MHz, $CDCl_3$):

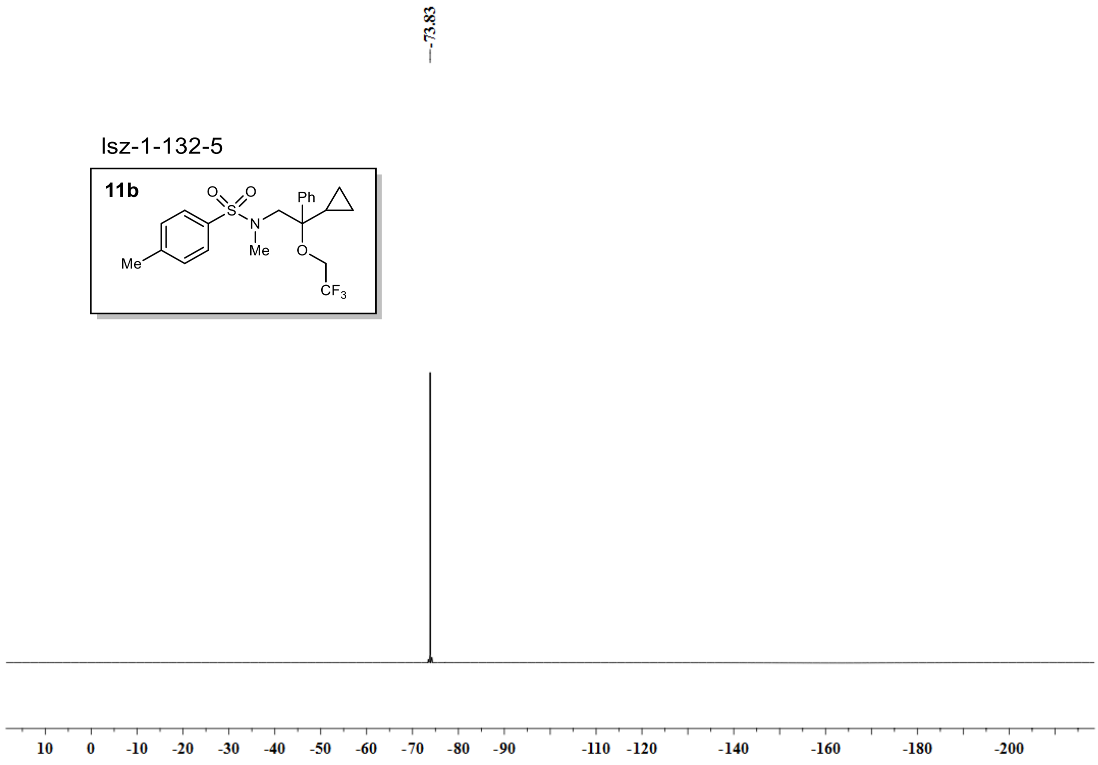

Supplementary References

1 Tan, Y. *et al.* Direct introduction of sulfonamide groups into quinoxalin-2(1*H*)-ones by Cu-catalyzed C3-H functionalization. *Chem. Asian J.* **15**, 3365-3369 (2020).

2 Dong, X., Sang, R., Wang, Q., Tang, X.-Y. & Shi, M. Copper-catalyzed trifluoromethylation and cyclization of aromatic-sulfonyl-group-tethered alkenes for the construction of 1,2-benzothiazinane dioxide type compounds. *Chem. Eur. J.* **19**, 16910-16915 (2013).

3 Zhang, H. *et al.* Highly enantioselective construction of fully substituted stereocenters enabled by *In Situ* phosphonium-containing organocatalysis. *ACS Catal.* **10**, 5698-5706 (2020).

4 Andna, L. & Miesch, L. Metal-free synthesis of activated ynesulfonamides and tertiary enesulfonamides. *Org. Biomol. Chem.* **17**, 5688-5692 (2019).

5 Polley, A., Bairy, G., Das, P. & Jana, R. Triple mode of alkylation with ethyl bromodifluoroacetate: *N*, or *O*-difluoromethylation, *N*-ethylation and *S*-(ethoxycarbonyl)difluoromethylation. *Adv. Synth. Catal.* **360**, 4161-4167 (2018).

6 Zhang, W., Xu, H., Xu, H. & Tang, W. DABCO-Catalyzed 1,4-bromolactonization of conjugated enynes: Highly stereoselective formation of a stereogenic center and an axially chiral allene. *J. Am. Chem. Soc.* **131**, 3832-3833 (2009).

7 Engl, S. & Reiser, O. Catalyst-free visible-light-mediated iodoamination of olefins and synthetic applications. *Org. Lett.* **23**, 5581-5586 (2021).
